# Stereoselective cobalt-catalyzed halofluoroalkylation of alkynes†
†Electronic supplementary information (ESI) available. See DOI: 10.1039/c7sc04916a


**DOI:** 10.1039/c7sc04916a

**Published:** 2018-01-05

**Authors:** Guojiao Wu, Axel Jacobi von Wangelin

**Affiliations:** a Institute of Organic Chemistry , University of Regensburg , Universitaetsstr. 31 , 93053 Regensburg , Germany; b Department of Chemistry , University of Hamburg , Martin Luther King Pl. 6 , 20146 Hamburg , Germany . Email: axel.jacobi@chemie.uni-hamburg.de

## Abstract

Stereoselective additions of highly functionalized reagents to available unsaturated hydrocarbons are an attractive synthetic tool due to their high atom economy, modularity, and rapid generation of complexity. We report efficient cobalt-catalyzed (*E*)-halofluoroalkylations of alkynes/alkenes that enable the construction of densely functionalized, stereodefined fluorinated hydrocarbons. The mild conditions (2 mol% cat., 20 °C, acetone/water, 3 h) tolerate various functional groups, *i.e.* halides, alcohols, aldehydes, nitriles, esters, and heteroarenes. This reaction is the first example of a highly stereoselective cobalt-catalyzed halo-fluoroalkylation. Unlike related cobalt-catalyzed reductive couplings and Heck-type reactions, it operates *via* a radical chain mechanism involving terminal halogen atom transfer which obviates the need for a stoichiometric sacrificial reductant.

## Abstract 2

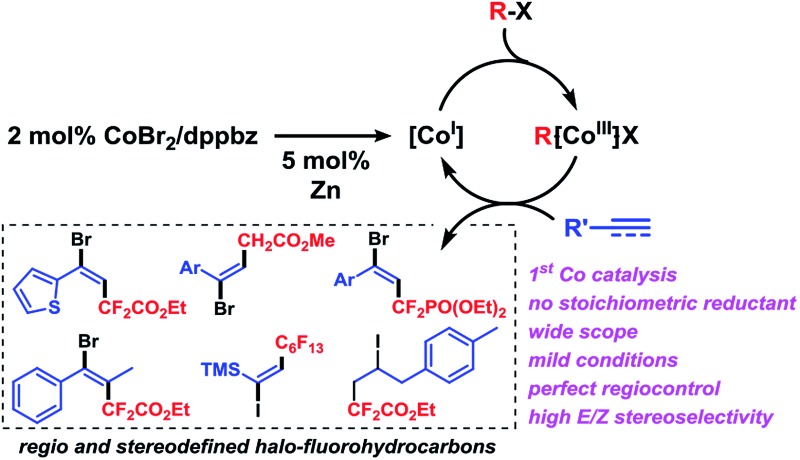
Stereoselective additions of functionalized reagents to unsaturated hydrocarbons are attractive due to the high atom economy, modularity and rapid generation of complexity. We report a stereoselective cobalt-catalyzed (*E*)-halofluoroalkylation of alkynes/alkenes that under mild conditions (2 mol% cat., 20 °C, acetone/water, 3 h). This reaction operates *via* a radical chain mechanism involving terminal halogen atom transfer which obviates the need for a stoichiometric sacrificial reductant.

## 1 Introduction

**Scheme 1:**
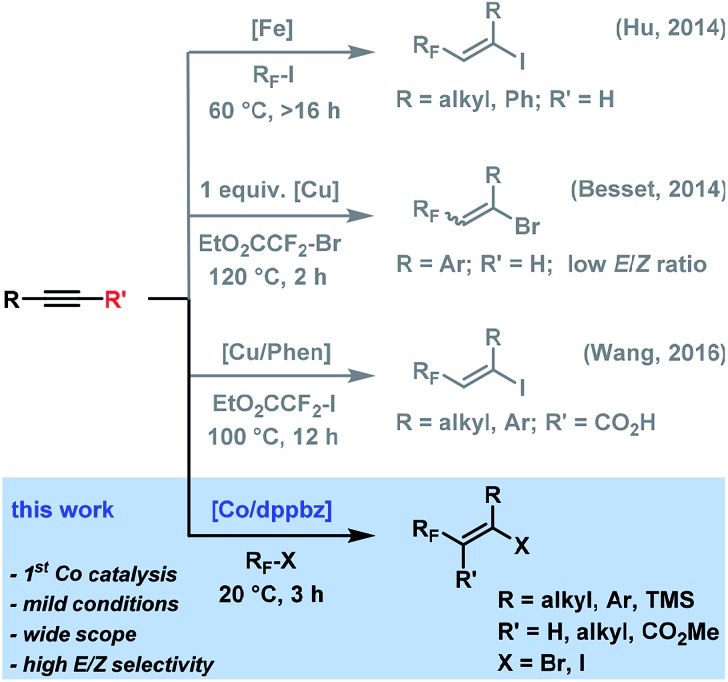
Metal-mediated halo-fluoroalkylations of alkynes.

Fluorinated hydrocarbons constitute key structural motifs in many bioactive molecules, agrochemicals, and pharmaceuticals due to their high metabolic stability, lipophilicity, and bioavailability compared with the parent compounds.^1^ Fluoroalkylation methods of easily accessible precursors have therefore attracted great interest in the past years.^2^,^3^ While many protocols are substitution processes that require highly pre-functionalized starting materials and produce unwanted by-products, direct additions to unsaturated hydrocarbons exhibit higher modularity and atom-efficiency and provide ample opportunities of regio- and stereocontrol. The addition of halo-fluoroalkanes to alkynes is an especially attractive tool due to the easy availability of the reagents and the great synthetic versatility of the resultant halo-fluoroalkenes. Many methods operate *via* an atom transfer radical addition (ATRA) mechanism in the presence of radical initiators (*e.g.* BEt_3_, AIBN, Na_2_S_2_O_3_ or light)^4^ that showed narrow substrate scope and poor selectivity. Mechanistically closely related transition metal-mediated halo-fluoroalkylations have been recently reported, but with a narrow focus on iodofluoro-alkylations and/or moderate stereo-control (Scheme 1). Hu *et al.* devised an iron-catalyzed addition of perfluoroalkyl iodide to alkynes with moderate to good *E*/*Z*-selectivities in the presence of Cs_2_CO_3_. The radical reaction with alkyl-substituted alkynes required long reaction times at 60 °C and could not convert perfluoroalkyl bromides.^5^ Besset *et al.* postulated a different mechanism for the copper-mediated synthesis of difluoromethyl alkenes from BrCF_2_CO_2_Et and alkynes. However, significantly lower stereoselectivities were obtained and stoichiometric amounts of copper salt were employed.^6^ Very recently, Wang and co-workers reported a copper-catalyzed decarboxylative ATRA reaction between ICF_2_CO_2_Et and substituted propiolic acids.^7^

Despite the developments of novel iron- and copper-catalyzed procedures, the reactions generally utilize expensive fluoroalkyl iodides as starting materials, high catalyst loadings, long reaction times, and high reaction temperatures. An efficient and robust yet highly stereoselective method that operates at mild conditions and low catalyst loadings and that is applicable to various fluoroalkyl halides would constitute a significant advancement of the current technology and have considerable use in the synthesis of densely functionalized fluorinated building blocks. To the best of our knowledge, there are no literature reports of ATRA reaction between alkyl halides and alkynes with low-valent cobalt catalysts. Here, we report the cobalt-catalyzed halofluoro-alkylation of alkynes which enables the highly regioselective and stereoselective synthesis of a diverse set of halofluoroalkenes under unprecedentedly mild conditions (Scheme 1).

**Scheme 2:**
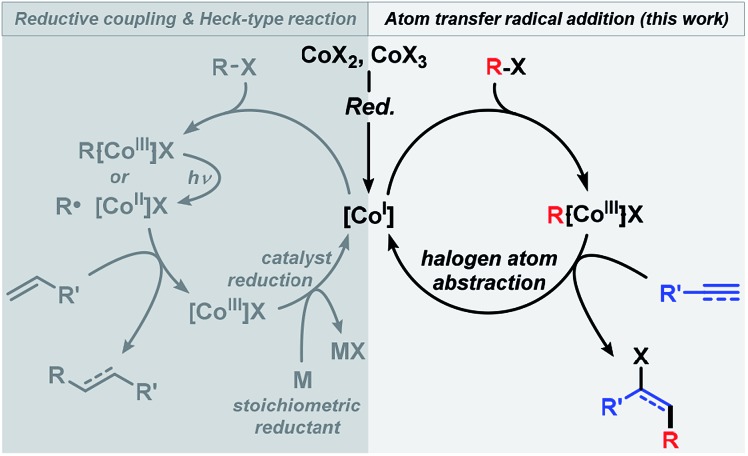
Generalized mechanistic dichotomy of radical addition reactions under low-valent cobalt catalysis.

The utility of alkyl halides in cross-couplings and reductive additions has recently been greatly enhanced by the development of low-valent iron group metal catalysts (Fe, Co, Ni)^8^–^10^ that engage in facile alkyl-X activation. The high propensity of late 3d transition metals to undergo single-electron transfer (SET) processes often results in the intermediacy of carbon-centered radical species.8g,h,9h,10f The reductive formation of alkyl radicals from alkyl-X electrophiles is thermodynamically favoured when the formal electron septet-carbon is stabilized by heteroatoms, conjugation, hyperconjugation, or inductive effects. Efficient cobalt catalysts have been reported for several reductive coupling reactions between alkyl halides and Michael-type acceptors as well as for Heck-type reactions between alkyl halides and alkenes.^11^ These processes mostly follow the same mechanistic scenarios involving (i) initial formation of a low-valent Co(i) species from a Co(ii,iii) precursor in the presence of a reductant; (ii) reductive cleavage of the alkyl halide to give an alkyl radical R˙ and a Co(ii) complex (by SET activation or homolysis of R–Co(iii)), (iii) addition of R˙ to the olefin and formation of an organocobalt species that is subject to disproportionation (Heck-type reaction) or hydrolysis (reductive coupling) to release the Co(iii) complex, (iv) regeneration of the Co(i) catalyst with a stoichiometric reductant (Scheme 2, left). We surmised that an ATRA reaction between alkyl halides and alkynes could follow a similar mechanism using low-valent Co catalysts but would require only catalytic amounts of a reductant (Scheme 2, right). Cobalt-catalyzed Heck-type and reductive coupling reactions were realized in the presence of stoichiometric reductants such as Zn, Mn, and Grignard reagents^11^ which effect the Co(iii) → Co(i) reduction. Cobalt-catalyzed ATRA reactions of alkynes with alkyl halides have not been reported.

## 2 Results and discussion

### 2.1 Discovery of a cobalt-catalyzed ATRA reaction

We commenced our investigations with the reaction of phenyl-acetylene (**1a**) and ethyl bromodifluoroacetate (**2a**). Variations of reaction conditions, solvents, and catalysts led to an optimized procedure that utilized a three-component catalyst comprising of CoBr_2_, dppbz (1,2-bis(diphenylphosphino)benzene) and zinc in acetone/water at 20 °C to furnish the synthesis of the desired adduct ethyl 4-bromo-2,2-difluoro-4-phenylbut-3-enoate (**3a**) in 83% yield (87% GC yield, Table 1, entry 1). Significantly lower yields were obtained when replacing dppbz with other bidentate phosphines or 2,2′-bipyridine (entries 5–8). Other transition metals were inactive (entries 9–12). CoCl_2_ and CoCl_2_·4H_2_O exhibited similar activity (entries 13, 14).

**Table 1:** Selected optimization experiments^*a*^

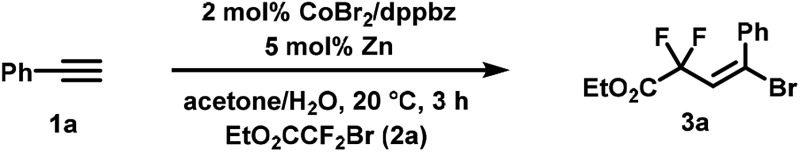		
Entry	Deviations from conditions above	**3a** ^*b*^ [%]
**1**	**None**	**87** (63)^*c*^
2	Without H_2_O	69
3	MeCN instead of acetone	65
4	Without CoBr_2_ or Zn or dppbz	0
5	dppe instead of dppbz	29^*d*^
6	dppen instead of dppbz	31^*d*^
7	dppf instead of dppbz	0^*d*^
8	bipy instead of dppbz	0^*d*^
9	FeBr_2_ instead of CoBr_2_	0^*d*^
10	NiCl_2_ instead of CoBr_2_	0^*d*^
11	CrCl_3_ instead of CoBr_2_	0^*d*^
12	Cp_2_TiCl_2_ instead of CoBr_2_	0^*d*^
13	CoCl_2_ instead of CoBr_2_	82^*d*^
14	CoCl_2_·4H_2_O instead of CoBr_2_	83^*d*^
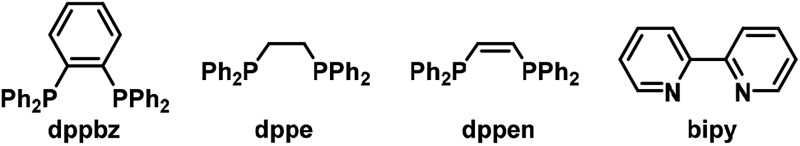		

^*a*^Conditions: **1a** (0.3 mmol), **2a** (0.45 mmol), CoBr_2_ (2 mol%), dppbz (2 mol%), Zn (5 mol%), 0.6 mL acetone/H_2_O, 20 °C, 3 h.

^*b*^GC yield *vs.* internal *n*-dodecane.

^*c*^1 mol% CoBr_2_, 1 mol% dppbz, 10 mol% Zn.

^*d*^5 mol% CoBr_2_, 5 mol% dppbz, 20 mol% Zn.

### 2.2 Substrate scope

**Scheme 3:**
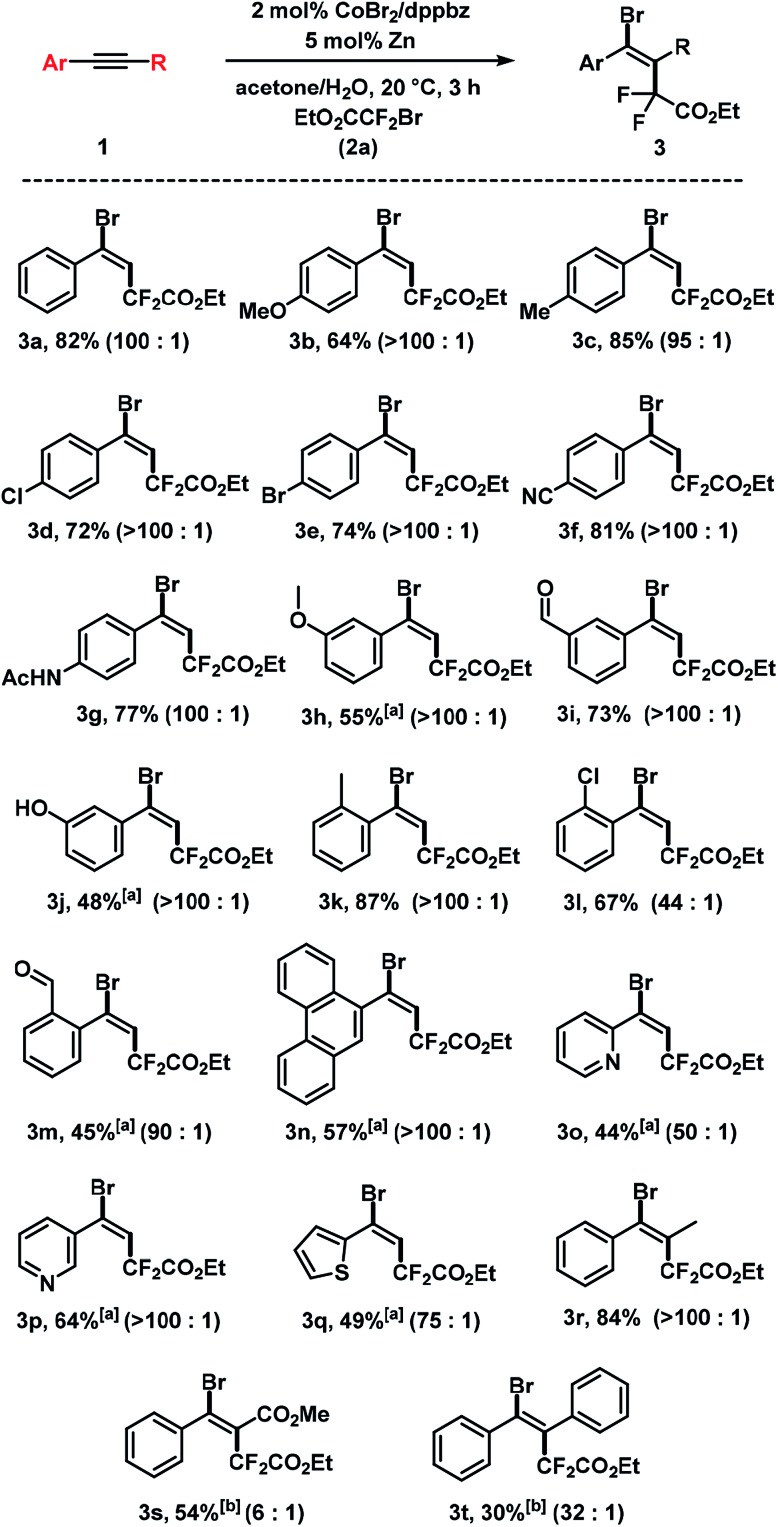
Cobalt-catalyzed bromo-carboxydifluoromethylation of alkynes. Standard conditions: **1** (0.3 mmol), **2a** (0.45 mmol), CoBr_2_ (2 mol%), dppbz (2 mol%) and Zn (5 mol%), 0.6 mL acetone/H_2_O, 20 °C, 3 h under N_2_. Isolated yields are given; *E*/*Z* ratios in parentheses (by ^19^F NMR). ^[a]^ 20 mol% Zn. ^[b]^ 40 mol% Zn, 8 h.

**Scheme 4:**
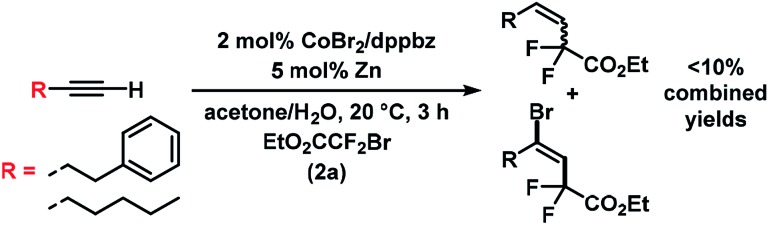
Reactions with alkyl-substituted terminal alkynes.

The substrate scope of reactions between terminal and internal aryl acetylenes and **2a** (Scheme 3). Many substitution patterns were tolerated (*ortho*, *meta*, *para*, electron-withdrawing, electron-donating substituents). The reaction displayed remarkable compatibility with functional groups including aldehydes, halides, nitriles, amides, hydroxyl, pyridines, thiophenes. All products were obtained with perfect regiocontrol and high *E*/*Z* diastereo-selectivity (>50/1). However, alkyl-substituted terminal alkynes fared poorer (Scheme 4). Reactions of **2a** with 1-heptyne and 4-phenyl-1-butyne, respectively, afforded mixtures of bromo-difluoro-alkylation and hydrodifluoroalkylation products in low yields.

**Scheme 5:**
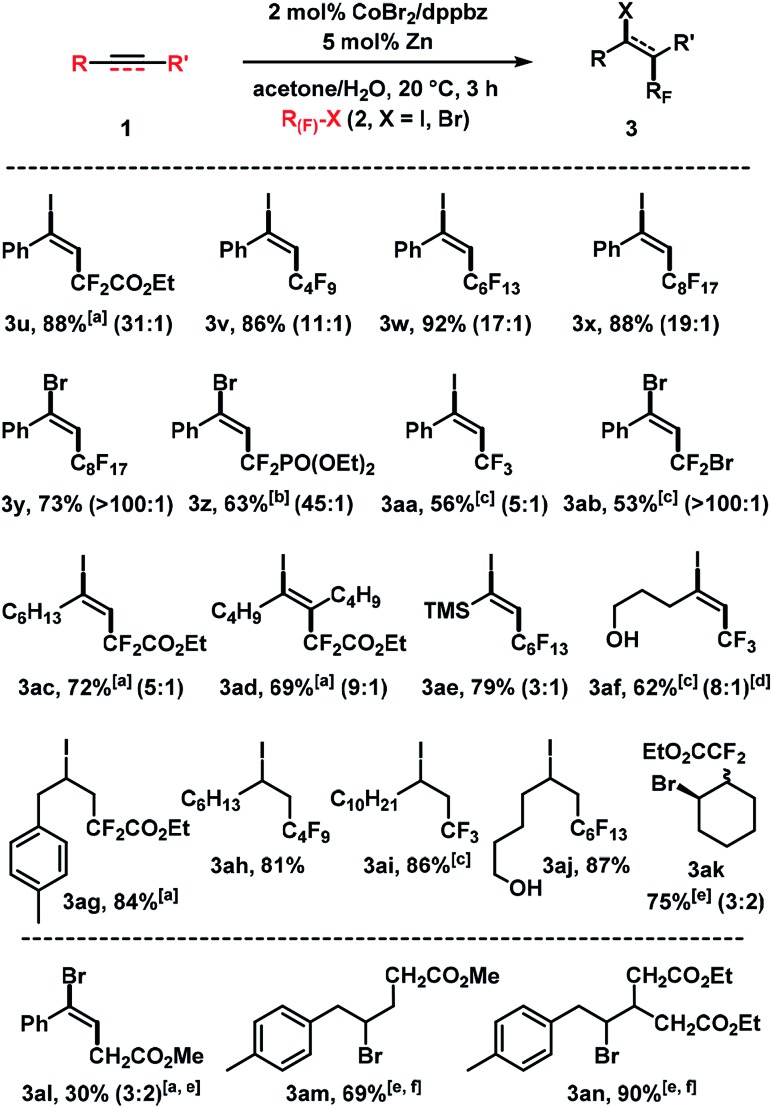
Cobalt-catalyzed halo-fluoroalkylation of alkynes and alkenes. Standard conditions: **1** (0.3 mmol), **2** (0.45 mmol), CoBr_2_ (2 mol%), dppbz (2 mol%), Zn (5 mol%), 0.6 mL acetone/H_2_O, 20 °C, 3 h under N_2_. Isolated yields given; *E*/*Z* ratios in parentheses (by ^19^F NMR). ^[a]^ 20 mol% Zn. ^[b]^ R_F_–Br (1.0 equiv.), 10 mol% Zn. ^[c]^ R_F_–X (3.0 equiv.), 10 mol% Zn. ^[d]^*E*/*Z* ratio of isolated products. ^[e]^ 10 mol% Zn. ^[f]^ 6 h.

We then examined the cobalt-catalyzed halofluoroalkylation with different fluoroalkyl halides (Scheme 5). Iododifluoroacetate ICF_2_CO_2_Et, perfluoroalkyl iodides such as C_4_F_9_I, C_6_F_13_I and C_8_H_17_I, and the perfluoroalkyl bromide C_8_F_17_Br were competent electrophiles which afforded the desired adducts in good to excellent yields. The fluoroalkyl bromides gave generally better *E*/*Z* selectivities than the iodides. While this trend is in full agreement with the literature, it can now be harnessed at much milder conditions (room temp., 2 mol% catalyst, 3 h). BrCF_2_PO(OEt)_2_, CF_3_I, and CF_2_Br_2_ afforded slightly lower yields; the reaction with CF_3_I exhibited low stereocontrol. Reactions of alkyl-substituted alkynes with fluoroalkyl iodides gave good yields and moderate *E*/*Z* selectivities (**3ac–3af**). The reaction conditions were also applied to reactions of (cyclo)alkenes with halofluoroacetates (**3ag–3ak**). A method extension to reactions of simple bromo-acetates with alkenes gave the desired adducts **3al–3an**.

**Scheme 6:**
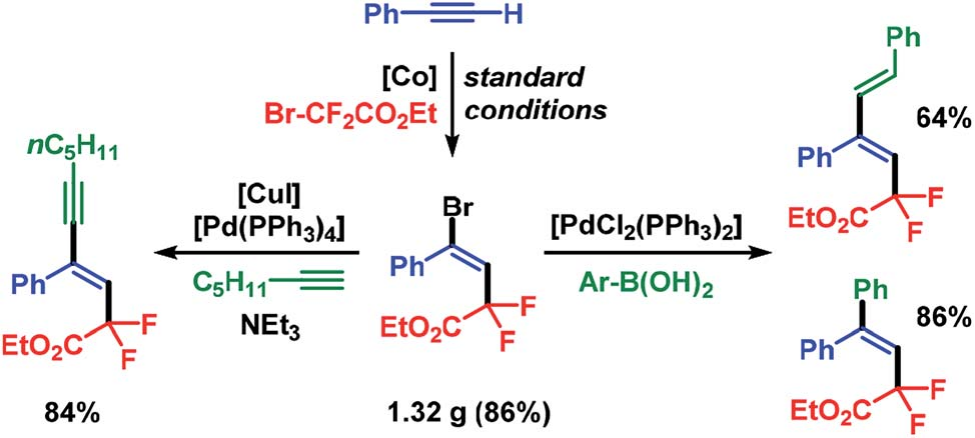
Post-ATRA transformations by cross-coupling reactions.

The synthetic utility of the stereoselective cobalt-catalyzed halo-fluoroalkylation protocol was demonstrated in a gram-scale setup which delivered pure ethyl (*E*)-4-bromo-2,2-difluoro-4-phenyl-but-3-enoate (**3a**) in 86% isolated yield (1.32 g) after 3 h. Substitution of the Br substituent in **3a** by Sonogashira and Suzuki cross-coupling reactions, respectively, afforded the fluorinated alkenes in very good yields and with complete retention of stereochemistry (Scheme 6).

### 2.3 Mechanistic studies

**Scheme 7:**
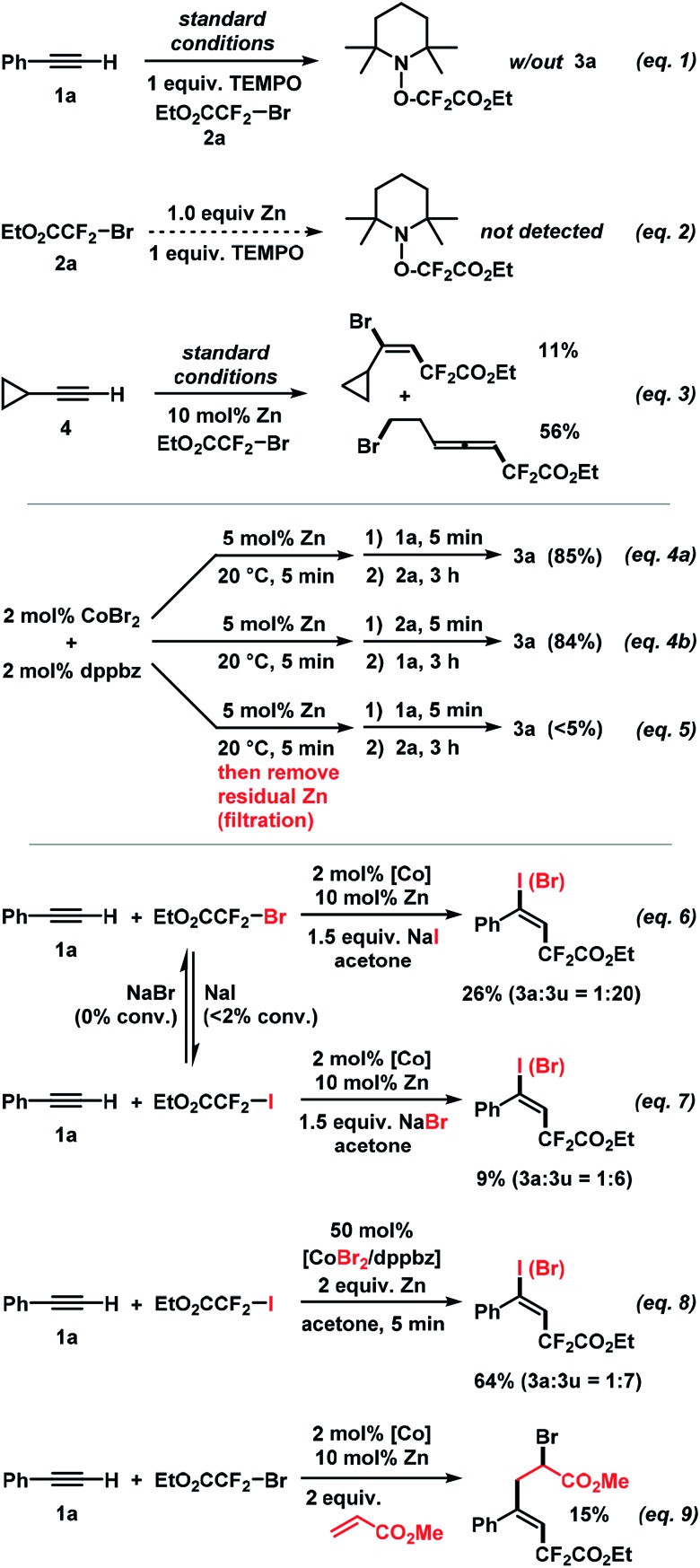
Key mechanistic experiments.

Further attention was devoted to the study of the reaction mechanism. In addition to the initial optimization reactions (Table 1), key mechanistic experiments were conducted. The model reaction between **1a** and **2a** was completely inhibited in the presence of 2,2,6,6-tetramethyl-1-piperidinyloxy (TEMPO). The TEMPO–CF_2_CO_2_Et adduct was observed by mass spectrometry (Scheme 7, eqn (1)). The TEMPO–CF_2_CO_2_Et adduct was not detected when treating **2a** with equimolar Zn which supports the notion that the SET reduction of the alkyl halide is induced by the cobalt catalyst (Scheme 7, eqn (2)). Upon employment of cyclo-propylacetylene (**4**), the vinylcyclopropane product was formed in 11% yield while ring-opening to the 7-bromohepta-3,4-dienoate (56%) was the major pathway. This is in full agreement with the intermediacy of an internal vinyl radical formed by radical addition of EtO_2_CCF_2_˙ to the alkyne (Scheme 7, eqn (3)). Identical rates and yields were observed in reactions where **1a** and **2a** were successively added to the catalyst solution. The reverse order of addition (**2a**, then **1a**) gave an identical result. Importantly, no product was formed when prior to the addition of **1a** and **2a** – the catalyst suspension (CoBr_2_, dppbz, Zn) was filtered (to remove residual zinc) or when the supernatant solution was decanted into a new reaction vessel (eqn (4)). These experiments suggest that the initially formed Co(i) species alone cannot catalyze the reaction but requires the presence of zinc, at least for the first turnover of the catalytic mechanism. Zn is employed only in catalytic amounts (5 mol%, *i.e.* 2.5 equiv. per Co)! The addition of sodium iodide and sodium bromide, respectively, shed light on the nature of the operating halogen transfer. **1a** and **2a** reacted with added NaI (1.5 equiv.) to give the iodo adduct **3u** as major product (**3a** : **3u** = 1 : 20, Scheme 7, eqn (6)). With NaBr added, the reaction between **1a** and **2b** gave **3a** and **3u** in a 1 : 6 ratio (Scheme 7, eqn (7)). Control experiments documented that no EtO_2_CCF_2_I was converted into EtO_2_CCF_2_Br using NaBr as additive; only minimal amounts of EtO_2_CCF_2_Br (<2%) were converted to EtO_2_CCF_2_I with NaI as additive under the same conditions. A similar outcome was observed when the standard reaction was performed with 50 mol% CoBr_2_/dppbz (**3a** : **3u** = 1 : 7, Scheme 7, eqn (8)). These experiments document that the halogen atom X in the product does not originate from the electrophilic R_F_X *via* a direct radical chain transfer but is transferred from the cobalt catalyst. This is a fine but important distinction from previously reported ATRA reactions that all involved halogen transfer from R_F_X to the vinyl radical. This has great implications for catalyst design and reaction development as the thermodynamics and kinetics of the halogen atom transfer step are no longer depending on the nature of the employed substrates but can be finely tuned through the stereoelectronic properties of the catalyst. We further believe that halogen atom transfer to a vinyl radical intermediate (rather than a vinyl cation) is operative: (i) the addition of water (as a nucleophile) resulted in no product bearing oxo functions; (ii) the presence of methyl acrylate as a radical acceptor led to the formation of the heptene-1,7-dioate *via* radical insertion of the acrylate (eqn (9)). A cationic intermediate would not add to this Michael acceptor.

**Fig. 1:**
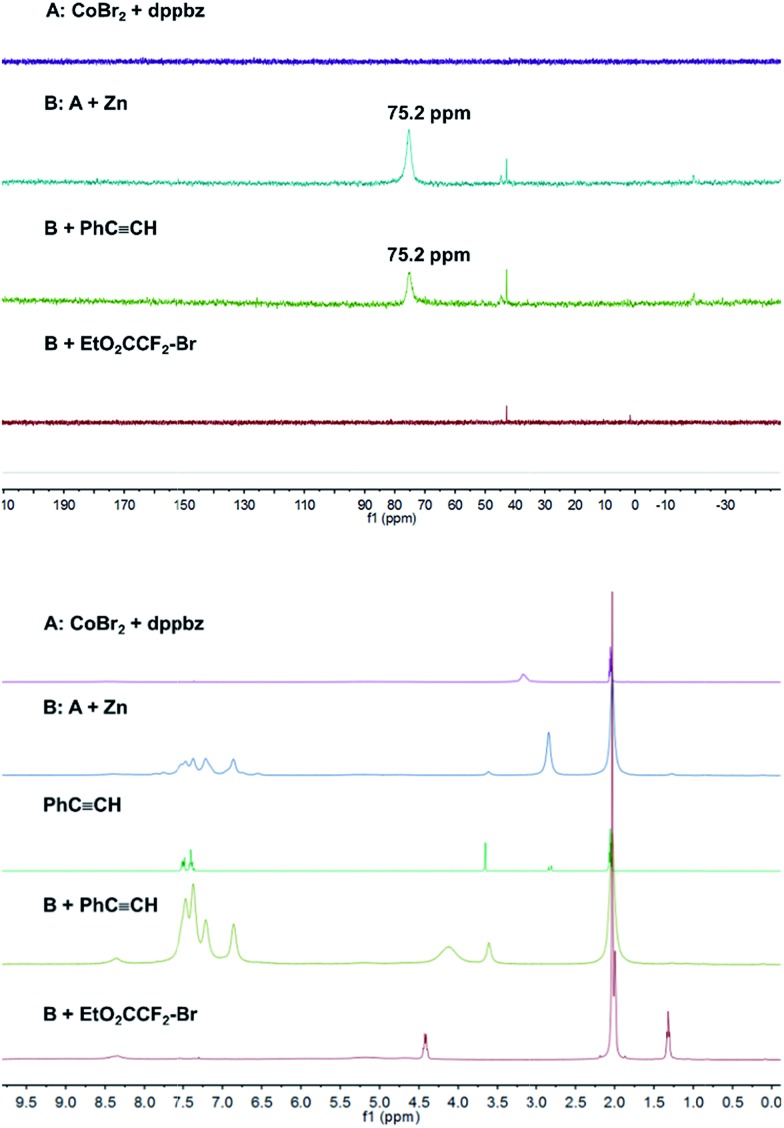
^31^P NMR (top) and ^1^H NMR (bottom) monitoring of catalyst formation and reductive electrophile activation.

**Fig. 2:**
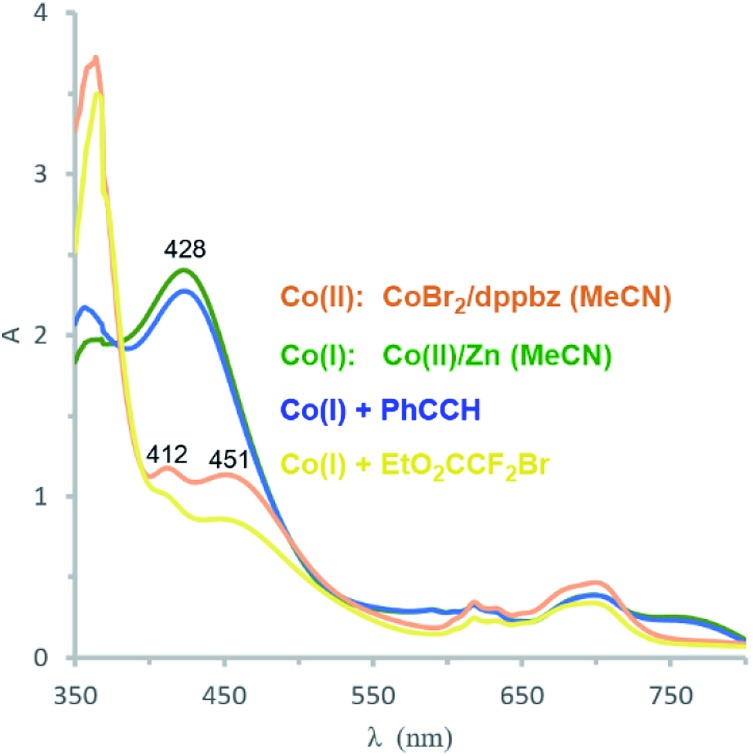
UV-vis absorption spectra: CoBr_2_/dppbz (orange); after reduction of Co(ii) with Zn to Co(i) (green); Co(i) with 1 equiv. **1a** (blue); Co(i) with 1 equiv. of **2a** (yellow).

Catalyst formation and substrate additions were monitored by ^31^P NMR and ^1^H NMR spectroscopy (Fig. 1). The reduction of the (NMR silent) CoBr_2_/dppbz mixture with Zn resulted in a Co(i) species with a ^31^P resonance at 75.2 ppm. The ^1^H NMR spectrum of this low-spin Co(i) complex gave signals 7–8 ppm. No changes were observed in ^31^P and ^1^H NMR spectra when phenyl acetylene (**1a**) was added to Co(i) which suggests the absence of significant alkyne-catalyst coordination. On the other hand, complete disappearance of the ^31^P (75.2 ppm) and ^1^H (7–8 ppm) signals was observed upon addition of EtO_2_CCF_2_Br (**2a**). This is a direct consequence of the reductive activation of **2a** which leads to a paramagnetic Co(ii,iii) species and the carbon-centered radical.^12^ These results are consistent with the UV-vis spectra (Fig. 2). Reduction of Co(ii) with Zn (and removal of residual Zn) resulted in an intense absorption of the Co(i) complex at 428 nm (green curve). Addition of **1a** to this solution gave no change of the absorption in this region (blue curve), whereas the addition of **2a** to Co(i) led to immediate colour change and the appearance of two weak bands at 412 and 451 nm (yellow curve).

**Scheme 8:**
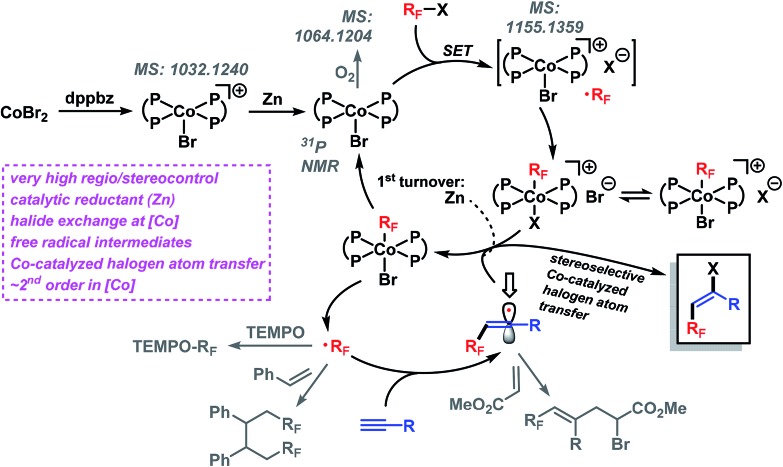
Proposed reaction mechanism.

The standard reaction between **1a** and **2a** went to completion within 90 min (with 2 mol% catalyst) and 12 min (4 mol% catalyst), respectively. Analysis of the initial rates (0.5–8 min, 1–4 mol% catalyst) displayed a near-2^nd^ order behavior of the catalyst concentration. We postulate the following reaction mechanism (Scheme 8). Complexation of dppbz with CoBr_2_ leads to the formation of [Co^II^(dppbz)_2_Br]^+^ as observed by the soft and inert mass spectrometric technique for sensitive organometallics LIFDI-MS (liquid injection field desorption ionization mass spectrometry). Reduction of the Co(ii) complex with equimolar Zn generates the catalytically active [Co^I^(dppbz)_2_Br].12*d* Exposure to oxygen/air gives [Co^III^(dppbz)_2_Br(O_2_)] (LIFDI-MS). [Co^I^(dppbz)_2_Br] effects the reductive single-electron activation of R_F_Br to give a [R_F_Co^III^(dppbz)_2_Br]^+^ species (LIFDI-MS)^12^ which is presumably a direct result of rapid combination of the intermediate Co(ii) complex and free radical R_F_˙.^14^ We have demonstrated that in the absence of residual zinc [R_F_Co^III^(dppbz)_2_Br]^+^ is not a catalyst (*vide supra*). Therefore, we propose – in contrast to the light-induced Co–R homolysis11g,^13^ – the reduction of [R_F_Co^III^(dppbz)_2_Br]^+^ by Zn in the first catalytic turnover to form the unstable complex [R_F_Co^II^(dppbz)_2_Br]. Dissociation of the fluoroalkyl radical R_F_˙ regenerates [Co^I^(dppbz)_2_Br]12h–j which can undergo another reductive activation of R_F_X to give [R_F_Co^III^(dppbz)_2_Br]^+^. The catalytic amounts of Zn present in the reaction dictate that another mechanism operates from the 2^nd^ turnover on, most likely an ATRA reaction involving halogen atom transfer from the cobalt complex [R_F_Co^III^(dppbz)_2_Br]^+^. Accordingly, the addition of R_F_˙ to the alkyne results in the formation of a vinyl radical intermediate which undergoes rapid halogen atom abstraction from [Co^III^(dppbz)_2_Br]^+^ to form the catalytically active Co(i) complex and R_F_˙.^15^ The high *E*-selectivity of the radical addition is a direct consequence of the steric hindrance by the R_F_ group in the vinyl radical.^16^ The higher *E*/*Z* stereoselectivity of the bromoalkylation over the iodoalkylation reactions can be explained by the shorter Co–Br bond (*vs.* Co–I) in the key catalytic Co(iii) species which effects an enhanced facial differentiation of the vinyl radical. The facile operation of this halogen atom transfer step with the intermediate vinyl radical is the key to the realization of an overall process that is catalytic in both metals, Co and Zn.

## 3 Conclusions

We have developed a convenient cobalt-catalyzed halofluoro-alkylation that exhibits wide substrate scope including terminal and internal alkynes, alkenes, and various fluoroalkyl and alkyl bromides and iodides. The protocol enables the highly regio-selective and stereoselective synthesis of densely functionalized halogenated (*E*)-alkenes under very mild reaction conditions (2 mol% catalyst, 5 mol% Zn, acetone/water, 20 °C, 3 h). Contrary to literature reports, mechanistic studies documented for the first time that the halogen atom transfer is a cobalt-mediated process. The R_F_Co^III^X complex is the key catalytic intermediate which generates the free R_F_˙ radical and mediates the halogen atom transfer to the terminal vinyl radical. This mechanistic deviation from substrate control to catalyst control may provide the basis for the development of related halogen atom transfer reactions through catalyst design. Further, this ATRA reaction operates without a stoichiometric reductant for the regeneration of the low-valent Co(i) catalyst. The high functional group tolerance and mild reaction conditions make this protocol highly attractive in the context of complex molecule synthesis with potential utility for medicinal chemistry endeavours.

## Conflicts of interest

There are no conflicts to declare.

## Supplementary Material

Supplementary information
