# Supplementary material for: Stereoselective cobalt-catalyzed halofluoroalkylation of alkynes
Source: Chem Sci. 2018 Jan 5;9(7):1795–802. doi: 10.1039/c7sc04916a (PMC5892352; doi:10.1039/c7sc04916a)
Supplement: Supplementary file 1 [file SC-009-C7SC04916A-s001.pdf]

Electronic Supplementary Material (ESI) for Chemical Science.
This journal is © The Royal Society of Chemistry 2018

Stereoselective Cobalt-Catalyzed Halofluoroalkylation of Alkynes

Guojiao Wu and Axel Jacobi von Wangelin*

Table of contents

1. General experimental methods S2
2. Method optimization S2
3. General procedure and characterization data S4
4. Mechanistic studies S15
5. Kinetic experiments S17
6. References S20
7. ^{1}H, ^{13}C, and ^{19}F spectra S21

1. General experimental methods.

Reagents were purchased from commercial suppliers and used without further purification. All solvents were used without distillation. Column chromatograph was performed on 35-70 mesh silica gel (Acros Organics). ^{1}H, ^{13}C, ^{19}F spectra were recorded on a Bruker Avance 300 or Avance 600 Kryo spectrometer using $CDCl_3$ as solvent. Chemical shifts are reported in ppm and referenced to residual solvent signal (CDCl3: ^{1}H NMR: δ 7.26 ppm, ^{13}C NMR: δ 77.0 ppm). High Resolution Mass Spectrometry (HRMS) were recorded on Finnigan MAT 900s. GC-yields was obtained using dodecane as internal standard with Gas chromatography with FID (GC-FID, HP6890 GC-System with injector 7683B and Agilent 7820A System, carried gas: H_2).

2. Method optimizations[a]

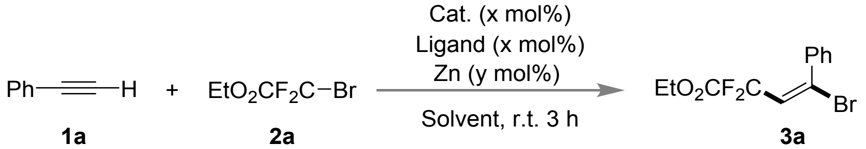

1a 2a 3a

entry	**Cat. (x mol%)**	**Ligand**	**solvent**	**3a** (%)[b]
1	$CoBr_2$ (5)	dppe	dioxane	ND
2	$CoBr_2$ (5)	dppf	dioxane	ND
3	$CoBr_2$ (5)	dppbz	dioxane	22
4	$CoBr_2$ (5)	dppb	dioxane	ND
5	$CoBr_2$ (5)	dppbz	Dio/H_2O = 30: 1	81
6	$CoBr_2$ (5)	dppbz	THF/H_2O = 30: 1	79
7	$CoBr_2$ (5)	dppbz	MeCN/H_2O = 30: 1	88
8	$CoBr_2$ (5)	dppbz	acetone/H_2O = 30: 1	88
9	$CoBr_2$ (5)	dppbz	DMF/H_2O = 30: 1	27
10	$CoBr_2$ (5)	dppbz	DMSO/H_2O = 30: 1	ND
11	$CoBr_2$ (5)	dppbz	PhMe/H_2O = 30: 1	ND
12	$CoBr_2$ (5)	dppbz	DCE/H_2O = 30: 1	81
13	$CoBr_2$ (5)	dppbz	EtOAc/H_2O = 30: 1	ND
14	$CoBr_2$ (5)	dppbz	Et_2O/H_2O = 30: 1	ND
**15**	$CoBr_2$ (5)	dppbz	EtOH/H_2O = 30: 1	20
**16**		dppbz (5)	acetone/H_2O = 30: 1	ND
**17**	$CoBr_2$ (5)		acetone/H_2O = 30: 1	ND
**18**	$CoBr_2$ (5)	dppbz	acetone/H_2O = 30: 1	ND[c]
**19**	$FeBr_2$ (5)	dppbz	acetone/H_2O = 30: 1	ND
**20**	$NiCl_2$ (5)	dppbz	acetone/H_2O = 30: 1	ND
**21**	$CrCl_3$ (5)	dppbz	acetone/H_2O = 30: 1	ND
**22**	$MnBr_2$ (5)	dppbz	acetone/H_2O = 30: 1	ND
**23**	$CuSO_4 \cdot 5H_2O$ (5)	dppbz	acetone/H_2O = 30: 1	ND
**24**	Cp_2TiCl_2 (5)	dppbz	acetone/H_2O = 30: 1	ND
**25**	$CoCl_2$ (5)	dppbz	acetone/H_2O = 30: 1	83
**26**	$CoCl_2 \cdot 4H_2O$ (5)	dppbz	acetone/H_2O = 30: 1	83

**27**	$CoBr_2$ (5)	PPh3	acetone/H_2O = 30: 1	ND[d]
**28**	$CoBr_2$ (5)	Xantphos	acetone/H_2O = 30: 1	ND
**29**	$CoBr_2$ (5)	DPEphos	acetone/H_2O = 30: 1	ND
**30**	$CoBr_2$ (5)	dppe	acetone/H_2O = 30: 1	27
**31**	$CoBr_2$ (5)	dppen	acetone/H_2O = 30: 1	31
**32**	$CoBr_2$ (5)	dppf	acetone/H_2O = 30: 1	ND
**33**	$CoBr_2$ (5)	dppb	acetone/H_2O = 30: 1	ND
**34**	$CoBr_2$ (5)	BINAP	acetone/H_2O = 30: 1	ND
**35**	$CoBr_2$ (5)	TMEDA	acetone/H_2O = 30: 1	ND
**36**	$CoBr_2$ (5)	bipy	acetone/H_2O = 30: 1	ND
**37**	$CoBr_2$ (5)	dtpy	acetone/H_2O = 30: 1	ND
**38**	$CoBr_2$ (5)	Phen	acetone/H_2O = 30: 1	ND
**49**	$CoBr_2$ (5)	L1	acetone/H_2O = 30: 1	ND
**40**	$CoBr_2$ (5)	L2	acetone/H_2O = 30: 1	ND
**41**	$CoBr_2$ (5)	L3	acetone/H_2O = 30: 1	ND
**43**	$NiCl_2$ (5)	dtpy	acetone/H_2O = 30: 1	ND
**44**	$NiCl_2$ (5)	TMEDA	acetone/H_2O = 30: 1	ND
**45**	$CoBr_2$ (2)	dppbz	acetone/H_2O = 30: 1	88
**46**	$CoBr_2$ (2)	dppbz	acetone/H_2O = 30: 1	27[e]
**47**	$CoBr_2$ (2)	dppbz	acetone/H_2O = 30: 1	87[f]
**48**	$CoBr_2$ (2)	dppbz	MeCN/H_2O = 30: 1	65
**49**	$CoBr_2$ (1)	dppbz	acetone/H_2O = 30: 1	63[g]
**50**	$CoBr_2$ (2)	dppbz	acetone	69[f]

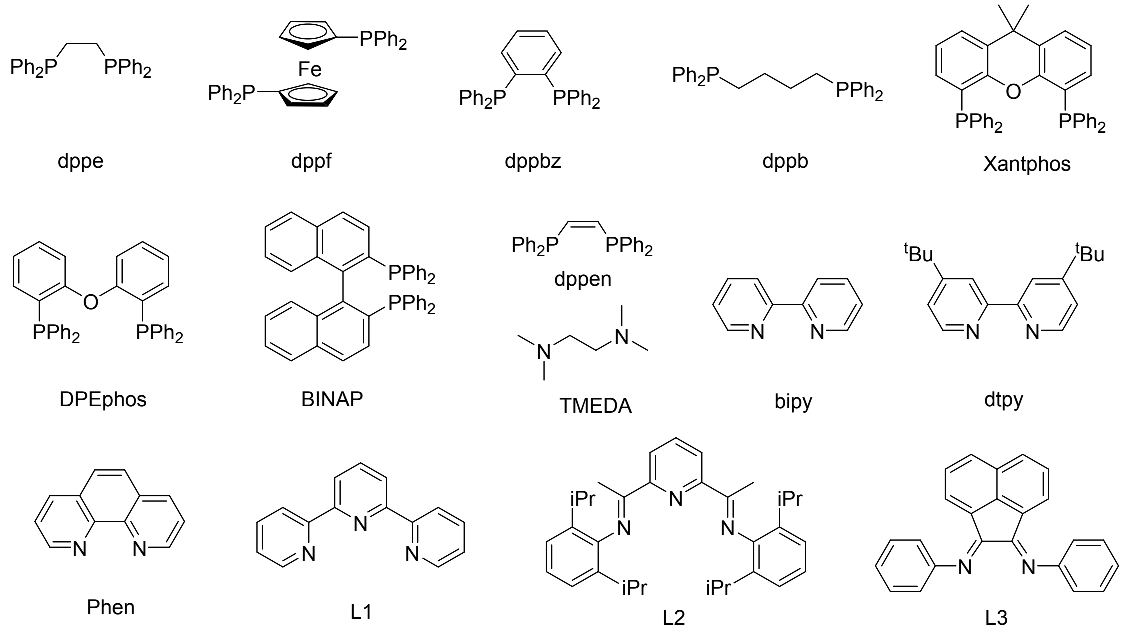

[a] Reaction conditions: **1a** (0.3 mmol), **2a** (0.45 mmol), catalyst (x mol%), ligand (x mol%) and Zn (20 mol%) in 0.6 mL acetone/H_2O for 3 hours. [b] GC yield using dodecane as internal standard. [c] Without Zn. [d] ligand (10 mol%). [e] Mn (20 mol%) instead of Zn. [f] Zn (5 mol%). [g] Zn (10 mol%)

3. General Procedure and characterization data

$CoBr_2$ (1.3 mg, 0.006 mmol, 0.02 equiv), dppbz (2.7 mg, 0.006 mmol, 0.02 equiv), Zn (1.0 mg, 0.015 mmol, 0.05 equiv) were dissolved in acetone/H_2O (30:1, 0.6 mL) and stirred for 2 minutes. Alkyne or alkene was added into the system (Note: if it is solid, weigh it with the catalyst and dissolve them together), followed with R_f-X (0.45 mmol, 1.5 equiv). The mixture was stirred at room temperature for 3 hours. The solvent was removed to leave a crude product, which was purified by column chromatography on silica gel to afford the product **3**.

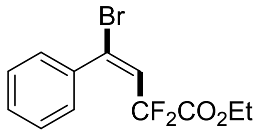

3a

The product **3a**[1] was purified with silica gel chromatography (Pe/EA = 20 : 1) as a colorless oil. ^{1}H NMR (300 MHz, $CDCl_3$) δ 7.37 (s, 5H), 6.50 (t, *J* = 11.1 Hz, 1H), 3.98 (q, *J* = 7.2 Hz, 2H), 1.19 (t, *J* = 7.2 Hz, 3H); ^{13}C NMR (75 MHz, $CDCl_3$) δ 162.5 (t, *J* = 33.4 Hz), 137.1, 133.5 (t, *J* = 10.2 Hz), 130.0, 128.5 (t, *J* = 2.1 Hz), 128.1, 125.0 (t, *J* = 28.7 Hz), 111.0 (t, *J* = 248.8 Hz), 63.1, 13.7; ^{19}F NMR (282 MHz, $CDCl_3$) δ -93.6 (*E*), -97.6 (*Z*)

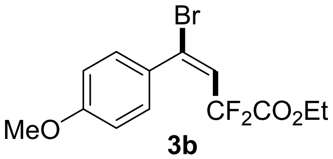

3b

The product **3b**[1] was purified with silica gel chromatography (Pe/EA = 20 : 1) as a colorless oil. ^{1}H NMR (300 MHz, $CDCl_3$) δ 7.35-7.30 (m, 2H), 6.88-6.83 (m, 2H), 6.44 (t, *J* = 11.0 Hz, 1H), 3.99 (q, *J* = 7.2 Hz, 2H), 1.19 (t, *J* = 7.2 Hz, 3H); ^{13}C NMR (75 MHz, $CDCl_3$) δ 162.6 (t, *J* = 33.4 Hz), 160.8, 134.0 (t, *J* = 10.4 Hz), 130.4 (t, *J* = 2.0 Hz), 129.3, 124.3 (t, *J* = 28.7 Hz), 113.4, 111.2 (t, *J* = 248.3 Hz), 63.1, 55.3, 13.7; ^{19}F NMR (282 MHz, $CDCl_3$) δ -93.6 (*E*).

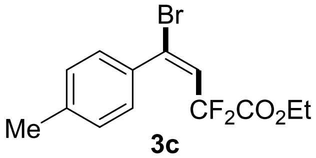

3c

The product **3c** was purified with silica gel chromatography (Pe/EA = 20 : 1) as a colorless oil. ^{1}H NMR (300 MHz, $CDCl_3$) δ 7.26 (d, *J* = 8.1 Hz, 2H), 7.26 (d, *J* = 8.4 Hz, 2H), 6.46 (t, *J* = 11.1 Hz, 1H), 3.99 (q, *J* = 7.2 Hz, 2H), 2.36 (s, 3H), 1.19 (t, *J* = 7.2 Hz, 3H); ^{13}C NMR (75 MHz, $CDCl_3$) δ 162.5 (t, *J* = 33.5 Hz), 140.3, 134.3, 133.9 (t, *J* = 10.2 Hz), 128.7, 128.5 (t, *J* = 2.0 Hz), 124.7 (t, *J* = 28.7 Hz), 111.2 (t, *J* = 248.6 Hz), 63.1, 21.3, 13.6; ^{19}F NMR (282 MHz, $CDCl_3$) δ -94.0 (*E*), -97.8 (*Z*); HRMS (EI) (*m/z*): $[M]^+$ calcd for $C_{13}H_{13}BrF_2O_2$: 318.0062, found: 318.0053.

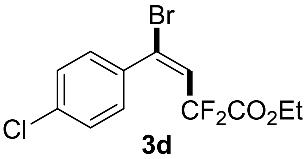

3d

The product **3d** was purified with silica gel chromatography (Pe/EA = 20 : 1) as a colorless oil. ^{1}H NMR (300 MHz, $CDCl_3$) δ 7.40-7.30 (m, 4H), 6.54 (td, *J* = 11.4, 0.8 Hz, 1H), 4.12 (q, *J* = 7.1 Hz, 2H), 1.27 (td, *J* = 7.1, 0.8 Hz, 3H); ^{13}C NMR (75 MHz, $CDCl_3$) δ 162.4 (t, *J* = 33.4 Hz), 136.0, 135.6, 132.1 (t, *J* = 9.4 Hz), 129.8 (t, *J* = 2.0 Hz), 128.4, 125.4 (t, *J* = 28.0 Hz), 110.9 (t, *J* = 249.9 Hz), 63.3, 13.7; ^{19}F

NMR (282 MHz, $CDCl_3$) δ -94.5 (*E*); HRMS (EI) (*m/z*): $[M]^+$ calcd for $C_{12}H_{10}BrClF_2O_2$: 337.9515, found: 337.9504.

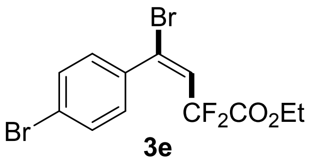
3e

The product **3e**[1] was purified with silica gel chromatography (Pe/EA = 20 : 1) as a colorless oil. ^{1}H NMR (300 MHz, $CDCl_3$) δ 7.45-7.42 (m, 2H), 7.19-7.16 (m, 2H), 6.43 (t, *J* = 11.5 Hz, 1H), 4.02 (q, *J* = 7.2 Hz, 2H), 1.17 (t, *J* = 7.2 Hz, 3H); ^{13}C NMR (75 MHz, $CDCl_3$) δ 162.4 (t, *J* = 33.4 Hz), 136.1, 132.1 (t, *J* = 9.4 Hz), 131.3, 130.3 (t, *J* = 2.1 Hz), 125.4 (t, *J* = 27.9 Hz), 124.3, 110.9 (t, *J* = 250.1 Hz), 63.3, 13.7; ^{19}F NMR (282 MHz, $CDCl_3$) δ -95.1 (*E*); HRMS (EI) (*m/z*): $[M]^+$ calcd for $C_{12}H_{10}BrClF_2O_2$: 337.9515, found: 337.9504.

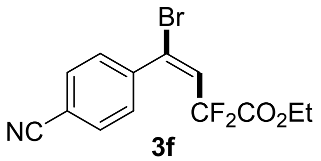
3f

The product **3f** was purified with silica gel chromatography (Pe/EA = 10 : 1) as a colorless oil. ^{1}H NMR (300 MHz, $CDCl_3$) δ 7.67 (d, *J* = 8.6 Hz, 2H), 7.48 (d, *J* = 8.4 Hz, 2H), 6.55 (t, *J* = 12.0 Hz, 1H), 4.17 (q, *J* = 7.1 Hz, 2H), 1.28 (t, *J* = 7.2 Hz, 3H); ^{13}C NMR (75 MHz, $CDCl_3$) δ 162.3 (t, *J* = 33.3 Hz), 141.6, 131.8, 130.6 (t, *J* = 8.3 Hz), 129.0 (t, *J* = 2.2 Hz), 126.0 (t, *J* = 27.0 Hz), 117.9, 113.5, 110.7 (t, *J* = 251.4 Hz), 63.5, 13.7; ^{19}F NMR (282 MHz, $CDCl_3$) δ -95.1 (*E*); HRMS (ESI) (*m/z*): $[M+H]^+$ calcd for $C_{13}H_{11}BrF_2NO_2$: 329.9936, found: 329.9940.

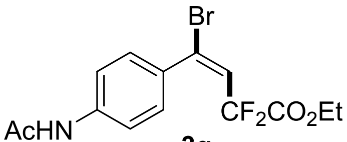
3g

The product **3g** was purified with silica gel chromatography (Pe/EA = 2 : 1) as a colorless oil. ^{1}H NMR (300 MHz, $CDCl_3$) δ 7.29-7.45 (m, 3H), 7.32 (d, *J* = 8.4 Hz, 2H), 6.46 (t, *J* = 11.2 Hz, 1H), 4.03 (q, *J* = 7.1 Hz, 2H), 1.21 (t, *J* = 7.2 Hz, 3H); ^{13}C NMR (75 MHz, $CDCl_3$) δ 169.0, 162.6 (t, *J* = 33.5 Hz), 139.6, 133.3 (t, *J* = 10.0 Hz), 132.5, 129.4, 124.6 (t, *J* = 28.4 Hz), 118.9, 111.0 (t, *J* = 248.8 Hz), 63.3, 24.5, 13.6; ^{19}F NMR (282 MHz, $CDCl_3$) δ -94.1 (*E*), -97.6 (*Z*); HRMS (ESI) (*m/z*): $[M+H]^+$ calcd for $C_{14}H_{15}BrF_2NO_3$: 362.0198, found: 362.0204.

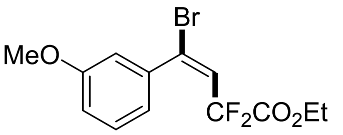
3h

The product **3h** was purified with silica gel chromatography (Pe/EA = 20 : 1) as a colorless oil. ^{1}H NMR (300 MHz, $CDCl_3$) δ 7.29-7.24 (m, 1H), 6.97-6.91 (m, 3H), 6.48 (t, *J* = 11.0 Hz, 1H), 3.99 (q, *J* = 7.2 Hz, 2H), 3.82 (s, 3H), 1.20 (t, *J* = 7.2 Hz, 3H); ^{13}C NMR (75 MHz, $CDCl_3$) δ 162.4 (t, *J* = 33.3 Hz), 158.9, 138.1, 133.3 (t, *J* = 10.4 Hz), 129.2, 125.0 (t, *J* = 28.9 Hz), 120.8 (t, *J* = 1.9 Hz), 116.0, 113.7 (t, *J* = 2.0 Hz), 111.0 (t, *J* = 248.7 Hz), 63.1, 55.3, 13.6; ^{19}F NMR (282 MHz, $CDCl_3$) δ -

94.0 (*E*); HRMS (EI) (*m/z*): $[M]^+$ calcd for $C_{14}H_{13}BrF_2O_3$: 334.0011, found: 334.0003.

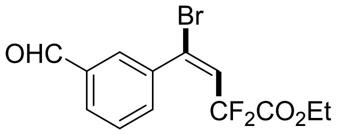

3i

The product **3i** was purified with silica gel chromatography (Pe/EA = 10 : 1) as a colorless oil. ^{1}H NMR (300 MHz, $CDCl_3$) δ 10.02 (s, 1H), 7.91-7.88 (m, 2H), 7.64-7.53 (m, 2H), 6.56 (t, *J* = 11.8 Hz, 1H), 4.12 (q, *J* = 7.1 Hz, 2H), 1.25 (t, *J* = 7.1 Hz, 3H); ^{13}C NMR (75 MHz, $CDCl_3$) δ 191.2, 162.4 (t, *J* = 33.3 Hz), 138.3, 136.1, 134.0, 131.5 (t, *J* = 8.7 Hz), 130.6, 129.5, 129.0, 125.8 (t, *J* = 27.4 Hz), 111.8 (t, *J* = 250.7 Hz), 63.4, 13.7; ^{19}F NMR (282 MHz, $CDCl_3$) δ -95.9 (*E*); HRMS (ESI) (*m/z*): $[M]^+$ calcd for $C_{13}H_{12}BrF_2O_3$: 332.9932, found: 332.9936.

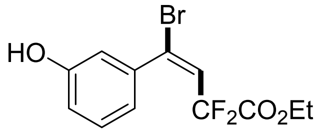

3j

The product **3j** was purified with silica gel chromatography (Pe/EA = 10 : 1) as a colorless oil. ^{1}H NMR (300 MHz, $CDCl_3$) δ 7.26-7.20 (m, 1H), 6.95-6.92 (m, 1H), 6.87-6.82 (m, 2H), 6.47 (t, *J* = 10.9 Hz, 1H), 5.70 (s, 1H), 4.00 (q, *J* = 7.2 Hz, 2H), 1.20 (t, *J* = 7.2 Hz, 3H); ^{13}C NMR (75 MHz, $CDCl_3$) δ 162.8 (t, *J* = 33.4 Hz), 155.2, 138.2, 131.1 (t, *J* = 10.7 Hz), 129.6, 125.0 (t, *J* = 29.2 Hz), 120.9, 117.3, 115.4, 111.0 (t, *J* = 248.3 Hz), 63.5, 13.6; ^{19}F NMR (282 MHz, $CDCl_3$) δ -93.6 (*E*); HRMS (EI) (*m/z*): $[M]^+$ calcd for $C_{12}H_{11}BrF_2O_3$: 319.9854, found: 319.9850.

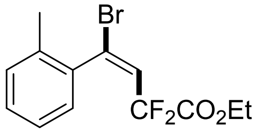

3k

The product **3k** was purified with silica gel chromatography (Pe/EA = 20 : 1) as a colorless oil. ^{1}H NMR (300 MHz, $CDCl_3$) δ 7.30-7.25 (m, 1H), 7.21-7.13 (m, 3H), 6.54 (t, *J* = 11.4 Hz, 1H), 3.98 (q, *J* = 7.2 Hz, 2H), 2.34 (s, 3H), 1.22 (t, *J* = 7.2 Hz, 3H); ^{13}C NMR (75 MHz, $CDCl_3$) δ 162.4 (t, *J* = 33.2 Hz), 136.3, 135.8, 132.9 (t, *J* = 10.1 Hz), 130.2, 129.9, 128.3 (t, *J* = 1.9 Hz), 126.2 (t, *J* = 28.4 Hz), 125.1, 110.9 (t, *J* = 248.6 Hz), 63.1, 19.2, 13.6; ^{19}F NMR (282 MHz, $CDCl_3$) δ -95.9 (d, *J* = 274.2 Hz, 1F, *E*), -98.1 (d, *J* = 274.2 Hz, 1F, *E*); HRMS (EI) (*m/z*): $[M]^+$ calcd for $C_{13}H_{13}BrF_2O_2$: 318.0062, found: 318.0057.

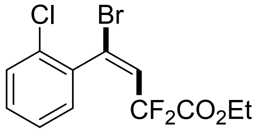

3l

The product **3l** was purified with silica gel chromatography (Pe/EA = 20 : 1) as a colorless oil. ^{1}H NMR (300 MHz, $CDCl_3$) δ 7.42-7.39 (m, 1H), 7.35-7.27 (m, 3H), 6.58 (t, *J* = 11.5 Hz, 1H), 4.15 (qd, *J* = 7.2, 2.7 Hz, 2H), 1.29 (t, *J* = 7.2 Hz, 3H); ^{13}C NMR (75 MHz, $CDCl_3$) δ 162.2 (t, *J* = 33.5 Hz), 135.8, 132.1 (t, *J* = 1.7 Hz), 130.9,129.9 (t, *J* = 2.0 Hz), 129.7, 129.3 (t, *J* = 9.1 Hz), 127.1 (t, *J* = 28.3 Hz), 126.6, 110.8 (t, *J* = 250.3 Hz), 63.3, 13.7; ^{19}F NMR (282 MHz, $CDCl_3$) δ -97.9 (d, *J* = 18.6 Hz, *E*), -98.9 (*Z*); HRMS (EI) (*m/z*): $[M]^+$ calcd for $C_{12}H_{10}BrClF_2O_2$: 337.9515, found: 337.9510.

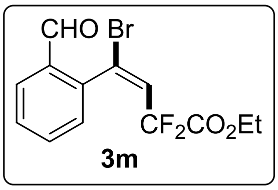

The product **3m** was purified with silica gel chromatography (Pe/EA = 10 : 1) as a colorless oil. ^{1}H NMR (300 MHz, $CDCl_3$) δ 10.21 (s, 1H), 7.95-7.93 (m, 1H), 7.62-7.54 (m, 2H), 7.35-7.32 (m, 1H), 6.67 (t, *J* = 11.5 Hz, 1H), 4.12 (qd, *J* = 7.2, 3.1 Hz, 2H), 1.26 (t, *J* = 7.2 Hz, 3H); ^{13}C NMR (75 MHz, $CDCl_3$) δ 190.0, 162.3 (t, *J* = 33.6 Hz), 139.2, 133.7, 132.0, 130.2, 129.4 (t, *J* = 2.0 Hz), 129.2, 129.0 (t, *J* = 8.8 Hz), 127.4 (t, *J* = 27.6 Hz), 110.8 (t, *J* = 251.6 Hz), 63.5, 13.7; ^{19}F NMR (282 MHz, $CDCl_3$) δ -97.3 (d, *J* = 26.2 Hz, *E*), -98.7 (*Z*); HRMS (EI) (*m/z*): $[M]^+$ calcd for $C_{13}H_{11}BrF_2O_3$: 331.9854, found: 331.9853.

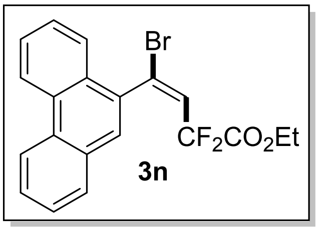

The product **3n** was purified with silica gel chromatography (Pe/EA = 20 : 1) as a colorless oil. ^{1}H NMR (300 MHz, $CDCl_3$) δ 8.73-8.65 (m, 2H), 8.06-8.03 (m, 1H), 7.92-7.89 (m, 1H), 7.74-7.60 (m, 5H), 7.72-7.68 (m, 1H), 7.35-7.32 (m, 1H), 6.82 (dd, *J* = 12.1, 8.2 Hz, 1H), 3.59 (qd, *J* = 10.8, 7.1 Hz, 1H), 3.39 (qd, *J* = 10.7, 7.1 Hz, 1H), 0.86 (t, *J* = 7.2 Hz, 3H); ^{13}C NMR (75 MHz, $CDCl_3$) δ 162.2 (dd, *J* = 34.3, 32.0 Hz), 132.4, 132.0 (dd, *J* = 11.5, 9.8 Hz), 130.8, 130.4, 130.3, 129.4, 128.5 (d, *J* = 32.2 Hz), 128.4 (t, *J* = 1.7 Hz), 128.1 (d, *J* = 32.3 Hz), 128.1, 127.2, 127.2, 126.9, 126.3, 122.9, 122.6, 111.1 (dd, *J* = 251.1, 247.2 Hz), 62.9, 13.2; ^{19}F NMR (282 MHz, $CDCl_3$) δ -94.1 (d, *J* = 276 Hz, 1F, *E*), -97.1 (d, *J* = 276 Hz, 1F, *E*); HRMS (EI) (*m/z*): $[M]^+$ calcd for $C_{20}H_{15}BrF_2O_2$: 404.0218, found: 404.0215.

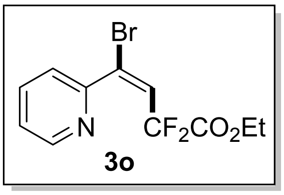

The product **3o**[1] was purified with silica gel chromatography (Pe/EA = 5 : 1) as a pale yellow oil. ^{1}H NMR (300 MHz, $CDCl_3$) δ 8.47-8.45 (m, 1H), 7.77-7.70 (m, 2H), 7.28-7.23 (m, 1H), 6.63 (t, *J* = 12.2 Hz, 1H), 4.20 (q, *J* = 7.1 Hz, 2H), 1.23 (t, *J* = 7.1 Hz, 3H); ^{13}C NMR (75 MHz, $CDCl_3$) δ 162.5 (t, *J* = 33.8 Hz), 152.4, 147.4, 137.1, 129.8 (d, *J* = 11.0 Hz), 128.4 (t, *J* = 31.3 Hz), 124.2, 124.0, 111.5 (t, *J* = 245.8 Hz), 62.5, 13.8; ^{19}F NMR (282 MHz, $CDCl_3$) δ -94.9 (*E*), -98.5 (*Z*).

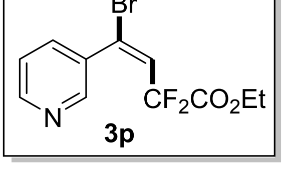

The product **3p** was purified with silica gel chromatography (Pe/EA = 5 : 1) as a pale yellow oil. ^{1}H NMR (300 MHz, $CDCl_3$) δ 8.62-8.59 (m, 2H), 7.70-7.67 (m, 1H), 7.33-7.31 (m, 1H), 6.59 (t, *J* = 11.8, 1H), 4.15 (q, *J* = 7.2 Hz, 2H), 1.27 (t, *J* = 7.1 Hz, 3H); ^{13}C NMR (75 MHz, $CDCl_3$) δ 162.4 (t, *J* = 33.3 Hz), 150.5, 148.6 (t, *J* = 2.4 Hz), 135.8 (t, *J* = 2.1 Hz), 133.6, 129.6 (t, *J* = 8.6 Hz), 126.5 (t, *J* = 27.2 Hz), 122.8, 110.8 (t, *J* = 251.1 Hz), 63.5, 13.7; ^{19}F NMR (282 MHz, $CDCl_3$) δ -95.6 (*E*); HRMS (EI) (*m/z*): $[M]^+$ calcd for $C_{11}H_{10}BrF_2NO_2$: 304.9858, found: 304.9847.

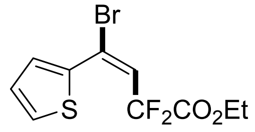

3q

The product **3q** was purified with silica gel chromatography (Pe/EA = 20 : 1) as a colorless oil. ^{1}H NMR (300 MHz, $CDCl_3$) δ 7.49 (dd, J = 5.1, 1.2 Hz, 1H), 7.30-7.29 (m, 1H), 6.98 (dd, J = 5.1, 3.7 Hz, 1H), 6.46 (t, *J* = 11.1, 1H), 4.07 (q, *J* = 7.2 Hz, 2H), 1.19 (t, *J* = 7.1 Hz, 3H); ^{13}C NMR (75 MHz, $CDCl_3$) δ 162.3 (t, *J* = 33.5 Hz), 138.8, 130.7 (t, *J* = 3.1 Hz), 129.9, 127.0, 125.3, 125.1 (t, *J* = 29.6 Hz), 111.1 (t, *J* = 247.9 Hz), 63.3, 13.7; ^{19}F NMR (282 MHz, $CDCl_3$) δ -92.7 (*E*), -97.4 (*E*); HRMS (EI) (*m/z*): $[M]^+$ calcd for $C_{10}H_9BrF_2O_2S$: 309.9469, found: 309.9473.

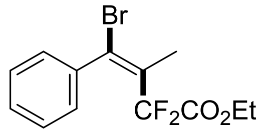

3r

The product **3r** was purified with silica gel chromatography (Pe/EA = 20 : 1) as a colorless oil. ^{1}H NMR (300 MHz, $CDCl_3$) δ 7.39-7.29 (m, 1H), 3.95 (q, *J* = 7.2 Hz, 2H), 2.24 (s, 3H), 1.24 (t, *J* = 7.2 Hz, 3H); ^{13}C NMR (75 MHz, $CDCl_3$) δ 162.8 (t, *J* = 33.7 Hz), 138.9, 130.4 (t, *J* = 24.4 Hz), 129.9 (t, *J* = 7.8 Hz), 129.2, 129.0 (t, *J* = 1.9 Hz), 127.9, 112.3 (t, *J* = 252.3 Hz), 62.9, 19.3 (t, *J* = 4.0 Hz), 13.6; ^{19}F NMR (282 MHz, $CDCl_3$) δ -94.8 (*E*); HRMS (ESI) (*m/z*): $[M+H]^+$ calcd for $C_{13}H_{14}BrF_2O_2$: 319.0140, found: 319.0145.

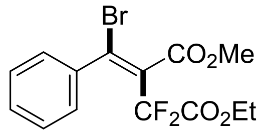

3s

The product **3s** was purified with silica gel chromatography (Pe/EA = 10 : 1) as a colorless oil. ^{1}H NMR (300 MHz, $CDCl_3$) δ 7.41 (s, 5H), 4.46 (q, *J* = 7.1 Hz, 0.33H, *Z*), 4.04-3.95 (m, 4.33H, *E*), 3.55 (s, 0.47H, *Z*), 1.44 (t, *J* = 7.1 Hz, 0.52H, *Z*), 1.22 (t, *J* = 7.2 Hz, 2.52H, *E*); ^{13}C NMR (75 MHz, $CDCl_3$) δ 163.8 (t, *J* = 2.6 Hz), 161.4 (t, *J* = 32.9 Hz), 136.7, 133.7 (t, *J* = 7.5 Hz), 130.2 (*Z*), 130.2, 130.1 (t, *J* = 27.5 Hz), 128.4 (*Z*), 128.2 (t, *J* = 2.0 Hz), 128.1, 110.5 (t, *J* = 253.9 Hz), 63.7 (*Z*), 63.4, 53.1, 52.8 (*Z*), 13.8 (*Z*), 13.6; ^{19}F NMR (282 MHz, $CDCl_3$) δ -93.2 (*E*), -99.1 (*Z*); HRMS (ESI) (*m/z*): $[M+H]^+$ calcd for $C_{14}H_{14}BrF_2O_4$: 363.0038, found: 363.0045.

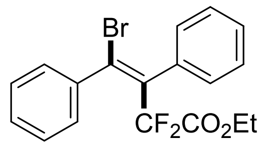

3t

The product **3t**[1] was purified with silica gel chromatography (Pe/EA = 10 : 1) as a colorless oil. ^{1}H NMR (300 MHz, $CDCl_3$) δ 7.47-7.39 (m, 10H), 3.90 (q, *J* = 7.2 Hz, 2H), 1.16 (t, *J* = 7.2 Hz, 3H); ^{13}C NMR (75 MHz, $CDCl_3$) δ 162.7 (t, *J* = 33.6 Hz), 138.5, 136.6 (t, *J* = 1.6 Hz), 135.7 (t, *J* = 24.8 Hz), 132.7 (t, *J* = 6.3 Hz), 129.5, 128.9 (t, *J* = 1.9 Hz), 128.6, 128.4, 128.1, 111.6 (t, *J* = 253.4 Hz), 63.0, 13.6; ^{19}F NMR (282 MHz, $CDCl_3$) δ -91.7 (*E*), -96.6 (*Z*).

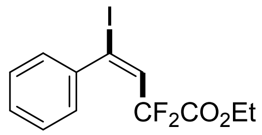

3u

The product **3u**[2] was purified with silica gel chromatography (Pe/EA = 10 : 1) as a colorless oil. ^{1}H NMR (300 MHz, $CDCl_3$) δ 7.36-7.28 (m, 5H), 6.72 (t, *J* = 10.9 Hz, 2H), 3.97 (t, *J* = 7.2 Hz, 2H), 1.20 (t, *J* = 7.2 Hz, 3H); ^{19}F NMR (282 MHz, $CDCl_3$) δ -94.3 (*E*), -98.6 (*Z*).

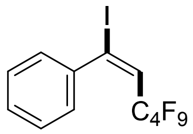

3v

The product **3v**[3] was purified with silica gel chromatography (Pe) as a colorless oil. ^{1}H NMR (300 MHz, $CDCl_3$) δ 7.38-7.27 (m, 5H), 6.59 (t, *J* = 13.5 Hz, 1H); ^{13}C NMR (75 MHz, $CDCl_3$) δ 141.3, 129.3, 128.0, 126.8 (t, *J* = 21.8 Hz), 126.8, 112.8 (t, *J* = 6.2 Hz); ^{19}F NMR (282 MHz, $CDCl_3$) δ -81.5 (t, *J* = 9.4 Hz, 2F), -105.9 (t, *J* = 11.3 Hz, 1.83F, *E*), -109.7 (t, *J* = 11.8 Hz, 0.16F, *Z*), -124.2 to -124.33 (m, 2F), -126.2 to -126.4 (m, 2F).

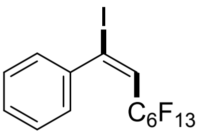

3w

The product **3w**[4] was purified with silica gel chromatography (Pe) as a colorless oil. ^{1}H NMR (300 MHz, $CDCl_3$) δ 7.38-7.27 (m, 5H), 6.59 (t, *J* = 13.5 Hz, 1H); ^{19}F NMR (282 MHz, $CDCl_3$) δ -81.3 (brs, 3F), -105.7 (t, *J* = 13.2 Hz, 1.89F, *E*), -109.4 (t, *J* = 12.5 Hz, 0.11F, *Z*), -122.2 to -122.3 (m, 2F), -123.4 (brs, 4F), -126.6 to -126.7 (m, 2F).

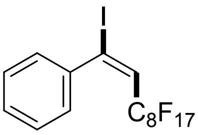

3x

The product **3x**[5] was purified with silica gel chromatography (Pe) as a colorless oil. ^{1}H NMR (300 MHz, $CDCl_3$) δ 7.37-7.27 (m, 5H), 6.59 (t, *J* = 13.5 Hz, 1H); ^{13}C NMR (75 MHz, $CDCl_3$) δ 141.3, 129.3, 128.0, 127.0 (t, *J* = 22.1 Hz), 126.8 (t, *J* = 2.2 Hz), 112.7 (t, *J* = 6.1 Hz); ^{19}F NMR (282 MHz, $CDCl_3$) δ -81.2 to -81.3 (m, 3F), -105.7 (t, *J* = 12.9 Hz, 1.94F, *E*), -109.4 (t, *J* = 12.7 Hz, 0.10F, *Z*), 122.0 (brs, 2H), -122.4 (brs, 4F), -123.3 (brs, 4F), -126.6 (s, 2F).

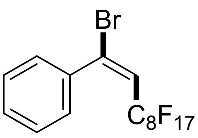

3y

The product **3y** was purified with silica gel chromatography (Pe) as a colorless oil. ^{1}H NMR (300 MHz, $CDCl_3$) δ 7.40-7.34 (m, 5H), 6.39 (t, *J* = 13.5 Hz, 1H); ^{13}C NMR (75 MHz, $CDCl_3$) δ 137.6, 137.3 (t, *J* = 5.7 Hz), 129.8, 128.1, 127.8 (t, *J* = 2.3 Hz), 119.3 (t, *J* = 22.3 Hz); ^{19}F NMR (282 MHz, $CDCl_3$) δ -81.2 (t, *J* = 9.9 Hz, 3F), -105.7 (t, *J* = 13.1 Hz, 2F), 122.0 (brs, 2H), -122.4 (brs, 4F), -123.3 (brs, 4F), -126.5 to 126.6 (m, 2F); HRMS (EI) (*m/z*): $[M]^+$ calcd for $C_{16}H_{16}BrF_{17}$: 599.9376, found: 599.9354.

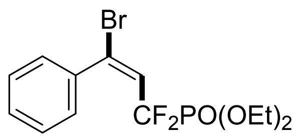

3z

The product **3z** was purified with silica gel chromatography (Pe) as a colorless oil. ^{1}H NMR (300 MHz, $CDCl_3$) δ 7.44-7.42 (m, 2H), 7.35-7.32 (m, 3H), 6.41 (td, *J* = 14.8, 1.8 Hz, 1H), 4.26-4.15 (m, 4H), 1.34 (t, *J* = 7.1 Hz, 6H); ^{13}C NMR (75 MHz, $CDCl_3$) δ 138.0

(d, J = 1.2 Hz), 133.3 (dt, J = 10.4, 7.5 Hz), 129.4, 128.3 (d, J = 1.3 Hz), 127.7, 123.1 (td, J = 21.0, 14.4 Hz), 119.3 (td, J = 262.9, 220.9 Hz), 64.8 (d, J = 6.8 Hz), 16.3 (d, J = 5.4 Hz); ^{19}F NMR (282 MHz, $CDCl_3$) δ -104.4 (d, J =109.9 Hz, *Z*), -104.8 (d, J =110.3 Hz, *E*); HRMS (ESI) (*m/z*): $[M]^+$ calcd for $C_{13}H_{11}BrF_2O_3P$: 369.0061, found: 369.0073.

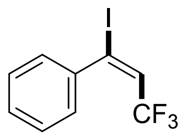

3aa

The product **3aa**[6] was purified with silica gel chromatography (Pe) as a colorless oil. ^{1}H NMR (300 MHz, $CDCl_3$) δ 7.51-7.48 (m, 0.34H, *Z*), 7.39-7.31 (m, 4.67H, *E*+*Z*), [6.64 (q, J = 7.3 Hz, *E*) and 6.58 (q, J = 7.4 Hz, *Z*), 1H], 4.26-4.15 (m, 4H), 1.34 (t, J = 7.1 Hz, 6H); ^{13}C NMR (75 MHz, $CDCl_3$) δ 141.9 (*Z*), 140.7, 129.5 (q, J = 33.6 Hz), 129.5, 128.5 (*Z*), 128.2 (*Z*),128.1, 127.2, 126.7 (q, J = 36.0 Hz, *Z*) 121.2 (q, J = 273.9 Hz), 111.1 (q, J = 6.3 Hz); ^{19}F NMR (282 MHz, $CDCl_3$) δ -57.7 (*E*), -60.1 (*E*).

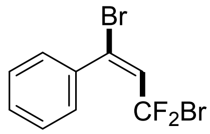

3ab

The product **3ab**[7] was purified with silica gel chromatography (Pe) as a colorless oil. ^{1}H NMR (300 MHz, $CDCl_3$) δ 7.45-7.377 (m, 5H), 6.65 (td, J = 11.4, 0.9 Hz, 1H); ^{13}C NMR (75 MHz, $CDCl_3$) δ 137.2, 131.5 (t, J = 5.6 Hz), 129.9, 128.9 (t, J = 25.4 Hz), 128.2, 128.0 (t, J = 1.9 Hz), 114.4 (q, J = 304.4 Hz); ^{19}F NMR (282 MHz, $CDCl_3$) δ -40.5.

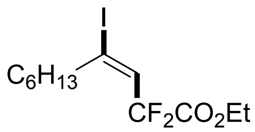

3ac

The product **3ac**[8] was purified with silica gel chromatography (Pe/EA = 20 : 1) as a colorless oil. ^{1}H NMR (300 MHz, $CDCl_3$) δ 6.39 (t, J = 13.2 Hz, 1H), 4.33 (q, J = 7.2 Hz, 2H), 2.59 (t, J = 7.3 Hz, 2H), 1.55-1.50 (m, 2H), 1.39-1.25 (m, 9H), 0.91-0.86 (m, 3H); ^{13}C NMR (75 MHz, $CDCl_3$) δ 163.2 (t, J = 34.4 Hz), 131.2 (t, J = 27.1 Hz), 128.1 (t, J = 29.6 Hz, *Z*), 119.7 (t, J = 7.6 Hz), 111.5 (t, J = 252.3 Hz), 63.3, 63.2 (*Z*), 46.7 (*Z*), 40.7, 31.5, 31.4 (*Z*), 29.8, 28.9 (*Z*), 28.0, 27.6 (*Z*), 22.5, 14.0, 13.9; ^{19}F NMR (282 MHz, $CDCl_3$) δ -97.7 (*E*), -97.8 (*Z*).

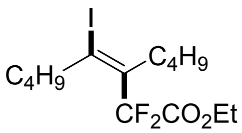

3ad

The product **3ad**[8] was purified with silica gel chromatography (Pe/EA = 20 : 1) as a colorless oil. ^{1}H NMR (300 MHz, $CDCl_3$) δ 4.31 (q, J = 7.2 Hz, 2H), 2.68-2.63 (m, 2H), 2.44-2.39 (m, 2H), 1.51-1.31 (m, 12H), 0.92 (q, J = 7.2 Hz, 6H); ^{13}C NMR (75 MHz, $CDCl_3$) δ 163.7 (t, J = 35.2 Hz), 136.5 (t, J = 22.6 Hz), 119.6 (t, J = 6.1 Hz, *Z*), 111.9 (t, J = 256.0 Hz), 63.1, 42.5 (t, J = 2.2 Hz), 39.1 (t, J = 3.7 Hz), 32.6, 30.1, 22.6, 21.7, 13.9, 13.8, 13.8; ^{19}F NMR (282 MHz, $CDCl_3$) δ -97.1 (*E*), -98.5 (*Z*).

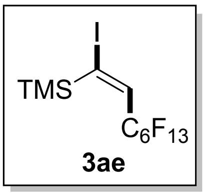

The product **3ae** was purified with silica gel chromatography (Pe) as a colorless oil. ^{1}H NMR (300 MHz, $CDCl_3$) δ 7.32 (t, *J* = 15.3 Hz, 1H), 6.78 (t, *J* = 13.1 Hz, 0.33H, *Z*), 0.33 (t, *J* = 1.5 Hz, 9H), 0.25 (s, 3H); ^{13}C NMR (75 MHz, $CDCl_3$) δ 138.9 (t, *J* = 23.3 Hz), 131.1 (t, *J* = 23.4 Hz, *Z*), 128.1 (t, *J* = 6.7 Hz), 1.14 (t, *J* = 3.7 Hz), -1.9 (*Z*); ^{19}F NMR (282 MHz, $CDCl_3$) δ -81.4 (m, 3H), -106.4 (t, *J* = 13.2 Hz, 1.48F, *E*), -109.9 (t, *J* = 12.9 Hz, 0.49F, *Z*), -122.3 (brs, 2F), -123.1 to -123.4 (m, 4F), -126.7 (m, 2F); HRMS (EI) (*m/z*): $[M]^+$ calcd for $C_{11}H_{10}F_{13}SiI$: 543.9383, found: 543.9350.

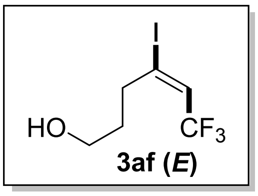

The product **3af** was purified with silica gel chromatography (Pe/EA = 5 : 1) as a colorless oil. ^{1}H NMR (300 MHz, $CDCl_3$) δ 6.42 (q, *J* = 7.7 Hz, 1H), 3.68 (brs, 2H), 2.74 (t, *J* = 7.5 Hz, 2H), 1.88-1.79 (m, 2H), 1.57 (brs, 1H); ^{13}C NMR (75 MHz, $CDCl_3$) δ 129.5 (q, *J* = 34.4 Hz), 121.8 (q, *J* = 274.2 Hz), 119.7 (q, *J* = 6.2 Hz), 61.1, 37.4, 32.4; ^{19}F NMR (282 MHz, $CDCl_3$) δ -58.4; HRMS (EI) (*m/z*): $[M-C_2H_4O]^+$ calcd for $C_4H_4F_3I$: 235.9304, found: 543.9287; $[M-I]^+$ calcd for $C_6H_8F_3O$: 153.0522, found: 153.0518.

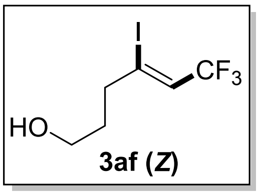

The product **3af** was purified with silica gel chromatography (Pe/EA = 5 : 1) as a colorless oil. ^{1}H NMR (300 MHz, $CDCl_3$) δ 6.40-6.33 (m, 1H), 3.69 (t, *J* = 6.1 Hz, 2H), 2.77-2.72 (m, 2H), 1.89-1.80 (m, 2H), 1.31 (brs, 1H); ^{13}C NMR (150 MHz, $CDCl_3$) δ 125.5 (q, *J* = 35.7 Hz), 121.3 (q, *J* = 271.2 Hz), 114.9 (q, *J* = 6.3 Hz), 60.7, 43.3, 31.7; ^{19}F NMR (282 MHz, $CDCl_3$) δ -60.6; HRMS (EI) (*m/z*): $[M-C_2H_4O]^+$ calcd for $C_4H_4F_3I$: 235.9304, found: 543.9295; $[M-I]^+$ calcd for $C_6H_8F_3O$: 153.0522, found: 153.0526.

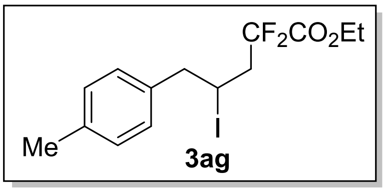

The product **3ag** was purified with silica gel chromatography (Pe/EA = 20 : 1) as a colorless oil. ^{1}H NMR (300 MHz, $CDCl_3$) δ 7.14 (d, *J* = 8.0 Hz, 2H), 7.08 (d, *J* = 8.0 Hz, 2H) 4.37-4.29 (m, 3H), 3.25-3.12 (m, 2H), 2.99-2.69 (m, 2H), 2.33 (s, 3H), 1.37 (t, *J* = 7.2 Hz, 3H); ^{13}C NMR (75 MHz, $CDCl_3$) δ 163.4 (t, *J* = 32.3 Hz), 136.8, 135.7, 129.2, 128.8, 115.2 (dd, *J* = 253.5, 251.5 Hz), 63.2, 46.8, 44.2 (t, *J* = 23.4 Hz), 22.3 (t, *J* = 3.8 Hz), 21.1, 13.8; ^{19}F NMR (282 MHz, $CDCl_3$) δ -102.2 (d, *J* = 263.3 Hz, 1F), -107.0 (d, *J* = 263.2 Hz, 1F); HRMS (EI) (*m/z*): $[M]^+$ calcd for $C_{14}H_{17}F_2IO_2$: 382.0236, found: 382.0221.

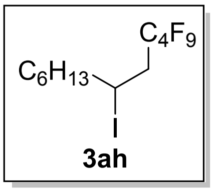

The product **3ah**[9] was purified with silica gel chromatography (Pe) as a colorless oil. ^{1}H NMR (300 MHz, $CDCl_3$) δ 4.38-4.29 (m, 1H), 3.02-2.67 (m, 2H), 1.88-1.72 (m, 2H), 1.52-1.29 (m, 8H), 0.89 (t, *J* = 6.7 Hz, 3H); ^{19}F NMR (282 MHz, $CDCl_3$) δ -81.5 to -81.6 (m, 3F), -112.5 (dm, *J* = 270.7 Hz,

1F), -115.4 (dm, J = 270.9 Hz, 1F), -125.0 to -125.1 (m, 2F), -126.3 to 126.5 (m, 2F).

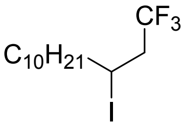

3ai

The product **3ai**[10] was purified with silica gel chromatography (Pe) as a colorless oil. ^{1}H NMR (300 MHz, $CDCl_3$) δ 4.42-4.15 (m, 1H), 3.00-2.71 (m, 2H), 1.85-1.68 (m, 2H), 1.58-1.49 (m, 1H), 1.43-1.27 (m, 15H), 0.88 (t, J = 6.7 Hz, 3H); ^{19}F NMR (282 MHz, $CDCl_3$) δ -64.4.

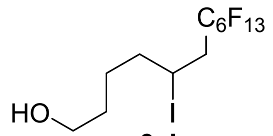

3aj

The product **3aj**[11] was purified with silica gel chromatography (Pe/EA = 5 : 1) as a colorless oil. ^{1}H NMR (300 MHz, $CDCl_3$) δ 4.39-4.30 (m, 1H), 3.68 (brs, 2H), 3.03-2.68 (m, 2H), 1.89-1.80 (m, 2H), 1.65-1.48 (m, 4H), 1.34 (brs, 1H); ^{19}F NMR (282 MHz, $CDCl_3$) δ -81.3 (tt, J = 10.0, 2.1 Hz, 3F), -112.2 (dm, J = 269.8 Hz, 1F), -115.2 (dm, J = 269.9 Hz, 1F), -122.3 (brs, 2F), -123.4 (brs, 2F), -124.1 to -124.2 (m, 2F), -126.6 to -126.7 (m, 2F).

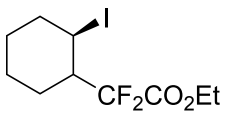

3ak

The product **3ak**[12] was purified with silica gel chromatography (Pe/EA = 20 : 1) as a colorless oil. ^{1}H NMR (300 MHz, $CDCl_3$) δ 4.33 (q, J = 7.1 Hz, 2H), 4.07 (td, J = 10.3, 4.2 Hz, 1H), 2.77-2.65 (m, 1H), 2.38-2.34 (m, 1H), 2.15-2.11 (m, 1H), 1.86-1.75 (m, 4H), 1.37 (t, J = 7.2 Hz, 5H); ^{19}F NMR (282 MHz, $CDCl_3$) δ -106.0 (d, J = 266.4 Hz, *trans*), -109.3 (d, J = 263.3 Hz, *cis*), -110.6 (d, J = 263.2 Hz, *cis*).

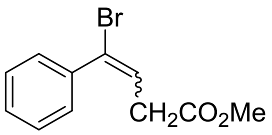

3al

The product **3al** was purified with silica gel chromatography (Pe/EA = 20 : 1) as a colorless oil. ^{1}H NMR (300 MHz, $CDCl_3$) δ 7.58-7.54 (m, 2H), 7.38-7.31 (m, 10.5H), 6.48 (t, J = 6.5 Hz, 1H), 6.39 (t, J = 7.7 Hz, 1.5H), 3.75 (s, 3H), 3.70 (s, 4.5H), 3.45 (d, J = 6.5 Hz, 2H), 3.06 (d, J = 7.7 Hz, 3H); ^{13}C NMR (75 MHz, $CDCl_3$) δ171.1, 170.7, 139.3, 137.9, 128.9, 128.9, 128.5, 128.3, 128.2, 127.6, 125.4, 123.9, 123.2, 52.2, 37.8, 35.9; HRMS (ESI) (m/z): [M+K]+ calcd for $C_{11}H_{11}BrO_2$: 253.9937, found: 253.9936.

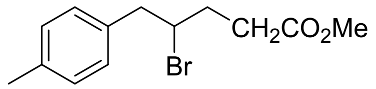

3am

The product **3al** was purified with silica gel chromatography (Pe/EA = 20 : 1) as a colorless oil. ^{1}H NMR (300 MHz, $CDCl_3$) δ 7.15-7.09 (m, 4H), 4.28-4.19 (m, 1H), 3.67 (s, 3H) 3.16 (qd, J = 14.2, 7.1 Hz, 2H), 2.70-2.46 (m, 2H), 2.34 (s, 3H), 2.24-2.21 (m, 1H), 2.07-1.94 (m, 1H); ^{13}C NMR (75 MHz, $CDCl_3$) δ173.1, 136.5, 134.9, 129.1, 129.0, 56.4, 51.6, 45.3, 33.0, 32.1, 21.0; HRMS (EI) (m/z): [M]+ calcd for $C_{13}H_{17}BrKO_2$: 323.0044, found: 323.0037.

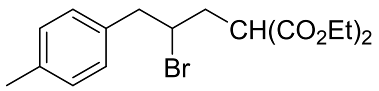

3an

The product **3an** was purified with silica gel chromatography (Pe/EA = 10 : 1) as a colorless oil. ^{1}H NMR (300 MHz, $CDCl_3$) δ 7.14-7.08 (m, 4H), 4.25-4.11 (m, 5H), 3.80 (dd, *J* = 10.5, 4.0 Hz, 1H), 3.17 (dd, *J* = 7.0, 1.9 Hz, 2H), 2.51 (ddd, *J* = 14.8, 10.5, 2.9 Hz, 1H), 2.33 (s, 3H), 2.23 (ddd, *J* = 14.9, 11.0, 4.0 Hz, 1H), 1.25 (q, *J* = 7.1 Hz, 6H); ^{13}C NMR (75 MHz, $CDCl_3$) δ 169.0, 168.7, 136.6, 134.7, 129.2, 129.1, 61.7, 61.6, 54.5, 50.7, 45.4, 37.1, 21.1, 14.0; HRMS (EI) (m/z): [M]+ calcd for $C_{13}H_{23}BrKO_4$: 409.0411, found: 409.0390.

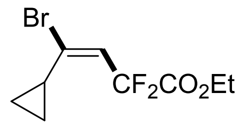

The product was purified with silica gel chromatography (Pe/EA = 20 : 1) as a colorless oil. ^{1}H NMR (300 MHz, $CDCl_3$) δ 6.17 (t, *J* = 13.3 Hz, 1H) 4.35 (q, *J* = 7.1 Hz, 2H), 2.15-2.07 (m, 1H), 1.36 (t, *J* = 7.1 Hz, 3H), 0.99-0.81 (m, 4H); ^{13}C NMR (150 MHz, $CDCl_3$) δ 163.4 (t, *J* = 34.8 Hz), 143.8, 122.4 (t, *J* = 27.8 Hz), 111.9 (t, *J* = 250.8 Hz), 63.3, 15.7 (t, *J* = 3.1 Hz), 13.9, 8.5; ^{19}F NMR (282 MHz, $CDCl_3$) δ -97.8; HRMS (EI) (*m/z*): $[M]^+$ calcd for $C_9H_{11}BrF_2O_2$: 267.99050, found: 267.99045.

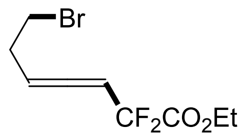

The product was purified with silica gel chromatography (Pe/EA = 20 : 1) as a colorless oil. ^{1}H NMR (300 MHz, $CDCl_3$) δ 5.73-5.63 (m, 1H) 5.62-5.52 (m, 1H), 4.33 (q, *J* = 7.1 Hz, 2H), 3.42 (t, *J* = 6.9 Hz, 2H), 2.65 (qd, *J* = 6.9, 2.9 Hz, 2H), 1.35 (t, *J* = 7.1 Hz, 3H), 0.99-0.81 (m, 4H); ^{13}C NMR (75 MHz, $CDCl_3$) δ 205.4, 163.4 (t, *J* = 33.9 Hz), 111.8 (t, *J* = 249.4 Hz), 95.3, 89.1 (t, *J* = 30.9 Hz), 63.1, 31.0 (t, *J* = 2.3 Hz), 30.5, 13.9; ^{19}F NMR (282 MHz, $CDCl_3$) δ -100.0; HRMS (EI) (*m/z*): $[M]^+$ calcd for $C_9H_{11}BrF_2O_2$: 267.9905, found: 267.9884.

Palladium-catalyzed Suzuki coupling of **3a**[2]

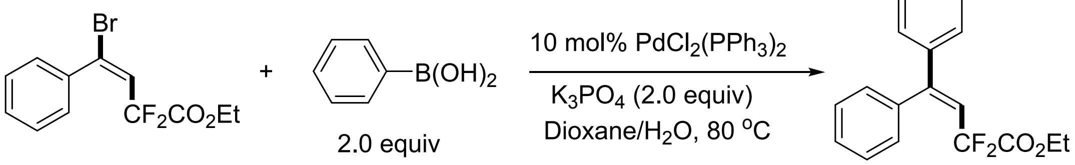

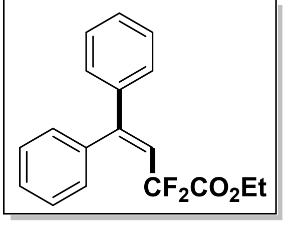

To a 10 mL of Schlenk tube were added phenyl boronic acid (2.0 equiv, 0.4 mmol, 48.8 mg), $PdCl_2(PPh_3)_2$ (10 mol%, 0.02 mol, 7.0 mg), K_3PO_4 (2.0 equiv, 0.4 mmol, 84.8 mg). The mixture was evacuated and backfilled with N_2 for 3 times. 1,4-dioxane (2 mL), H_2O (50 *u*L) and **3a** (1.0 equiv, 0.2 mmol, 61.0 mg) were added subsequently. The mixture was stirred at 80 °C for 16 hours. After cooling to room temperature, the solvent was removed under vacuum and purified by flash column chromatography on silica gel (PE/EA = 20 :

1) to give the desired product. as a colorless oil (52.1 mg) with 86% yield. ^{1}H NMR (300 MHz, $CDCl_3$) δ 7.38-7.31 (m, 6H) 7.29-7.25 (m, 2H), 7.22-7.19 (m, 2H), 6.28 (t, *J* = 11.8 Hz, 1H), 3.91 (q, *J* = 7.2 Hz, 2H), 1.17 (t, *J* = 7.2 Hz, 3H); ^{19}F NMR (282 MHz, $CDCl_3$) δ -91.1.

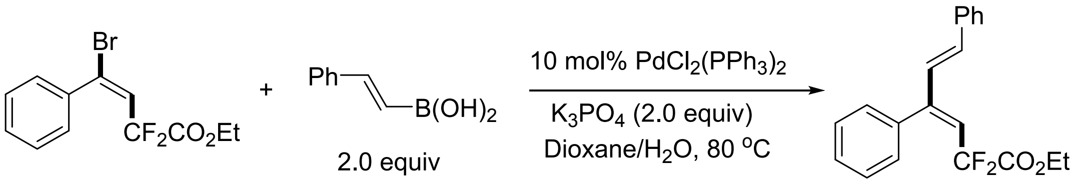

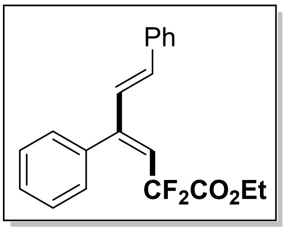

To a 10 mL of Schlenk tube were added (*E*)-phenyl vinylboronic acid (2.0 equiv, 0.4 mmol, 58.2 mg), $PdCl_2(PPh_3)_2$ (10 mol%, 0.02 mol, 7.0 mg), K_3PO_4 (2.0 equiv, 0.4 mmol, 84.8 mg). The mixture was evacuated and backfilled with N_2 for 3 times. 1,4-dioxane (2 mL), H_2O (50 *u*L) and **3a** (1.0 equiv, 0.2 mmol, 61.0 mg) were added subsequently. The mixture was stirred at 80 °C for 4 hours. After cooling to room temperature, the solvent was removed under vacuum and purified by flash column chromatography on silica gel (PE/EA = 20 : 1) to give the desired product as a pale yellow oil (42.1 mg) with 64% yield. ^{1}H NMR (300 MHz, $CDCl_3$) δ 7.42-7.38 (m, 3H) 7.36-7.27 (m, 5H), 7.22-7.19 (m, 2H), 7.01 (d, *J* = 15.9 Hz, 1H), 6.21 (d, *J* = 15.9 Hz, 1H), 6.04 (t, *J* = 12.1 Hz, 1H), 3.94 (q, *J* = 7.2 Hz, 2H), 1.20 (t, *J* = 7.2 Hz, 3H); ^{19}F NMR (282 MHz, $CDCl_3$) δ -92.6.

Palladium-catalyzed Sonogashira coupling of **3a**

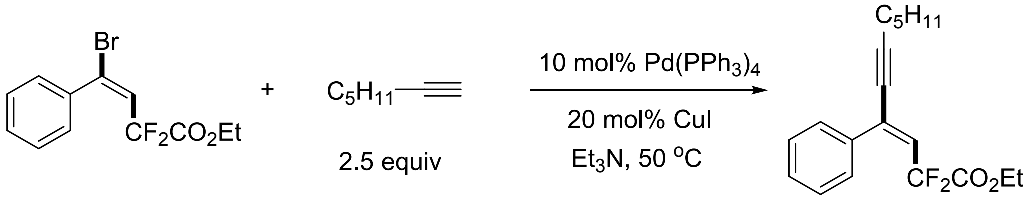

To a 10 mL of Schlenk tube were added $PdCl_2(PPh3)_2$ (10 mol%, 0.02 mol, 7.0 mg), CuI (20 mol%, 0.04 mmol, 7.8 mg). The mixture was evacuated and backfilled with N_2 for 3 times. Et_3N (2.0 mL), **3a** (1.0 equiv, 0.2 mmol, 61.0 mg) and 1-heptyne (2.5 equiv, 0.5 mmol, 48.0 mg) were added subsequently. The mixture was stirred at 50 °C for 16 hours. After cooling to room temperature, the solvent was removed under vacuum and purified by flash column chromatography on silica gel (PE/EA = 20 : 1) to give the desired product. ^{1}H NMR (300 MHz,

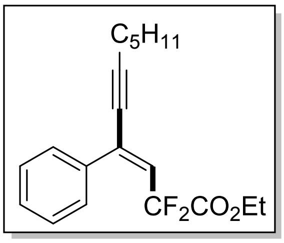

$CDCl_3$) δ 7.38-7.32 (m, 5H), 6.17 (t, *J* = 12.3 Hz, 1H), 3.89 (q, *J* = 7.2 Hz, 2H), 2.34 (t, *J* = 7.1 Hz, 2H), 1.58-1.53 (m, 2H), 1.38-1.33 (m, 4H), 1.12 (t, *J* = 7.2 Hz, 3H), 0.90 (t, *J* = 7.1 Hz, 3H); ^{13}C NMR (75 MHz, $CDCl_3$) δ 163.0 (t, *J* = 33.8 Hz), 136.1, 134.3 (t, *J* = 10.2 Hz), 128.9, 128.5 (t, *J* = 2.1 Hz), 128.1, 125.9 (t, *J* = 28.3 Hz), 112.0 (t, *J* = 244.9 Hz), 95.8 (t, *J* = 1.5 Hz), 80.9 (t, *J* = 2.3 Hz), 62.9, 31.1, 28.1, 22.2, 19.7, 14.0, 13.6; ^{19}F NMR

(282 MHz, $CDCl_3$) δ -92.1.

4. Mechanistic studies

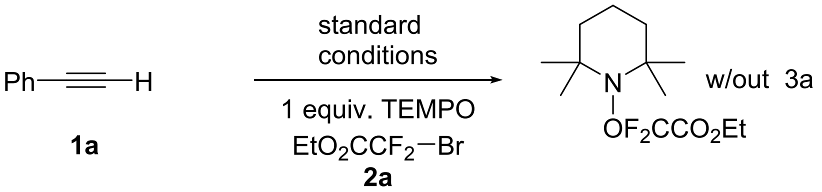

$CoBr_2$ (1.3 mg, 0.006 mmol, 0.02 equiv), dppbz (2.7 mg, 0.006 mmol, 0.02 equiv), Zn (1.0 mg, 0.015 mmol, 0.05 equiv) were dissolved in acetone and stirred for 2 minutes. **1a** was added into the system, followed with **2a** (0.45 mmol, 1.5 equiv) and TEMPO (0.3 mmol, 1.0 equiv) [Note: **2a** and TEMPO dissolved in 0.2 mL acetone, then added into the system]. The mixture was stirred at room temperature for 3 hours. No product **3a** was detected by GC-MS analysis, but TEMPO-CF_2CO_2Et.

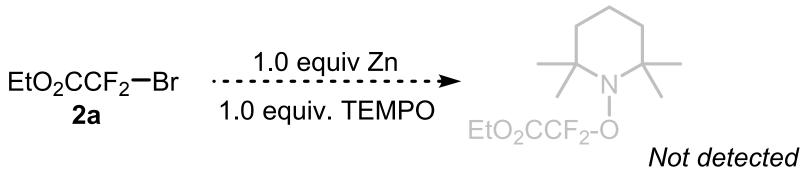

Zn (6.4 mg, 0.1 mmol, 1.0 equiv) was added into the reaction tube (N_2 protected), and acetone/H_2O (30:1, 0.6 mL) was added, followed by 0.2 mL acetone containing 2a (20.6 mg, 0.1 mmol, 1.0 equiv) and TEMPO (0.1 mmol, 15.4 mg, 1.0 equiv). The mixture stirred for 3 hours at room temperature. No TEMPO-CF2CO2Et was detected by GC-MS analysis.

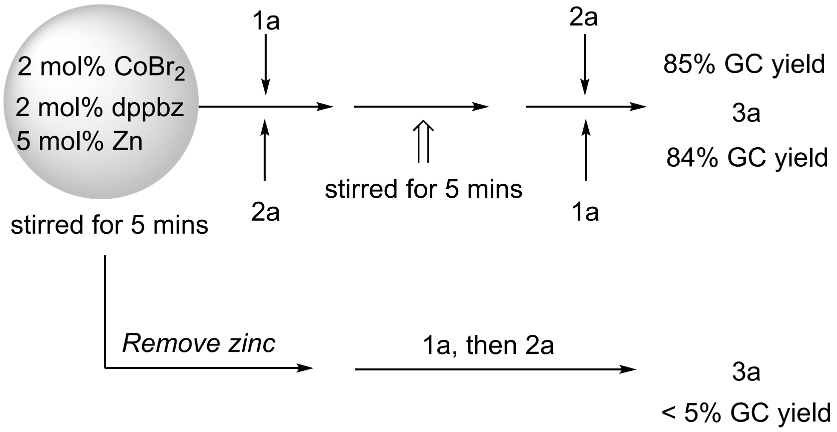

$CoBr_2$ (1.3 mg, 0.006 mmol, 0.02 equiv), dppbz (2.7 mg, 0.006 mmol, 0.02 equiv), Zn (1.0 mg, 0.015 mmol, 0.05 equiv) were dissolved in acetone and stirred for 5 minutes. **1a** (**2a**) was added into the system and stirred for another 5 minutes. Then **2a** (**1a**) was added into the system and reacted for 3 hours at room temperature to give the desired product **3a** with 85% (84%) GC yield. While when the solution of $CoBr_2$ (1.3 mg, 0.006 mmol, 0.02 equiv), dppbz (2.7 mg, 0.006 mmol,

0.02 equiv), Zn (1.0 mg, 0.015 mmol, 0.05 equiv) reacted in acetone for 5 minutes was transferred into a new reaction to remove the zinc, **3a** was obtained less than 5% GC yield after 3 hours reaction.

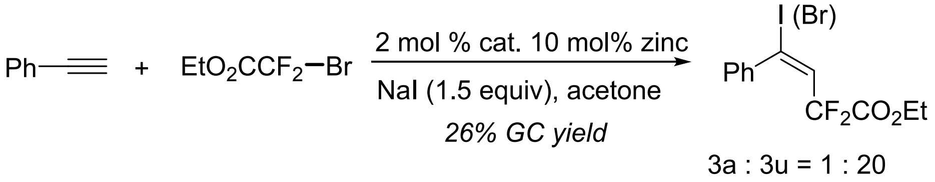

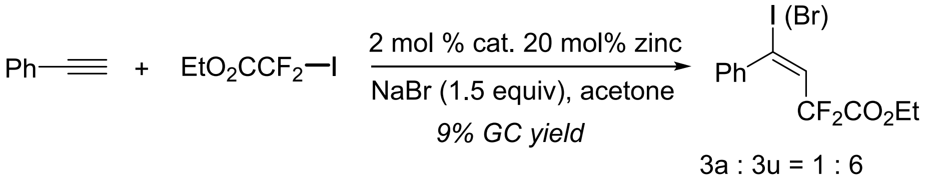

$CoBr_2$ (1.3 mg, 0.006 mmol, 0.02 equiv), dppbz (2.7 mg, 0.006 mmol, 0.02 equiv), Zn (2.0 mg, 0.03 mmol, 0.10 equiv), NaI (NaBr) (0.45 mmol, 1.5 equiv) were dissolved in 1 mL acetone and stirred for 2 minutes. Phenyl acetylene (0.3 mmol, 1.0 equiv) was added into the system, followed by EtO_2CF_2Br (EtO_2CF_2I) (0.45 mmol, 1.5 equiv). The mixture was stirred at room temperature for 3 hours. **3a** and **3u** were obtained with 26% (9%) GC yield with the ratio 1 : 20 (1 : 6).

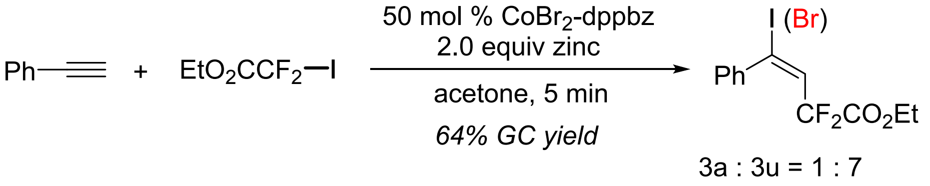

$CoBr_2$ (0.05 mmol, 0.5 equiv), dppbz (0.05 mmol, 0.5 equiv), Zn (0.2 mmol, 2.0 equiv) were dissolved in 3 mL acetone and stirred for 5 minutes. Phenyl acetylene (0.1 mmol, 1.0 equiv) was added into the system, followed by EtO_2CF_2I (0.15 mmol, 1.5 equiv). The mixture was stirred at room temperature for 3 hours. **3a** and **3u** were obtained with 64% GC yield with the ratio 1 : 7.

^{31}P NMR and ^{1}H NMR monitor:

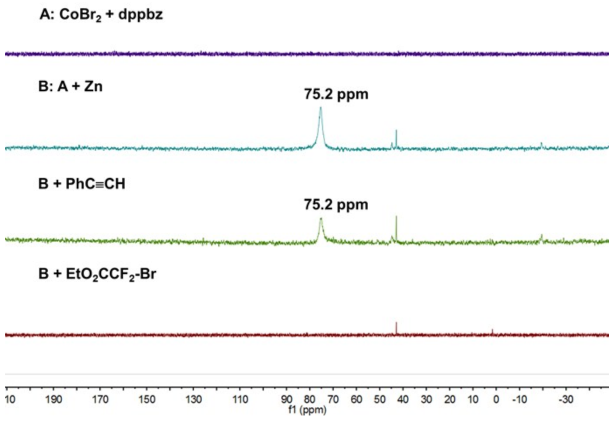

A: $CoBr_2$+dppbz: $CoBr_2$ (0.02 mmol) and dppbz (0.02 mmol) were dissolve in d^{6}-acetone (1.0 mL) and stirred for 15 minutes.

B: $CoBr_2$+dppbz+Zn: $CoBr_2$ (0.02 mmol) and dppbz (0.02 mmol) and zinc (0.04 mmol) were dissolve in d^{6}-acetone (1.0 mL) and stirred for 15 minutes.

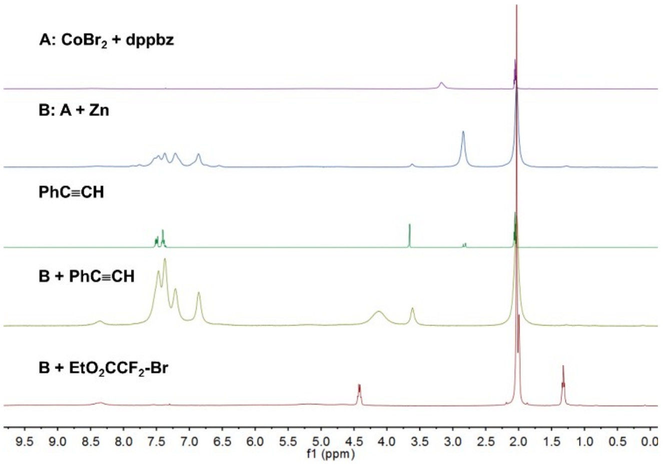

B + PhCCH: $CoBr_2$ (0.02 mmol) and dppbz (0.02 mmol) and zinc (0.04 mmol) were dissolve in d[6]-acetone (1.0 mL) and stirred for 15 minutes, then added PhCCH (0.02 mmol)

B + EtO_2CCF_2Br: $CoBr_2$ (0.05 mmol) and dppbz (0.05 mmol) and zinc (0.1 mmol) were dissolve in d[6]-acetone (3 mL) and stirred for 15 minutes, then added EtO_2CCF_2Br (0.06 mmol) reacted for another 2 minutes.

5. Kinetic experiments

$CoBr_2$ (0.01 or 0.02 or 0.04 equiv), dppbz (0.01 or 0.02 or 0.04 equiv), Zn (3.8 mg, 0.1 equiv) were dissolved in acetone/H_2O (30:1, 1.2 mL) and stirred for 5 minutes. Dodecane (0.6 mmol, 1.0 equiv) was added as the internal standard. Then phenyl acetylene (0.6 mmol, 1.0 equiv) was added into the system. $BrCF_2CO_2Et$ (0.9 mmol, 1.5 equiv) added quickly and the reaction started. Taken about 2 *u*L reaction solution for GC analysis at the indicated times: for $CoBr_2$ (0.01 equiv) : 1 min, 2 min, 3 min, 4 min, 5 min, 6 min, 7 min, 8 min, 10 min, 12 min, 14 min, 16 min, 20 min, 25 min, 30 min, 40 min, 50 min, 60 min, 80 min, 100 min, 120 min, 150 min, 180 min; for $CoBr_2$ (0.02 equiv):, 0.5 min, 1 min, 1.5 min, 2 min, 2.5 min, 3 min, 3.5 min, 4 min, 5 min, 6 min, 8 min, 10 min, 12 min, 14 min, 20 min, 25 min, 30 min, 40 min, 50 min, 70 min, 90 min, 110 min; for $CoBr_2$ (0.02 equiv): 0.5 min, 1 min, 1.5 min, 2 min, 2.5 min, 3 min, 3.5 min, 4 min, 4.5 min, 5 min, 5.5 min, 6 min, 6.5 min, 7 min, 7.5 min, 8 min, 9 min, 10 min, 11 min, 12 min, 14 min. Repeat two times for every concentrations.

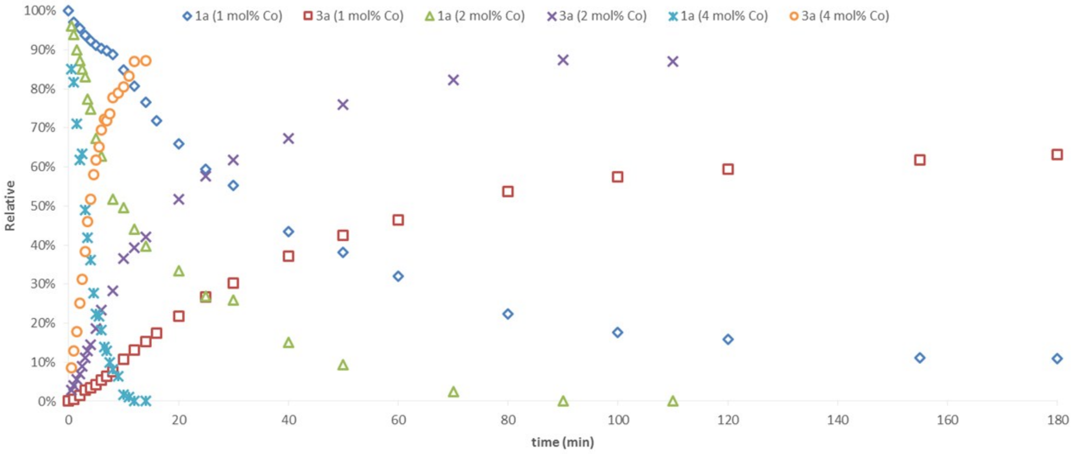

Figure 1. GC-determined relative amount of phenyl acetylene **1a** and product **3a** vs time with different cobalt-catalyst different concentrations.

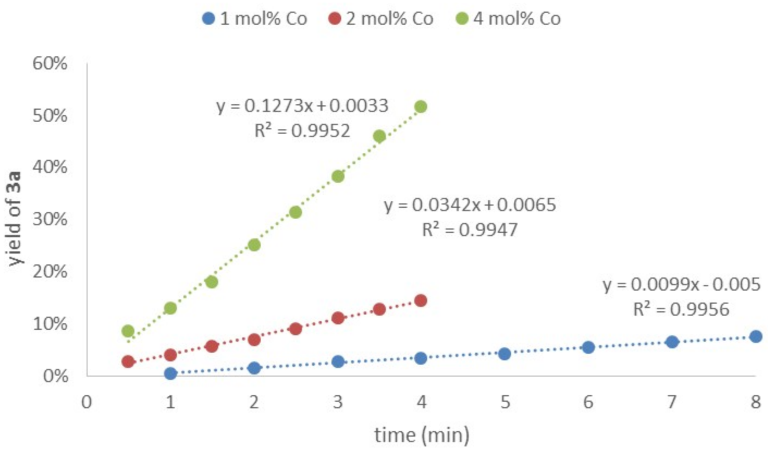

Figure 2. Initial kinetic rate calculated with different cobalt-catalyst concentrations.

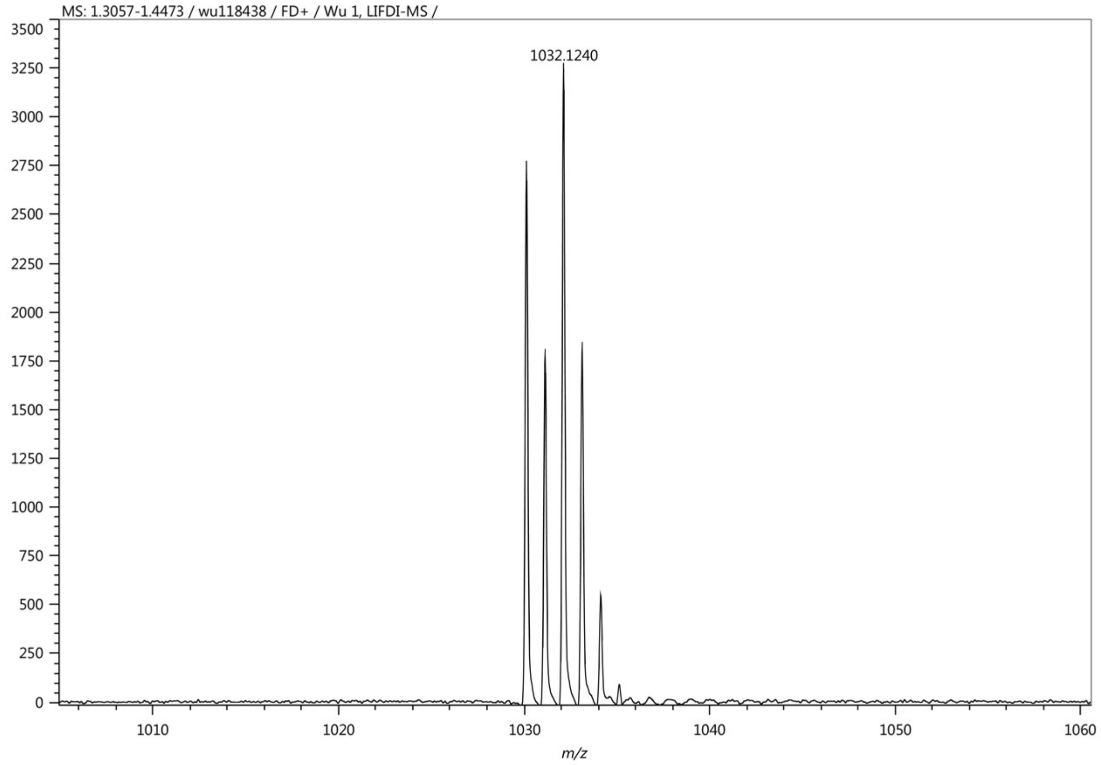

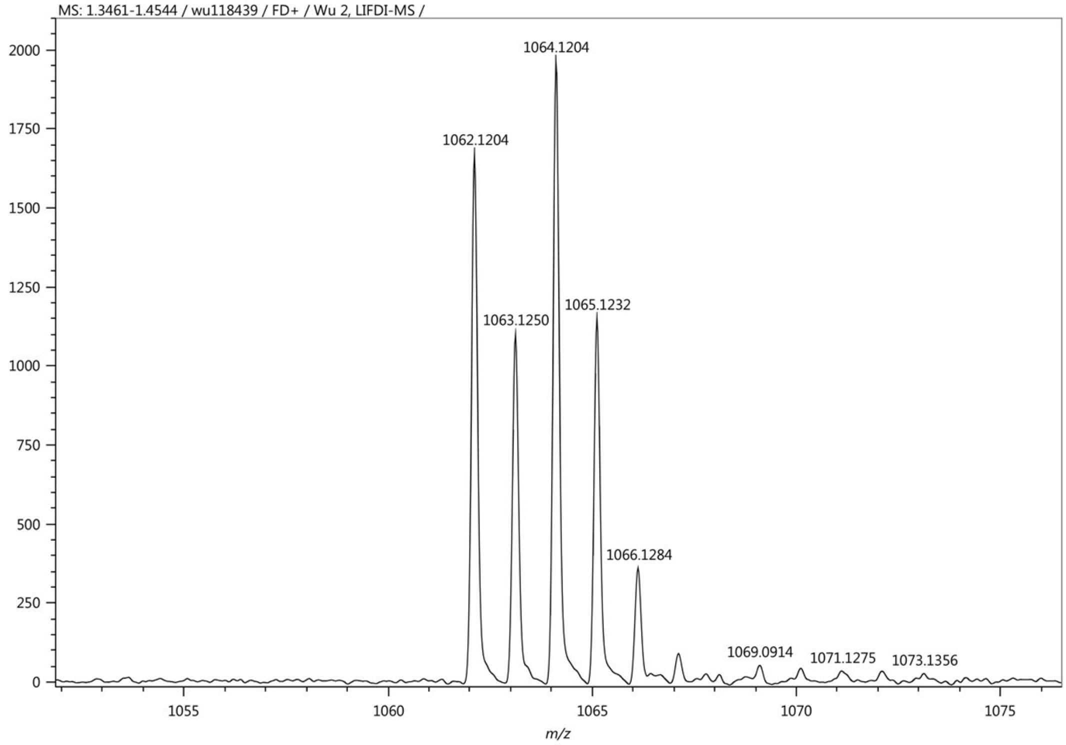

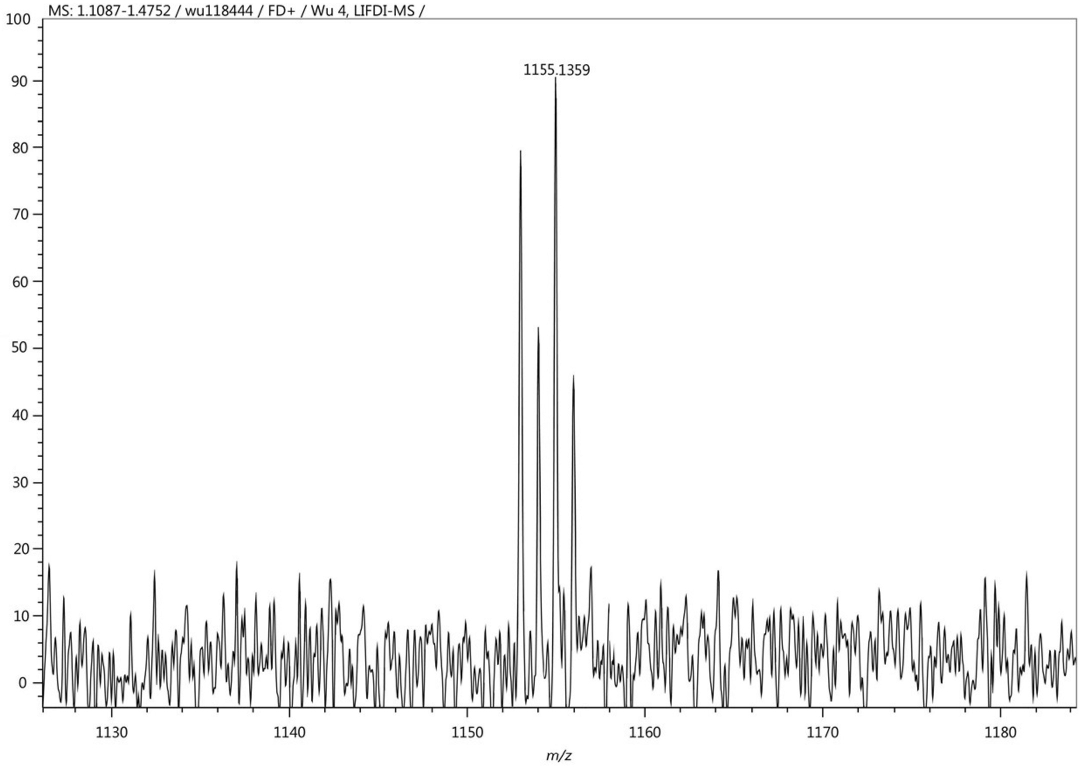

Figure 3. LIFDI mass-spectra of species: $[Co^{II}Br(dppbz)_2]^+$ (top): calcd. 1032.1196, found 1032.1240; $[Co^{III}(dppbz)_2Br(O_2)]^+$ (centre): calcd. 1064.1095, found 1064.1204; and $[EtO_2CCF_2Co^{III}Br(dppbz)_2]^+$ (bottom): calcd. 1155.1454, found 1155.1359.

6. References

1. Belhomme, M.-C.; Dru, D.; Xiong, H.-Y.; Chard, D.; Besset, T.; Poisson, T.; Pannecoucke, X. *Synthesis* **2014**, 1859.
2. Li, G.; Cao, Y.-X.; Luo, C.-G.; Su, Y.-M.; Li, Y.; Lan, Q.; Wang, X.-S. *Org. Lett.* **2016**, *18*, 4806.
3. Konno, T.; Chae, J.; Kanda, M.; Nagai, G.; Tamura, K.; Ishihara, T.; Yamanaka, H. *Tetrahedron* **2003**, *59*, 7571.
4. Saito, S.; Kawasaki, T.; Tsuboya, N.; Yamamoto, Y. *J. Org. Chem*. **2001**, *66*, 796.
5. Beniazza, R.; Atkinson, R.; Absalon, C.; Castet, F.; Denisov, S. A.; McClenaghan, N. D.; Lastécouères, D.; Vincent, J.-M. *Adv. Synth. Catal*. **2016**, *358*, 2949.
6. Hang, Z.; Li, Z.; Liu, Z.-Q. *Org. Lett*. **2014**, *16*, 3648.
7. Hu, C.-M.; Chen, J. *J. Fluorine Chem*., **1994**, *66*, 79.
8. Xu, T.; Cheung, C.; Hu, X. *Angew. Chem. Int. Ed.* **2014**, *53*, 4910.
9. Zeng, R.; Fu, C.; Ma, S. *Angew. Chem. Int. Ed.* **2012**, *51*, 3888.
10. Iqbal, N.; Choi, S.; Kim, E.; Cho, E. J. *J. Org. Chem*. **2012**, *77*, 11383.
11. Wallentin, C.-J.; Nguyen, J. D.; Finkbeiner, P.; Stephenson, C. R. J. *J. Am. Chem. Soc*. **2012**, *134*, 8875.
12. Nguyen, J. D.; Tucker, J. W.; Konieczynska, M. D.; Stephenson, C. R. J. *J. Am. Chem. Soc.* **2011**, *133*, 4160.

7. ^{1}H, ^{13}C, and ^{19}F Spectra

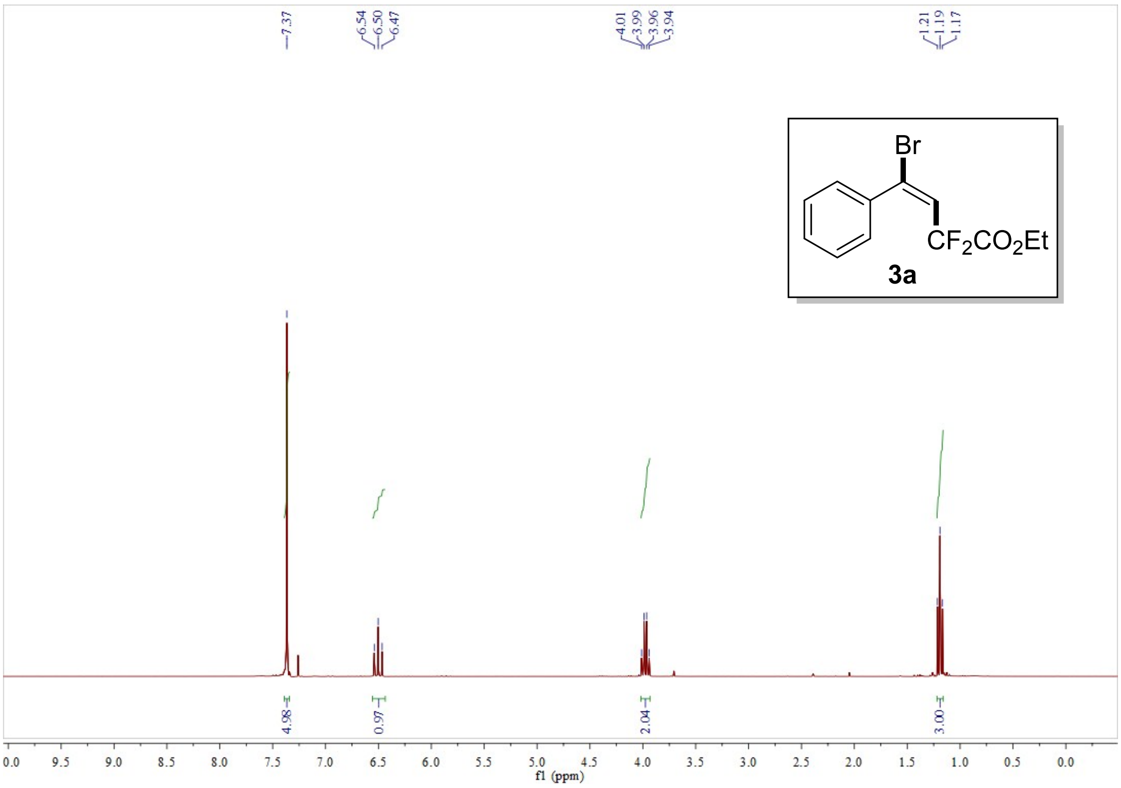

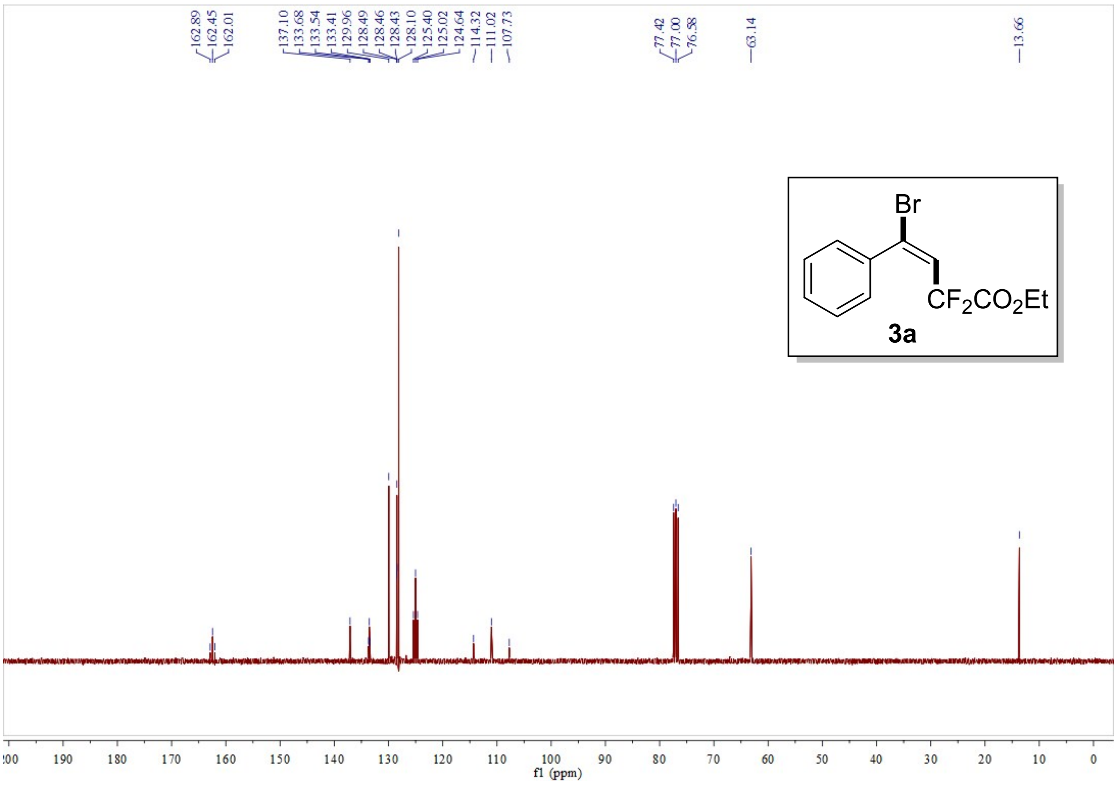

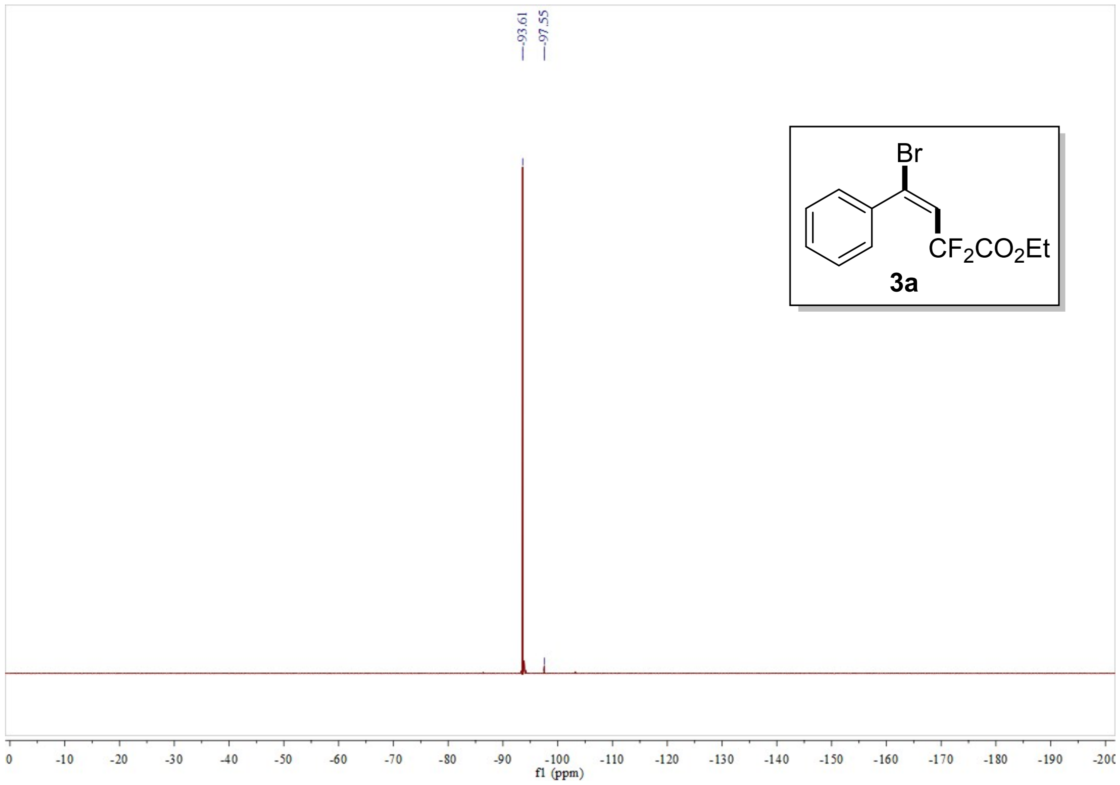

—93.61
—97.55
Br
CF2CO2Et
3a
0 -10 -20 -30 -40 -50 -60 -70 -80 -90 -100 -110 -120 -130 -140 -150 -160 -170 -180 -190 -200
f1 (ppm)

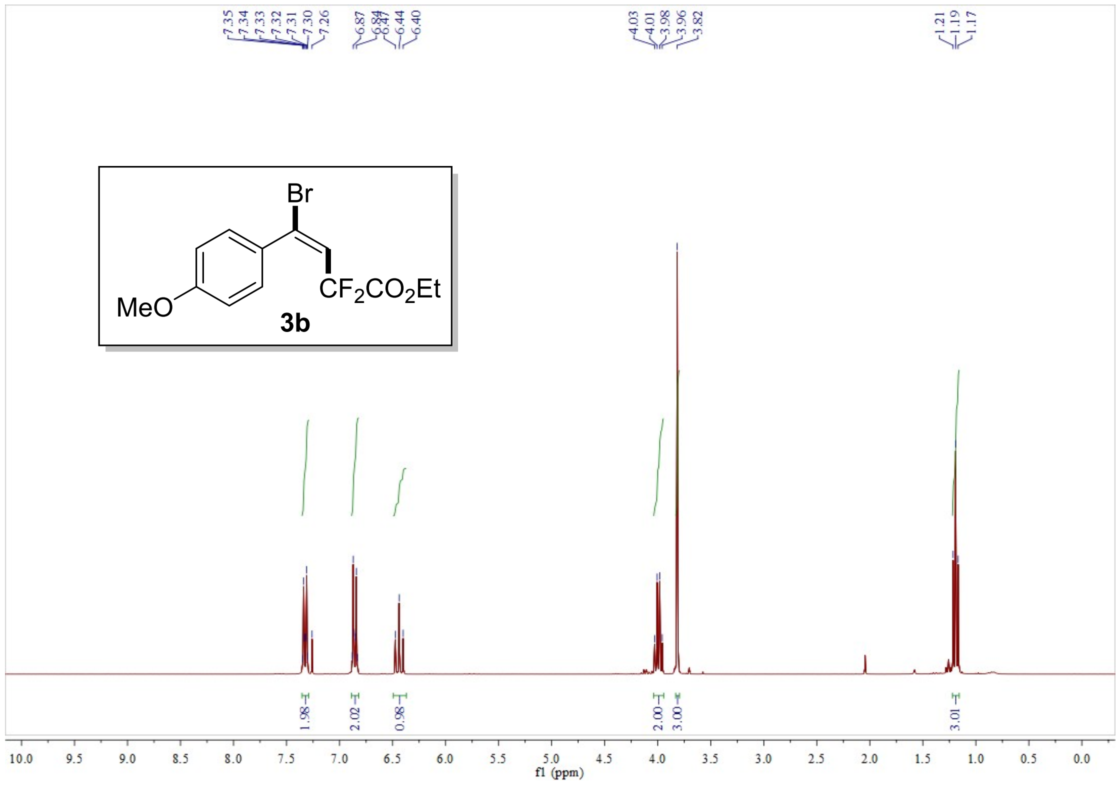

7.35 7.34 7.33 7.32 7.31 7.30 7.26
6.87 6.84 6.47 6.44 6.40
4.03 4.01 3.98 3.96 3.82
1.21 1.19 1.17
Br
MeO
CF2CO2Et
3b
1.98 2.02 0.98 2.00 3.00 3.01
10.0 9.5 9.0 8.5 8.0 7.5 7.0 6.5 6.0 5.5 5.0 4.5 4.0 3.5 3.0 2.5 2.0 1.5 1.0 0.5 0.0
f1 (ppm)

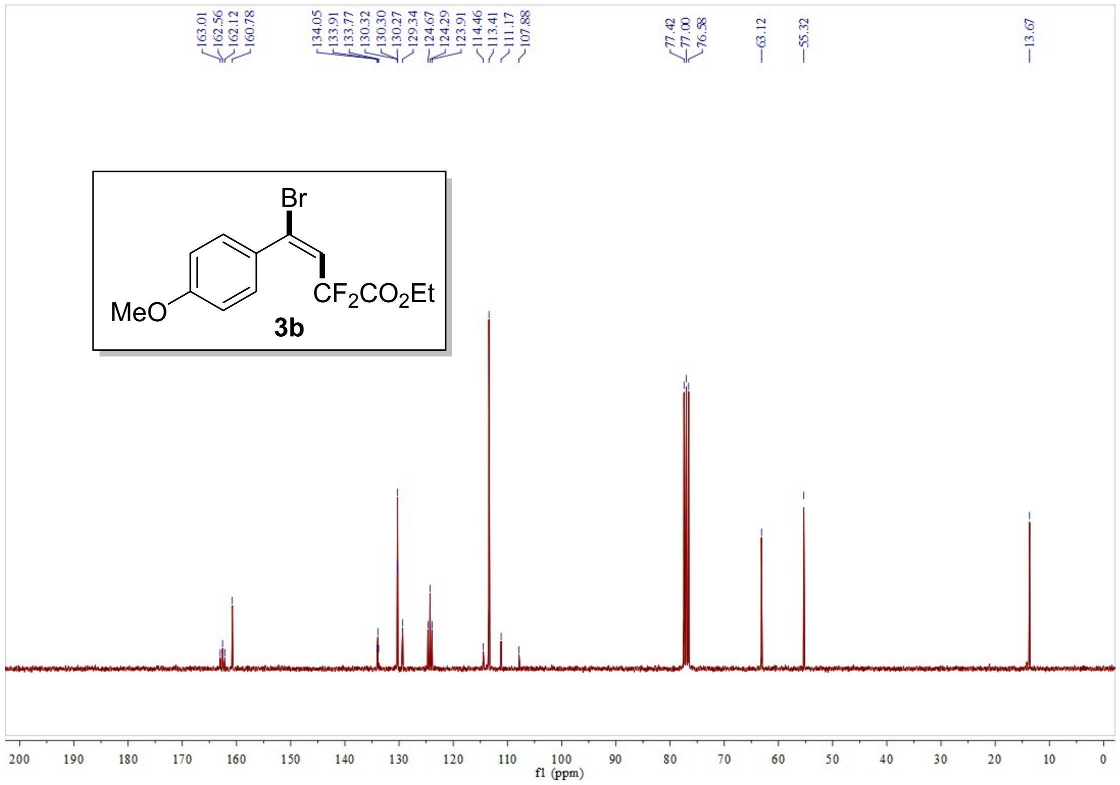
163.01
162.56
162.12
160.78
134.05
133.91
133.77
130.32
130.30
130.27
129.34
124.67
124.29
123.91
114.46
113.41
111.17
107.88
77.42
77.00
76.58
63.12
55.32
13.67
Br
MeO
CF2CO2Et
3b
f1 (ppm)

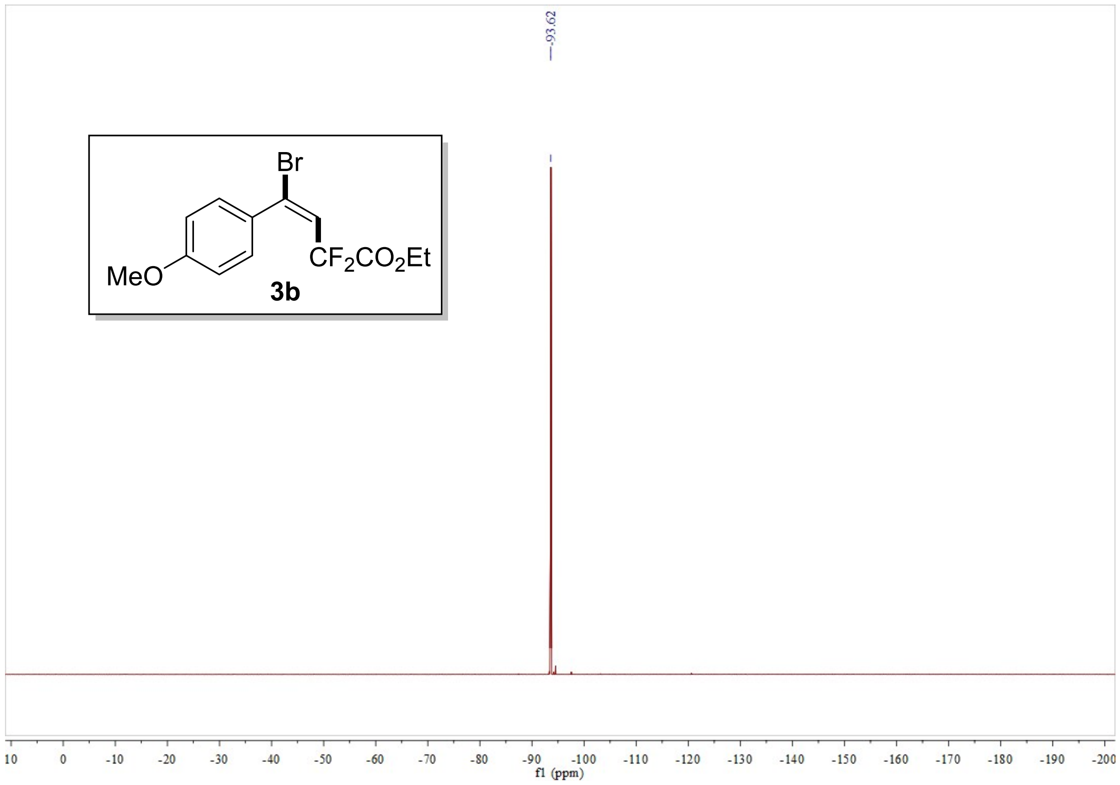
-93.62
Br
MeO
CF2CO2Et
3b
f1 (ppm)

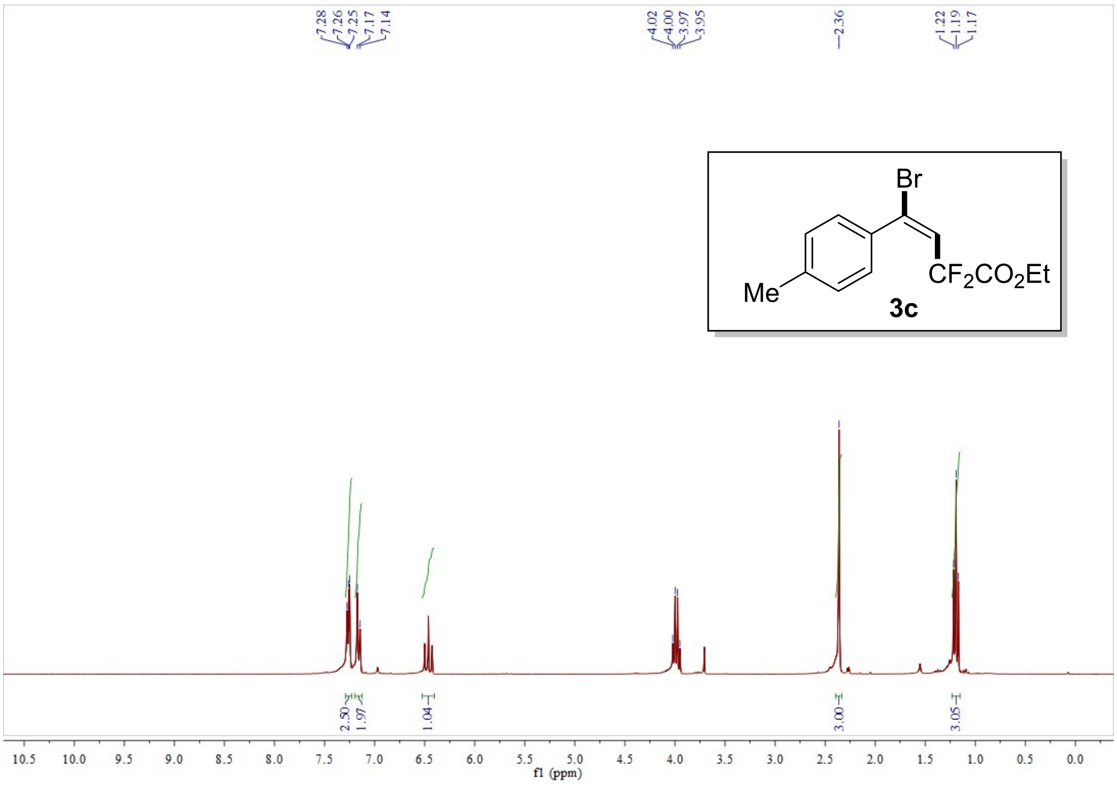
7.28
7.26
7.25
7.17
7.14
4.02
4.00
3.97
3.95
2.36
1.22
1.19
1.17
Br
Me
CF_2CO_2Et
3c
2.50
1.97
1.04
3.00
3.05
f1 (ppm)

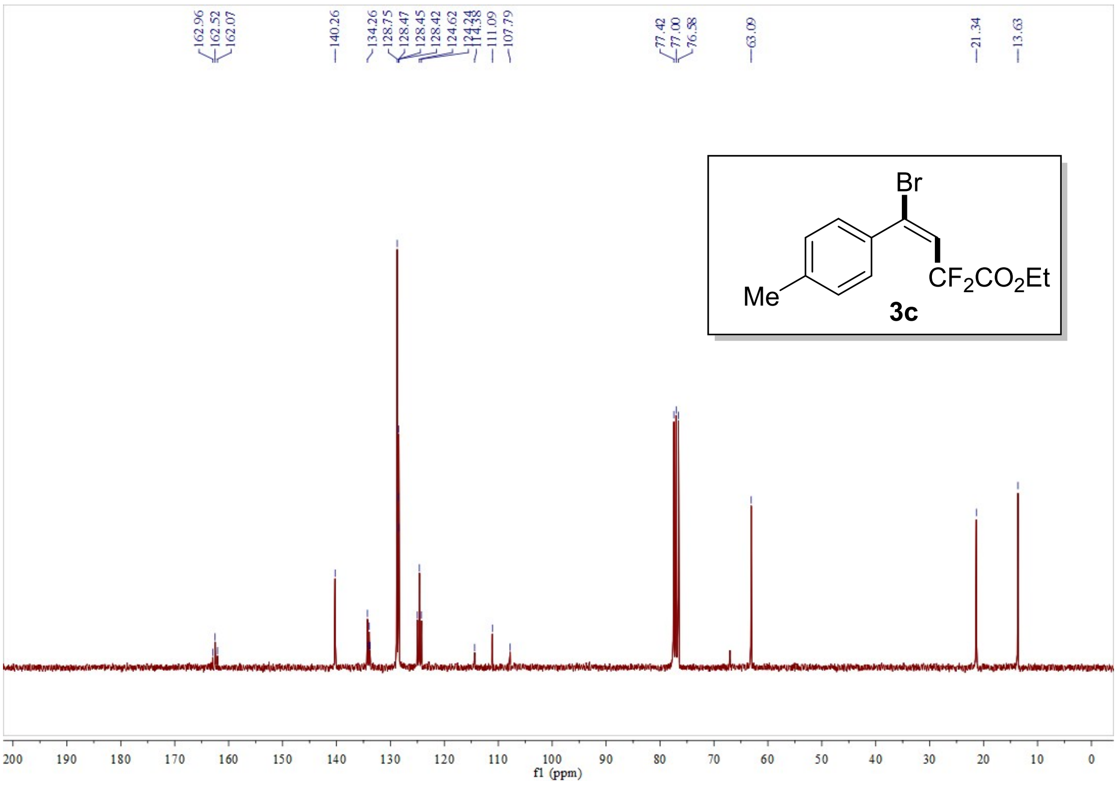
162.96
162.52
162.07
140.26
134.26
128.75
128.47
128.45
128.42
124.62
124.34
114.38
111.09
107.79
77.42
77.00
76.58
63.09
21.34
13.63
Br
Me
CF_2CO_2Et
3c
f1 (ppm)

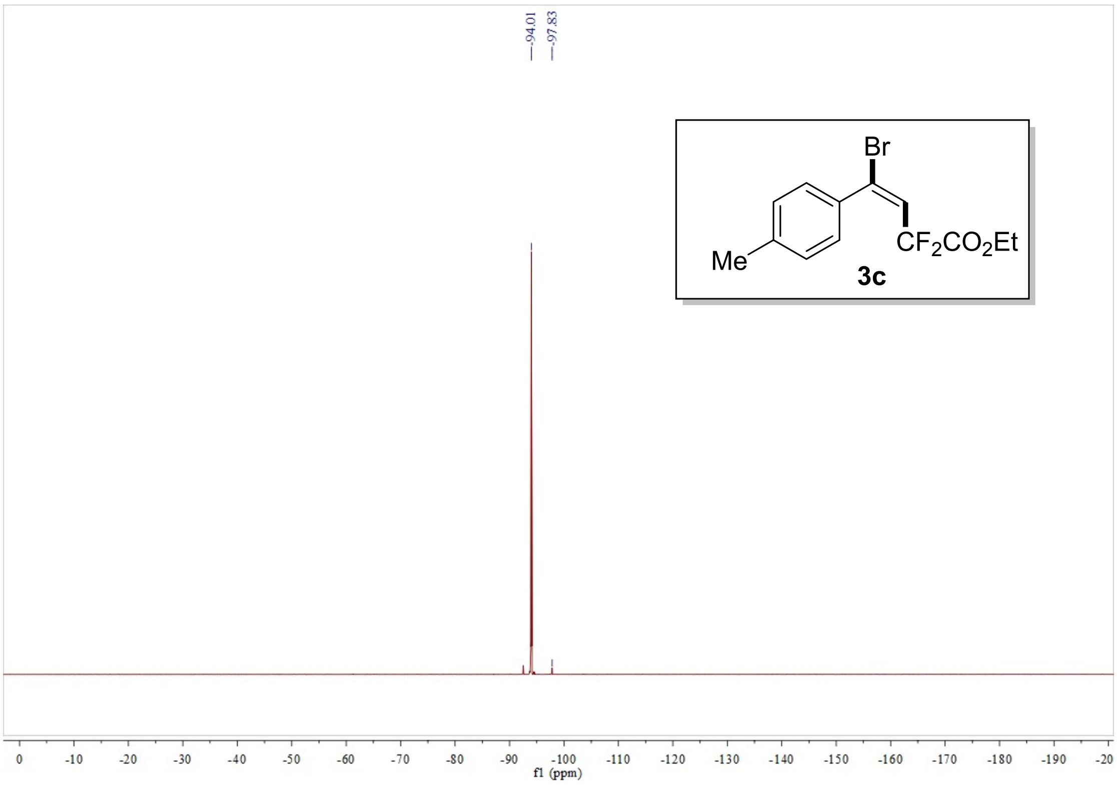

—94.01
—97.83
Br
Me
CF2CO2Et
3c
0 -10 -20 -30 -40 -50 -60 -70 -80 -90 -100 -110 -120 -130 -140 -150 -160 -170 -180 -190 -20
f1 (ppm)

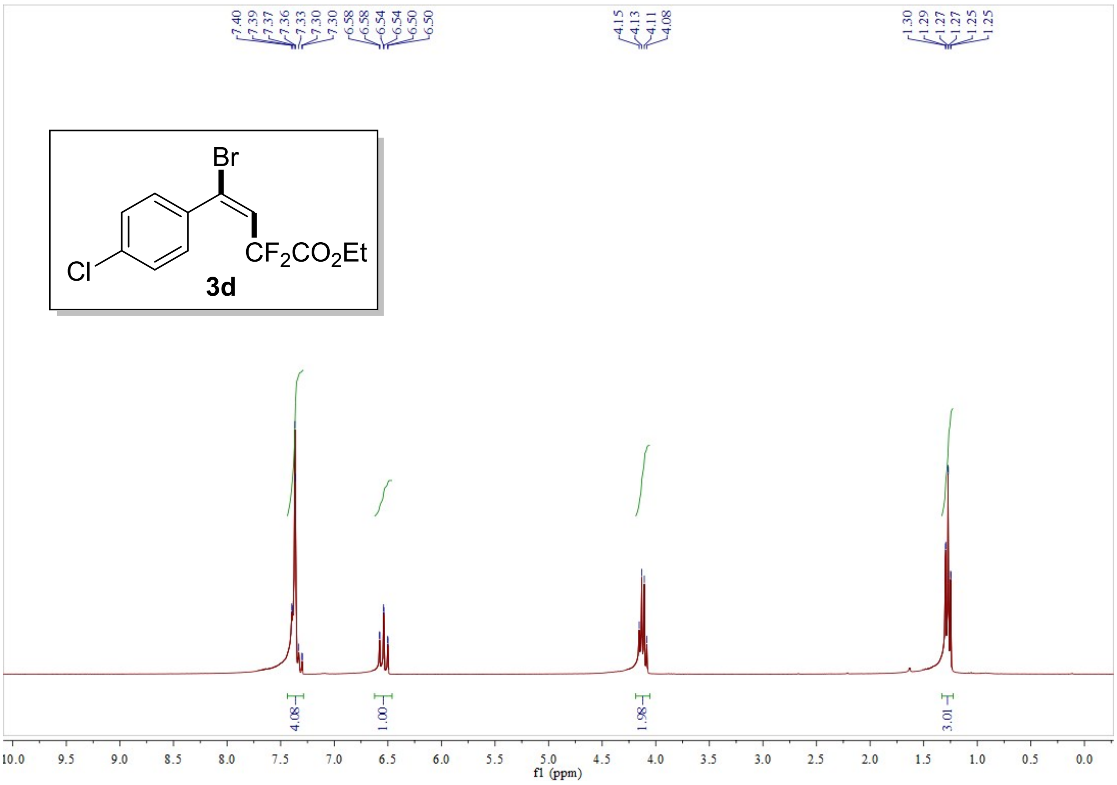

7.40 7.39 7.37 7.36 7.33 7.30 7.30 6.58 6.58 6.54 6.54 6.50 6.50
4.15 4.13 4.11 4.08
1.30 1.29 1.27 1.27 1.25 1.25
Br
Cl
CF2CO2Et
3d
4.08
1.00
1.98
3.01
10.0 9.5 9.0 8.5 8.0 7.5 7.0 6.5 6.0 5.5 5.0 4.5 4.0 3.5 3.0 2.5 2.0 1.5 1.0 0.5 0.0
f1 (ppm)

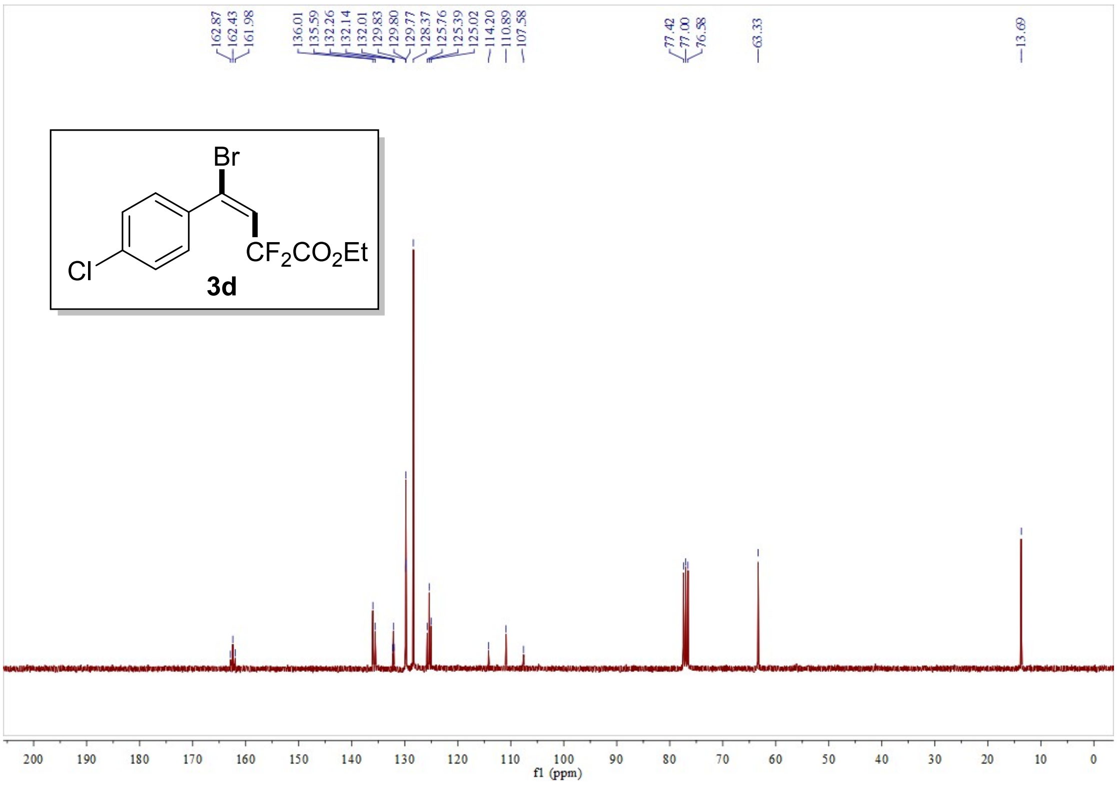
162.87
162.43
161.98
136.01
135.59
132.26
132.14
132.01
129.83
129.80
129.77
128.37
125.76
125.39
125.02
114.20
110.89
107.58
77.42
77.00
76.58
63.33
13.69
Br
Cl
CF2CO2Et
3d
f1 (ppm)

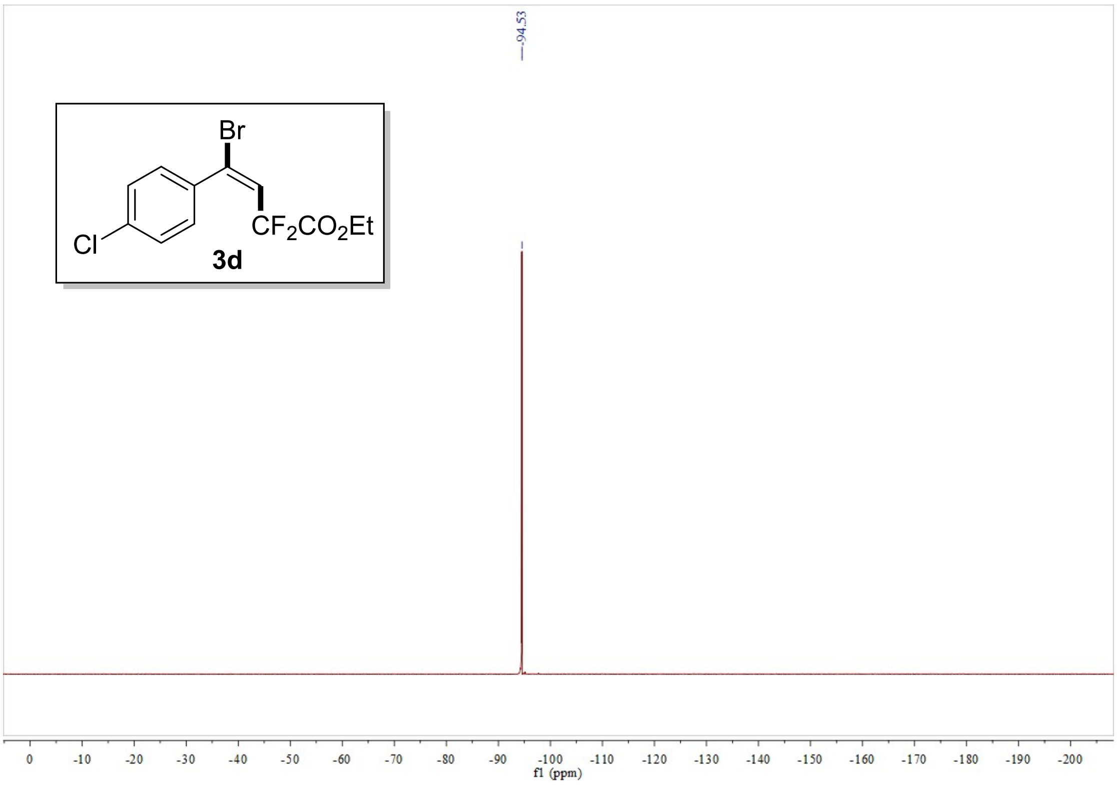
-94.53
Br
Cl
CF2CO2Et
3d
f1 (ppm)

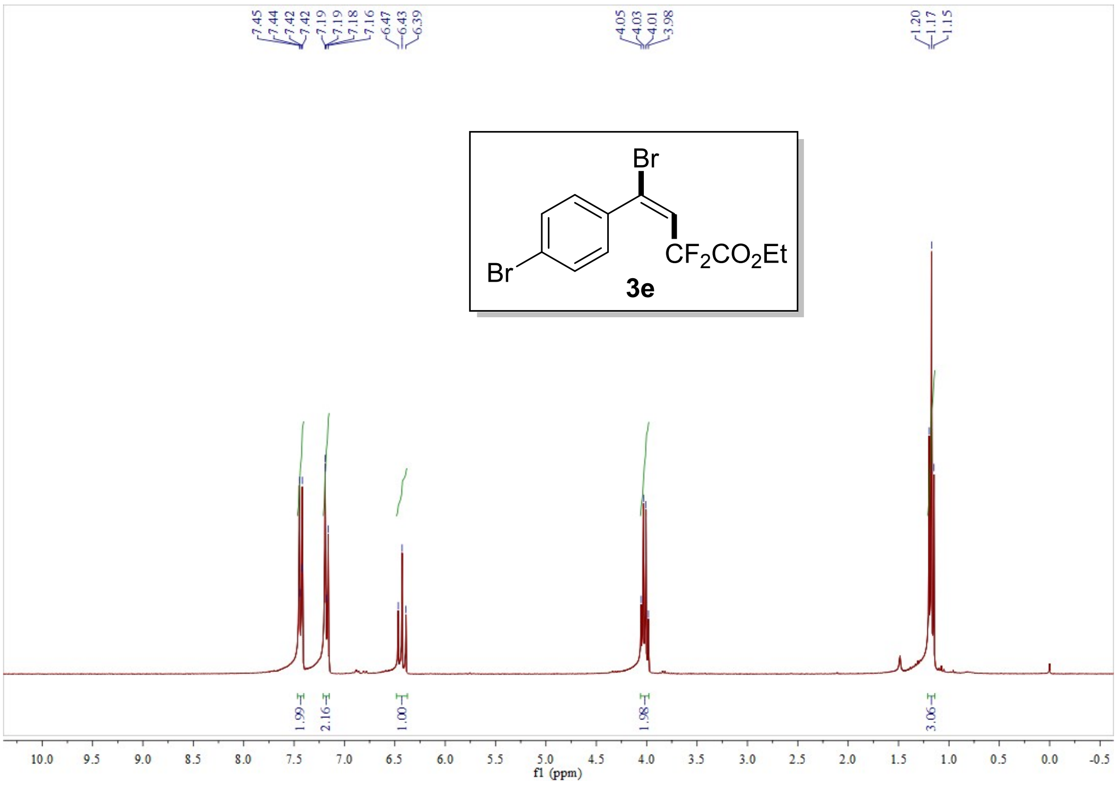
7.45
7.44
7.42
7.42
7.19
7.19
7.18
7.16
6.47
6.43
6.39
4.05
4.03
4.01
3.98
1.20
1.17
1.15
Br
Br
CF2CO2Et
3e
1.99
2.16
1.00
1.98
3.06
10.0
9.5
9.0
8.5
8.0
7.5
7.0
6.5
6.0
5.5
5.0
4.5
4.0
3.5
3.0
2.5
2.0
1.5
1.0
0.5
0.0
-0.5
f1 (ppm)

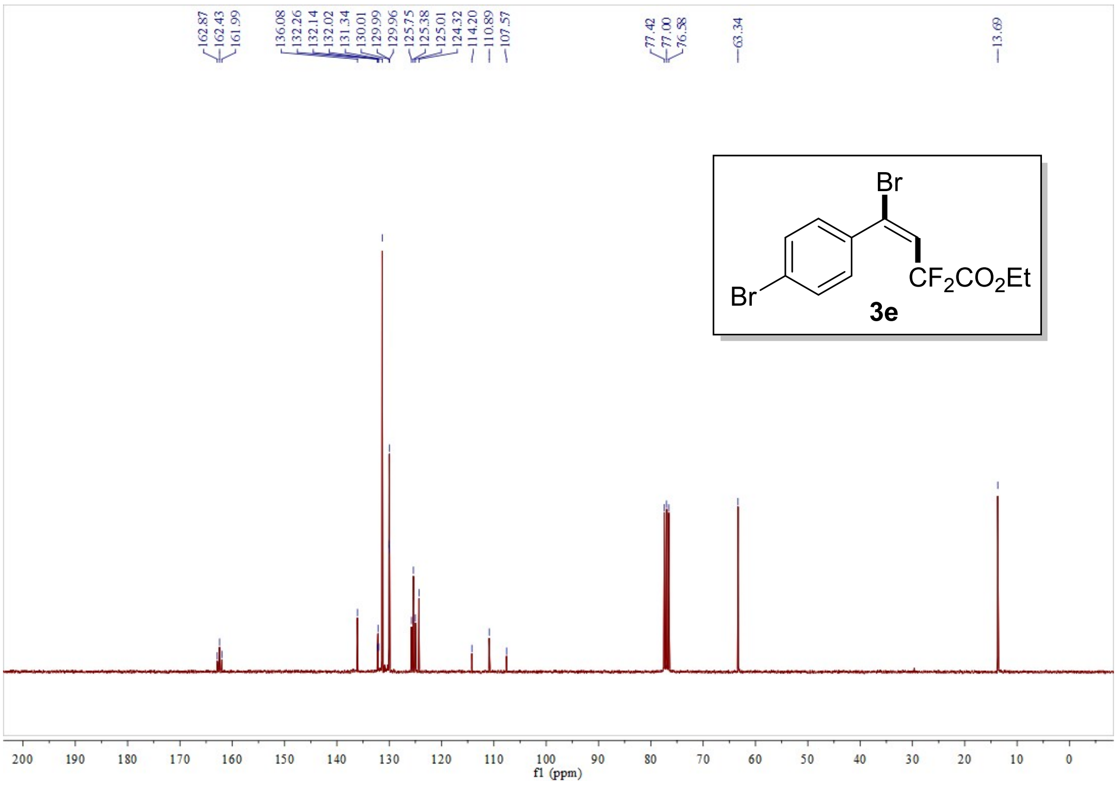
162.87
162.43
161.99
136.08
132.26
132.14
132.02
131.34
130.01
129.99
129.96
125.75
125.38
125.01
124.32
114.20
110.89
107.57
77.42
77.00
76.58
63.34
13.69
Br
Br
CF2CO2Et
3e
200
190
180
170
160
150
140
130
120
110
100
90
80
70
60
50
40
30
20
10
0
f1 (ppm)

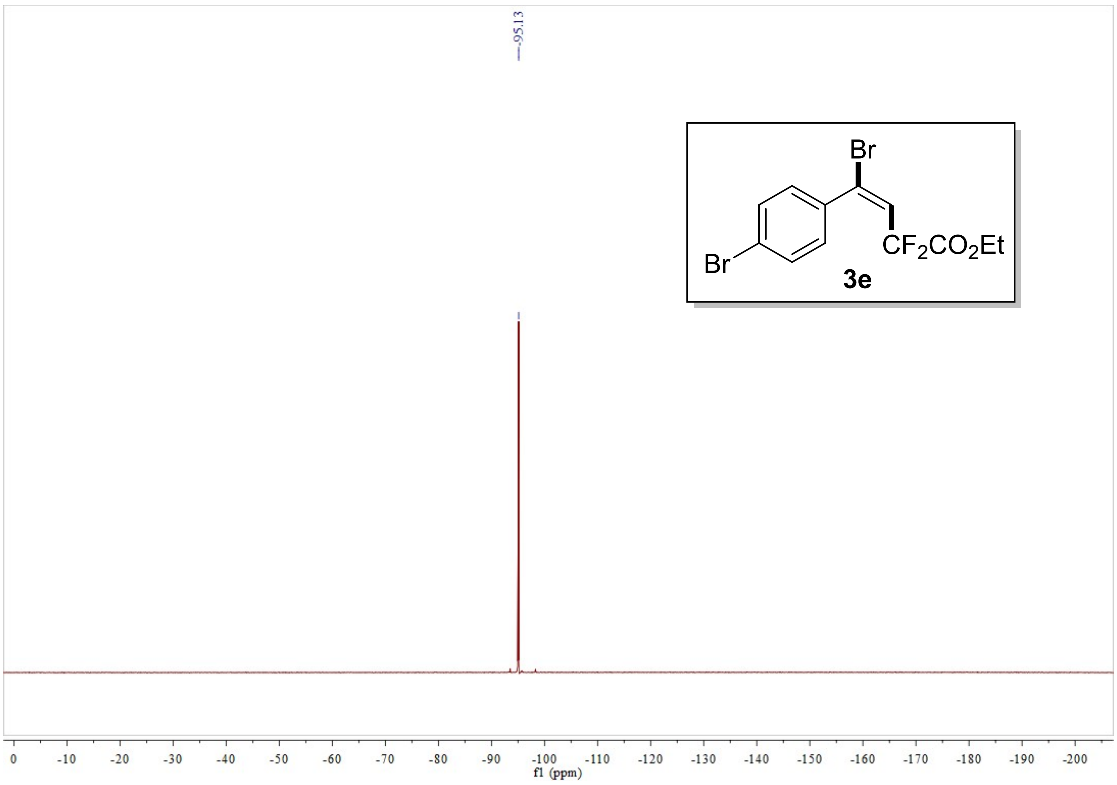

-95.13
Br
Br
CF2CO2Et
3e
0
-10
-20
-30
-40
-50
-60
-70
-80
-90
-100
-110
-120
-130
-140
-150
-160
-170
-180
-190
-200
f1 (ppm)

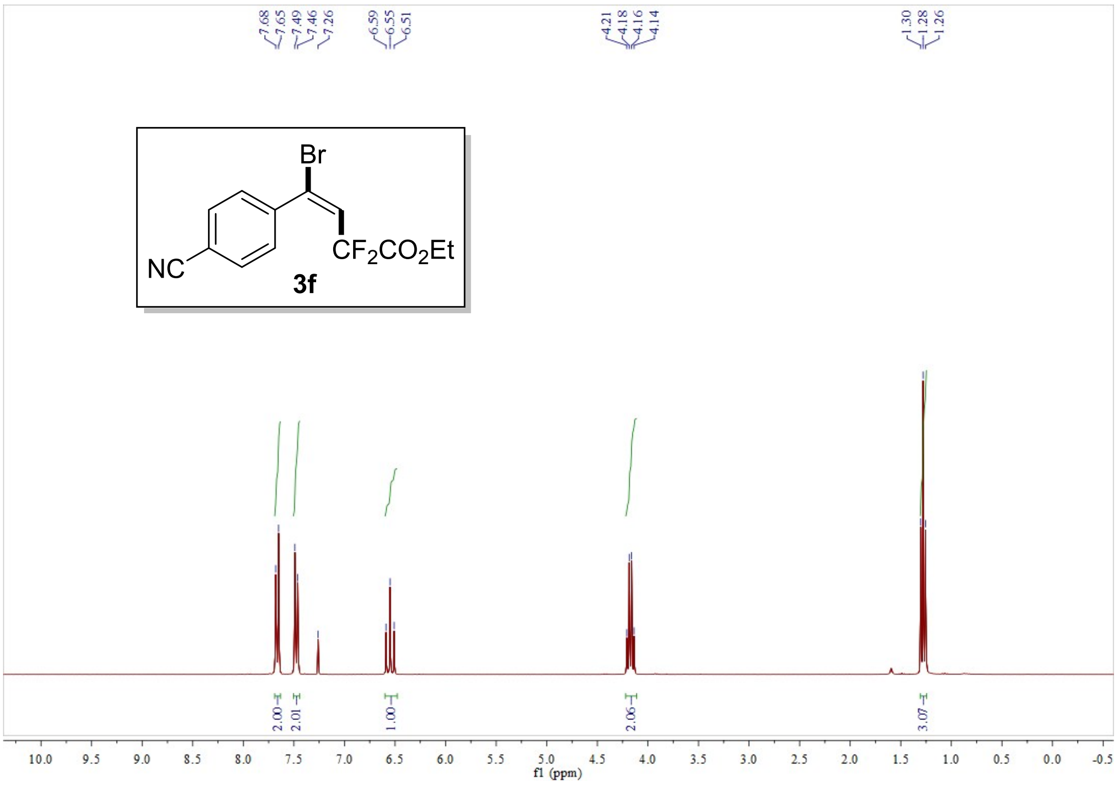

7.68
7.65
7.49
7.46
7.26
6.59
6.55
6.51
4.21
4.18
4.16
4.14
1.30
1.28
1.26
Br
NC
CF2CO2Et
3f
2.00
2.01
1.00
2.06
3.07
10.0
9.5
9.0
8.5
8.0
7.5
7.0
6.5
6.0
5.5
5.0
4.5
4.0
3.5
3.0
2.5
2.0
1.5
1.0
0.5
0.0
-0.5
f1 (ppm)

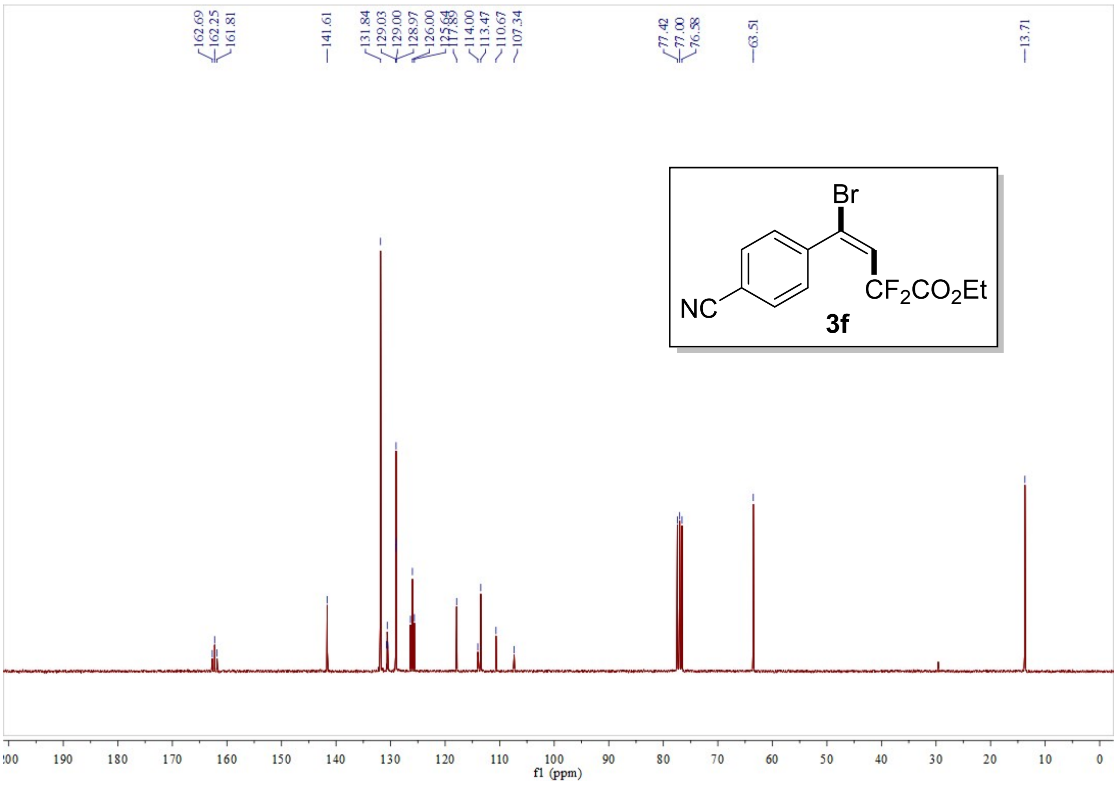
162.69
162.25
161.81
141.61
131.84
129.03
129.00
128.97
126.00
114.00
113.47
110.67
107.34
77.42
77.00
76.58
63.51
13.71
Br
NC
CF2CO2Et
3f
200
190
180
170
160
150
140
130
120
110
100
90
80
70
60
50
40
30
20
10
0
f1 (ppm)

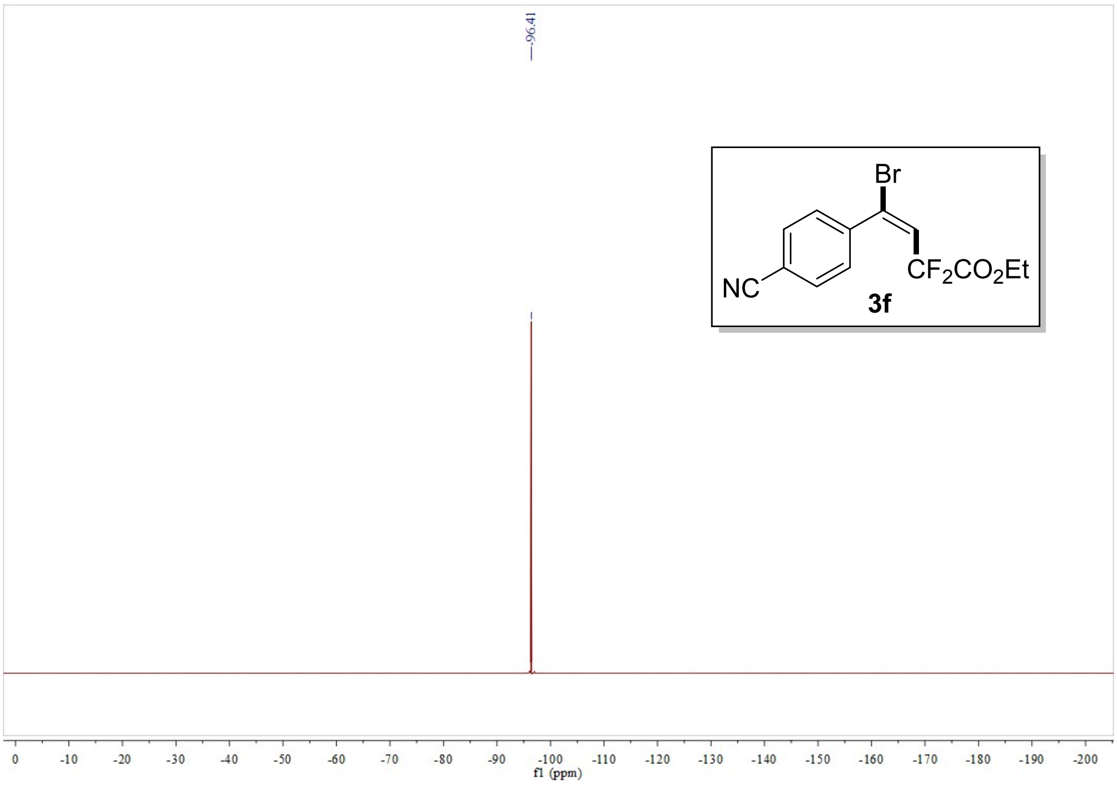
-96.41
Br
NC
CF2CO2Et
3f
0
-10
-20
-30
-40
-50
-60
-70
-80
-90
-100
-110
-120
-130
-140
-150
-160
-170
-180
-190
-200
f1 (ppm)

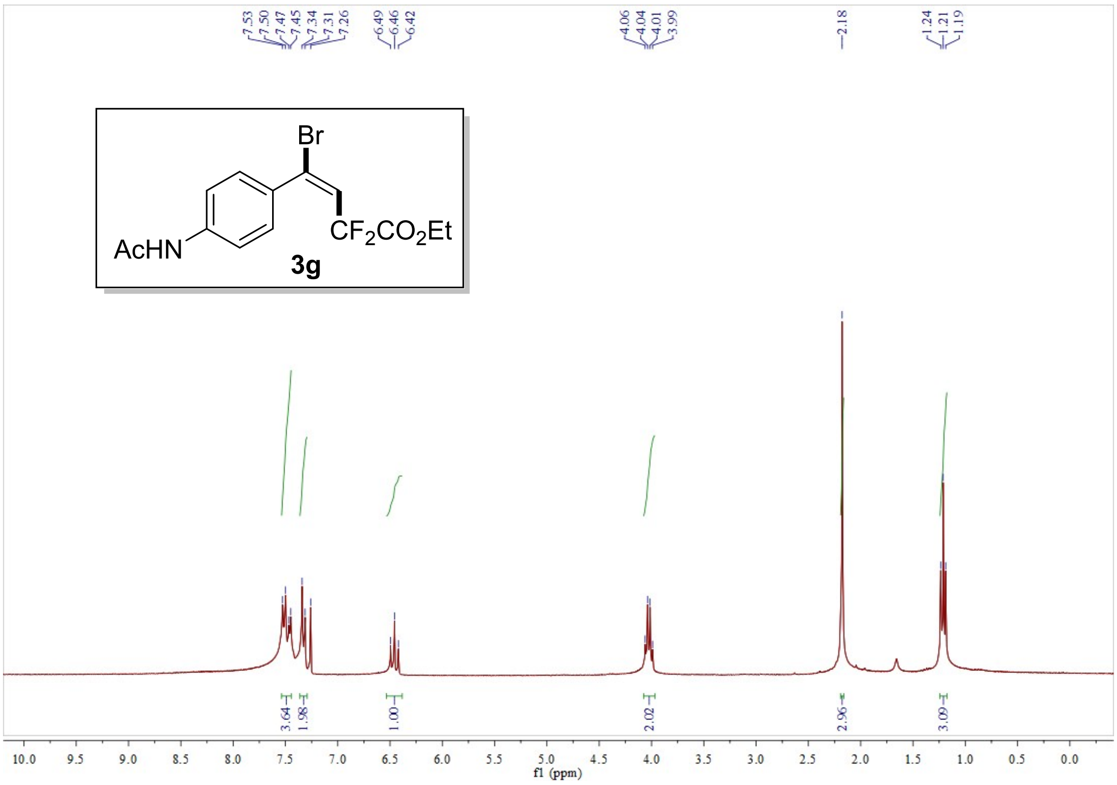

7.53
7.50
7.47
7.45
7.34
7.31
7.26
6.49
6.46
6.42
4.06
4.04
4.01
3.99
2.18
1.24
1.21
1.19
Br
AcHN
CF2CO2Et
3g
3.64
1.98
1.00
2.02
2.96
3.09
f1 (ppm)

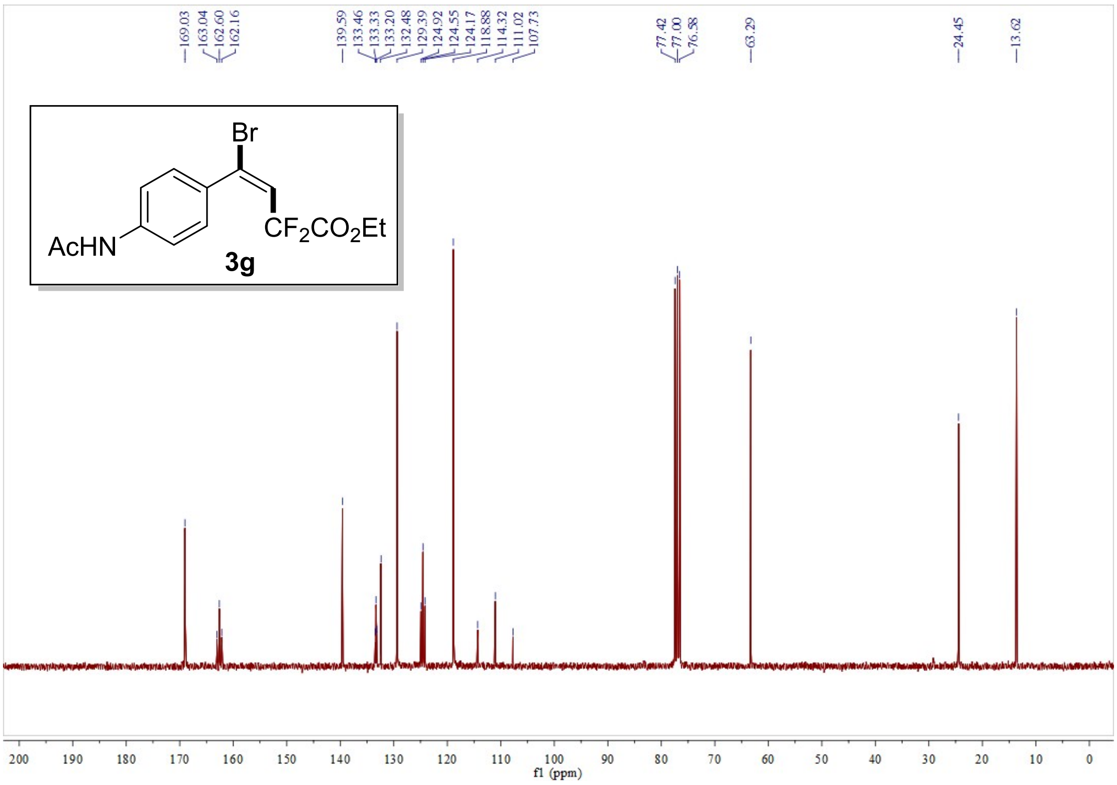

169.03
163.04
162.60
162.16
139.59
133.46
133.33
133.20
132.48
129.39
124.92
124.55
124.17
118.88
114.32
111.02
107.73
77.42
77.00
76.58
63.29
24.45
13.62
Br
AcHN
CF2CO2Et
3g
f1 (ppm)

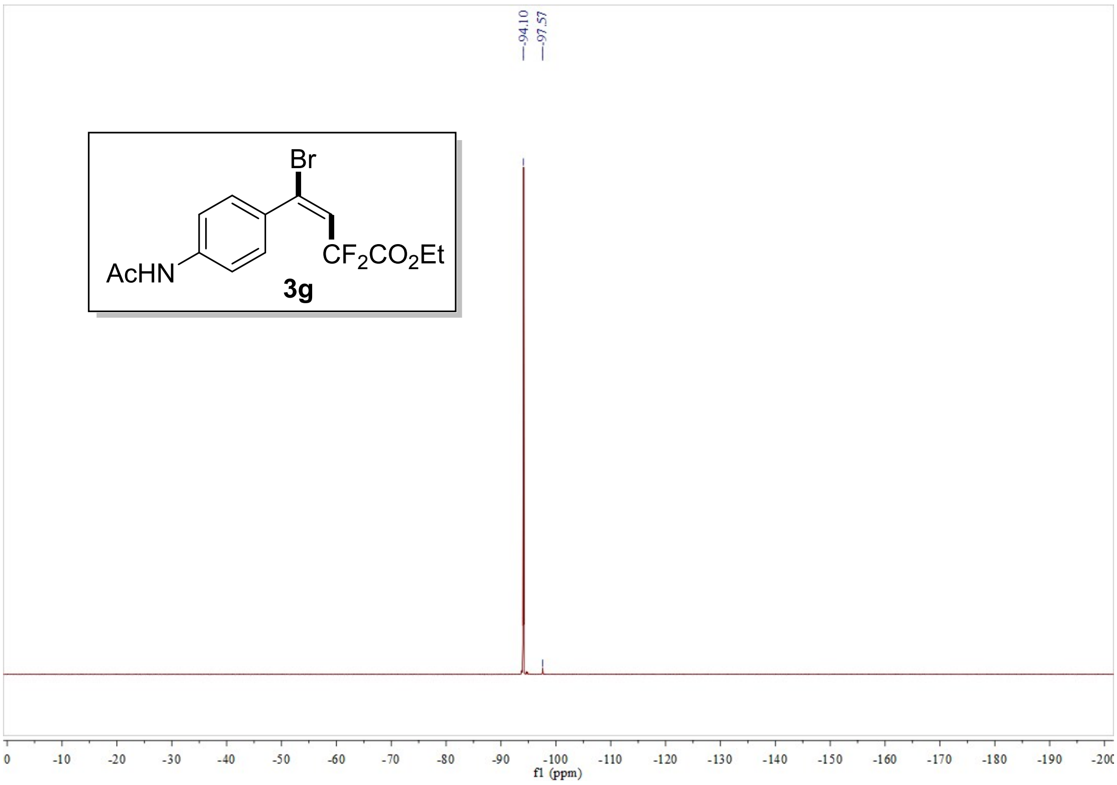

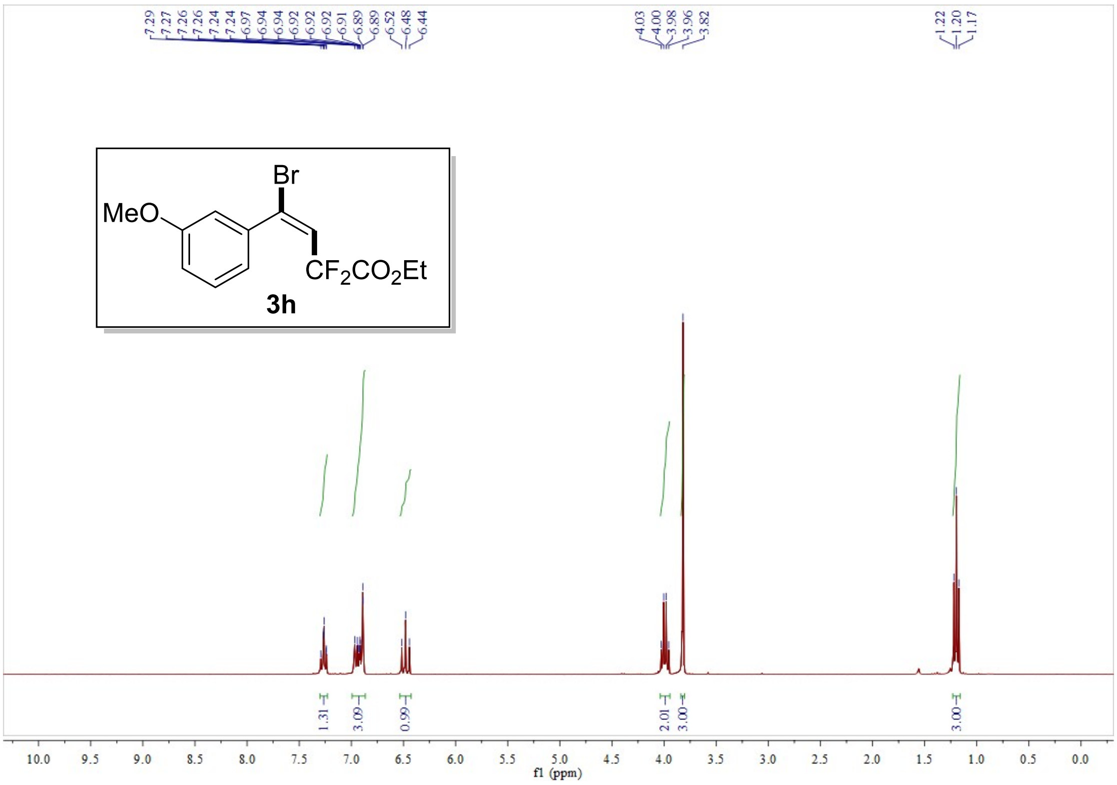

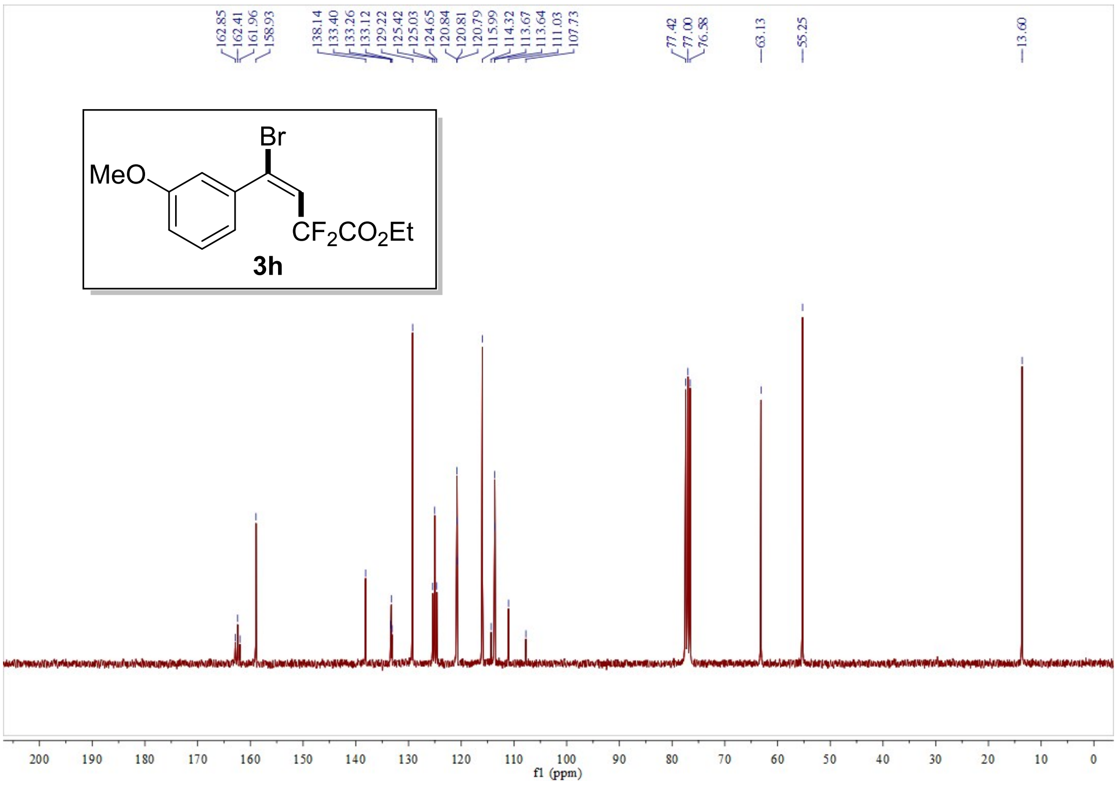
162.85
162.41
161.96
158.93
138.14
133.40
133.26
133.12
129.22
125.42
125.03
124.65
120.84
120.81
120.79
115.99
114.32
113.67
113.64
111.03
107.73
77.42
77.00
76.58
63.13
55.25
13.60
Br
MeO
CF2CO2Et
3h
200 190 180 170 160 150 140 130 120 110 100 90 80 70 60 50 40 30 20 10 0
f1 (ppm)

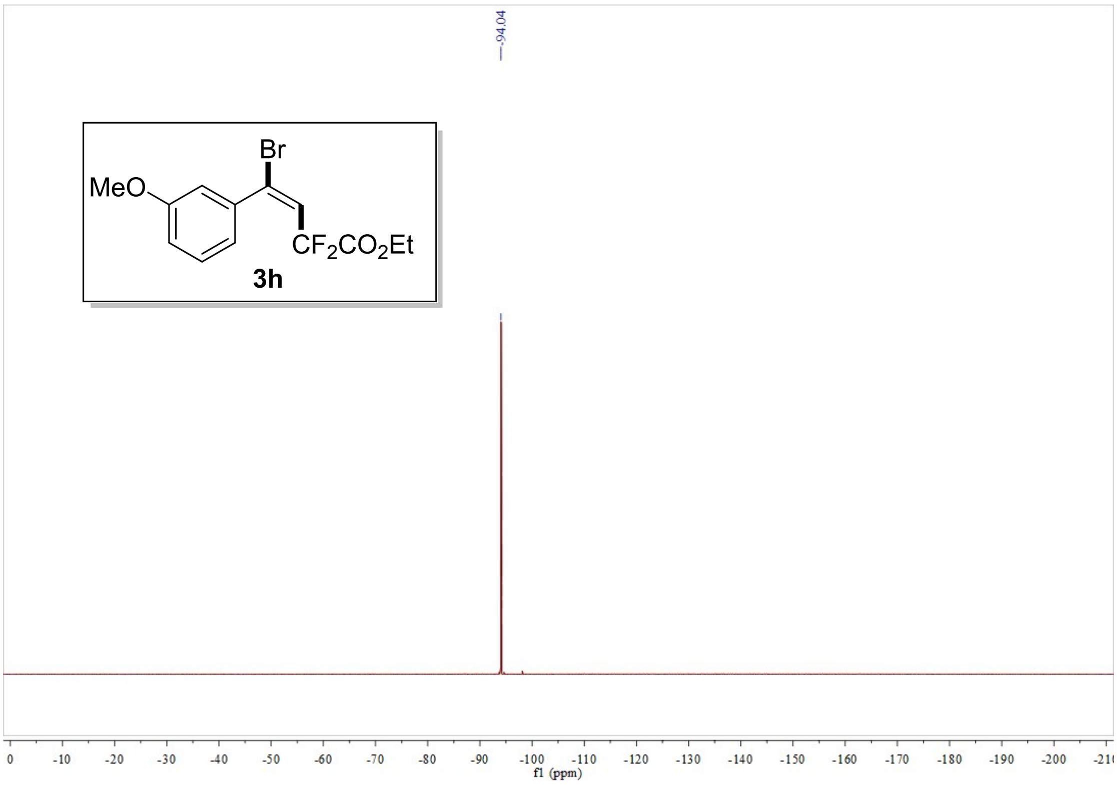
-94.04
Br
MeO
CF2CO2Et
3h
0 -10 -20 -30 -40 -50 -60 -70 -80 -90 -100 -110 -120 -130 -140 -150 -160 -170 -180 -190 -200
f1 (ppm)

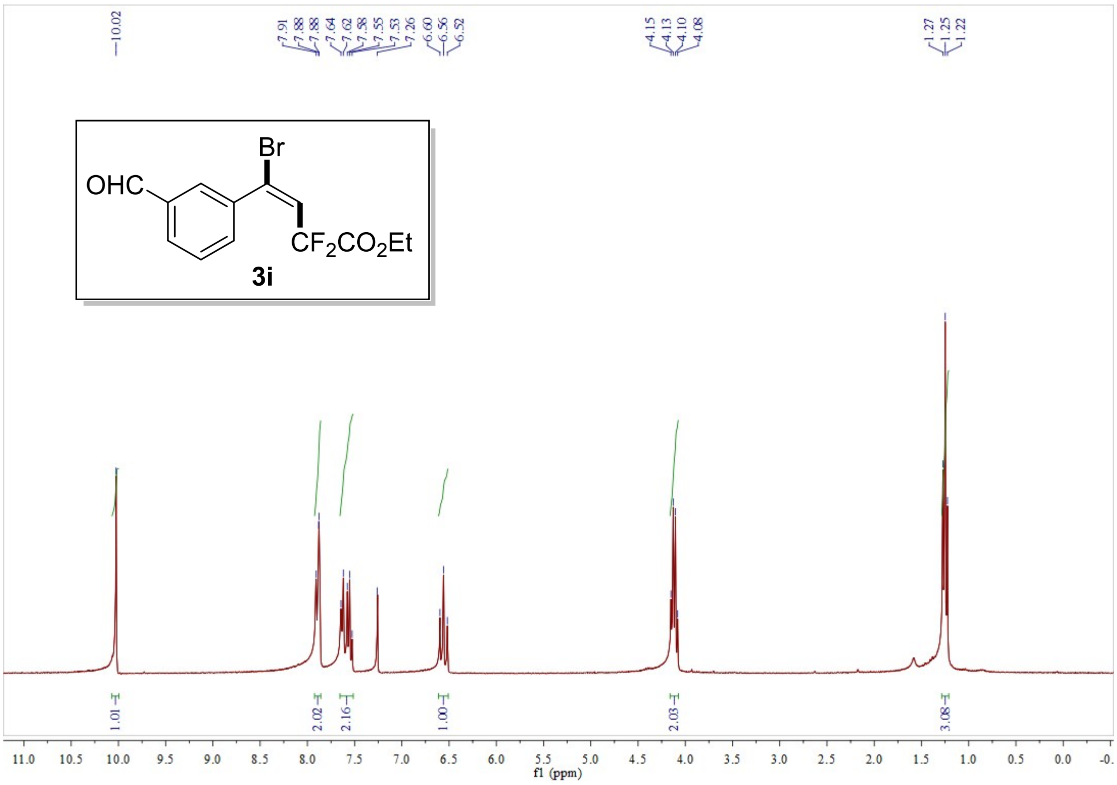
Br
OHC
CF2CO2Et
3i
f1 (ppm)

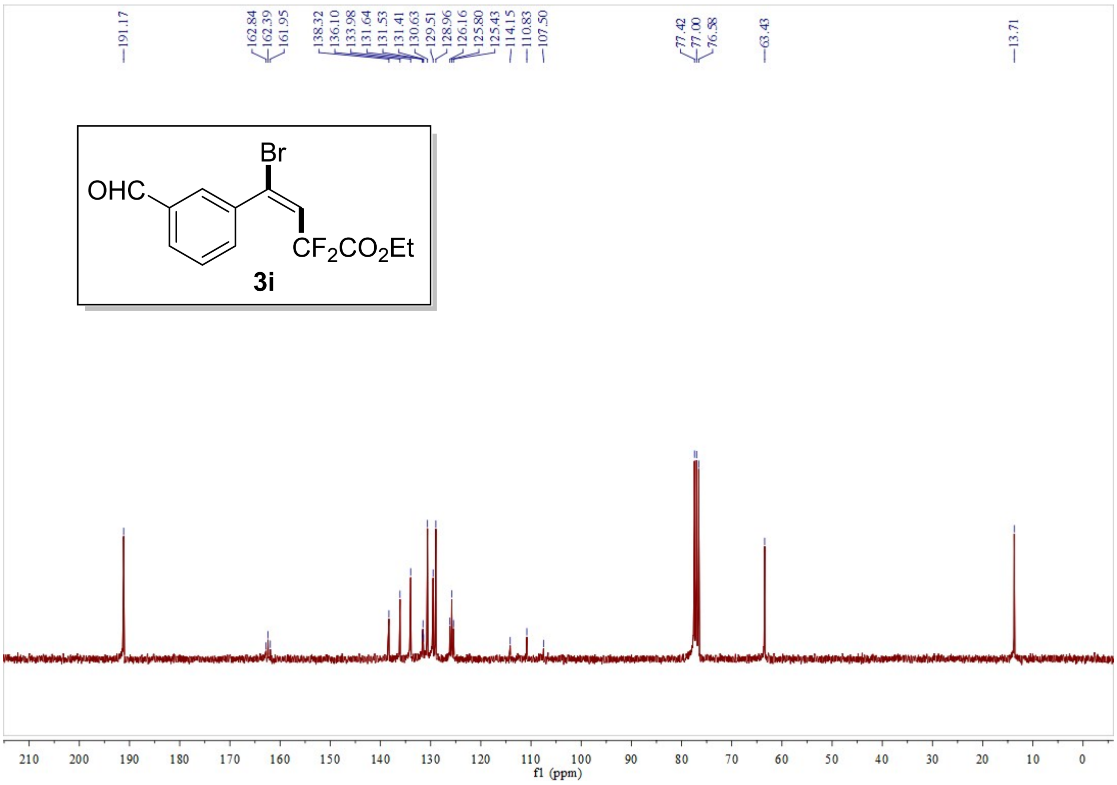
Br
OHC
CF2CO2Et
3i
f1 (ppm)

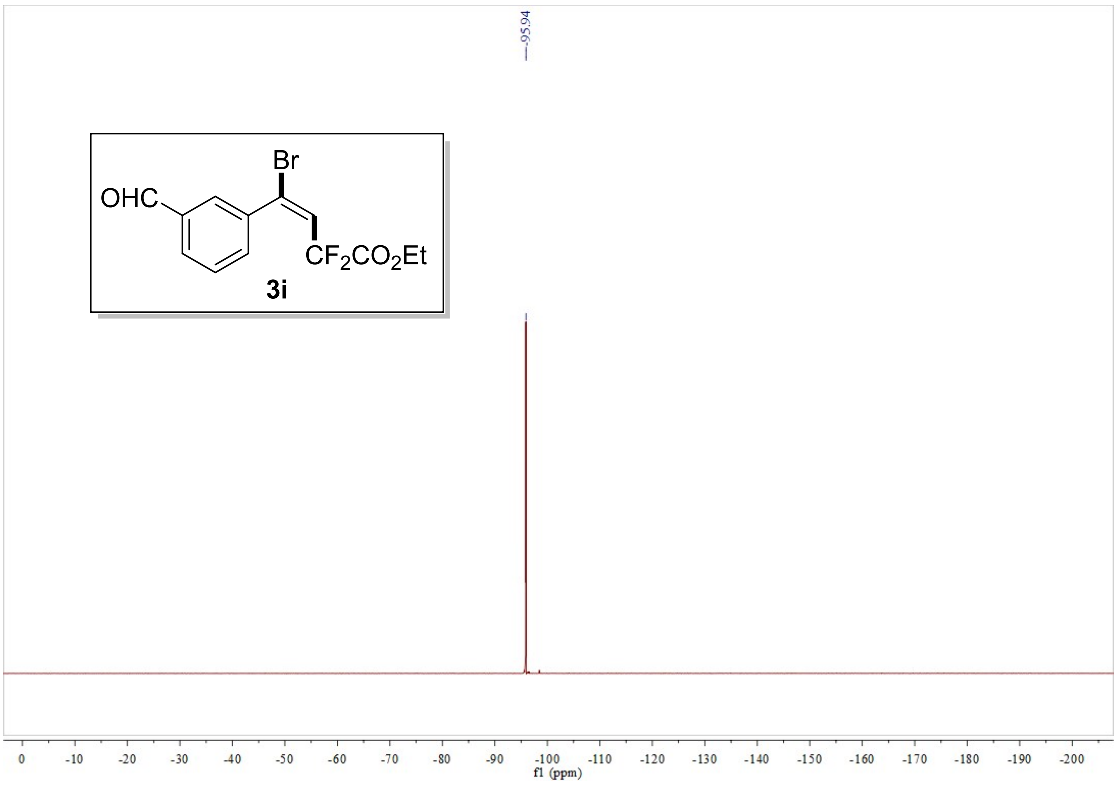
-95.94
OHC
Br
CF_2CO_2Et
3i
f1 (ppm)

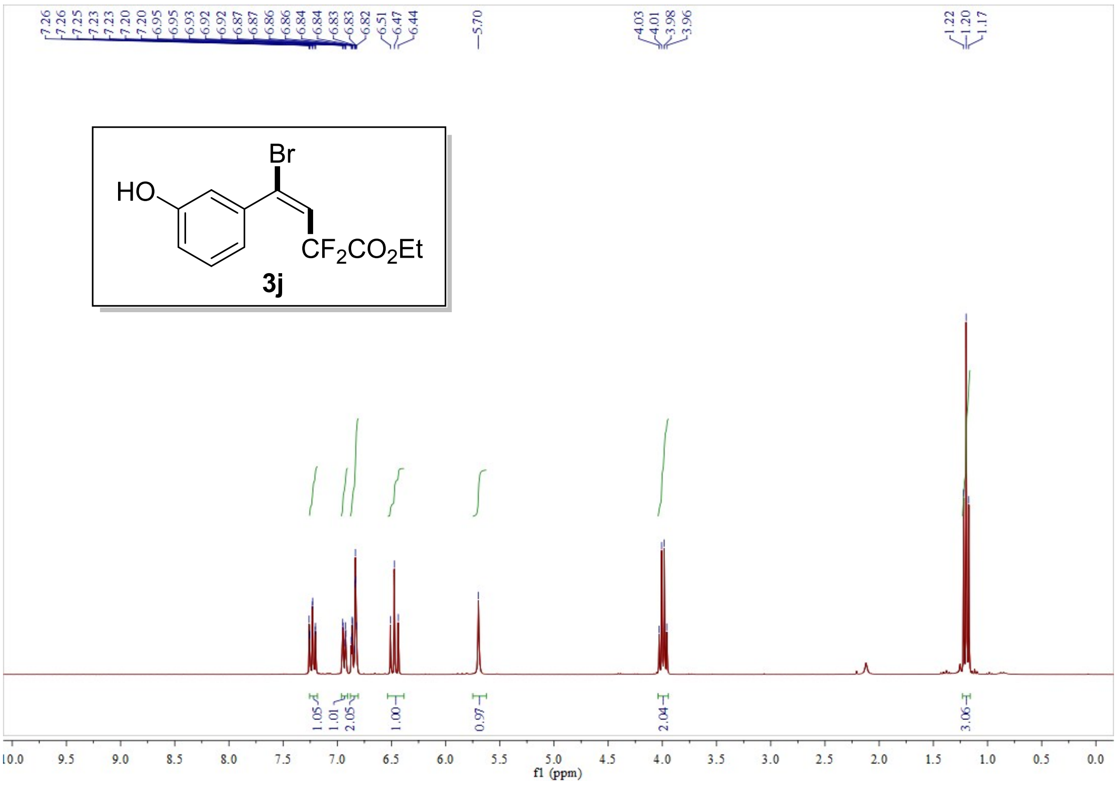
7.26
7.26
7.25
7.23
7.23
7.20
7.20
6.95
6.95
6.93
6.92
6.92
6.87
6.87
6.86
6.86
6.84
6.84
6.83
6.83
6.82
6.51
6.47
6.44
5.70
4.03
4.01
3.98
3.96
1.22
1.20
1.17
HO
Br
CF_2CO_2Et
3j
1.05
1.01
2.05
1.00
0.97
2.04
3.06
f1 (ppm)

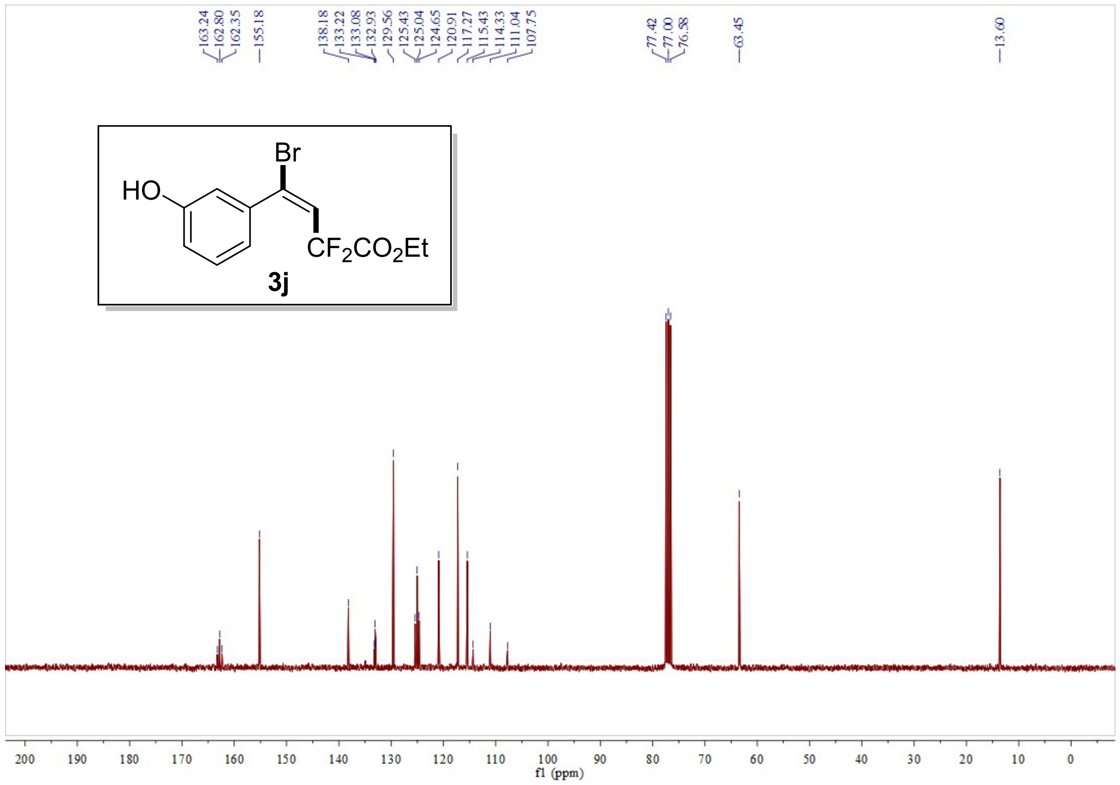
163.24
162.80
162.35
155.18
138.18
133.22
133.08
132.93
129.56
125.43
125.04
124.65
120.91
117.27
115.43
114.33
111.04
107.75
77.42
77.00
76.58
63.45
13.60
HO
Br
CF2CO2Et
3j
f1 (ppm)

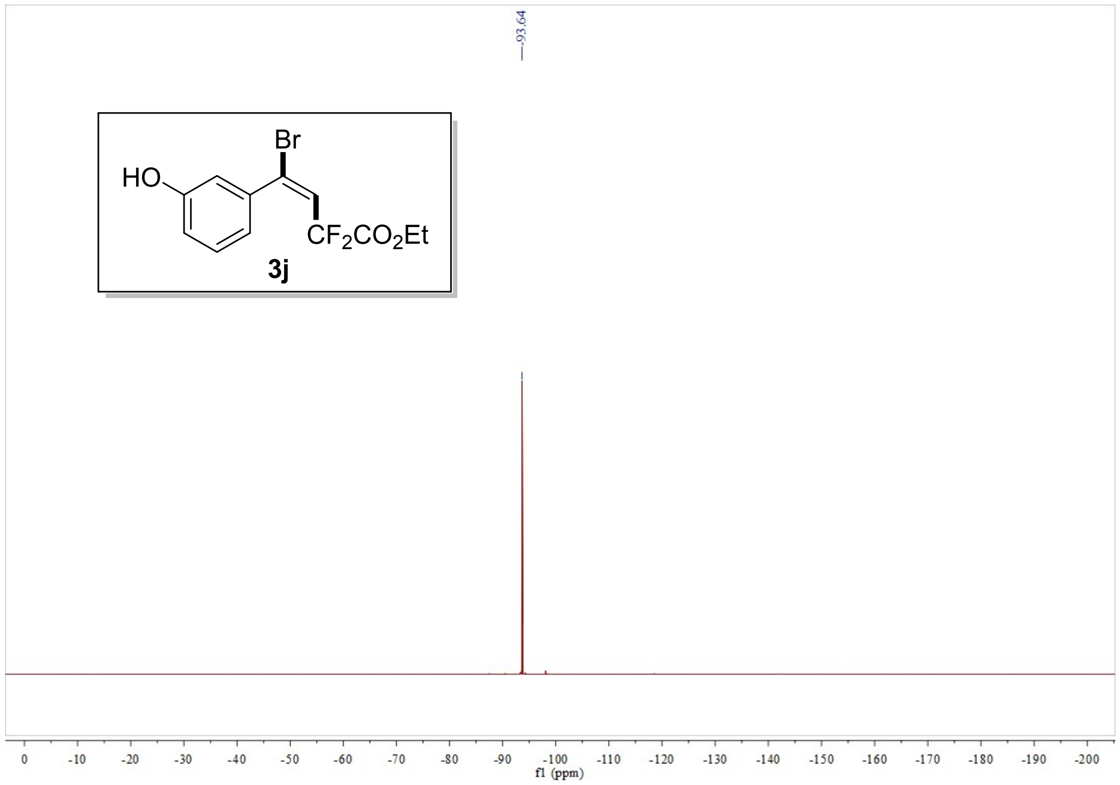
-93.64
HO
Br
CF2CO2Et
3j
f1 (ppm)

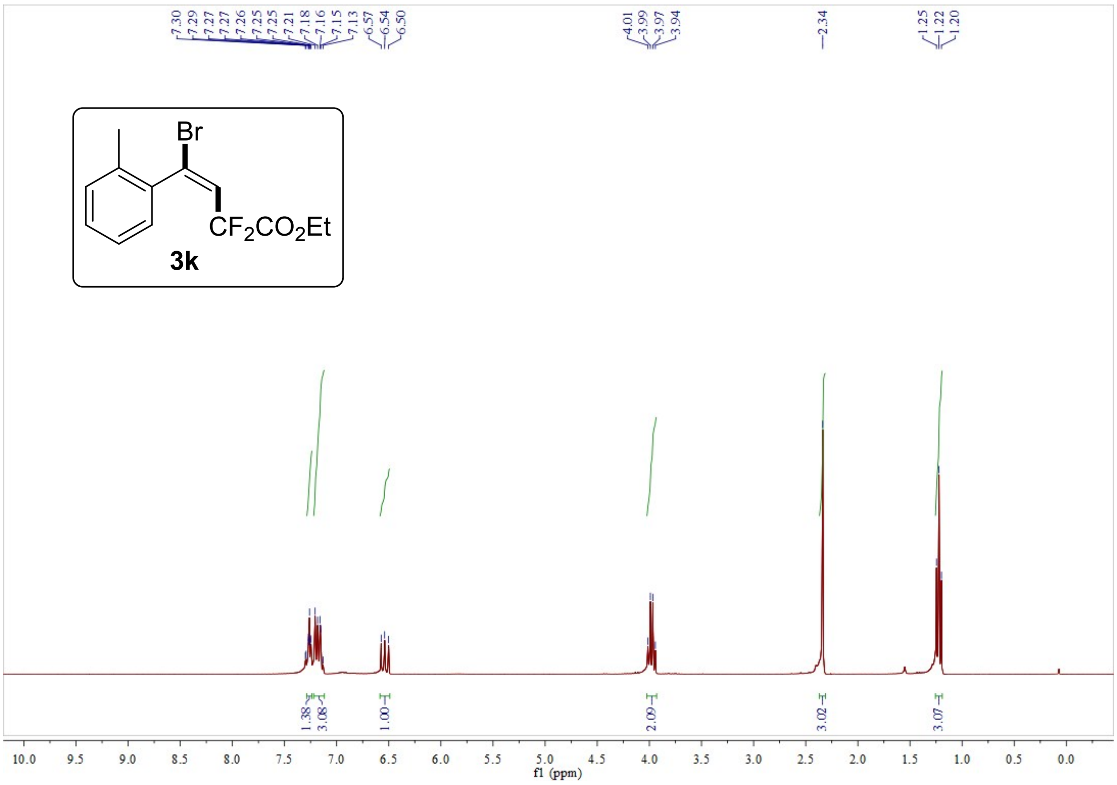
7.30
7.29
7.27
7.27
7.26
7.25
7.25
7.21
7.18
7.16
7.15
7.13
6.57
6.54
6.50
4.01
3.99
3.97
3.94
2.34
1.25
1.22
1.20
Br
CF2CO2Et
3k
1.38
3.08
1.00
2.09
3.02
3.07
10.0 9.5 9.0 8.5 8.0 7.5 7.0 6.5 6.0 5.5 5.0 4.5 4.0 3.5 3.0 2.5 2.0 1.5 1.0 0.5 0.0
f1 (ppm)

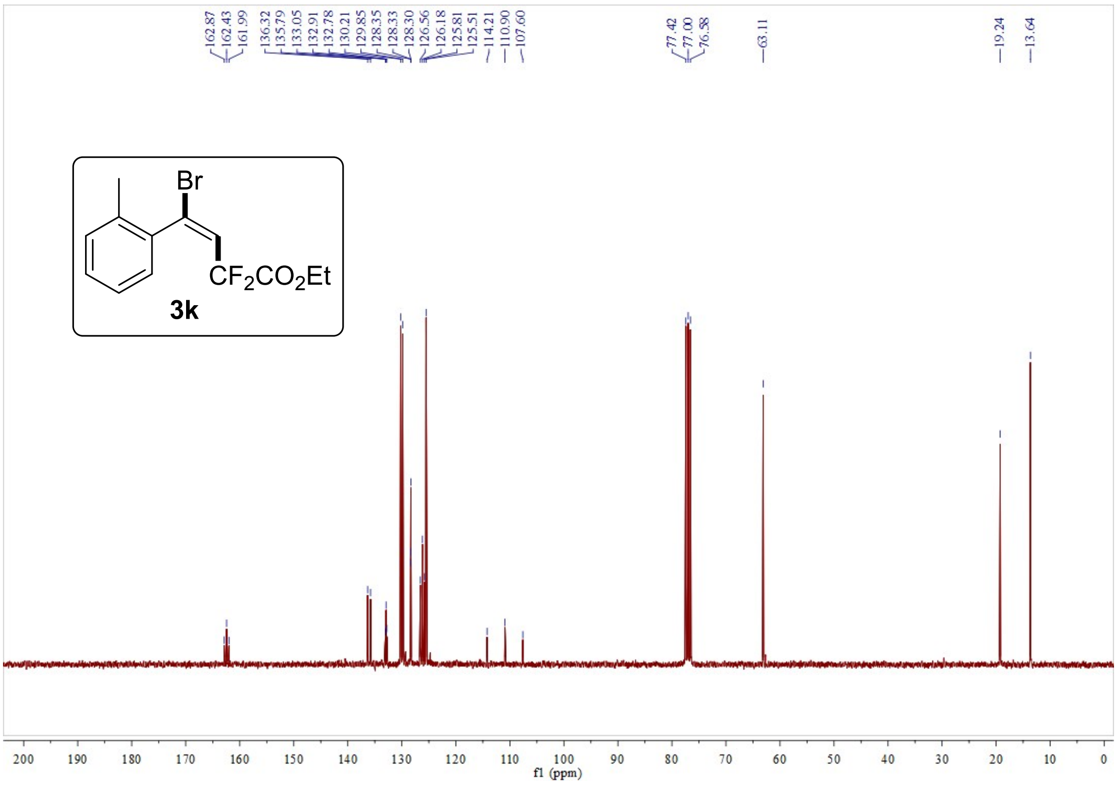
162.87
162.43
161.99
136.32
135.79
133.05
132.91
132.78
130.21
129.85
128.35
128.33
128.30
126.56
126.18
125.81
125.51
114.21
110.90
107.60
77.42
77.00
76.58
63.11
19.24
13.64
Br
CF2CO2Et
3k
200 190 180 170 160 150 140 130 120 110 100 90 80 70 60 50 40 30 20 10 0
f1 (ppm)

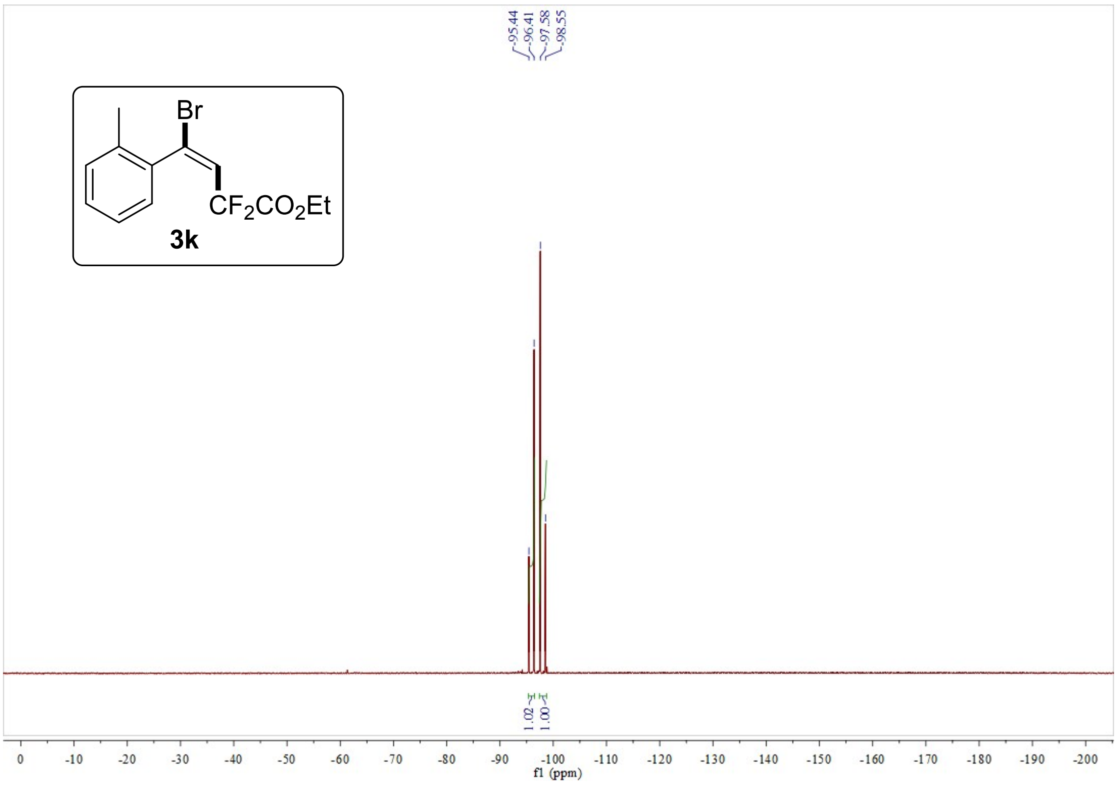
-95.44
-96.41
-97.58
-98.55
Br
CF2CO2Et
3k
1.02
1.00
f1 (ppm)

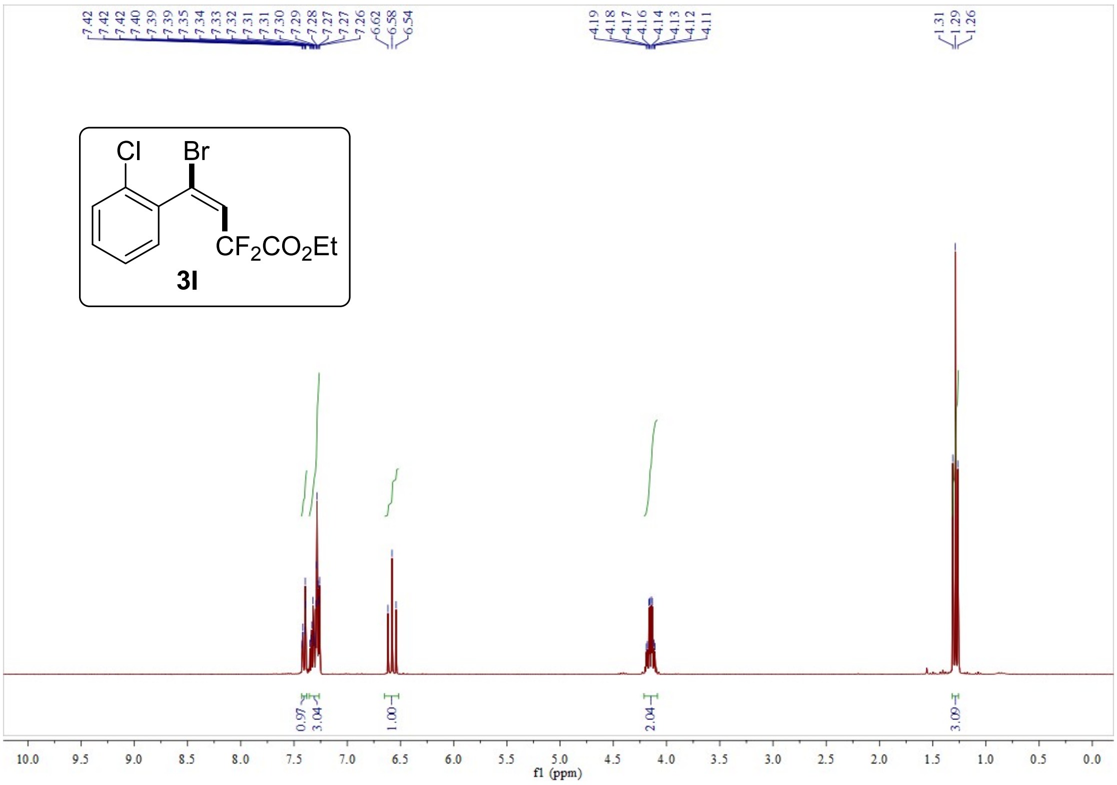
Cl
Br
CF2CO2Et
3l
0.97
3.04
1.00
2.04
3.09
f1 (ppm)

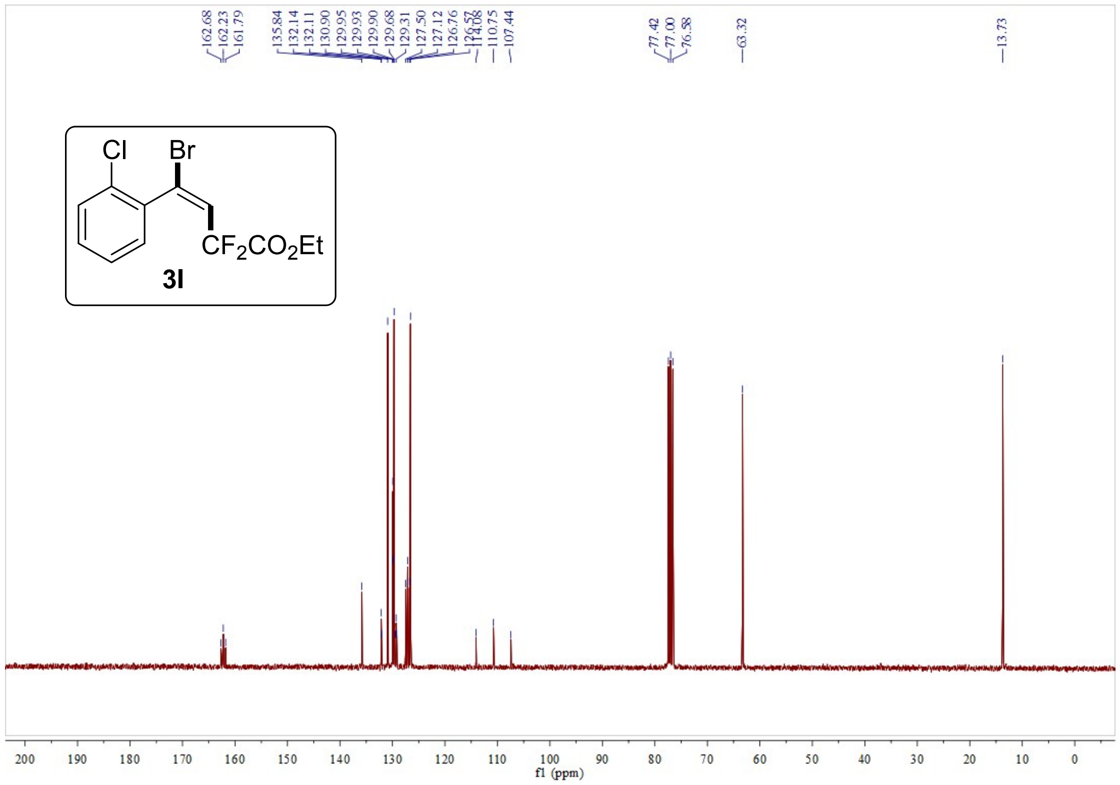
162.68
162.23
161.79
135.84
132.14
132.11
130.90
129.95
129.93
129.90
129.68
129.31
127.50
127.12
126.76
126.57
114.08
110.75
107.44
77.42
77.00
76.58
63.32
13.73
Cl
Br
CF2CO2Et
3l
f1 (ppm)

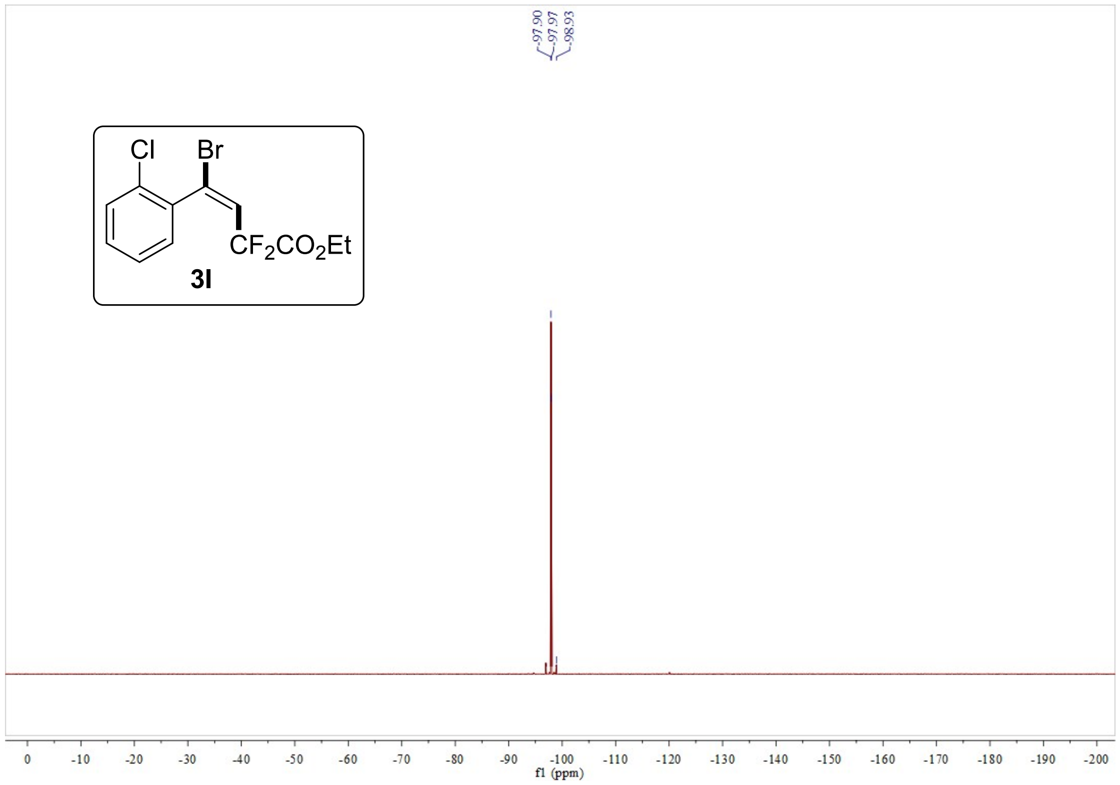
-97.90
-97.97
-98.93
Cl
Br
CF2CO2Et
3l
f1 (ppm)

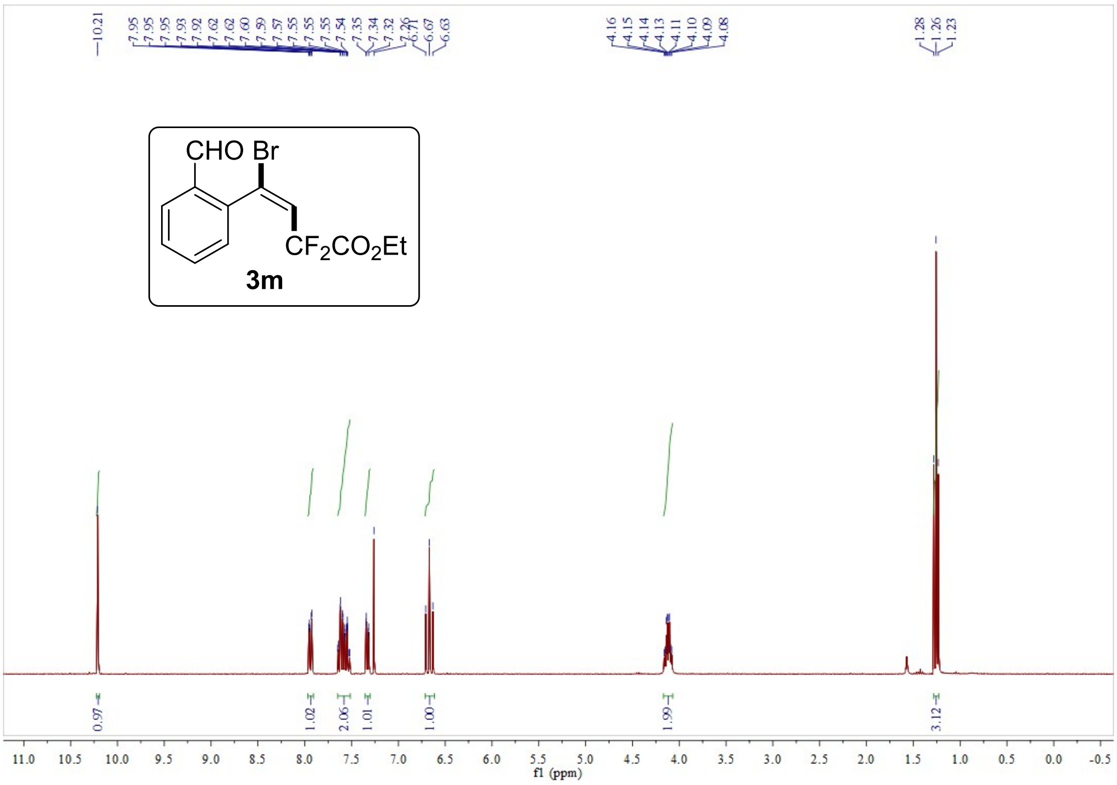
CHO Br
CF2CO2Et
3m
f1 (ppm)

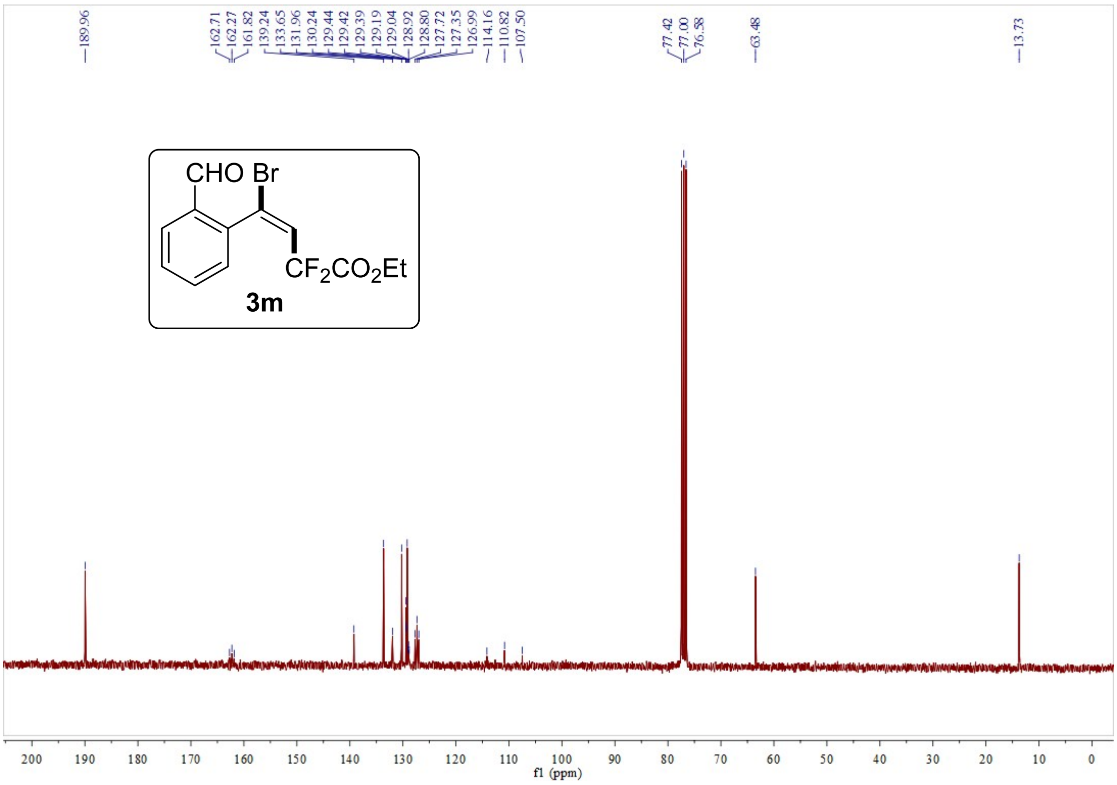
CHO Br
CF2CO2Et
3m
f1 (ppm)

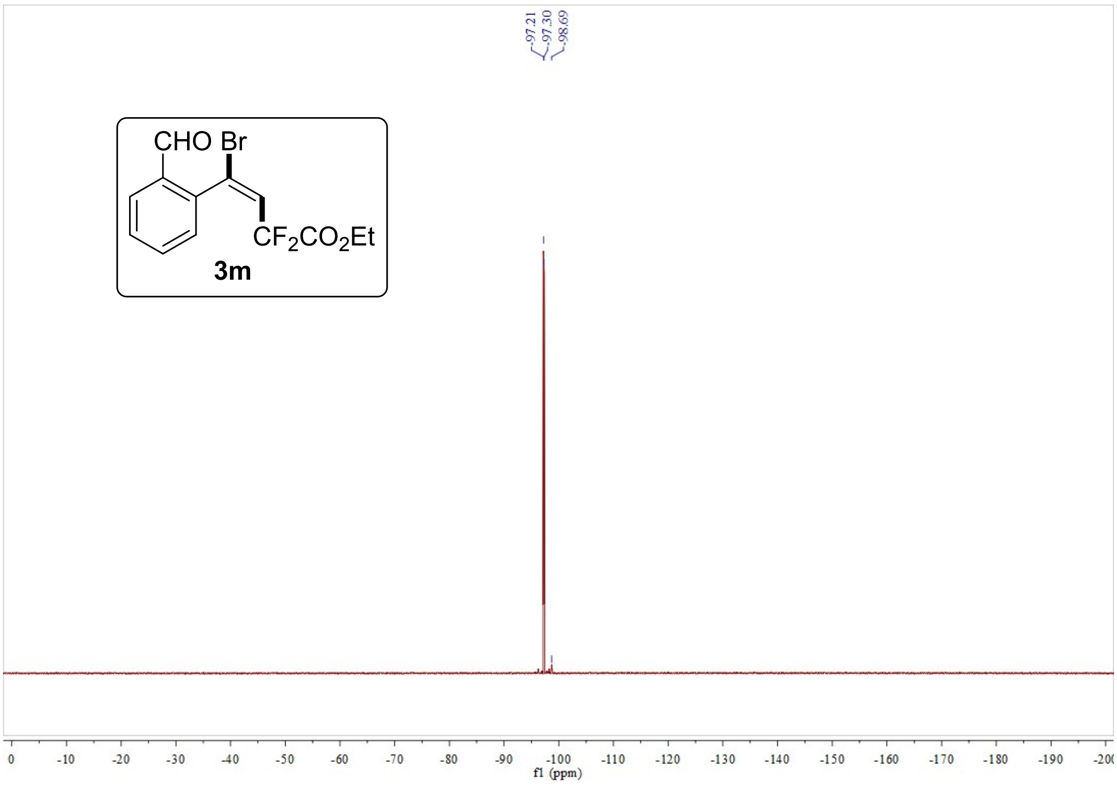

-97.21
-97.30
-98.69
CHO Br
CF2CO2Et
3m
0 -10 -20 -30 -40 -50 -60 -70 -80 -90 -100 -110 -120 -130 -140 -150 -160 -170 -180 -190 -200
f1 (ppm)

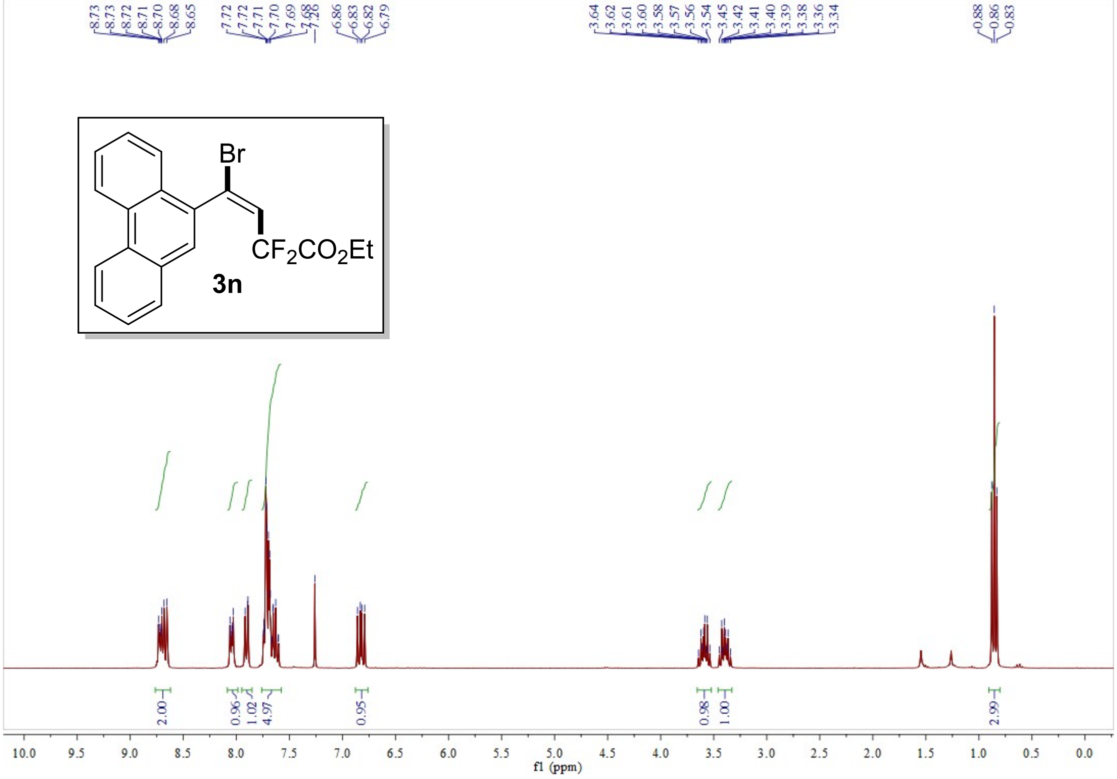

8.73 8.73 8.72 8.71 8.70 8.68 8.65
7.72 7.72 7.71 7.70 7.69 7.68 7.26
6.86 6.83 6.82 6.79
3.64 3.62 3.61 3.60 3.58 3.57 3.56 3.54 3.45 3.42 3.41 3.40 3.39 3.38 3.36 3.34
0.88 0.86 0.83
Br
CF2CO2Et
3n
2.00 0.96 1.02 4.97 0.95 0.98 1.00 2.99
10.0 9.5 9.0 8.5 8.0 7.5 7.0 6.5 6.0 5.5 5.0 4.5 4.0 3.5 3.0 2.5 2.0 1.5 1.0 0.5 0.0
f1 (ppm)

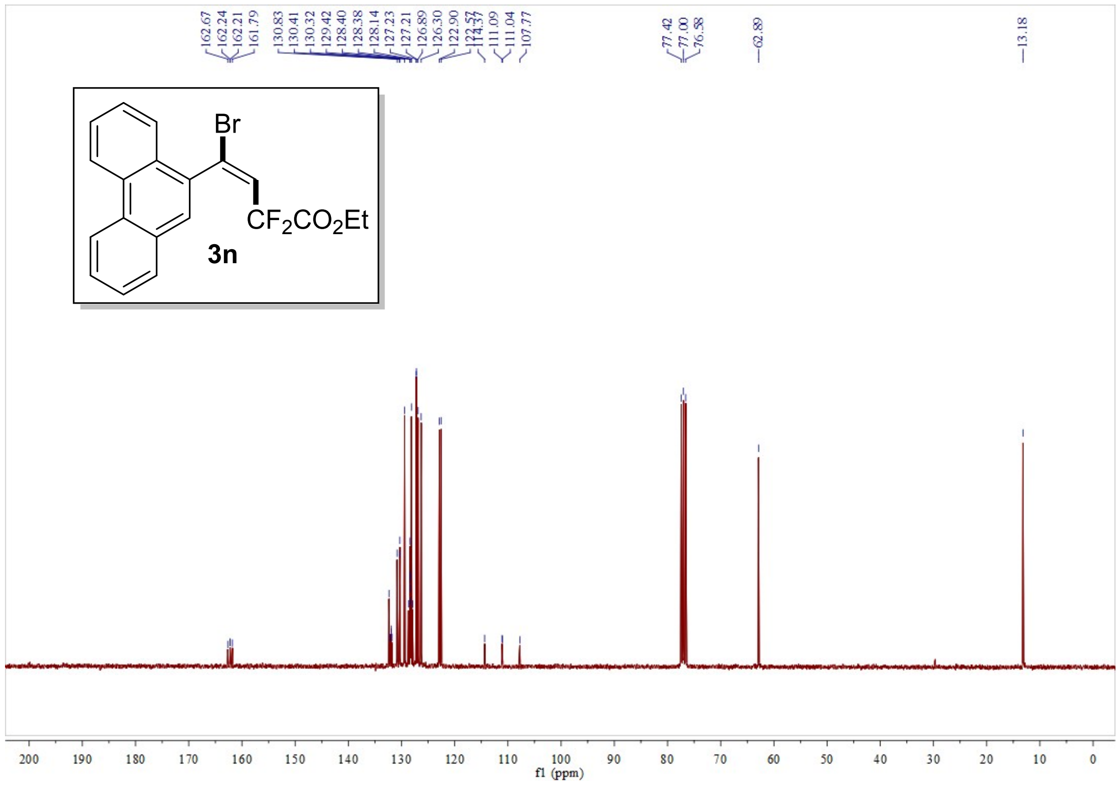
162.67
162.24
162.21
161.79
130.83
130.41
130.32
129.42
128.40
128.38
128.14
127.23
127.21
126.89
126.30
122.90
122.57
114.37
111.09
111.04
107.77
77.42
77.00
76.58
62.89
13.18
Br
CF2CO2Et
3n
200 190 180 170 160 150 140 130 120 110 100 90 80 70 60 50 40 30 20 10 0
f1 (ppm)

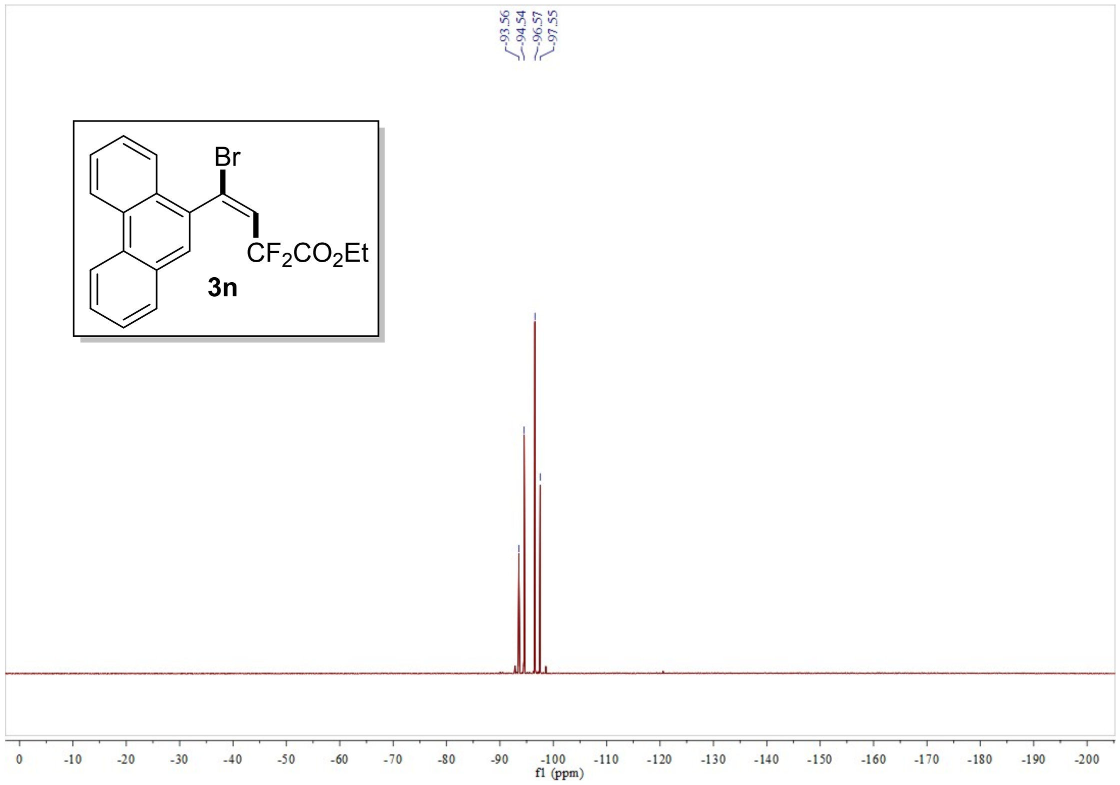
-93.56
-94.54
-96.57
-97.55
Br
CF2CO2Et
3n
0 -10 -20 -30 -40 -50 -60 -70 -80 -90 -100 -110 -120 -130 -140 -150 -160 -170 -180 -190 -200
f1 (ppm)

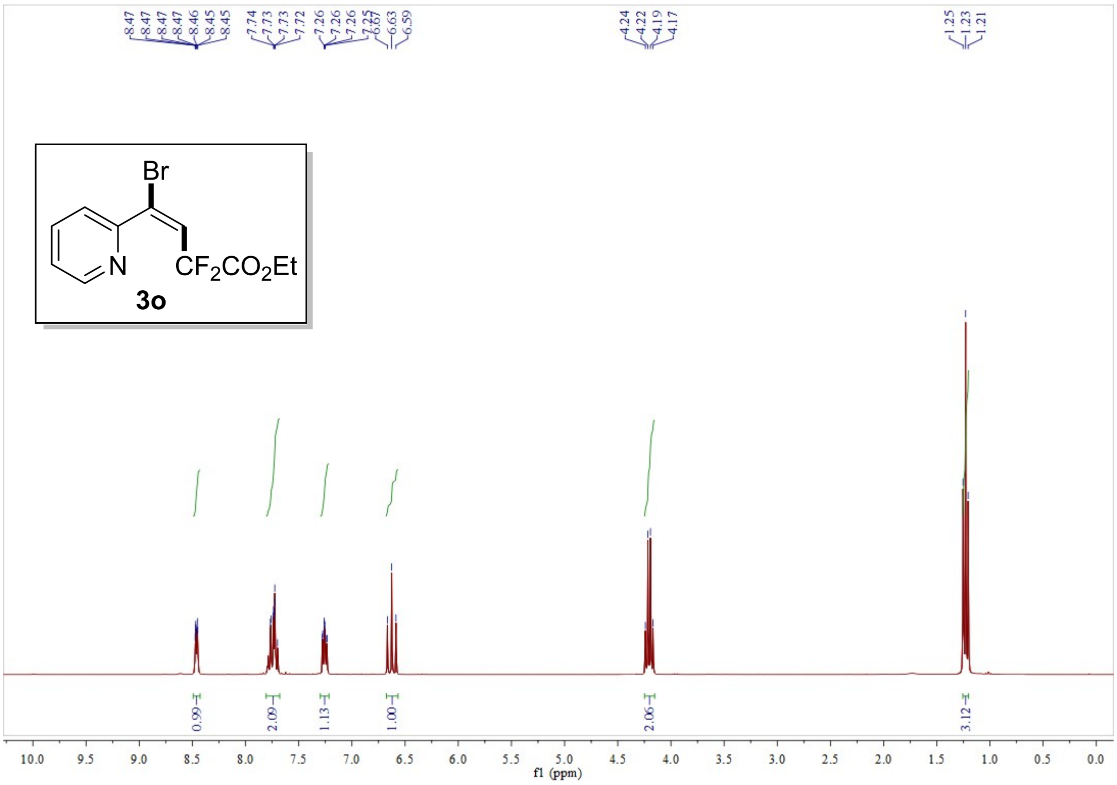
8.47
8.47
8.47
8.47
8.46
8.45
8.45
7.74
7.73
7.73
7.72
7.26
7.26
7.26
7.25
6.67
6.63
6.59
4.24
4.22
4.19
4.17
1.25
1.23
1.21
Br
N
CF2CO2Et
3o
0.99
2.09
1.13
1.00
2.06
3.12
f1 (ppm)

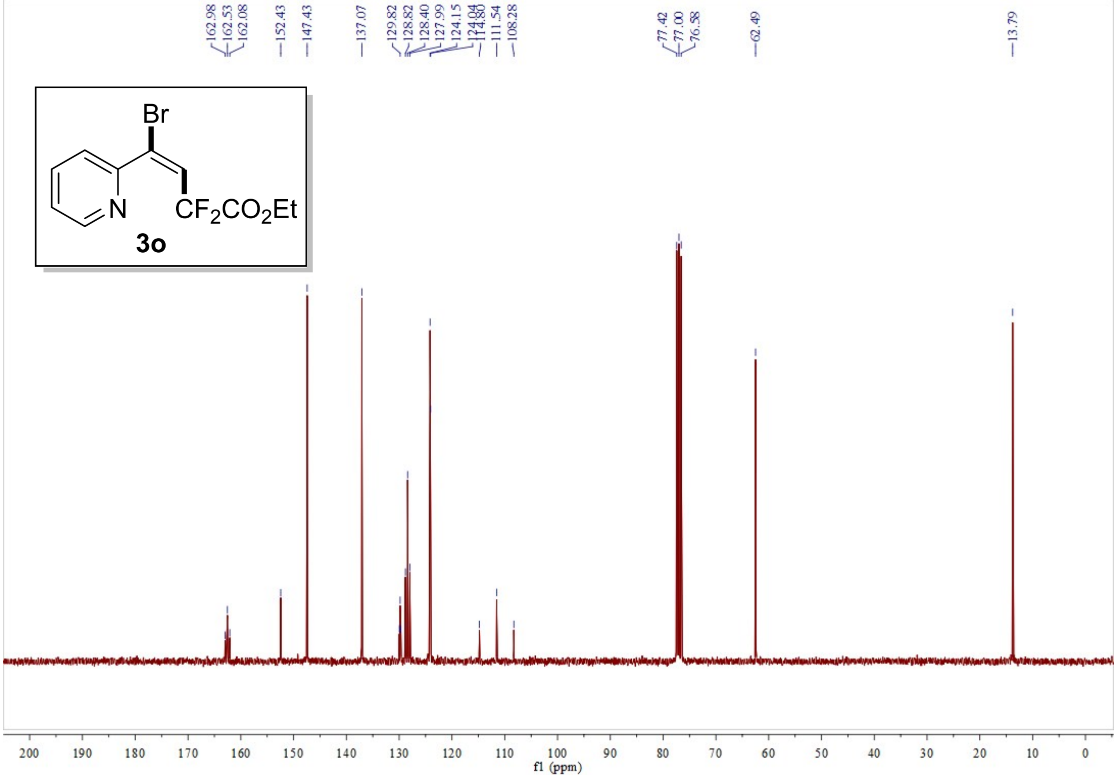
162.98
162.53
162.08
152.43
147.43
137.07
129.82
128.82
128.40
127.99
124.15
124.04
114.80
111.54
108.28
77.42
77.00
76.58
62.49
13.79
Br
N
CF2CO2Et
3o
f1 (ppm)

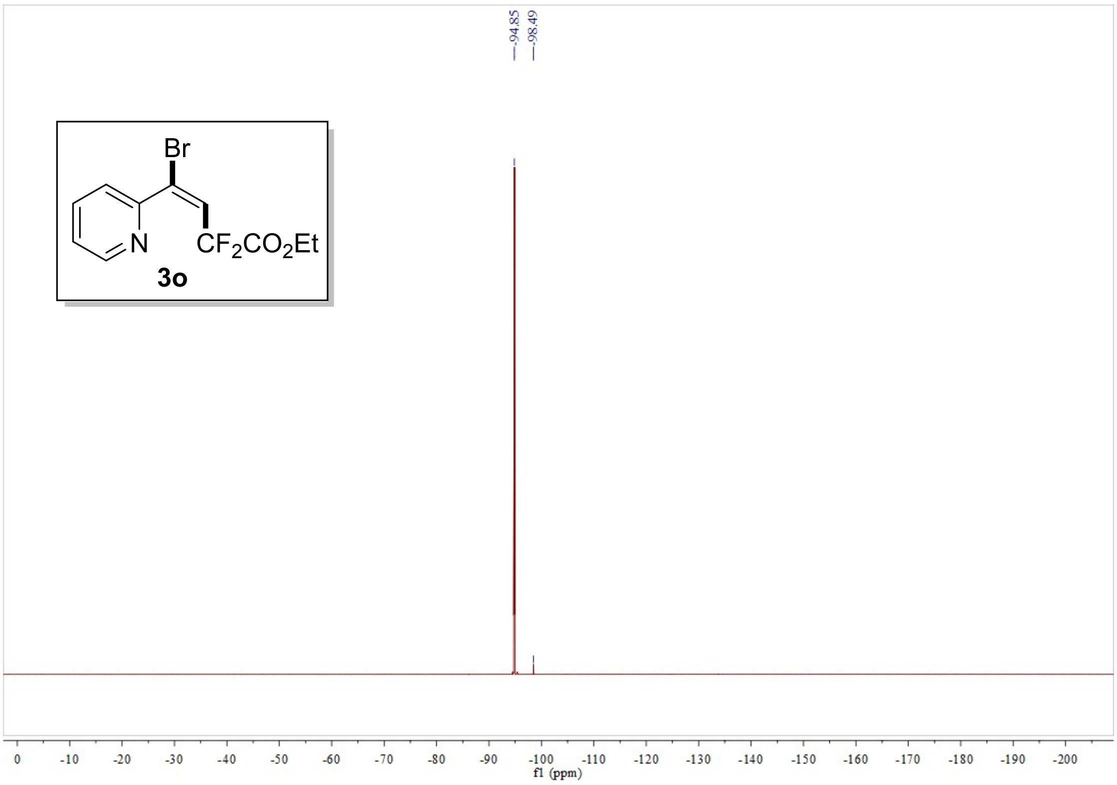
-94.85
-98.49
Br
N
CF2CO2Et
3o
0 -10 -20 -30 -40 -50 -60 -70 -80 -90 -100 -110 -120 -130 -140 -150 -160 -170 -180 -190 -200
f1 (ppm)

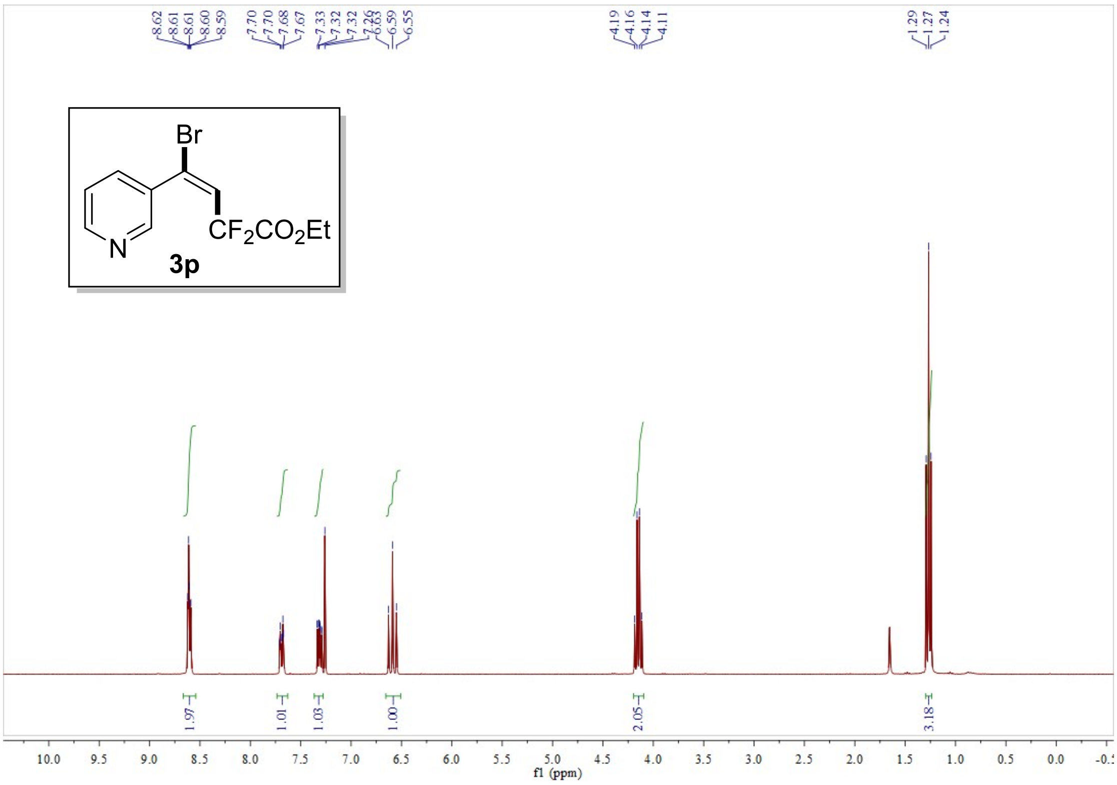
8.62 8.61 8.61 8.60 8.59 7.70 7.70 7.68 7.67 7.33 7.32 7.32 6.59 6.55
4.19 4.16 4.14 4.11
1.29 1.27 1.24
Br
N
CF2CO2Et
3p
1.97 1.01 1.03 1.00 2.05 3.18
10.0 9.5 9.0 8.5 8.0 7.5 7.0 6.5 6.0 5.5 5.0 4.5 4.0 3.5 3.0 2.5 2.0 1.5 1.0 0.5 0.0
f1 (ppm)

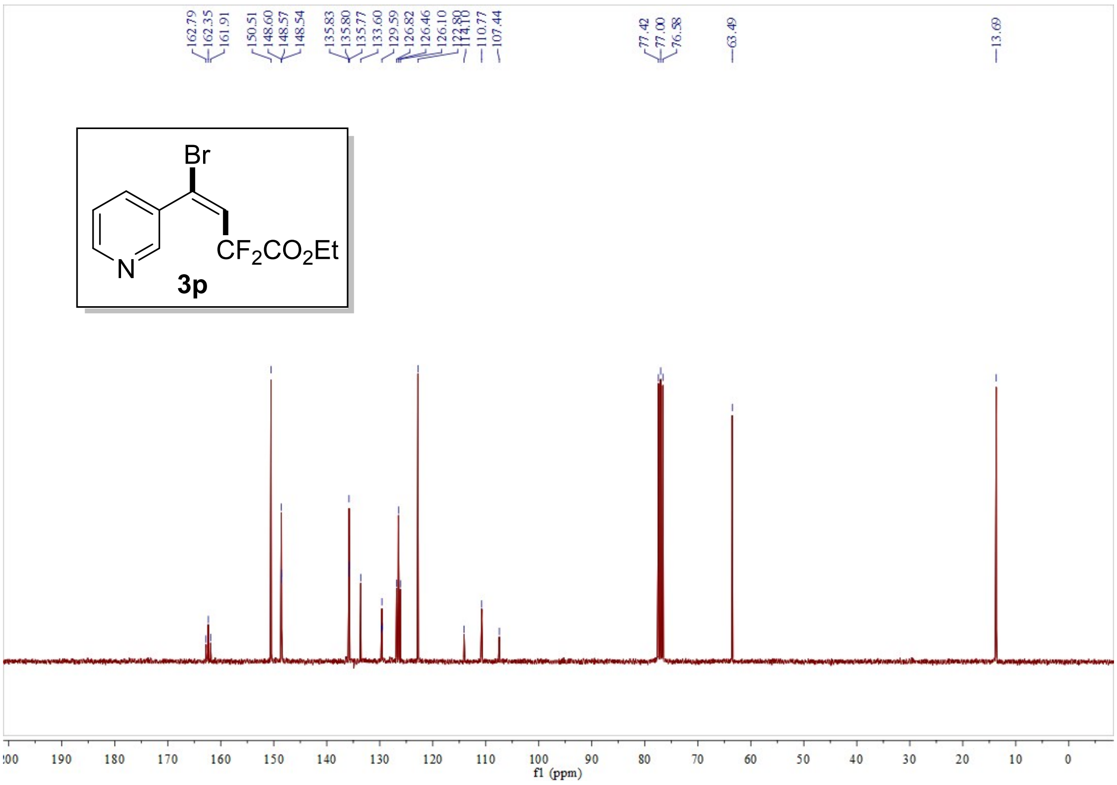
162.79
162.35
161.91
150.51
148.60
148.57
148.54
135.83
135.80
135.77
133.60
129.59
126.82
126.46
126.10
122.80
114.10
110.77
107.44
77.42
77.00
76.58
63.49
13.69
Br
CF2CO2Et
N
3p
fl (ppm)

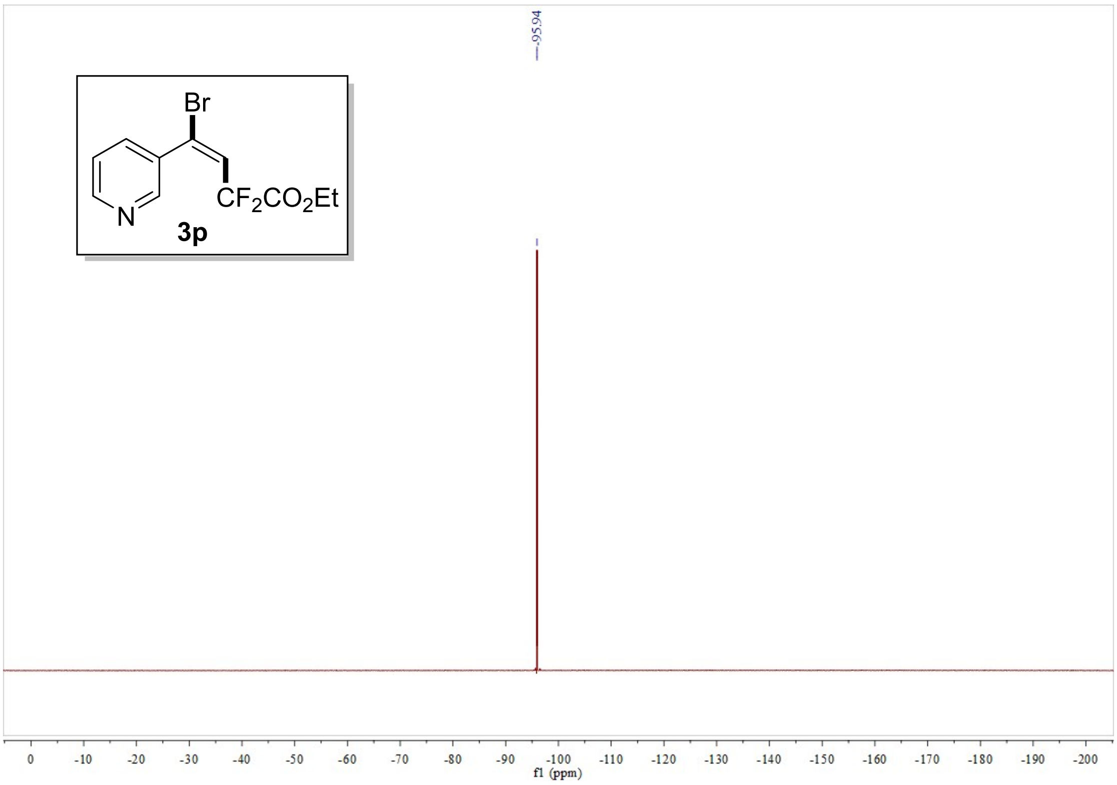
-95.94
Br
CF2CO2Et
N
3p
fl (ppm)

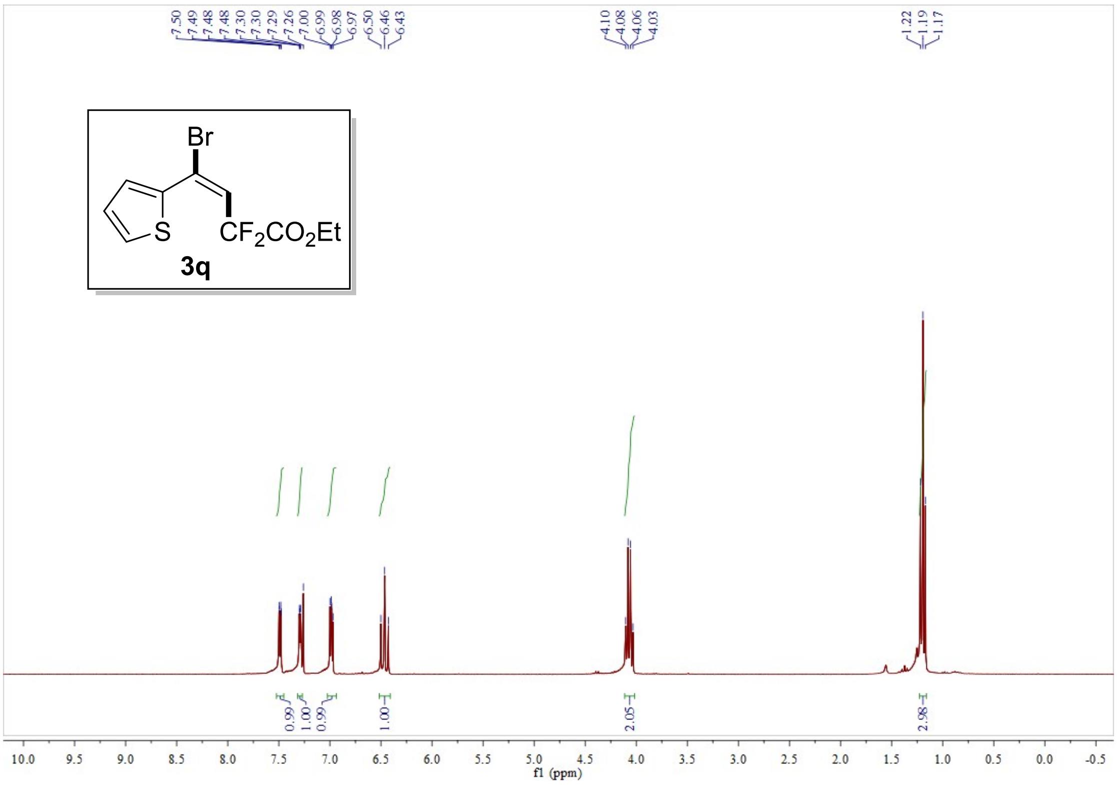
7.50
7.49
7.48
7.48
7.30
7.30
7.29
7.26
7.00
6.99
6.98
6.97
6.50
6.46
6.43
4.10
4.08
4.06
4.03
1.22
1.19
1.17
Br
S
CF2CO2Et
3q
0.99
1.00
0.99
1.00
2.05
2.98
10.0 9.5 9.0 8.5 8.0 7.5 7.0 6.5 6.0 5.5 5.0 4.5 4.0 3.5 3.0 2.5 2.0 1.5 1.0 0.5 0.0 -0.5
f1 (ppm)

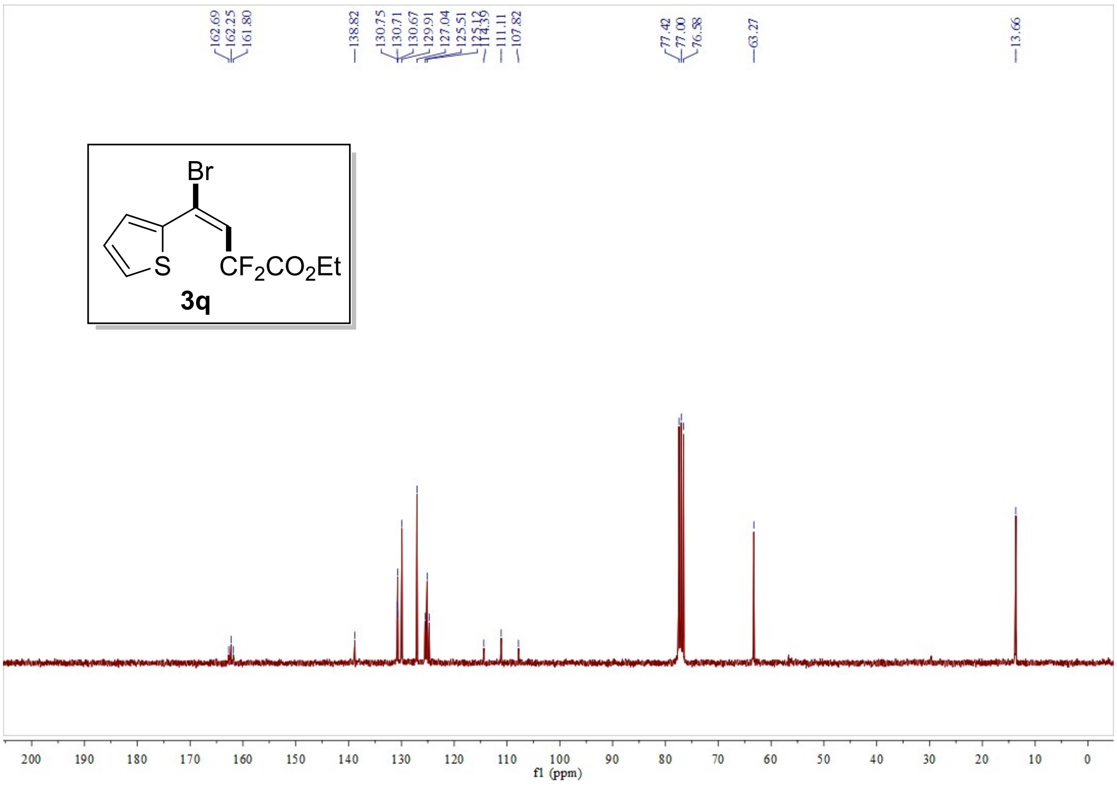
162.69
162.25
161.80
138.82
130.75
130.71
130.67
129.91
127.04
125.51
125.12
114.39
111.11
107.82
77.42
77.00
76.58
63.27
13.66
Br
S
CF2CO2Et
3q
200 190 180 170 160 150 140 130 120 110 100 90 80 70 60 50 40 30 20 10 0
f1 (ppm)

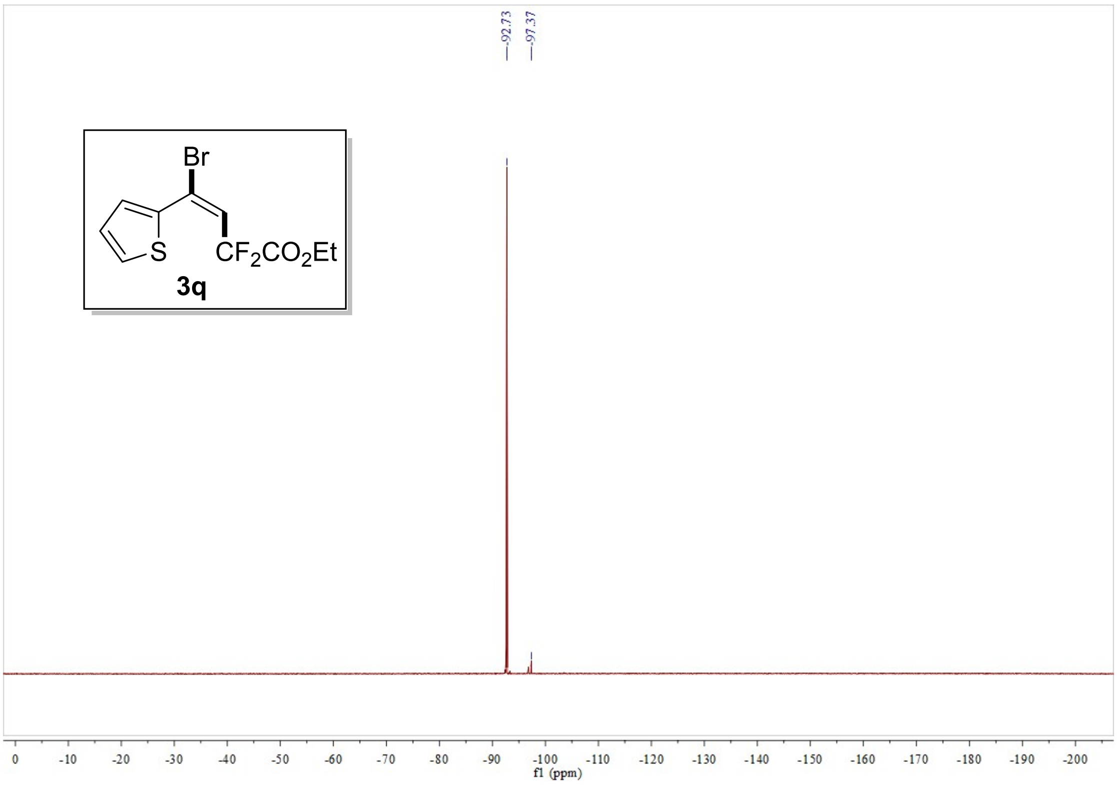
-92.73
-97.37
Br
S
CF2CO2Et
3q
f1 (ppm)

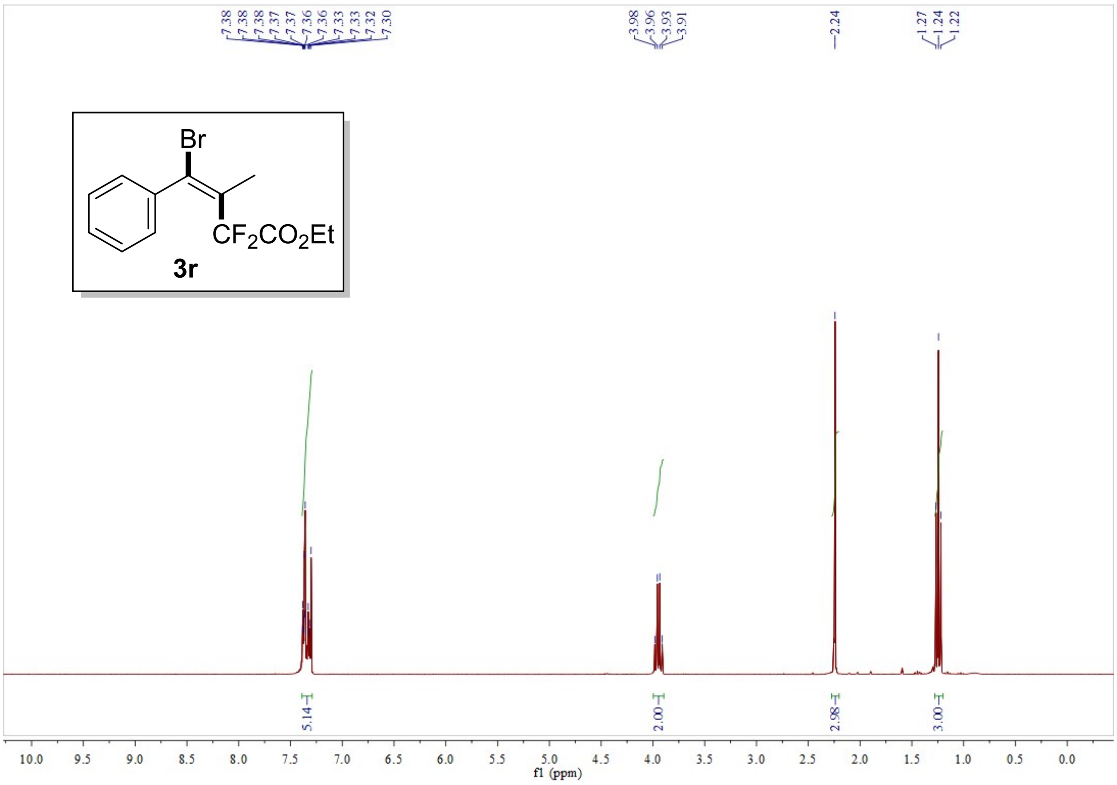
7.38
7.38
7.38
7.37
7.37
7.36
7.36
7.33
7.33
7.32
7.30
3.98
3.96
3.93
3.91
2.24
1.27
1.24
1.22
Br
CF2CO2Et
3r
5.14
2.00
2.98
3.00
f1 (ppm)

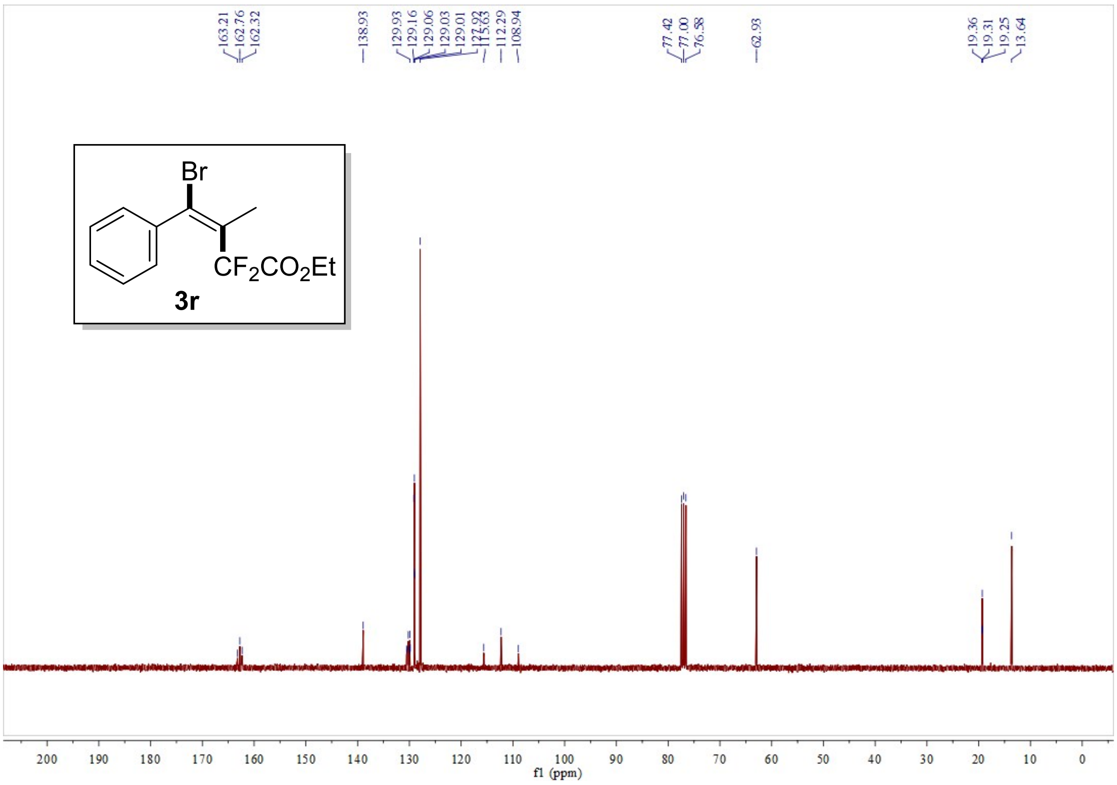
163.21
162.76
162.32
138.93
129.93
129.16
129.06
129.03
129.01
127.92
115.63
112.29
108.94
77.42
77.00
76.58
62.93
19.36
19.31
19.25
13.64
Br
CF_2CO_2Et
3r
200
190
180
170
160
150
140
130
120
110
100
90
80
70
60
50
40
30
20
10
0
f1 (ppm)

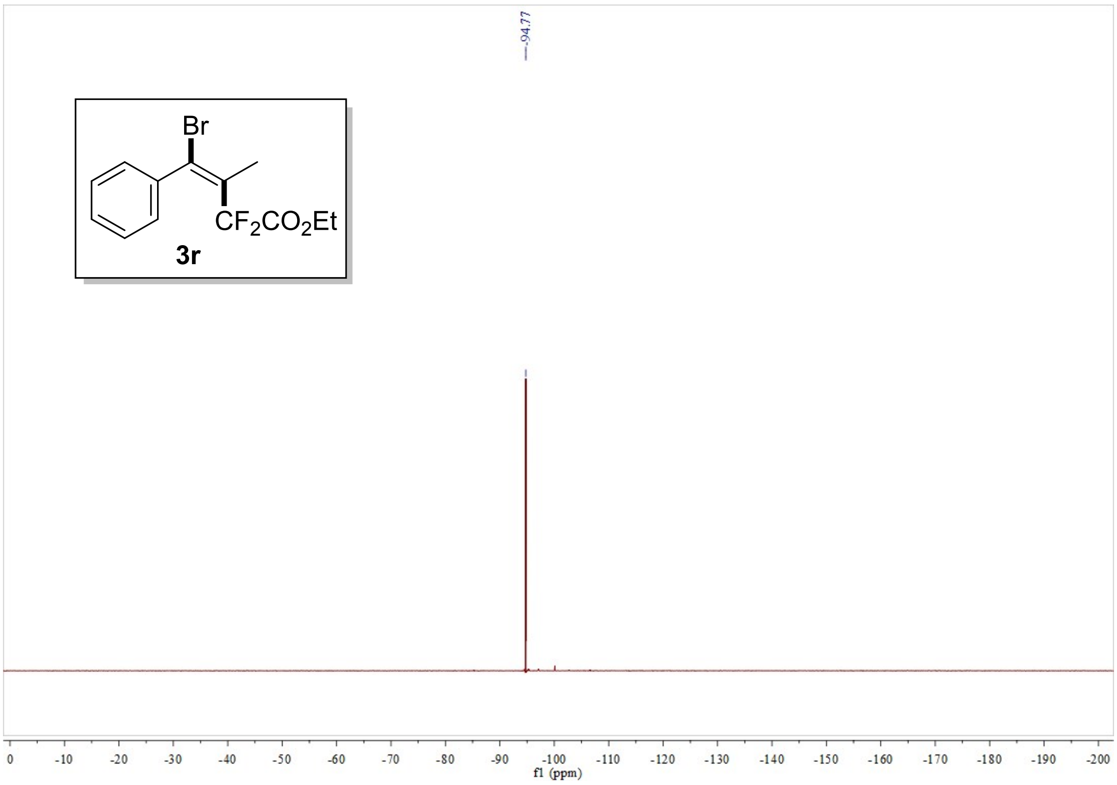
-94.77
Br
CF_2CO_2Et
3r
0
-10
-20
-30
-40
-50
-60
-70
-80
-90
-100
-110
-120
-130
-140
-150
-160
-170
-180
-190
-200
f1 (ppm)

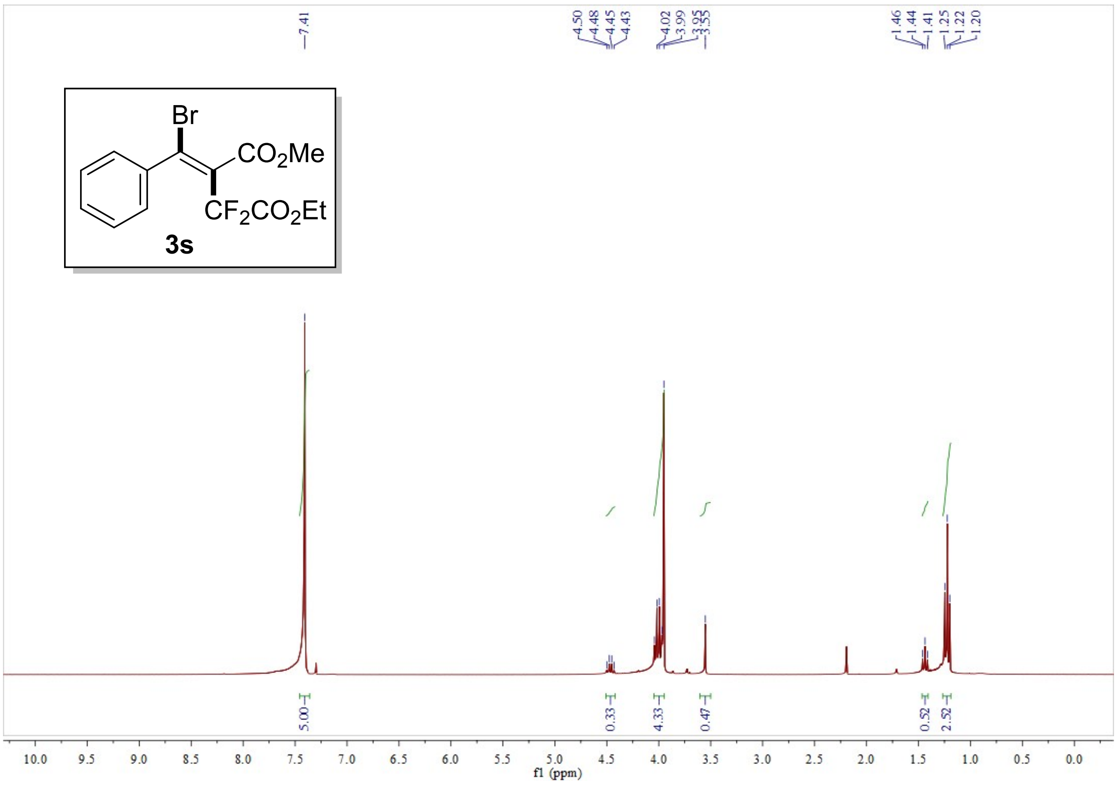
7.41
4.50
4.48
4.45
4.43
4.02
3.99
3.95
3.55
1.46
1.44
1.41
1.25
1.22
1.20
Br
CO2Me
CF2CO2Et
3s
5.00
0.33
4.33
0.47
0.52
2.52
f1 (ppm)

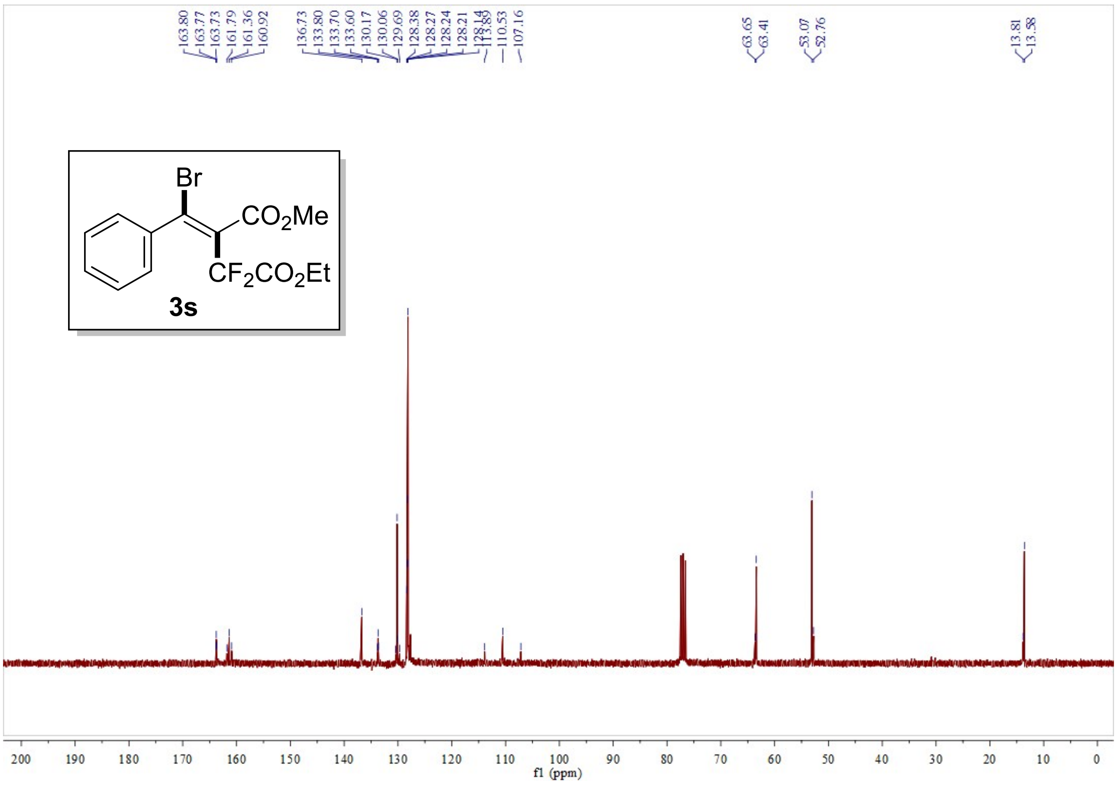
163.80
163.77
163.73
161.79
161.36
160.92
136.73
133.80
133.70
133.60
130.17
130.06
129.69
128.38
128.27
128.24
128.21
110.53
107.16
63.65
63.41
53.07
52.76
13.81
13.58
Br
CO2Me
CF2CO2Et
3s
f1 (ppm)

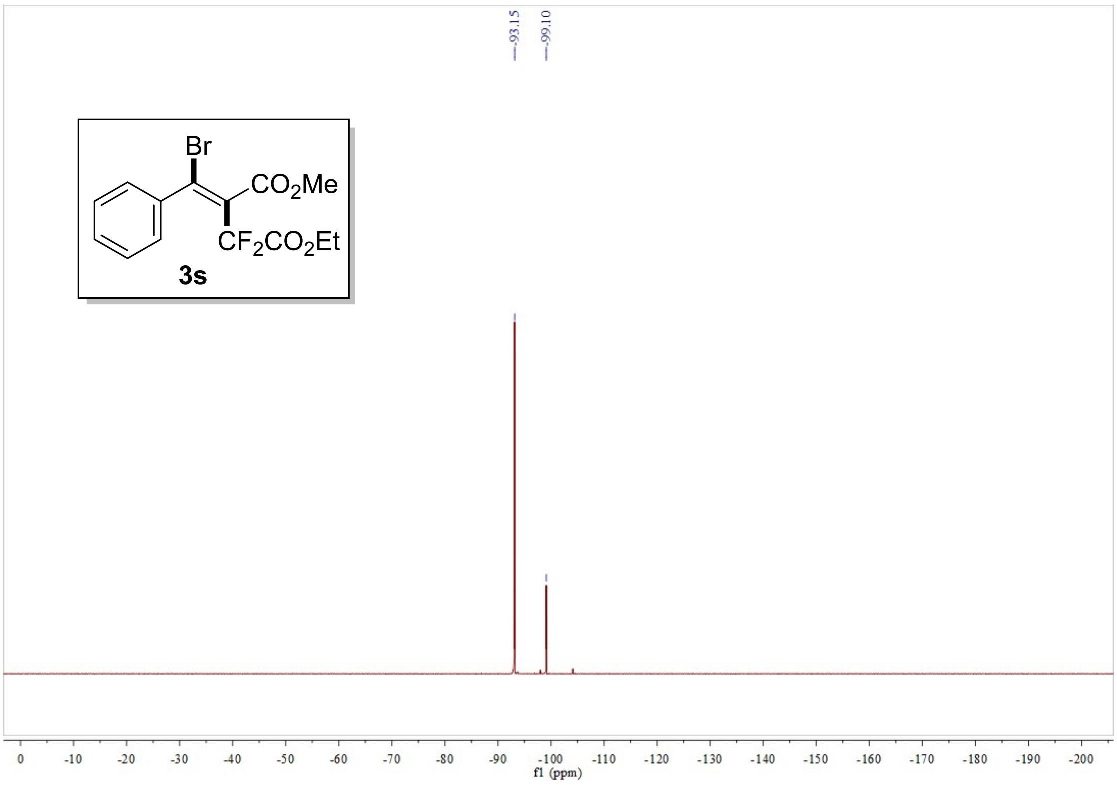
-93.15
-99.10
Br
CO_2Me
CF_2CO_2Et
3s
0 -10 -20 -30 -40 -50 -60 -70 -80 -90 -100 -110 -120 -130 -140 -150 -160 -170 -180 -190 -200
f1 (ppm)

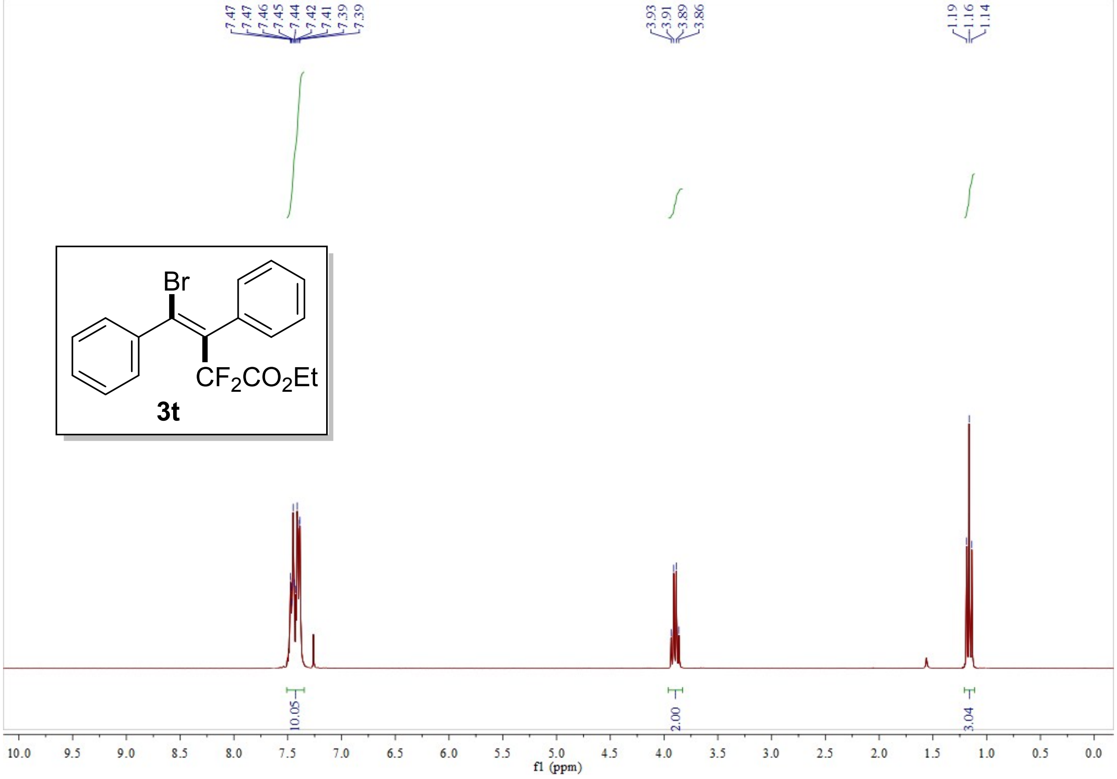
7.47 7.47 7.46 7.45 7.44 7.42 7.41 7.39 7.39
3.93 3.91 3.89 3.86
1.19 1.16 1.14
Br
CF_2CO_2Et
3t
10.05
2.00
3.04
10.0 9.5 9.0 8.5 8.0 7.5 7.0 6.5 6.0 5.5 5.0 4.5 4.0 3.5 3.0 2.5 2.0 1.5 1.0 0.5 0.0
f1 (ppm)

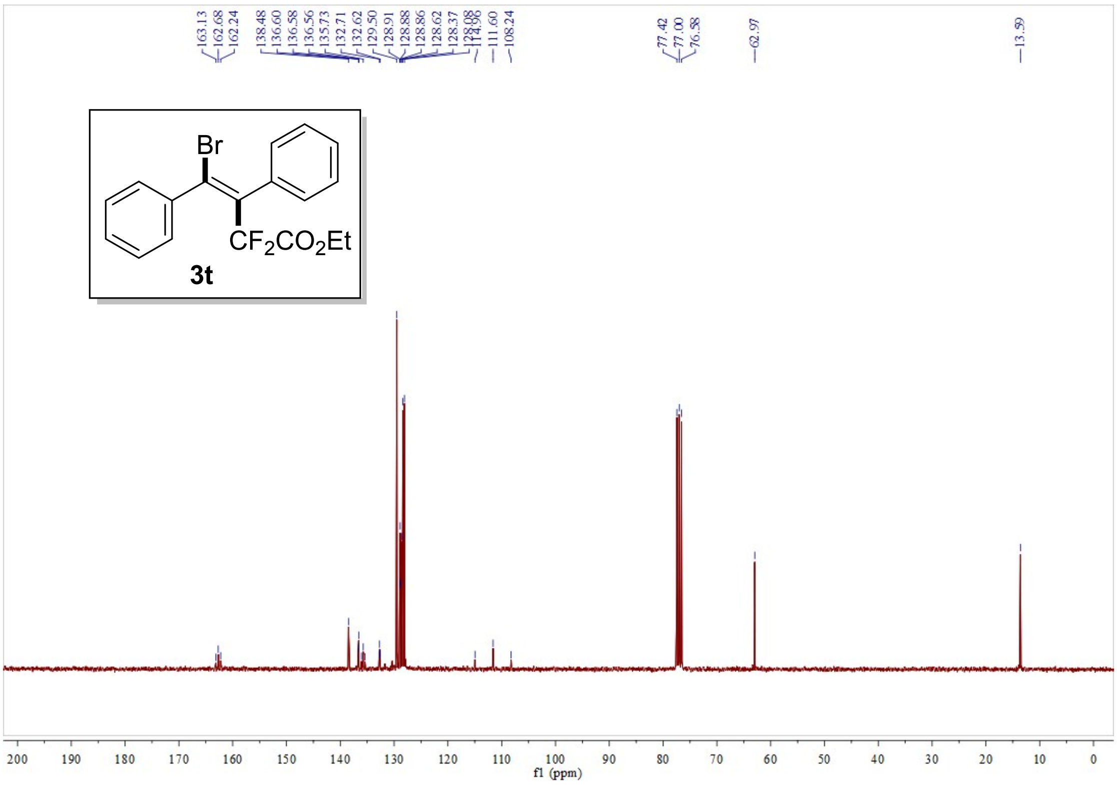
163.13
162.68
162.24
138.48
136.60
136.58
136.56
135.73
132.71
132.62
129.50
128.91
128.88
128.86
128.62
128.37
128.08
114.96
111.60
108.24
77.42
77.00
76.58
62.97
13.59
Br
CF2CO2Et
3t
200 190 180 170 160 150 140 130 120 110 100 90 80 70 60 50 40 30 20 10 0
f1 (ppm)

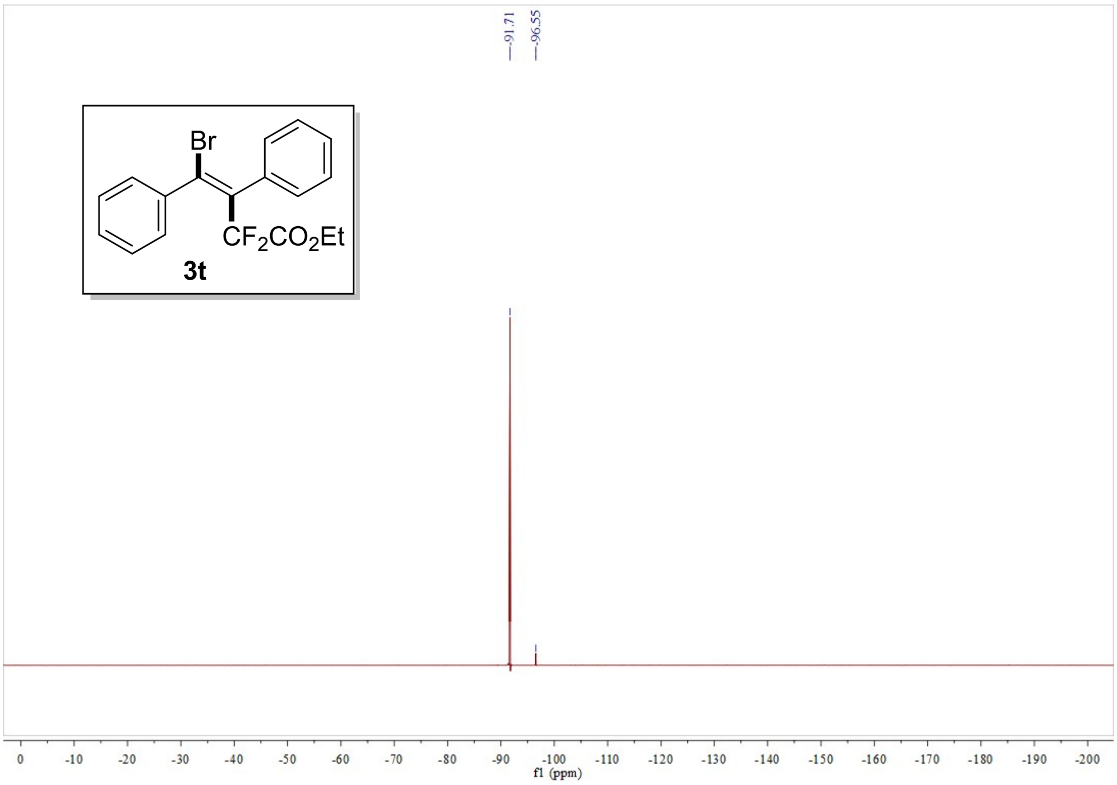
-91.71
-96.55
Br
CF2CO2Et
3t
0 -10 -20 -30 -40 -50 -60 -70 -80 -90 -100 -110 -120 -130 -140 -150 -160 -170 -180 -190 -200
f1 (ppm)

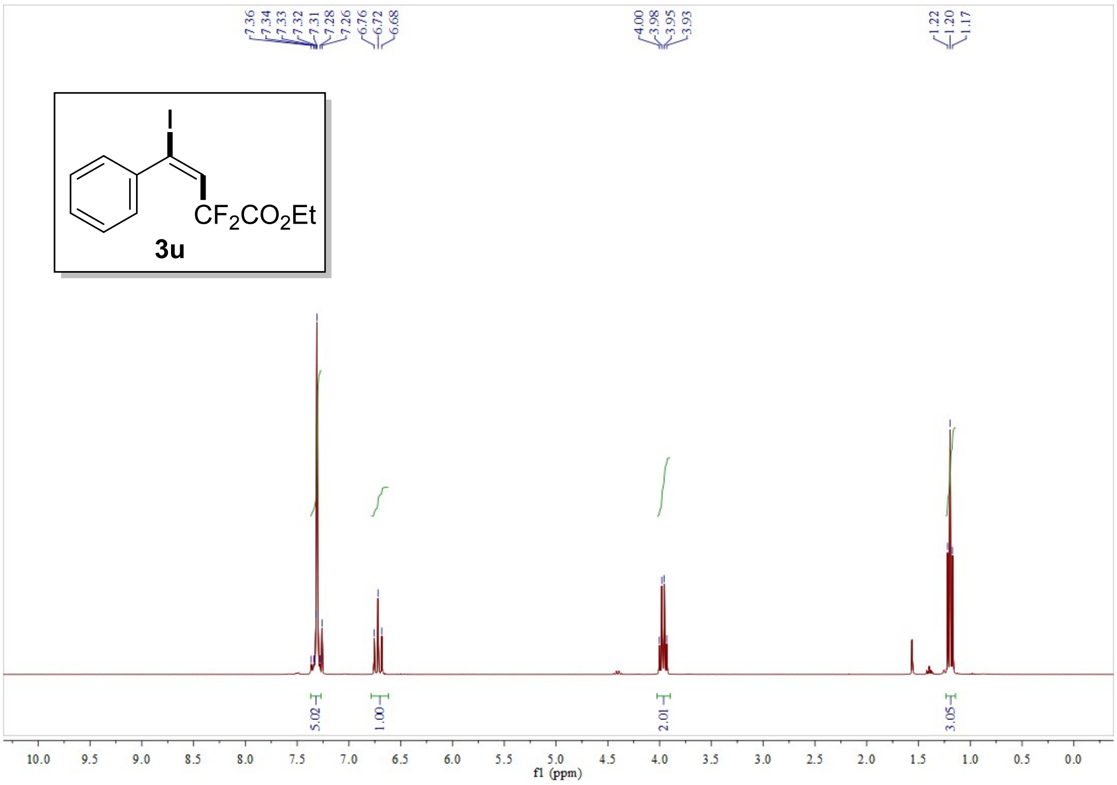
7.36
7.34
7.33
7.32
7.31
7.28
7.26
6.76
6.72
6.68
4.00
3.98
3.95
3.93
1.22
1.20
1.17
I
CF2CO2Et
3u
5.02
1.00
2.01
3.05
10.0
9.5
9.0
8.5
8.0
7.5
7.0
6.5
6.0
5.5
5.0
4.5
4.0
3.5
3.0
2.5
2.0
1.5
1.0
0.5
0.0
f1 (ppm)

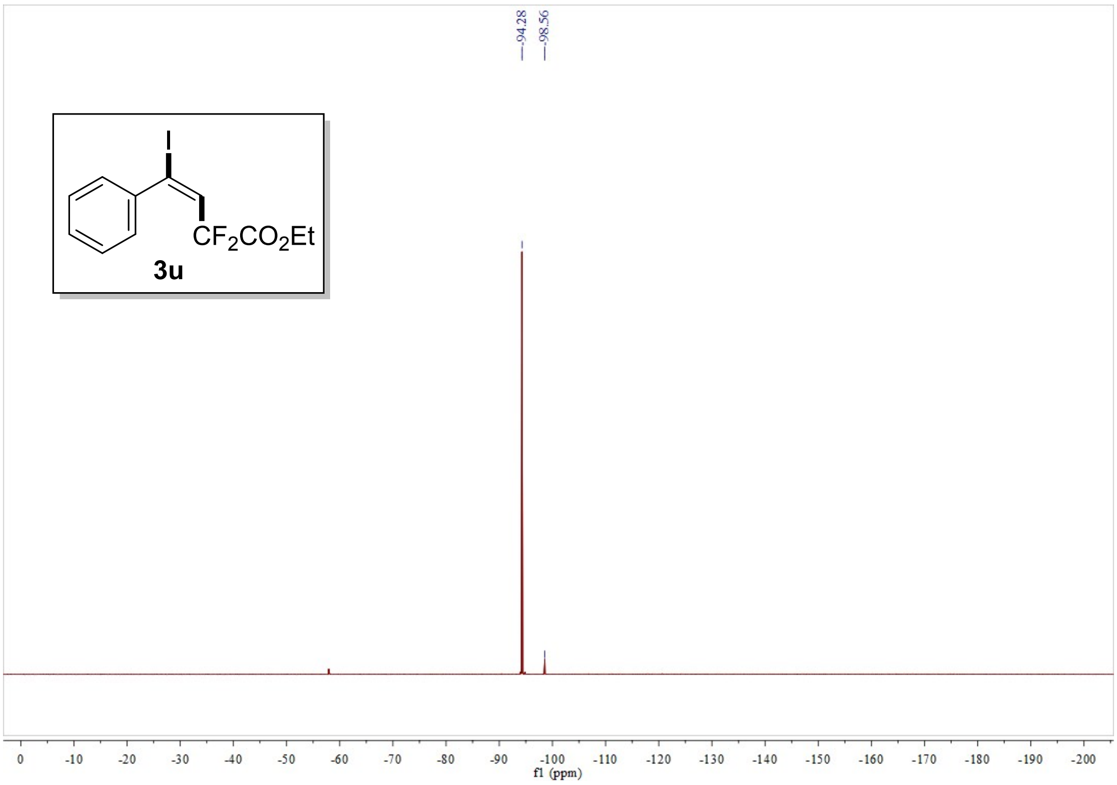
-94.28
-98.56
I
CF2CO2Et
3u
0
-10
-20
-30
-40
-50
-60
-70
-80
-90
-100
-110
-120
-130
-140
-150
-160
-170
-180
-190
-200
f1 (ppm)

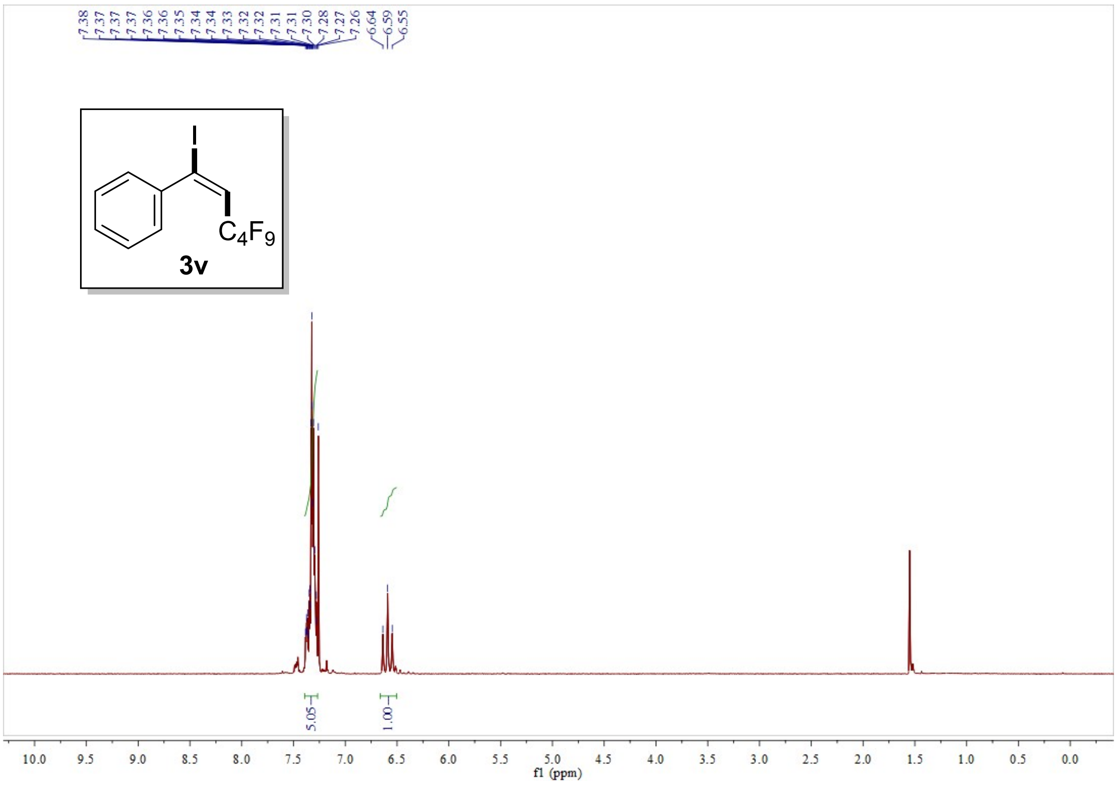
7.38
7.37
7.37
7.37
7.36
7.36
7.35
7.34
7.34
7.33
7.32
7.32
7.31
7.31
7.30
7.28
7.27
7.26
6.64
6.59
6.55
C4F9
3v
5.05
1.00
f1 (ppm)

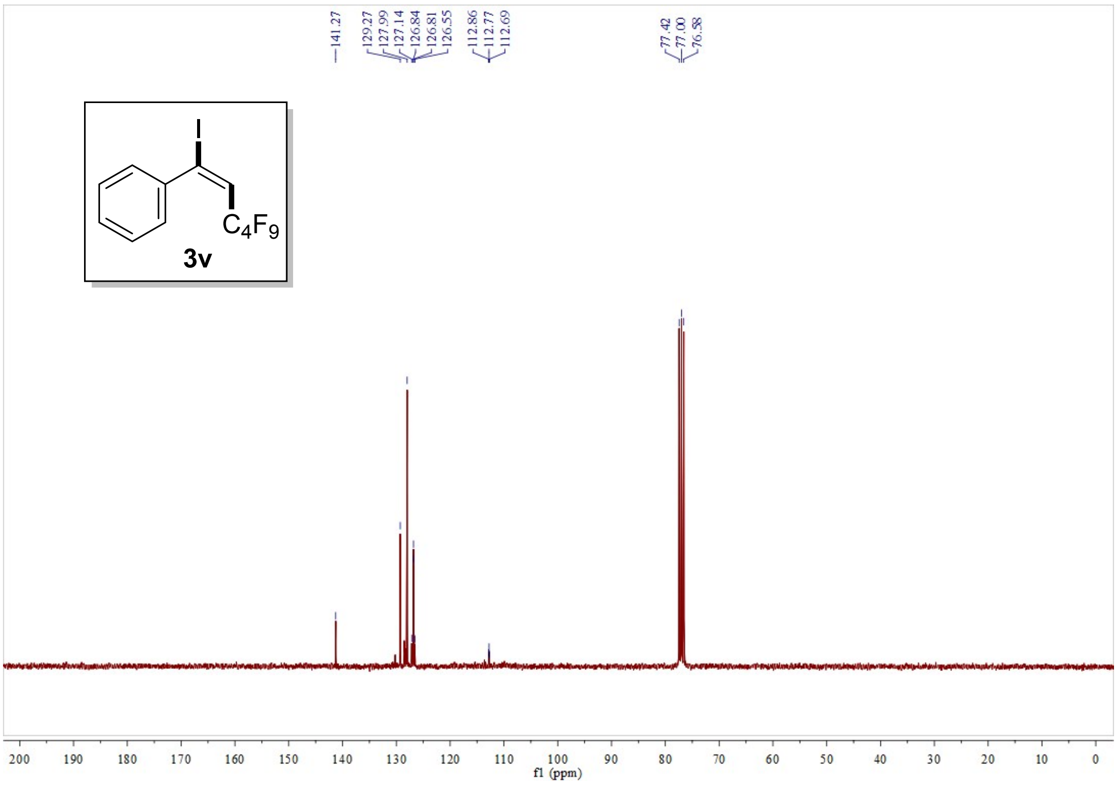
141.27
129.27
127.99
127.14
126.84
126.81
126.55
112.86
112.77
112.69
77.42
77.00
76.58
C4F9
3v
f1 (ppm)

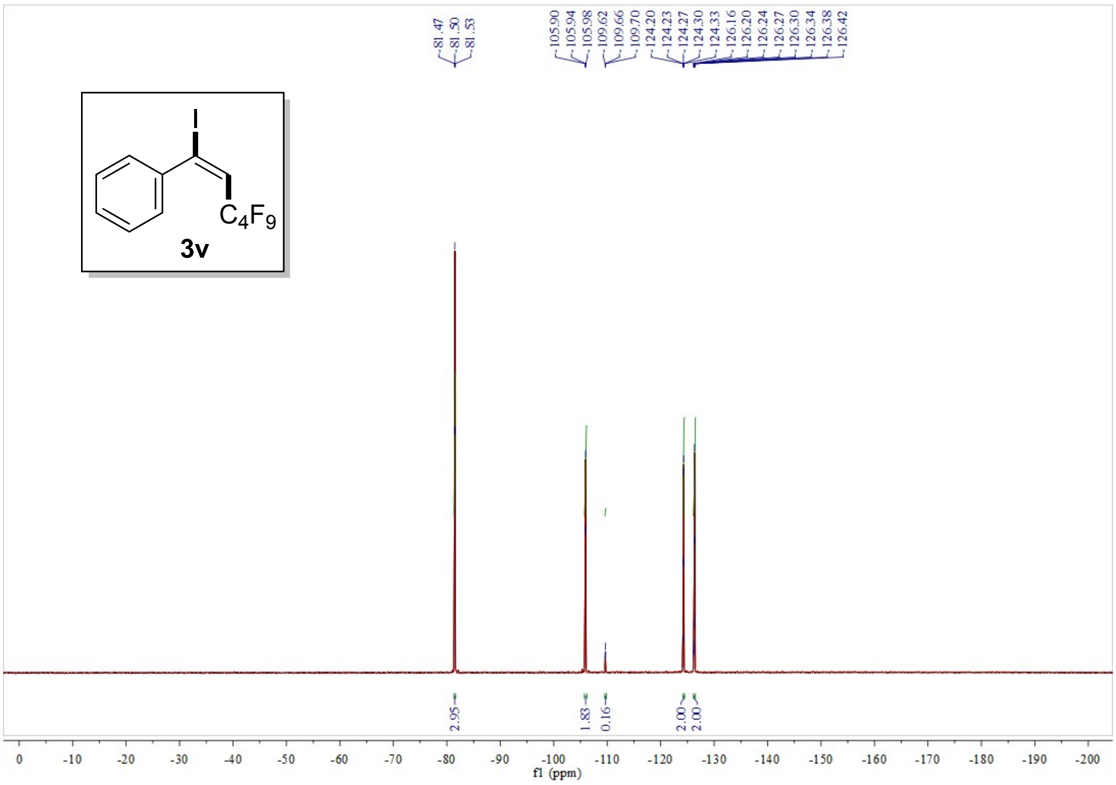

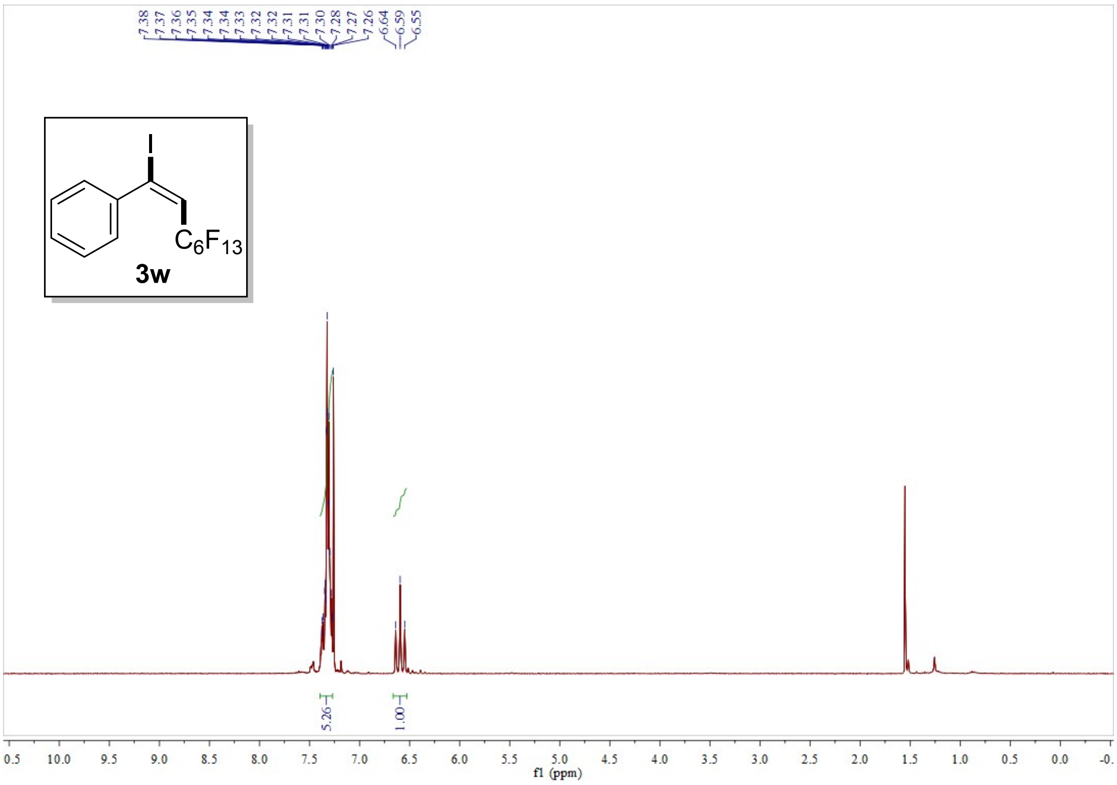

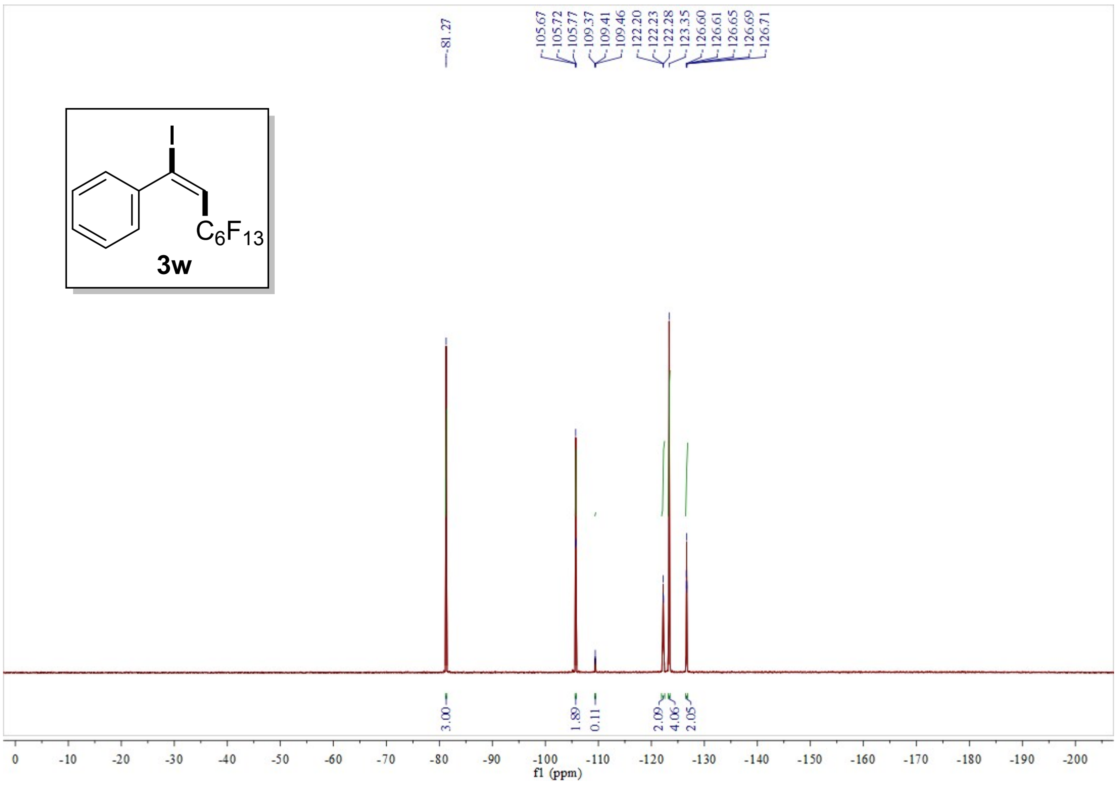

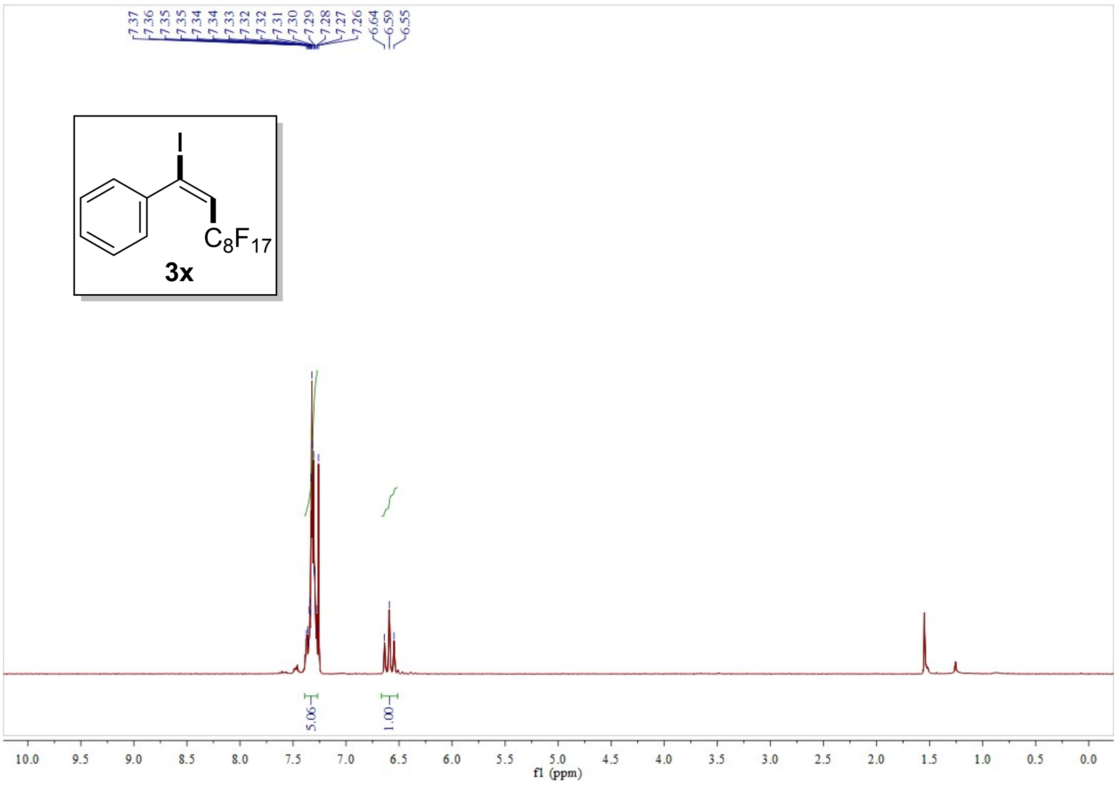

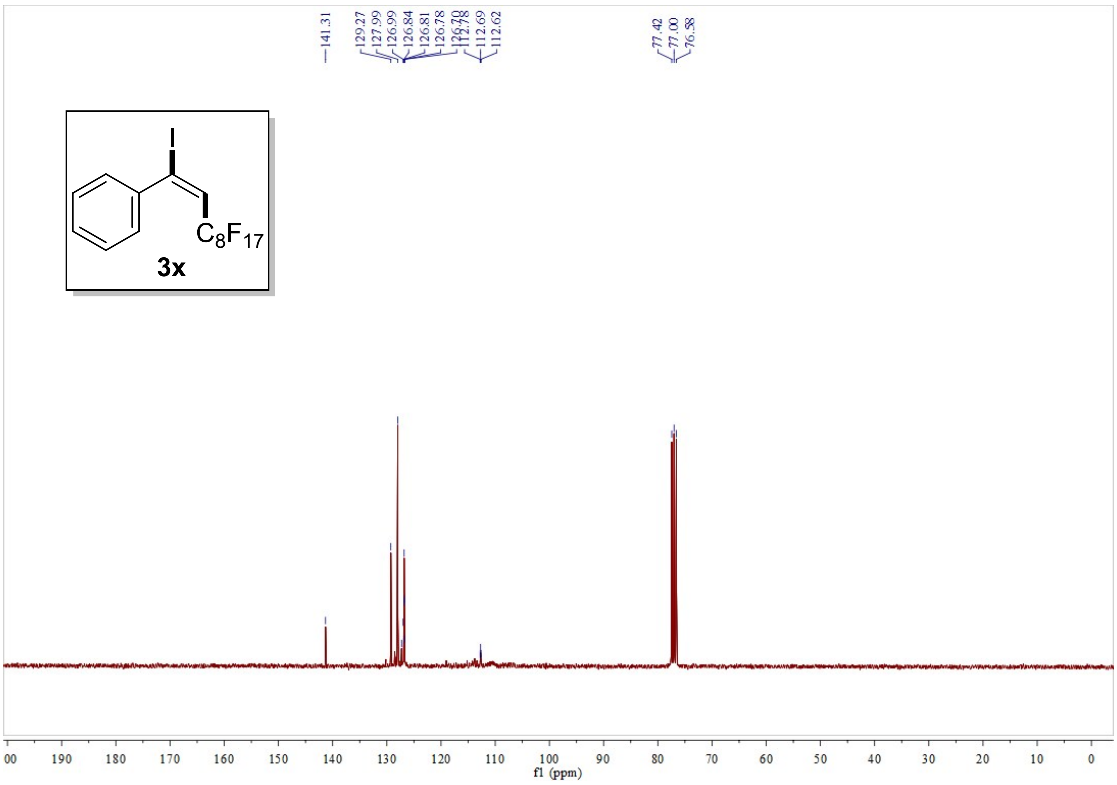
141.31
129.27
127.99
126.99
126.84
126.81
126.78
112.69
112.62
77.42
77.00
76.58
3x
C8F17
I
f1 (ppm)

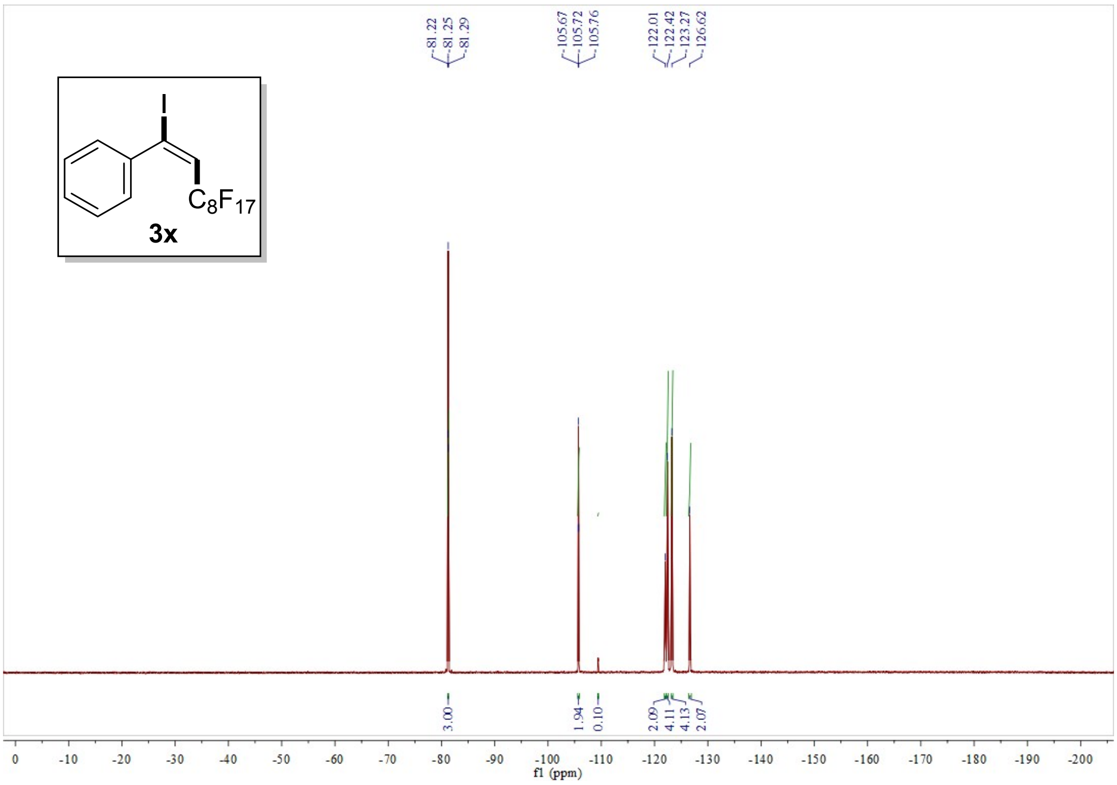
-81.22
-81.25
-81.29
-105.67
-105.72
-105.76
-122.01
-122.42
-123.27
-126.62
3x
C8F17
I
3.00
1.94
0.10
2.09
4.11
4.13
2.07
f1 (ppm)

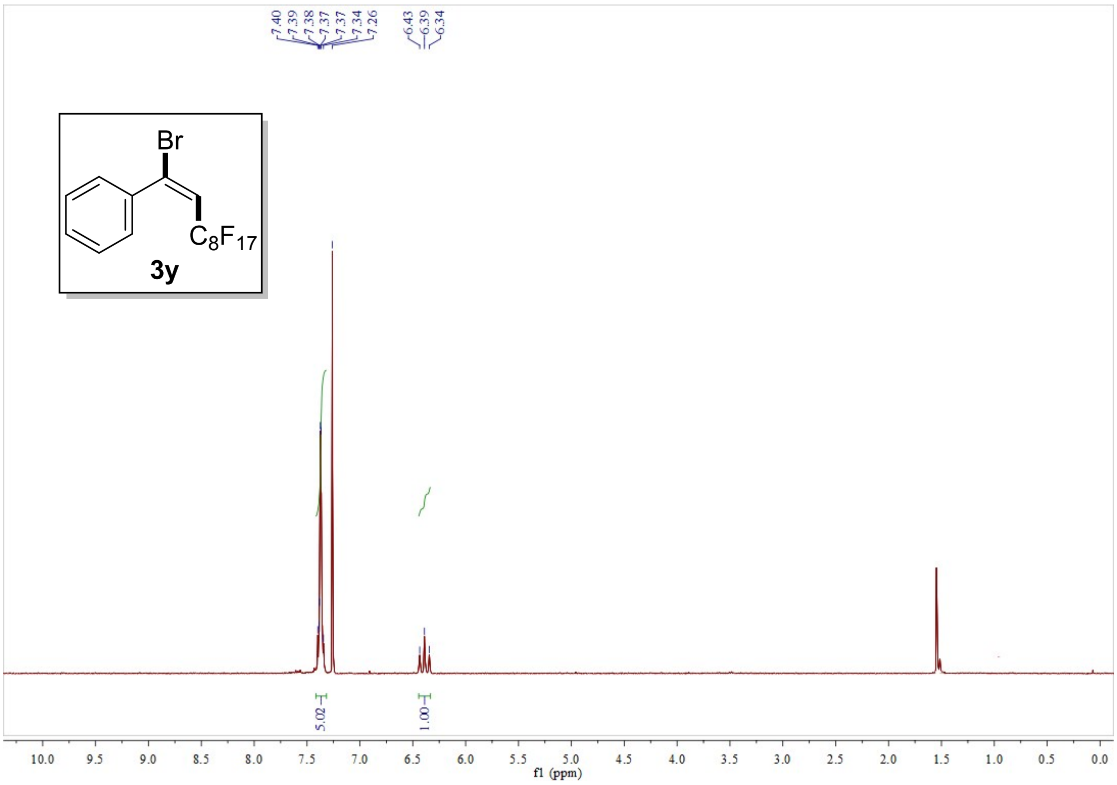
7.40 7.39 7.38 7.37 7.37 7.34 7.26
6.43 6.39 6.34
Br
C8F17
3y
5.02
1.00
f1 (ppm)

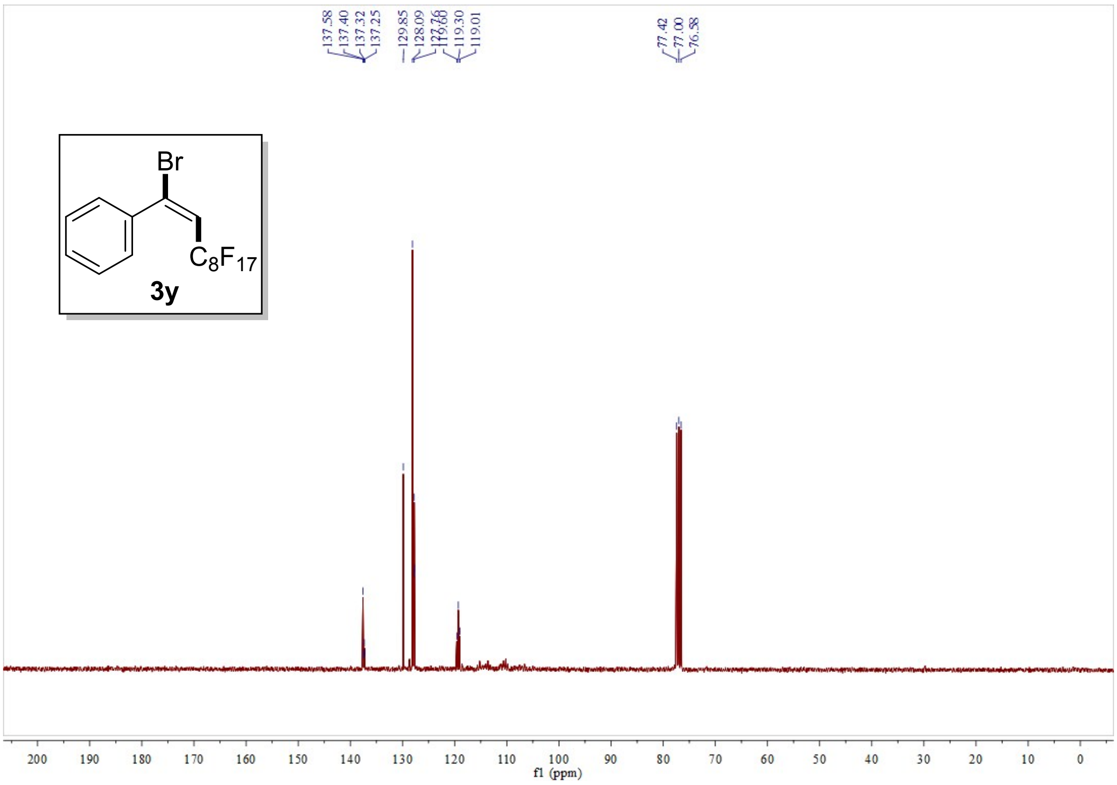
137.58 137.40 137.32 137.25
129.85 128.09 119.30 119.01
77.42 77.00 76.58
Br
C8F17
3y
f1 (ppm)

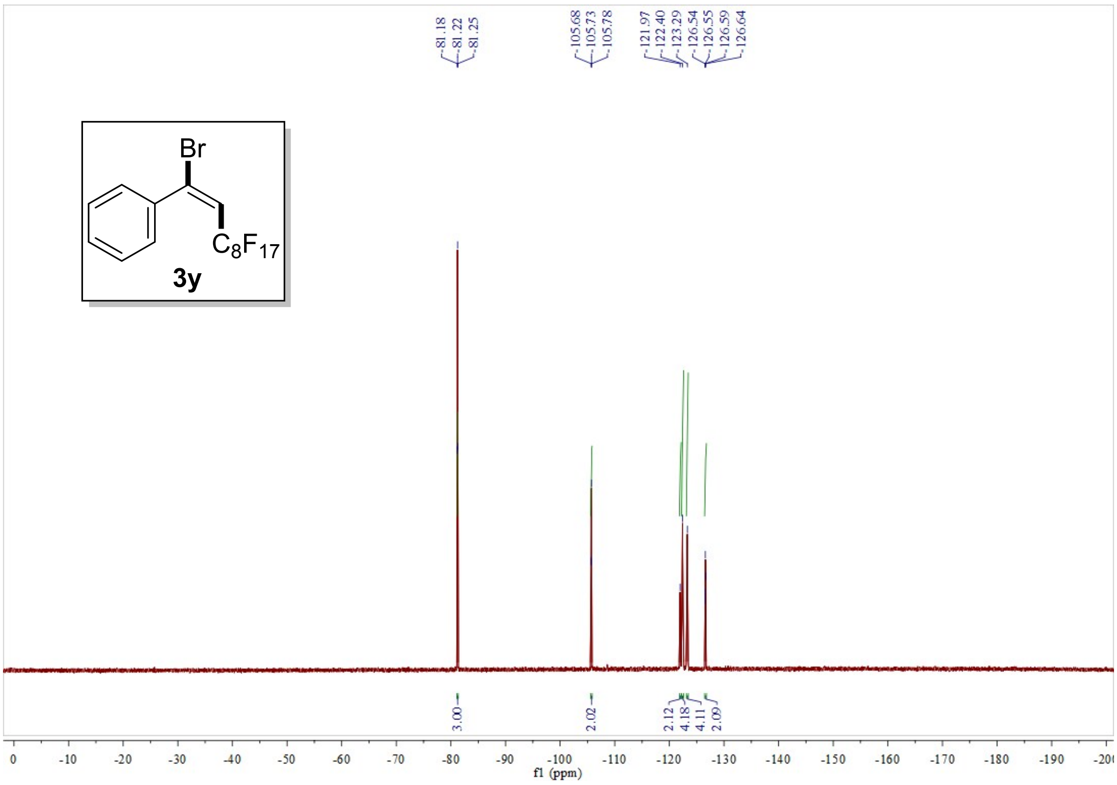
Br
C8F17
3y
f1 (ppm)

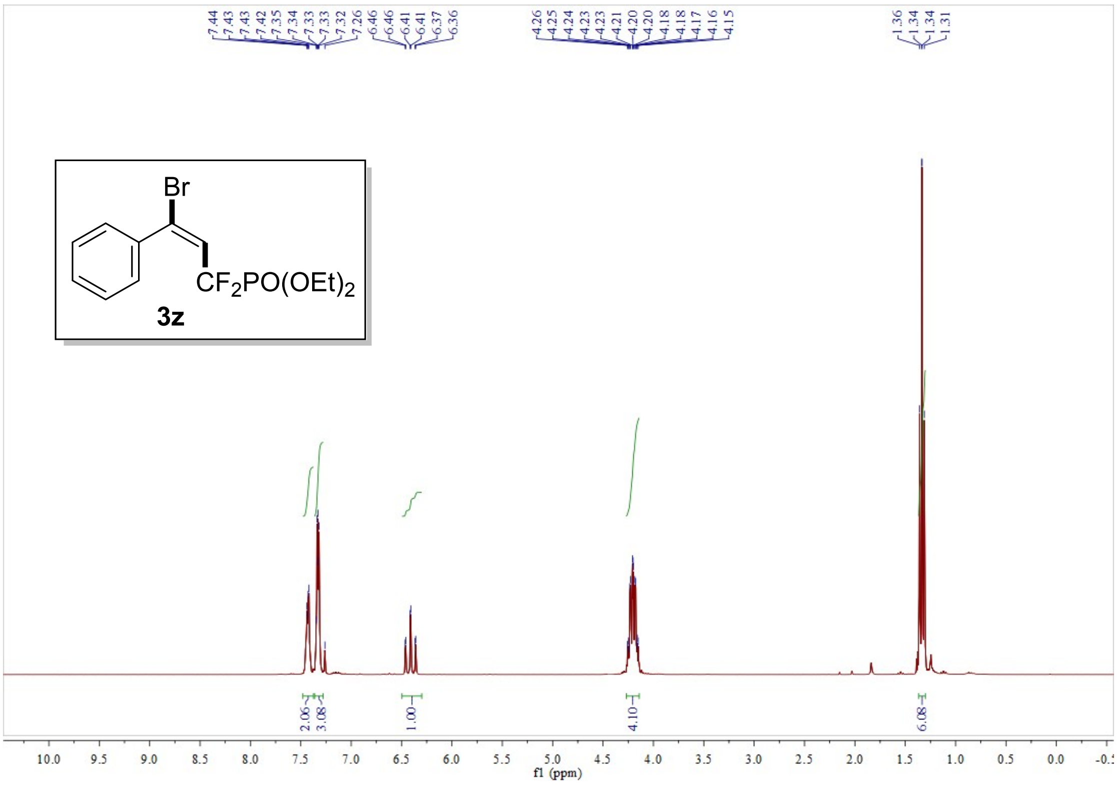
Br
CF2PO(OEt)2
3z
f1 (ppm)

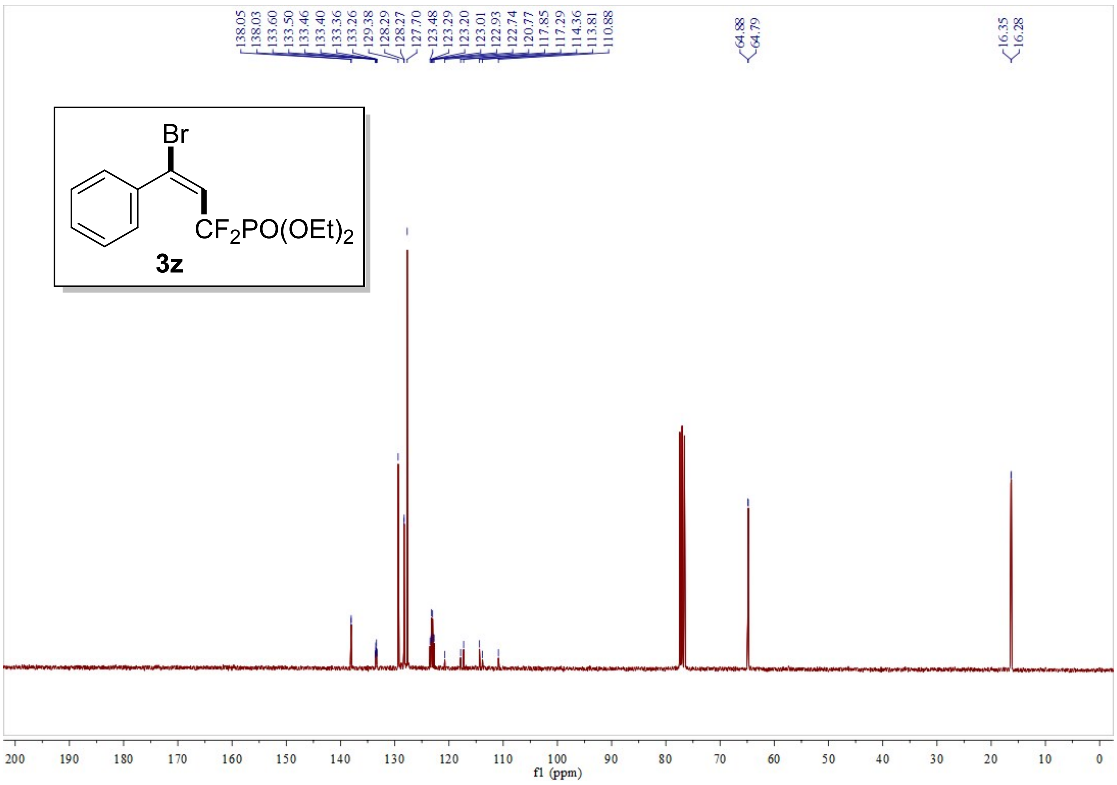
138.05
138.03
133.60
133.50
133.46
133.40
133.36
133.26
129.38
128.29
128.27
127.70
123.48
123.29
123.20
123.01
122.93
122.74
120.77
117.85
117.29
114.36
113.81
110.88
64.88
64.79
16.35
16.28
Br
CF2PO(OEt)2
3z
f1 (ppm)

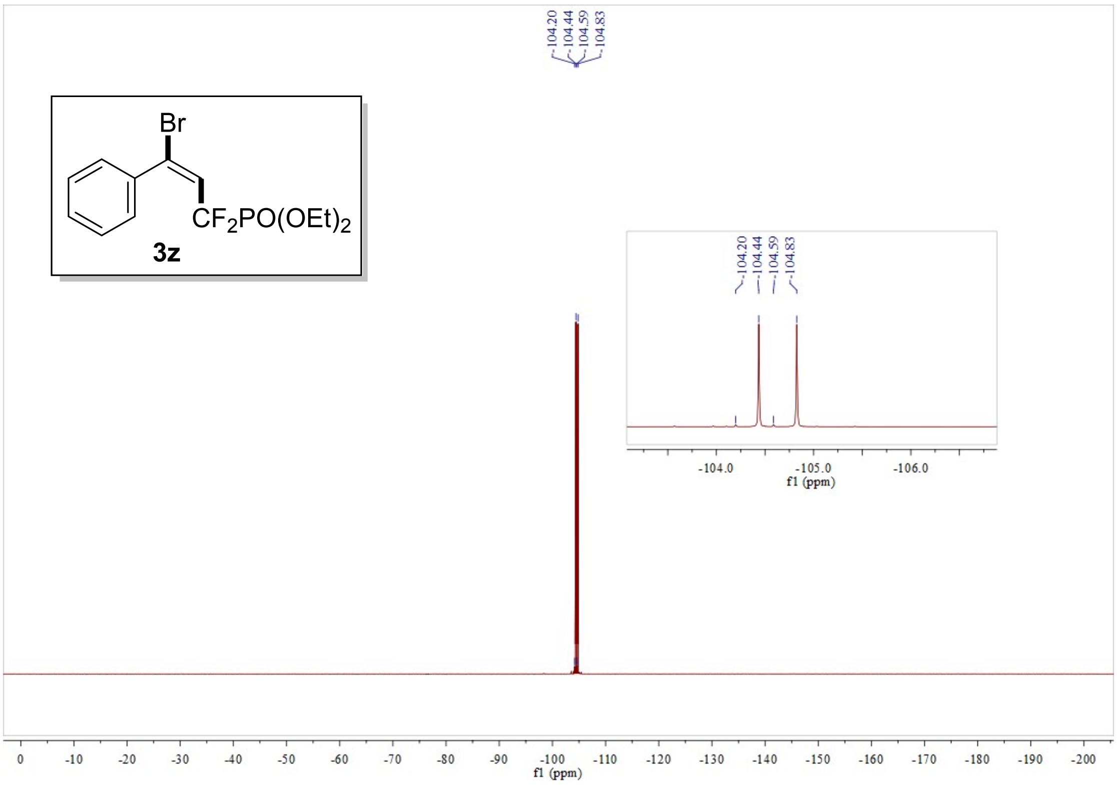
-104.20
-104.44
-104.59
-104.83
Br
CF2PO(OEt)2
3z
f1 (ppm)

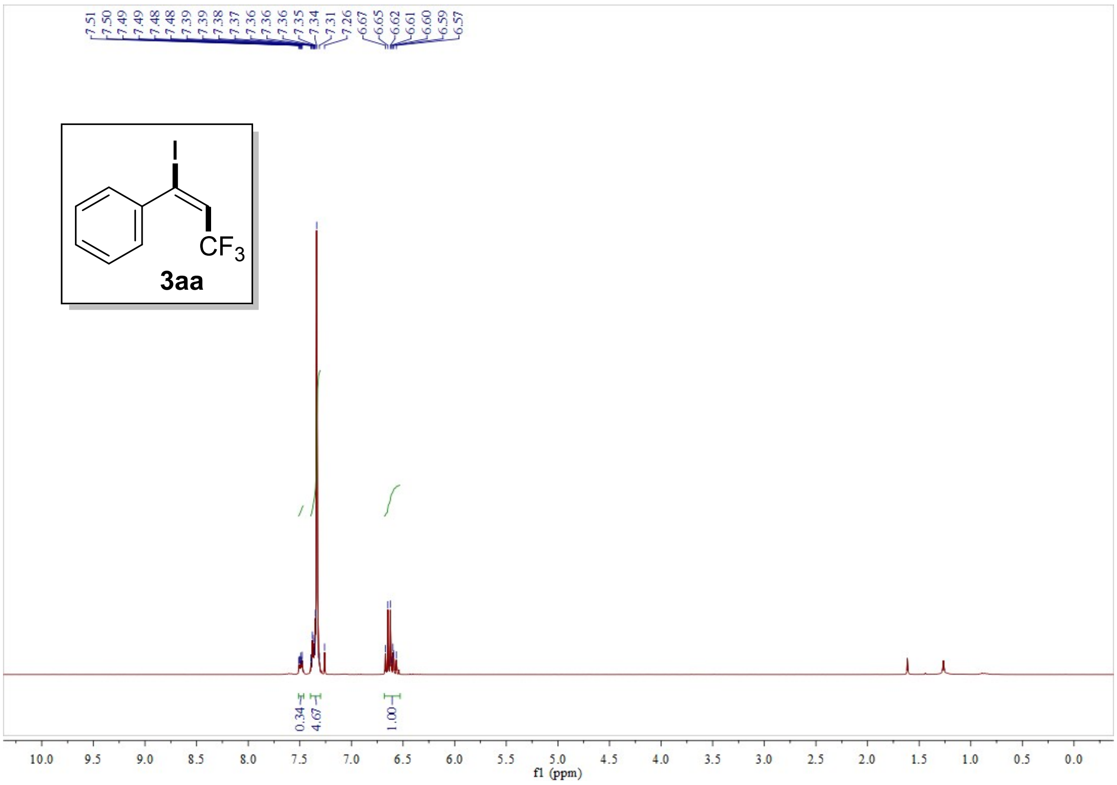
7.51
7.50
7.49
7.49
7.48
7.48
7.39
7.39
7.38
7.37
7.36
7.36
7.36
7.35
7.34
7.31
7.26
6.67
6.65
6.62
6.61
6.60
6.59
6.57
CF3
3aa
0.34
4.67
1.00
10.0 9.5 9.0 8.5 8.0 7.5 7.0 6.5 6.0 5.5 5.0 4.5 4.0 3.5 3.0 2.5 2.0 1.5 1.0 0.5 0.0
f1 (ppm)

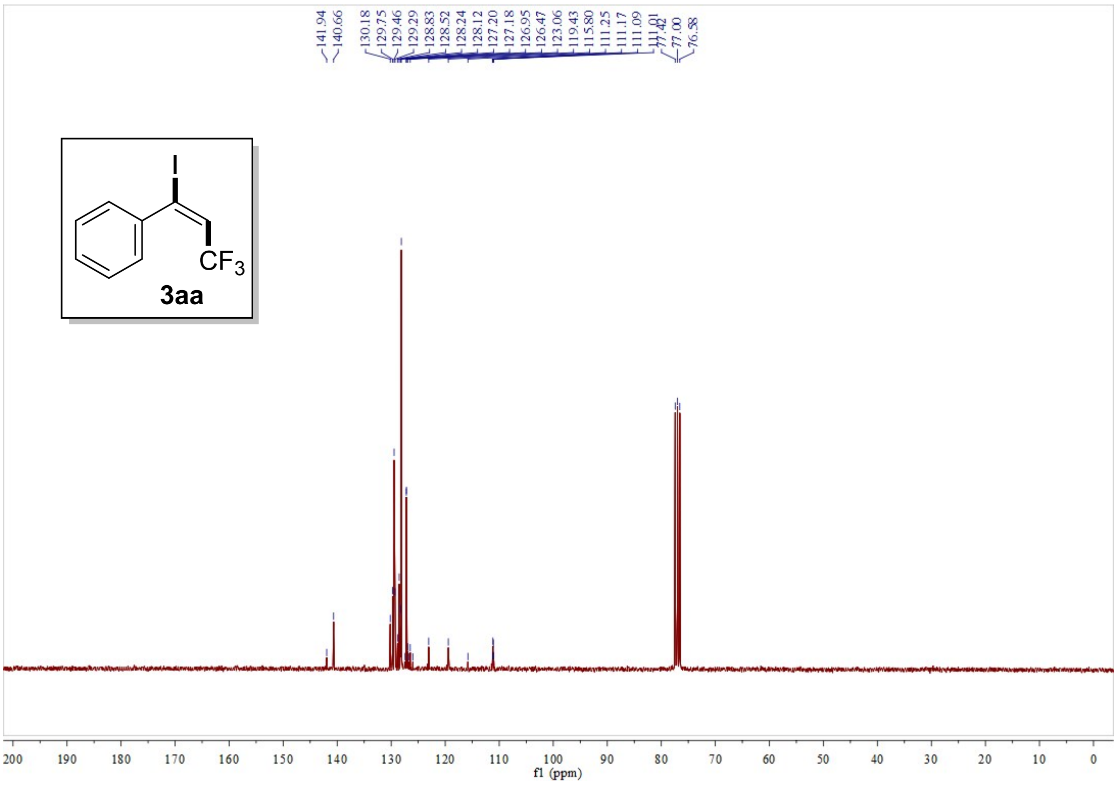
141.94
140.66
130.18
129.75
129.46
129.29
128.83
128.52
128.24
128.12
127.20
127.18
126.95
126.47
123.06
119.43
115.80
111.25
111.17
111.09
111.01
77.42
77.00
76.58
CF3
3aa
200 190 180 170 160 150 140 130 120 110 100 90 80 70 60 50 40 30 20 10 0
f1 (ppm)

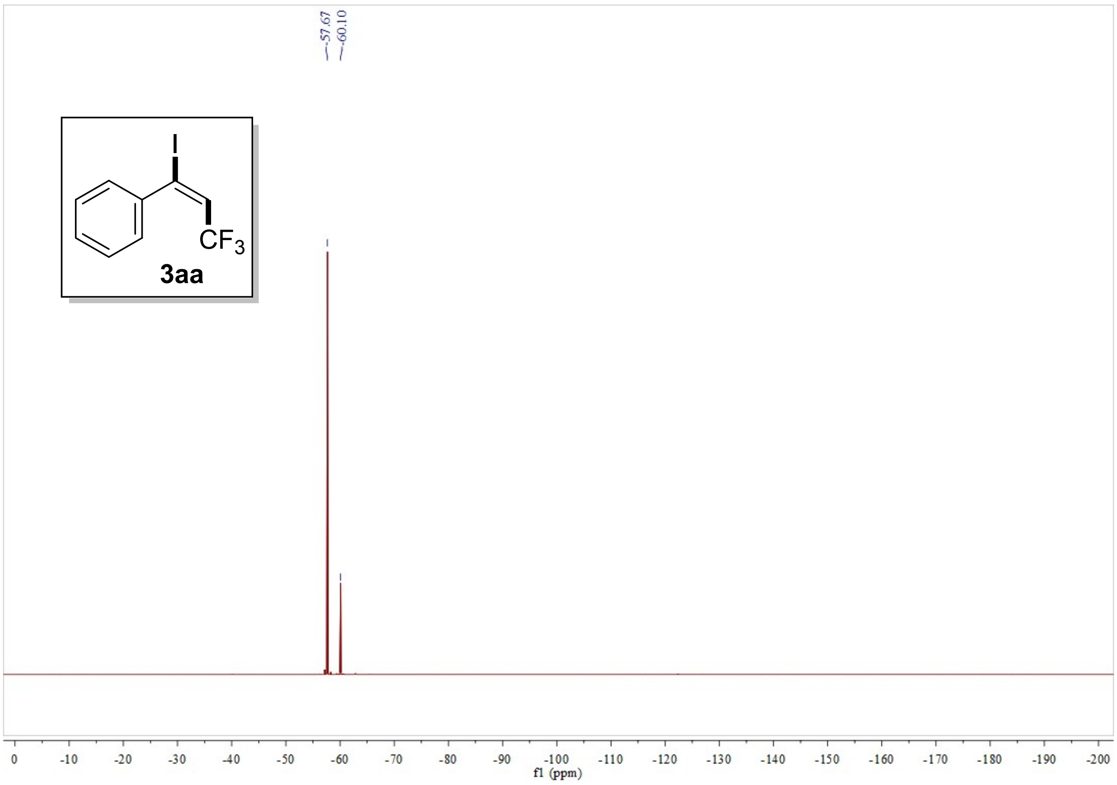
-57.67
-60.10
I
CF3
3aa
0 -10 -20 -30 -40 -50 -60 -70 -80 -90 -100 -110 -120 -130 -140 -150 -160 -170 -180 -190 -200
f1 (ppm)

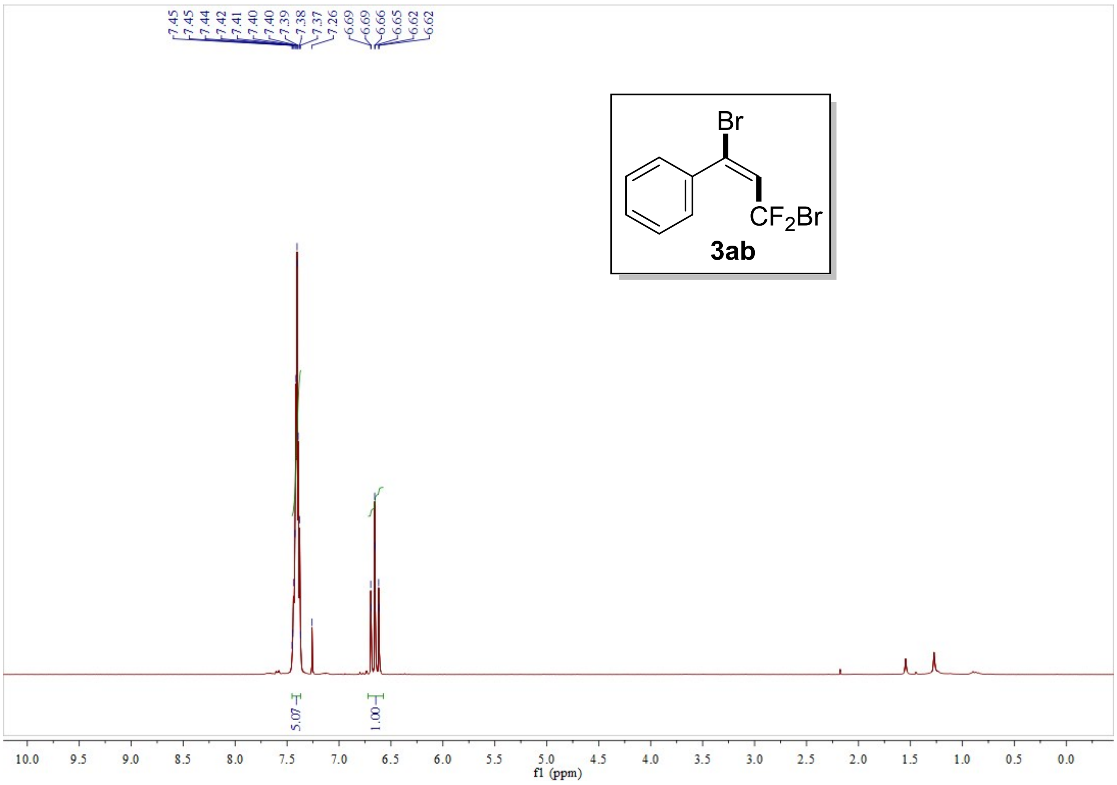
7.45 7.45 7.44 7.42 7.41 7.40 7.40 7.39 7.38 7.37 7.26 6.69 6.69 6.66 6.65 6.62 6.62
Br
CF2Br
3ab
5.07
1.00
10.0 9.5 9.0 8.5 8.0 7.5 7.0 6.5 6.0 5.5 5.0 4.5 4.0 3.5 3.0 2.5 2.0 1.5 1.0 0.5 0.0
f1 (ppm)

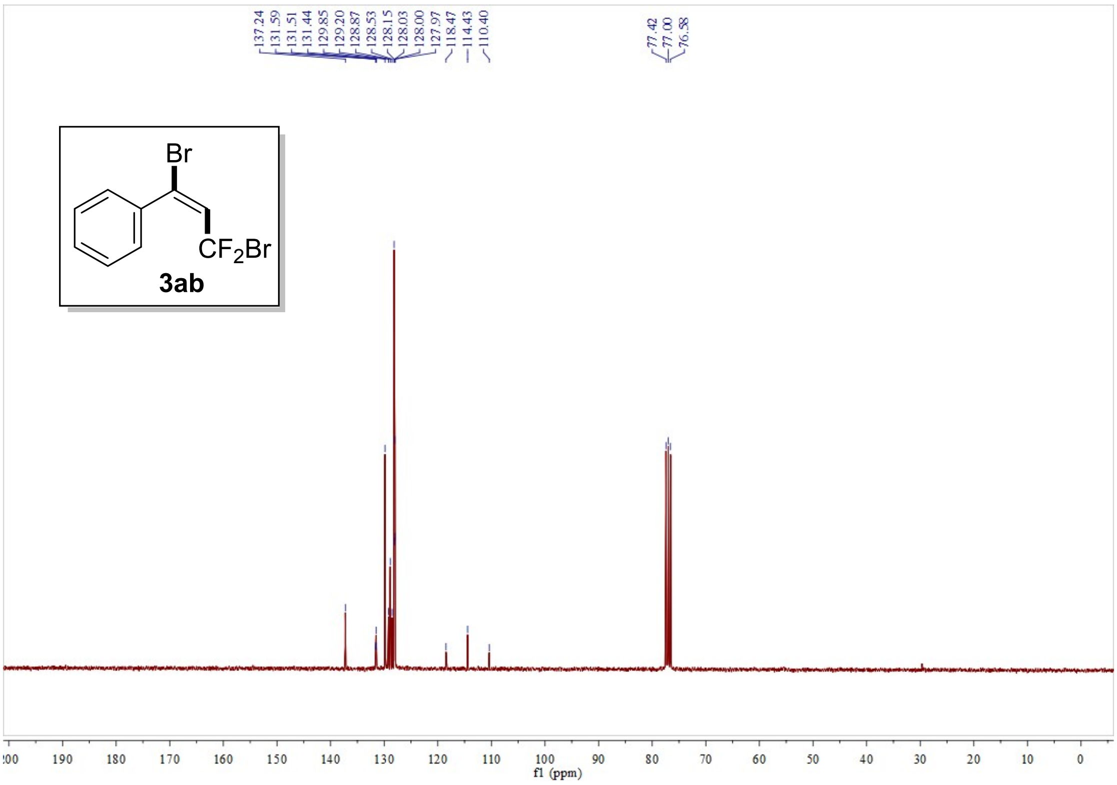
137.24
131.59
131.51
131.44
129.85
129.20
128.87
128.53
128.15
128.03
128.00
127.97
118.47
114.43
110.40
77.42
77.00
76.58
Br
CF2Br
3ab
f1 (ppm)

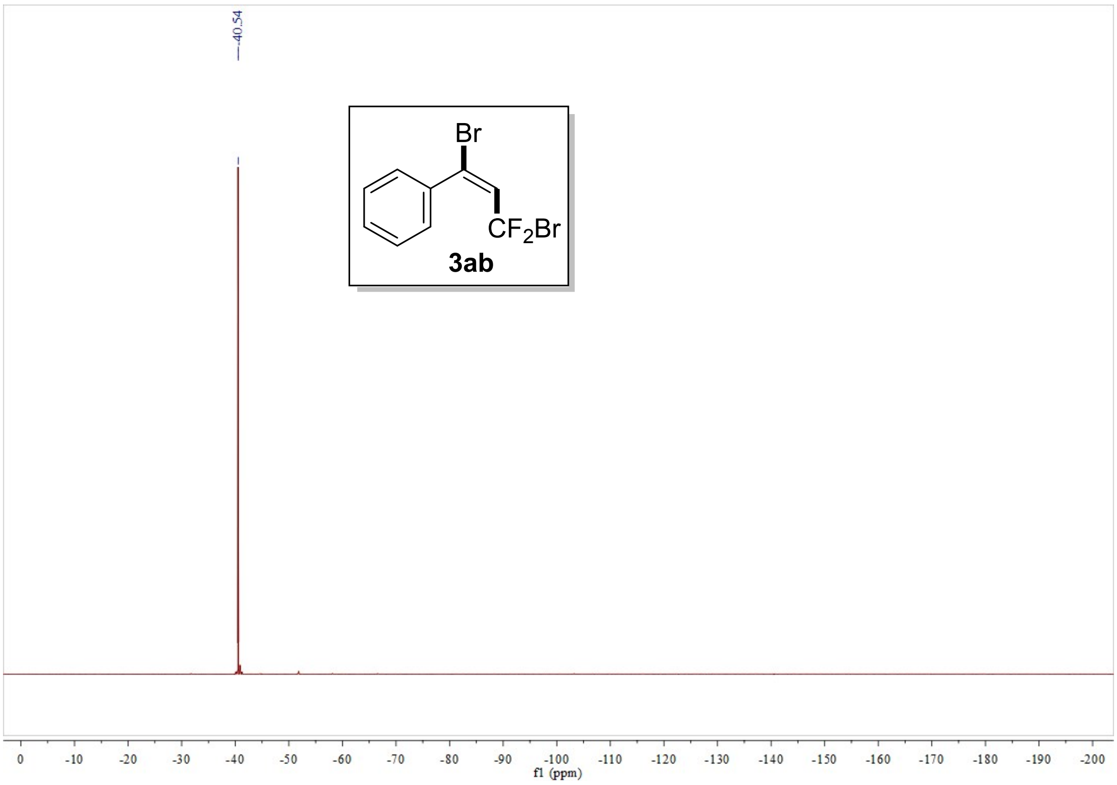
-40.54
Br
CF2Br
3ab
f1 (ppm)

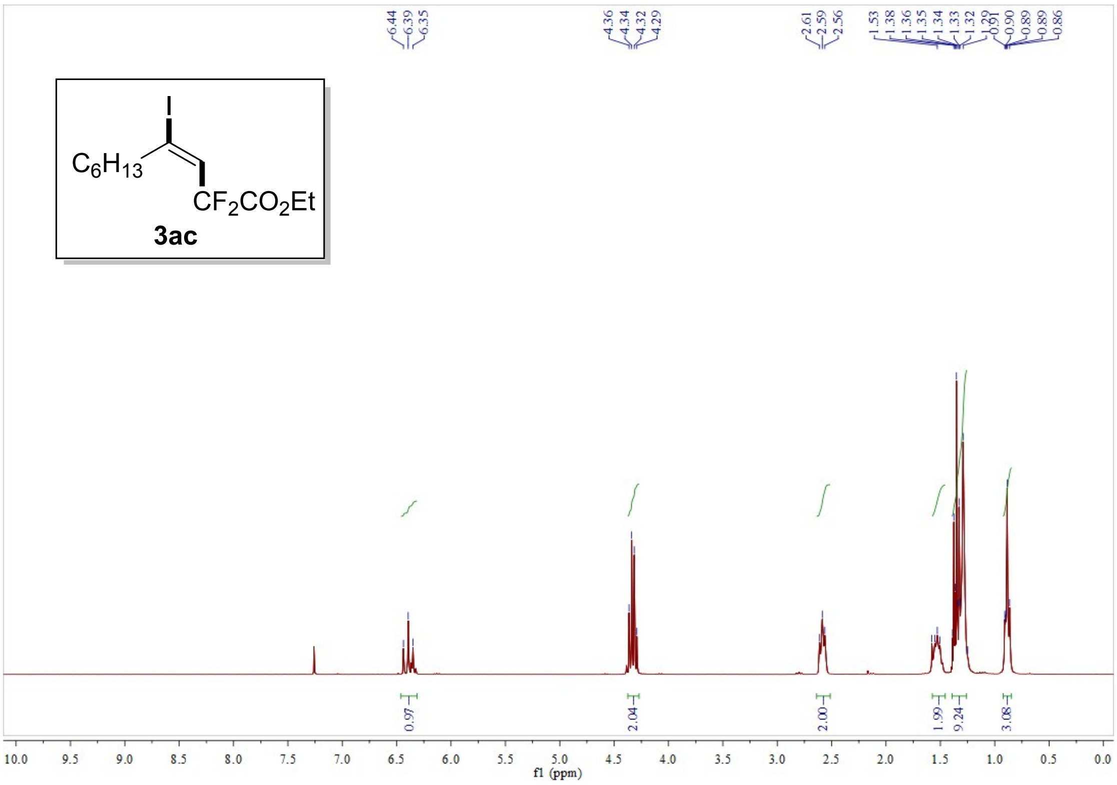
6.44
6.39
6.35
4.36
4.34
4.32
4.29
2.61
2.59
2.56
1.53
1.38
1.36
1.35
1.34
1.33
1.32
1.29
0.91
0.90
0.89
0.89
0.86
I
C6H13
CF2CO2Et
3ac
0.97
2.04
2.00
1.99
9.24
3.08
10.0 9.5 9.0 8.5 8.0 7.5 7.0 6.5 6.0 5.5 5.0 4.5 4.0 3.5 3.0 2.5 2.0 1.5 1.0 0.5 0.0
f1 (ppm)

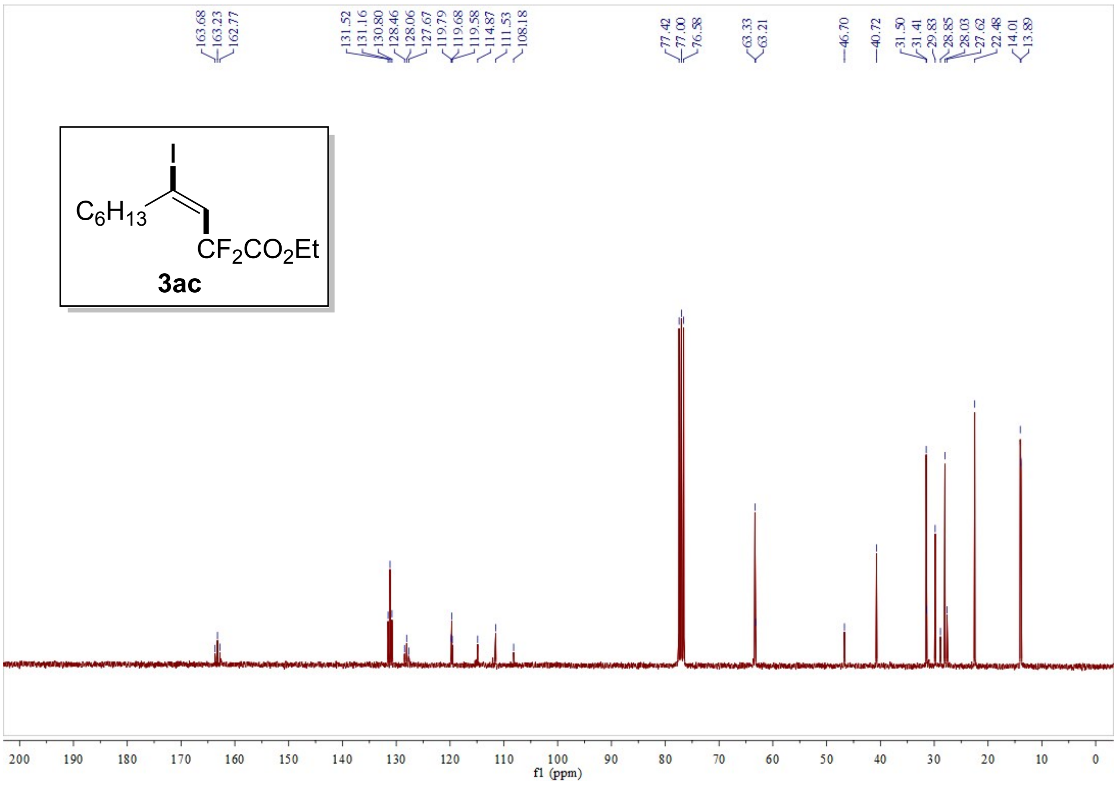
163.68
163.23
162.77
131.52
131.16
130.80
128.46
128.06
127.67
119.79
119.68
119.58
114.87
111.53
108.18
77.42
77.00
76.58
63.33
63.21
46.70
40.72
31.50
31.41
29.83
28.85
28.03
27.62
22.48
14.01
13.89
I
C6H13
CF2CO2Et
3ac
200 190 180 170 160 150 140 130 120 110 100 90 80 70 60 50 40 30 20 10 0
f1 (ppm)

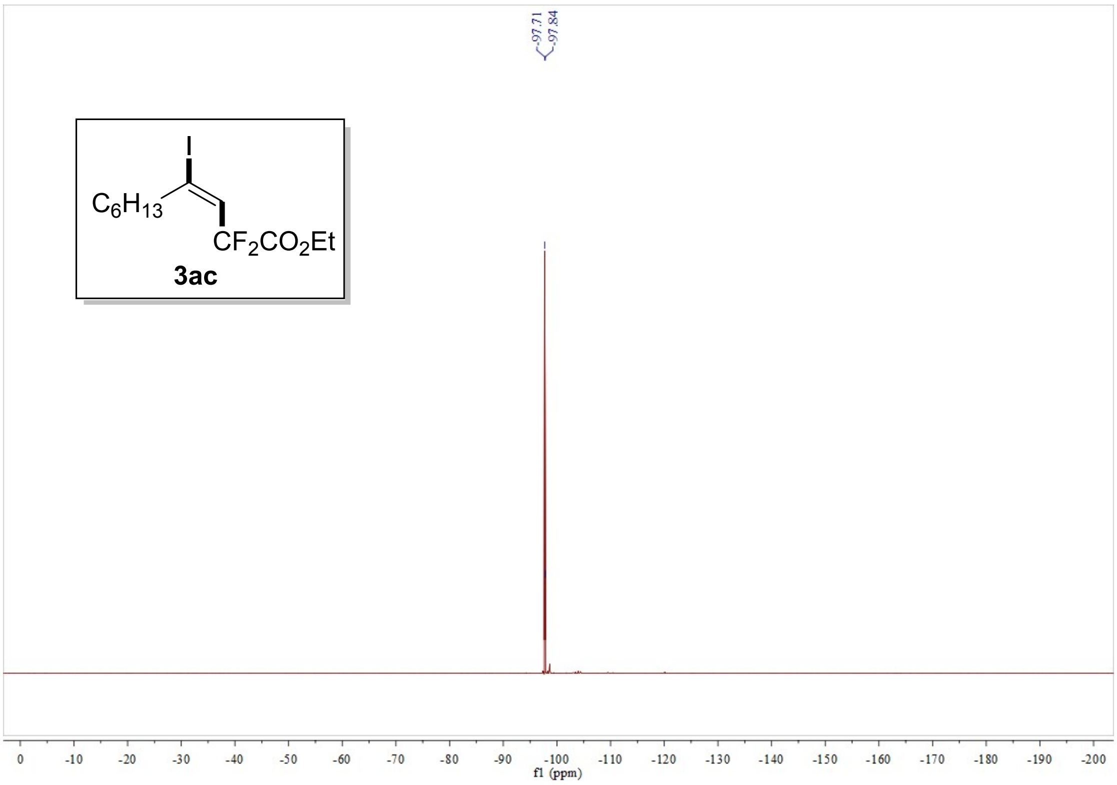
-97.71
-97.84
C6H13
I
CF2CO2Et
3ac
f1 (ppm)

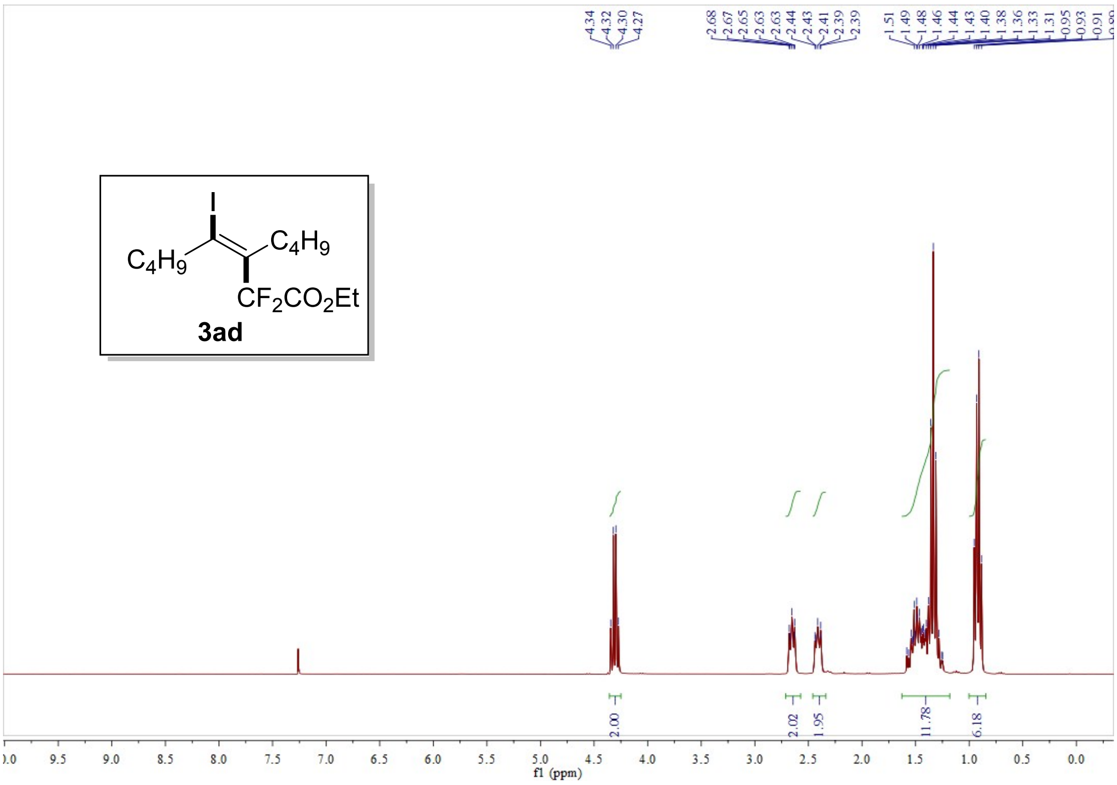
4.34
4.32
4.30
4.27
2.68
2.67
2.65
2.63
2.63
2.44
2.43
2.41
2.39
2.39
1.51
1.49
1.48
1.46
1.44
1.43
1.40
1.38
1.36
1.33
1.31
0.95
0.93
0.91
I
C4H9
C4H9
CF2CO2Et
3ad
2.00
2.02
1.95
11.78
6.18
f1 (ppm)

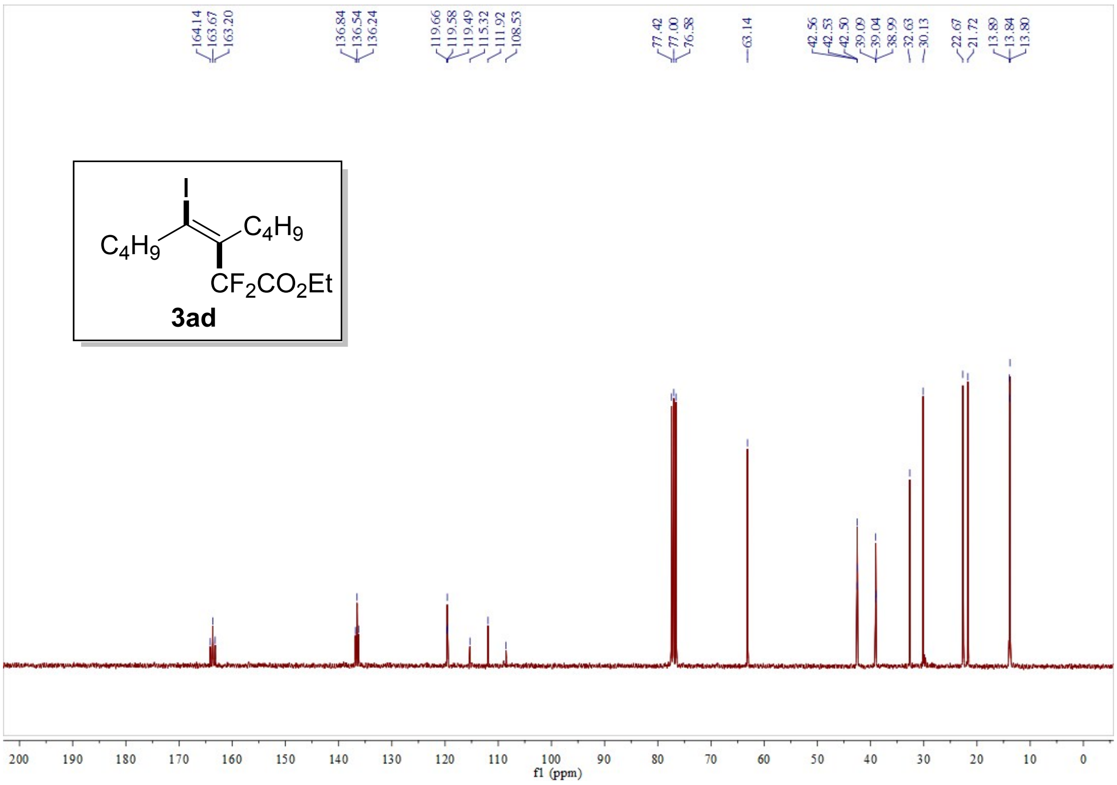
164.14
163.67
163.20
136.84
136.54
136.24
119.66
119.58
119.49
115.32
111.92
108.53
77.42
77.00
76.58
63.14
42.56
42.53
42.50
39.09
39.04
38.99
32.63
30.13
22.67
21.72
13.89
13.84
13.80
3ad
f1 (ppm)

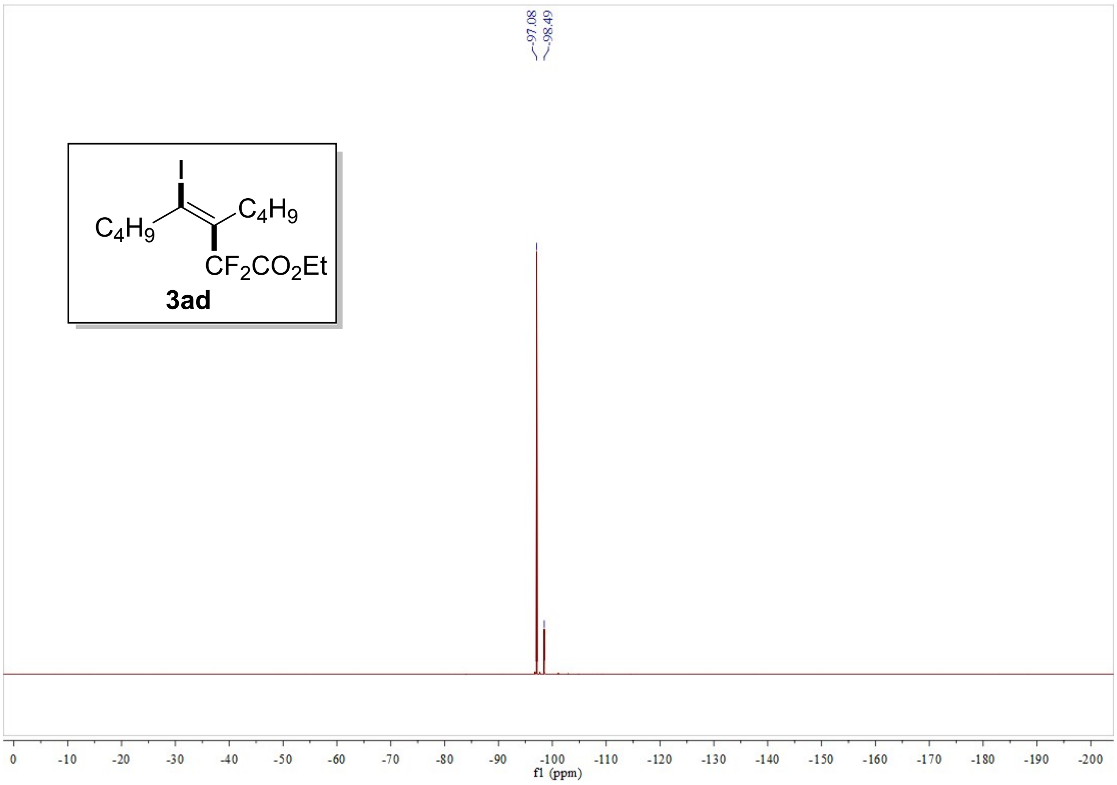
-97.08
-98.49
3ad
f1 (ppm)

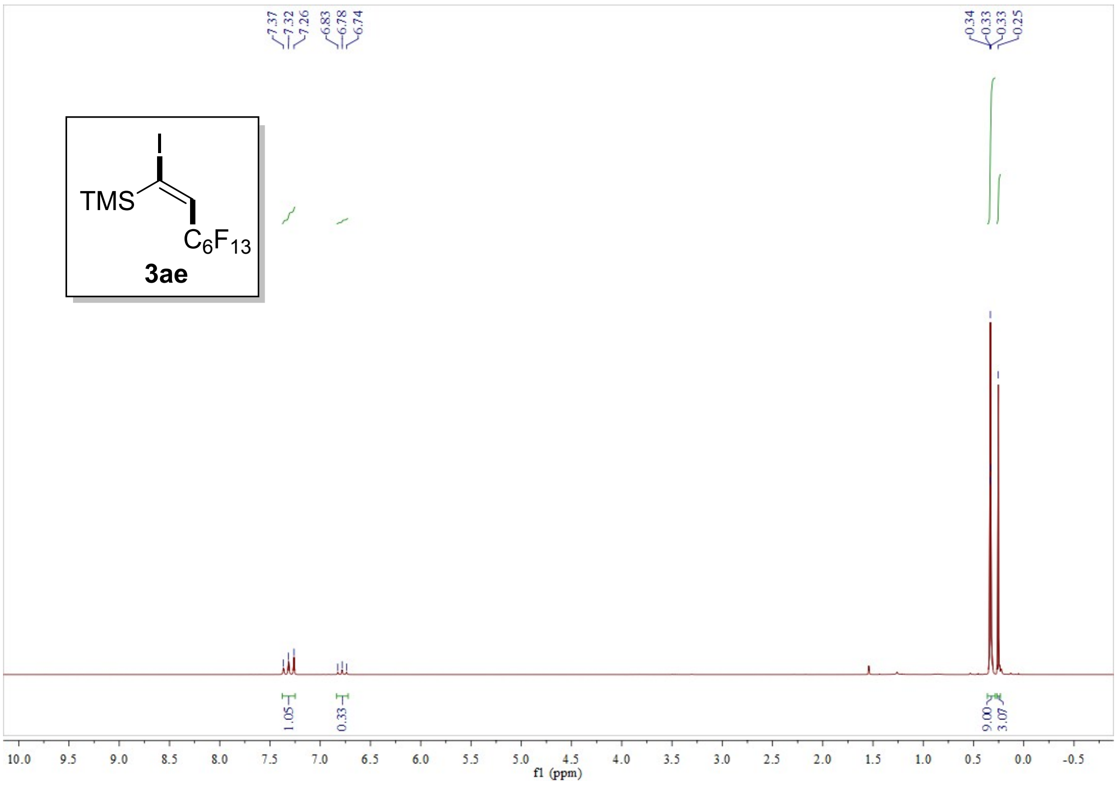
TMS
I
C_6F_{13}
3ae
f1 (ppm)

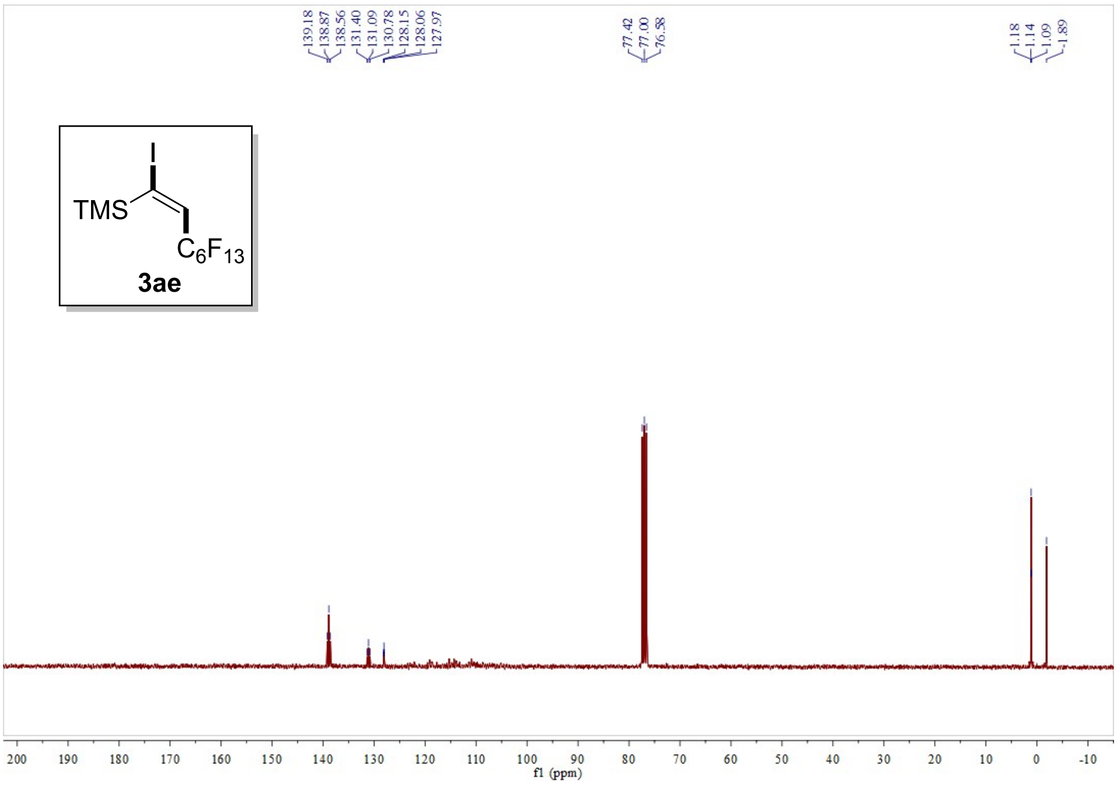
TMS
I
C_6F_{13}
3ae
f1 (ppm)

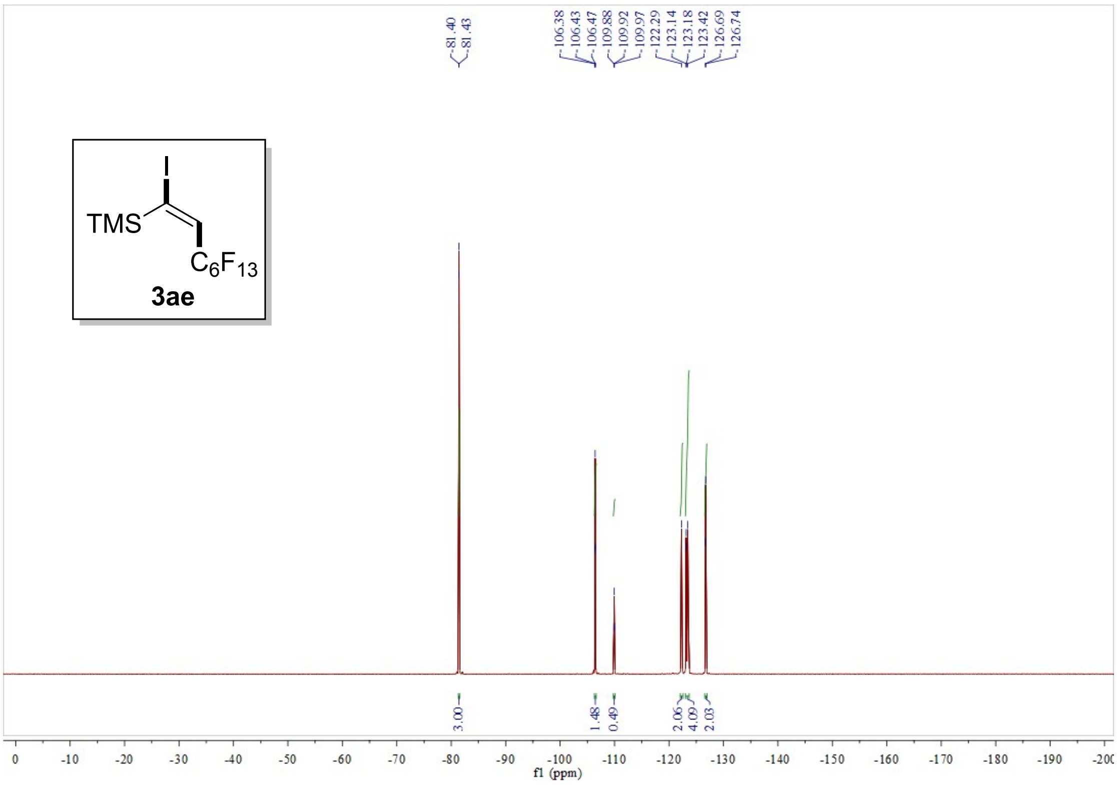
TMS
I
C_6F_{13}
3ae
f1 (ppm)

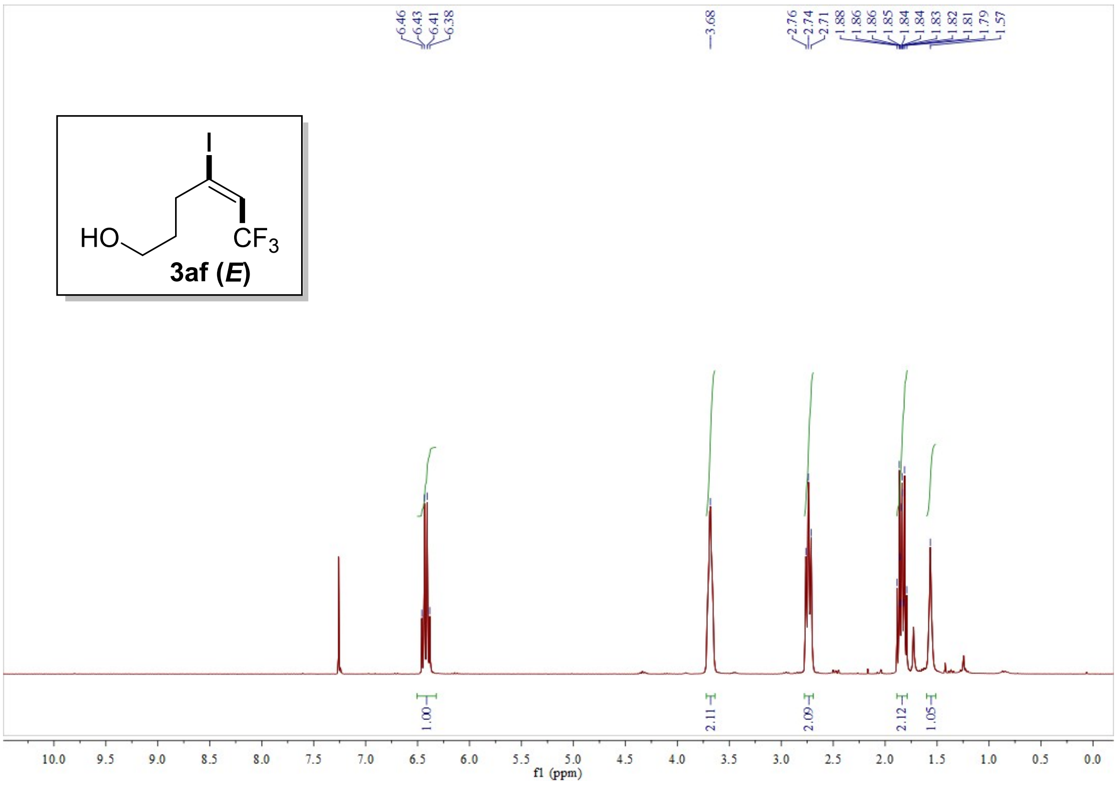
HO
I
CF_3
3af (E)
f1 (ppm)

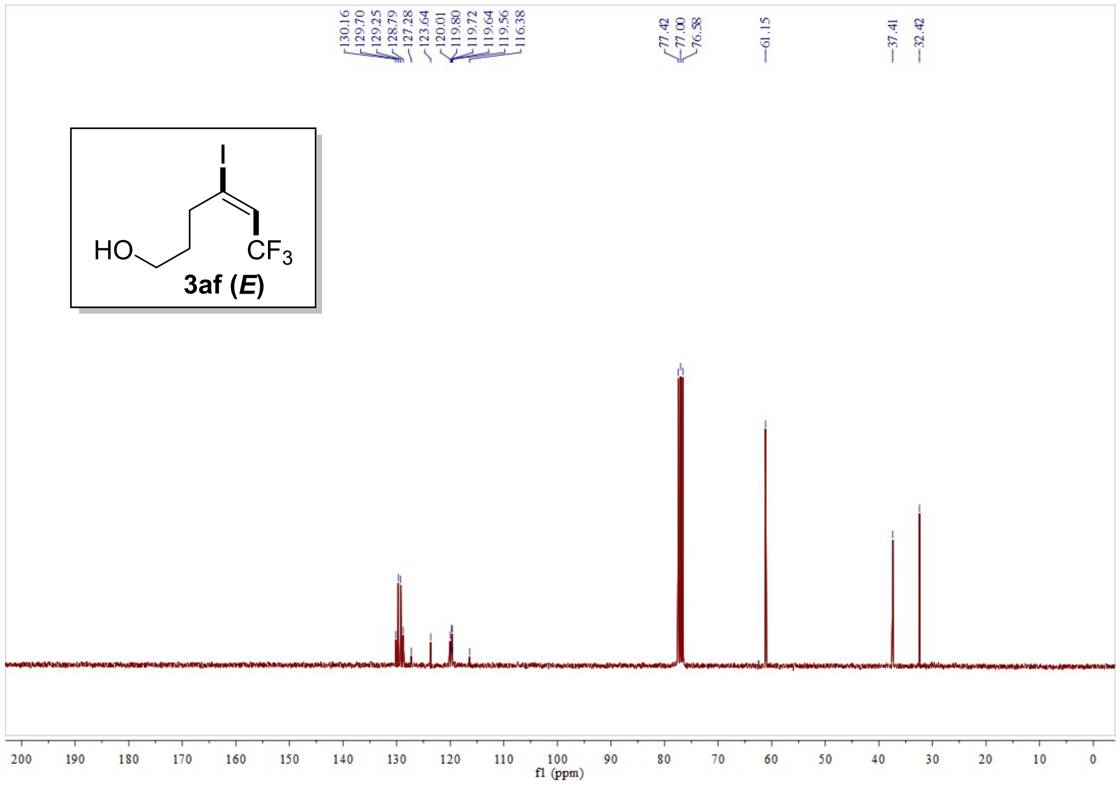
130.16
129.70
129.25
128.79
127.28
123.64
120.01
119.80
119.72
119.64
119.56
116.38
77.42
77.00
76.58
61.15
37.41
32.42
HO
I
CF3
3af (E)
f1 (ppm)

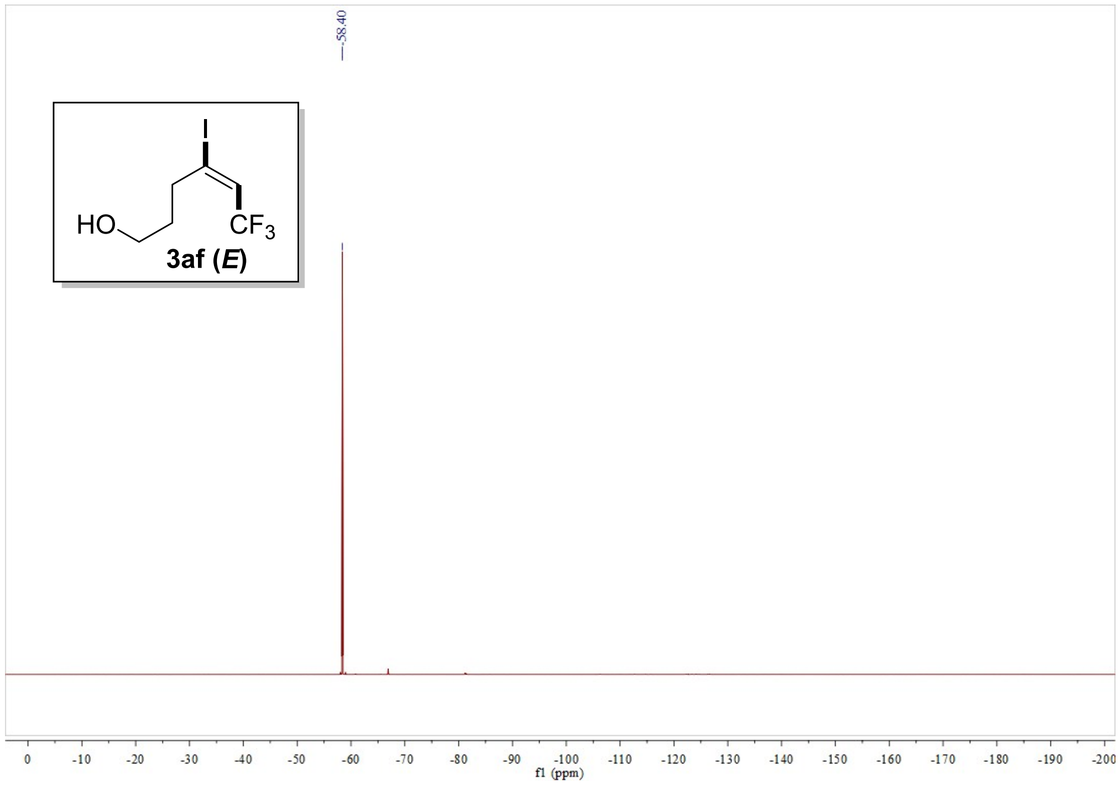
-58.40
HO
I
CF3
3af (E)
f1 (ppm)

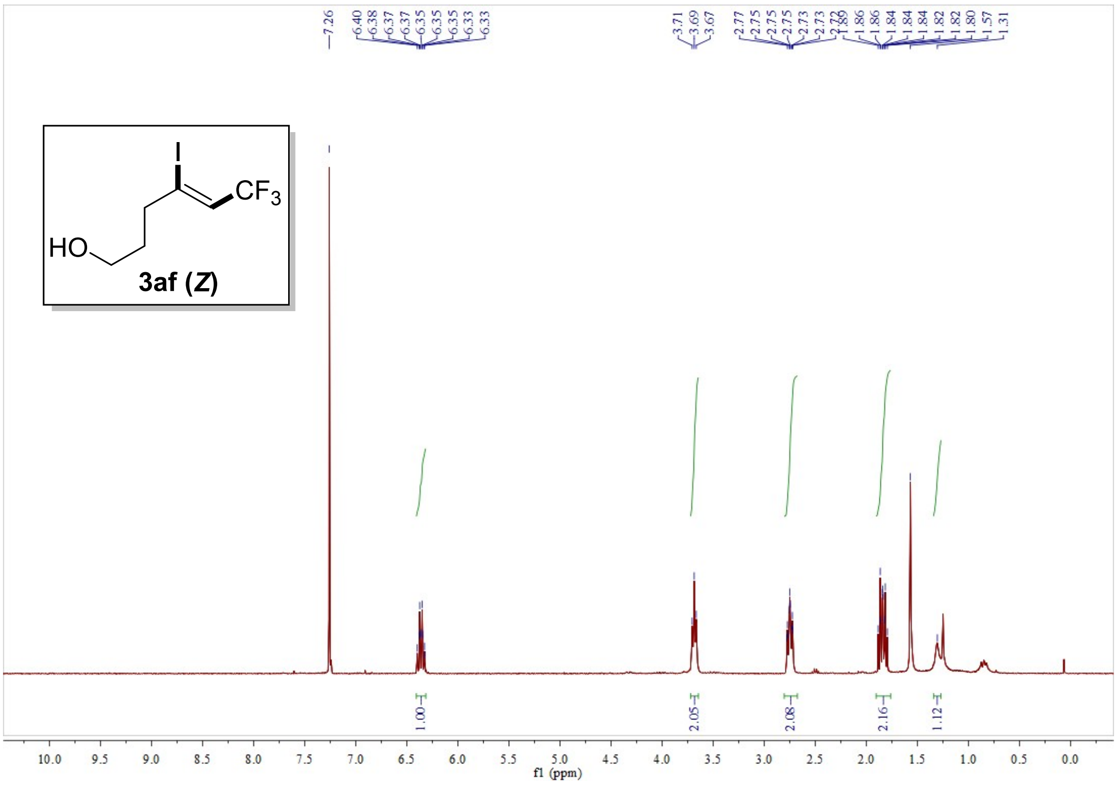

3af (Z)
HO
CF3
I
f1 (ppm)

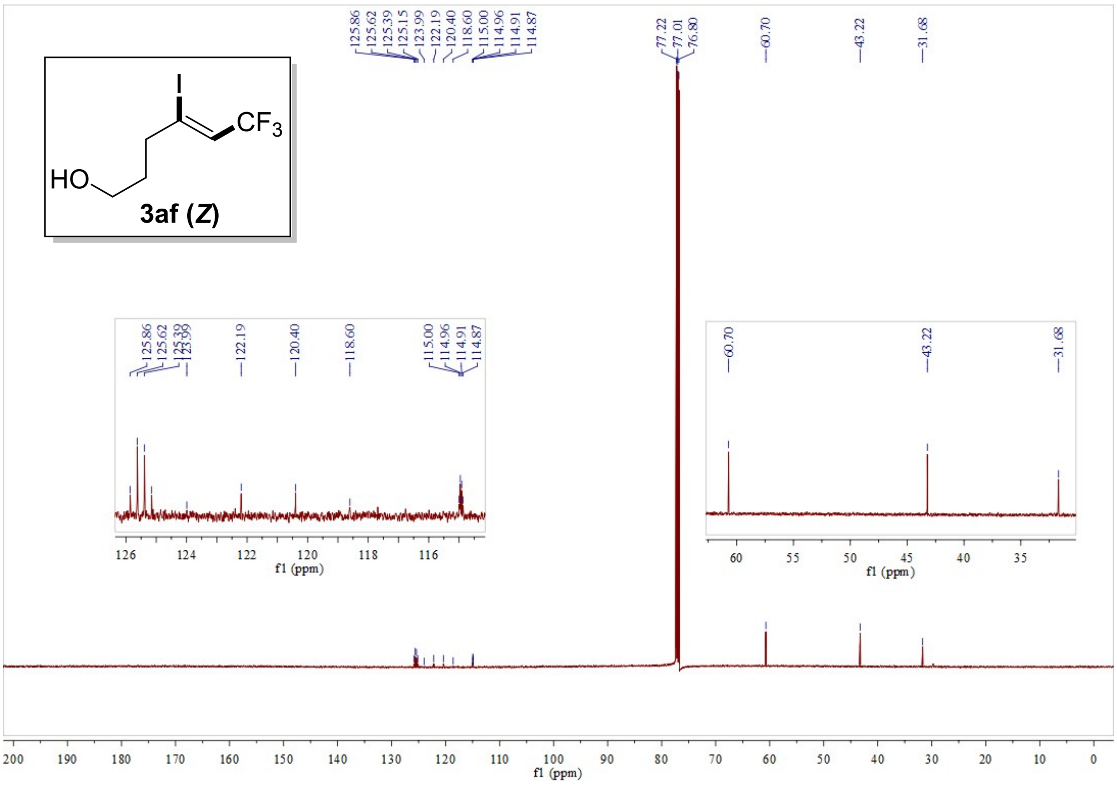

3af (Z)
HO
CF3
I
f1 (ppm)

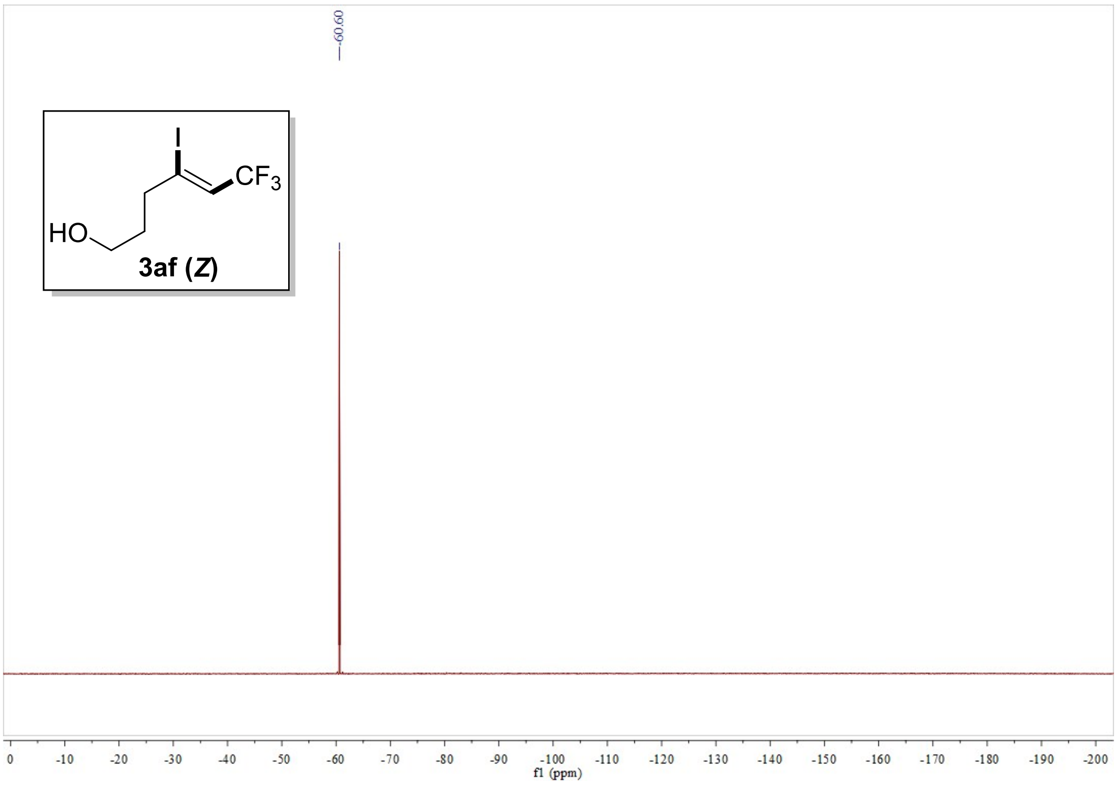

-60.60
HO
I
CF3
3af (Z)
f1 (ppm)

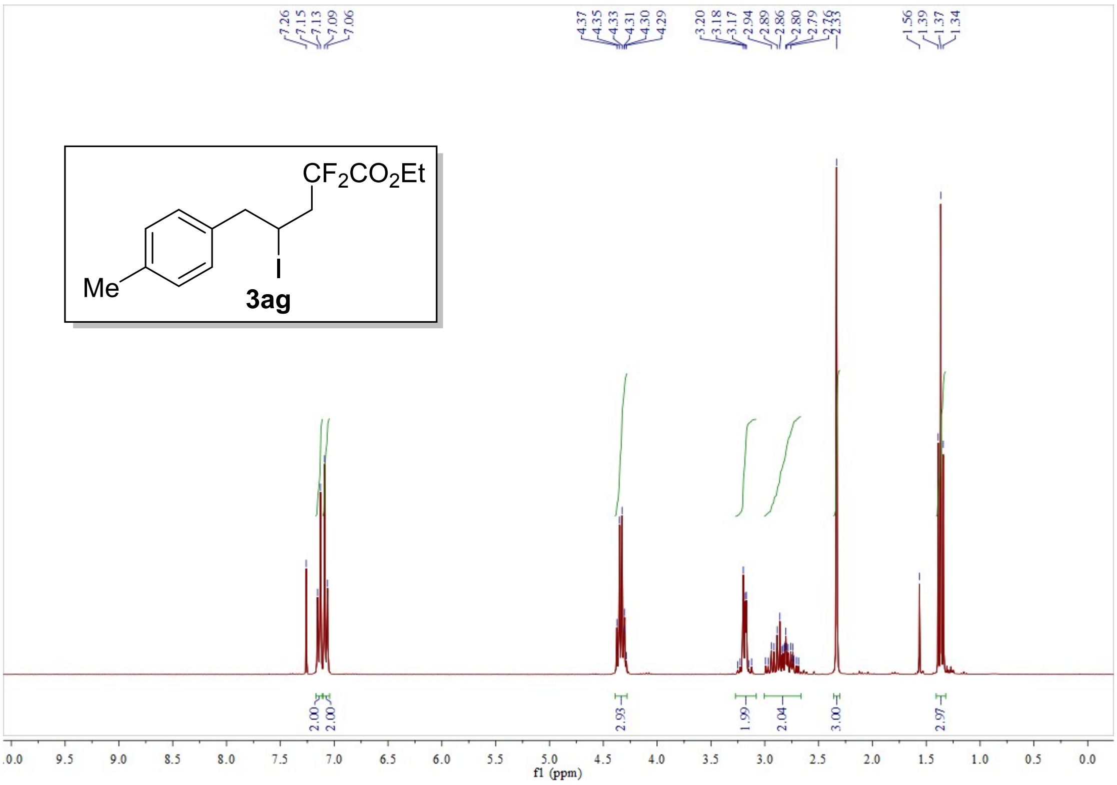

7.26
7.15
7.13
7.09
7.06
4.37
4.35
4.33
4.31
4.30
4.29
3.20
3.18
3.17
2.94
2.89
2.86
2.80
2.79
2.36
2.33
1.56
1.39
1.37
1.34
CF2CO2Et
Me
I
3ag
2.00
2.00
2.93
1.99
2.04
3.00
2.97
f1 (ppm)

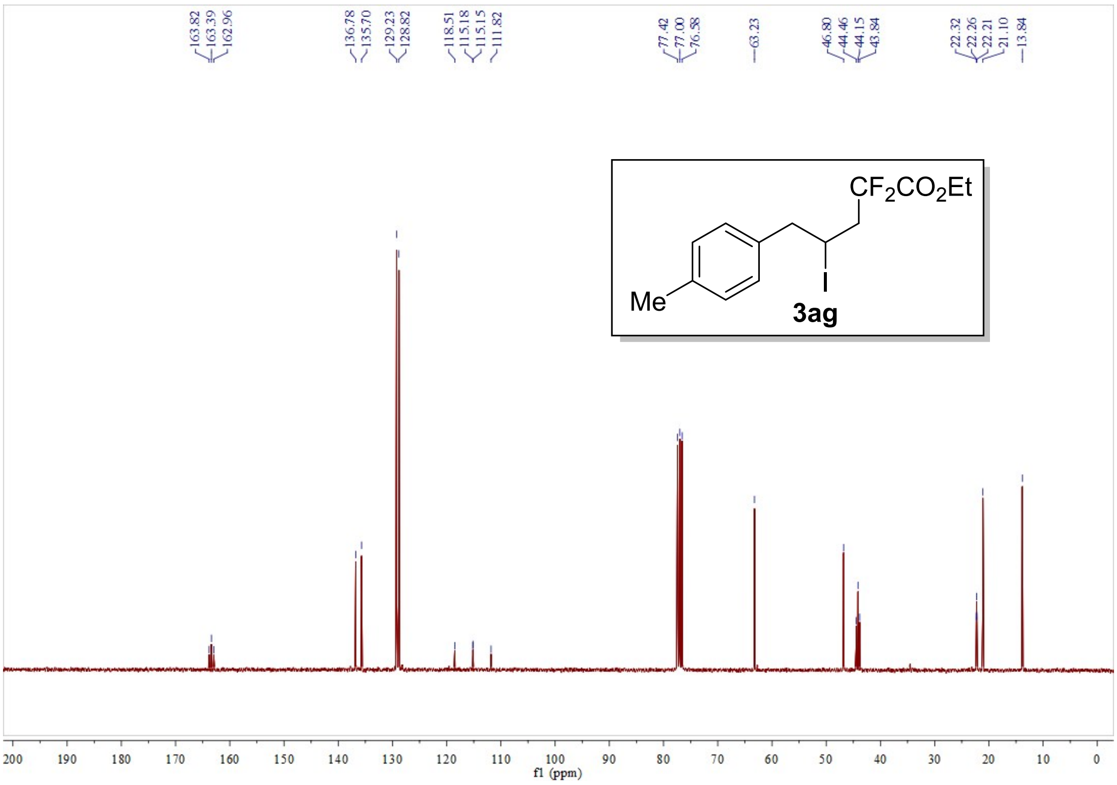
163.82
163.39
162.96
136.78
135.70
129.23
128.82
118.51
115.18
115.15
111.82
77.42
77.00
76.58
63.23
46.80
44.46
44.15
43.84
22.32
22.26
22.21
21.10
13.84
CF2CO2Et
Me
I
3ag
200 190 180 170 160 150 140 130 120 110 100 90 80 70 60 50 40 30 20 10 0
f1 (ppm)

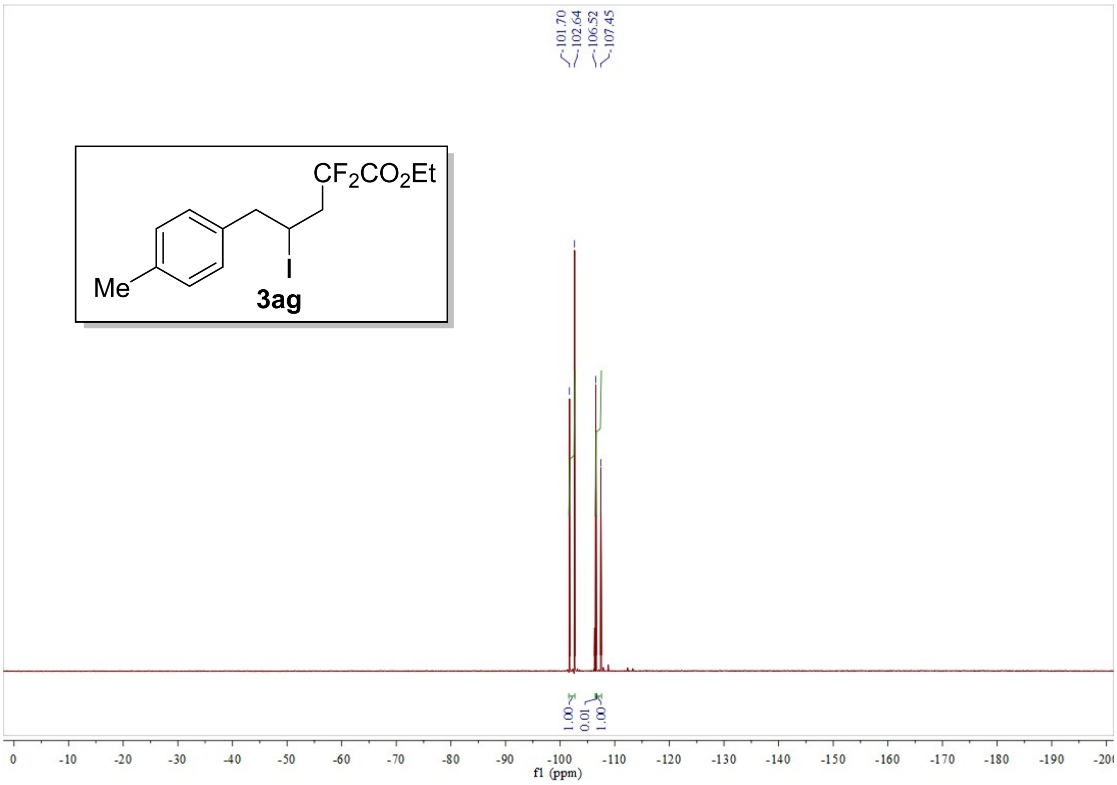
-101.70
-102.64
-106.52
-107.45
CF2CO2Et
Me
I
3ag
1.00
0.01
1.00
0 -10 -20 -30 -40 -50 -60 -70 -80 -90 -100 -110 -120 -130 -140 -150 -160 -170 -180 -190 -200
f1 (ppm)

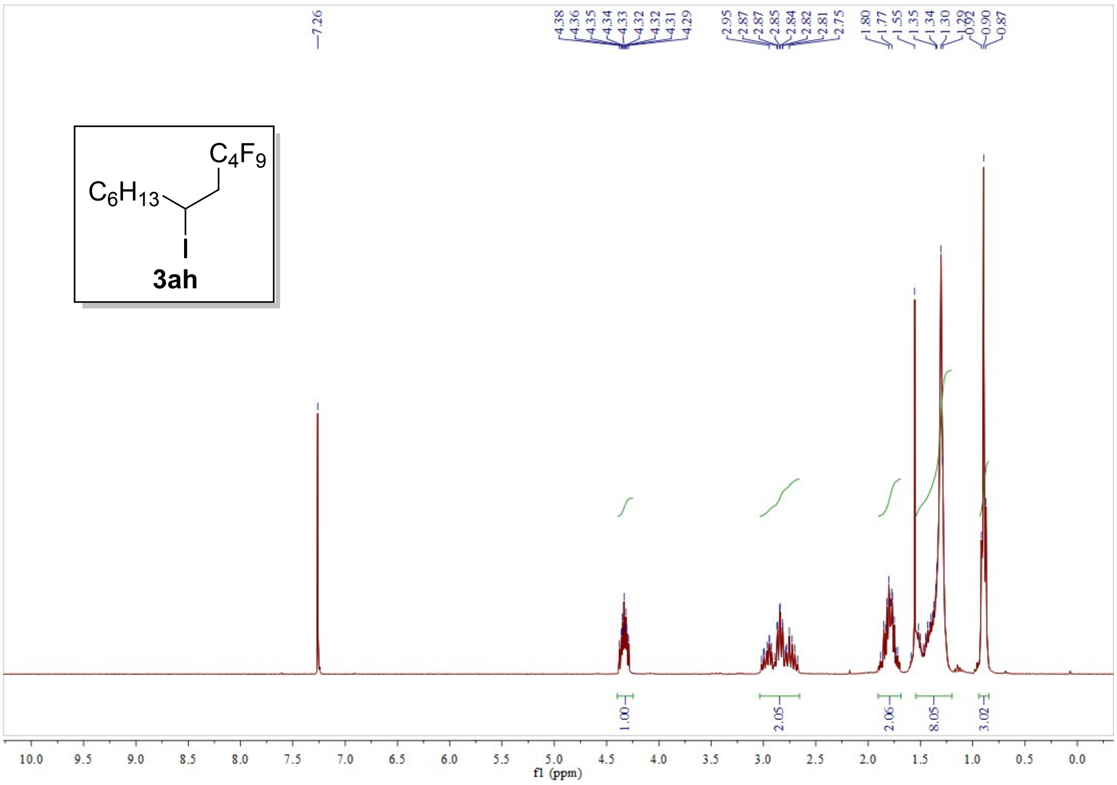

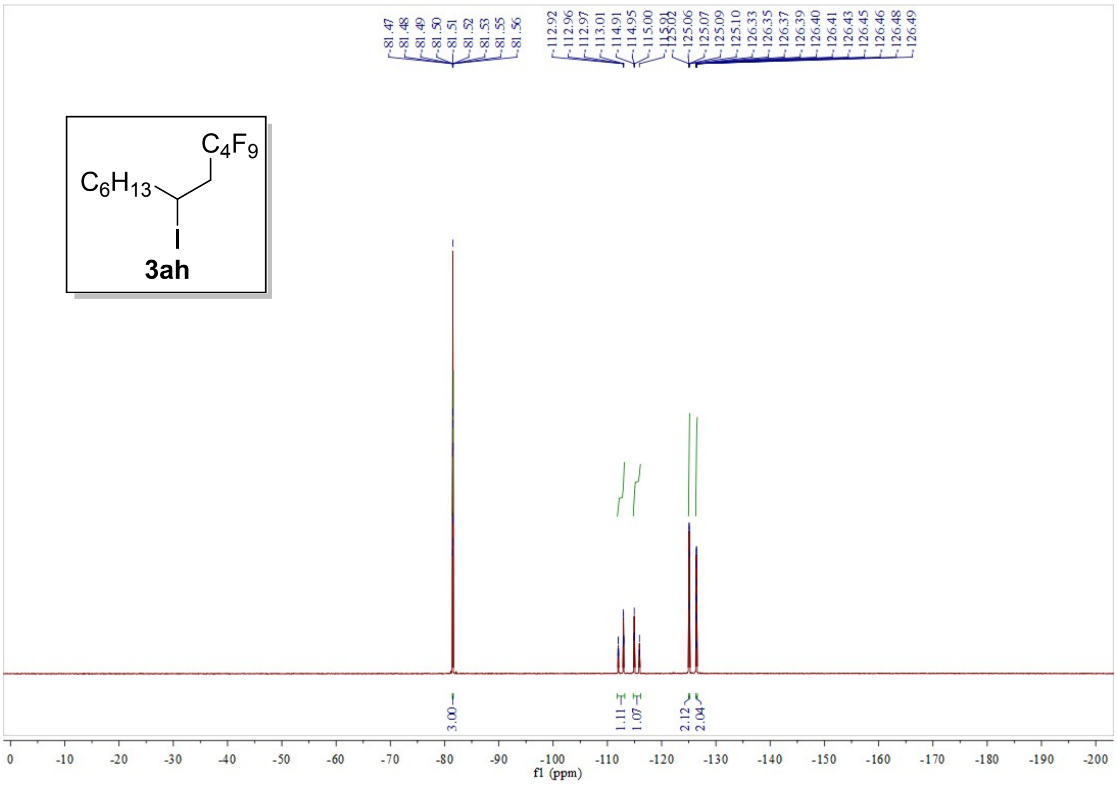

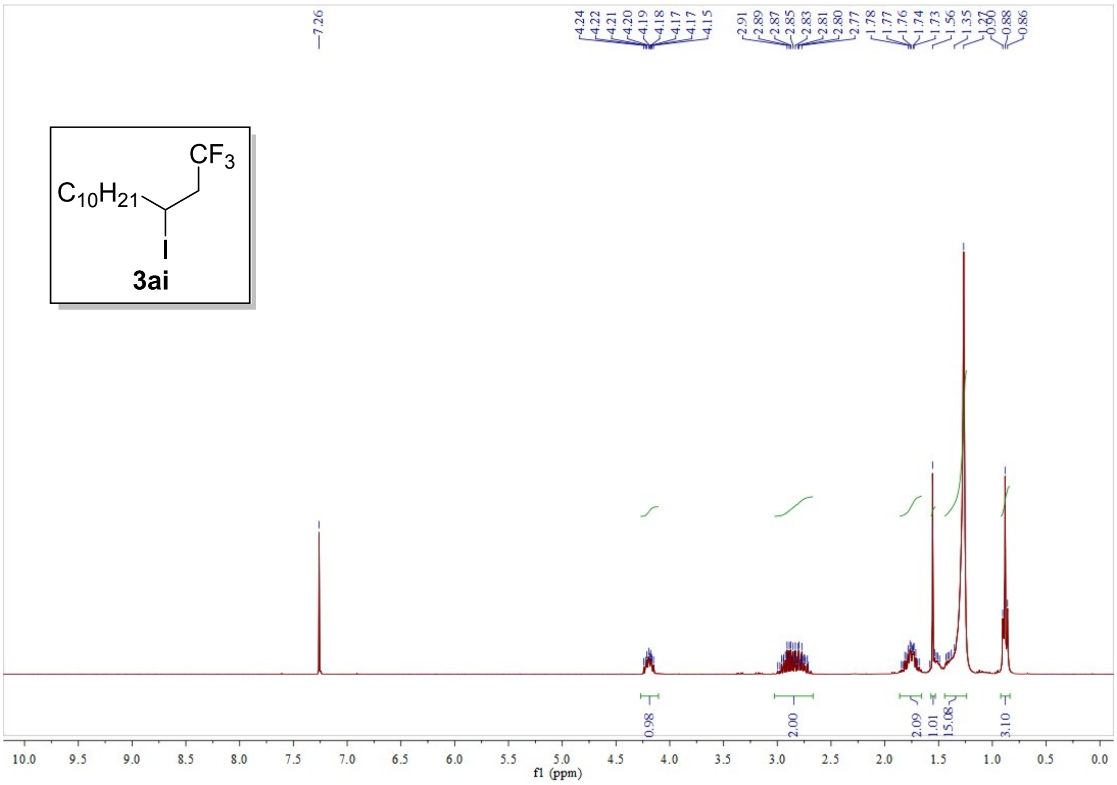
7.26
4.24
4.22
4.21
4.20
4.19
4.18
4.17
4.17
4.15
2.91
2.89
2.87
2.85
2.83
2.81
2.80
2.77
1.78
1.77
1.76
1.74
1.73
1.56
1.35
1.27
0.90
0.88
0.86
CF3
C10H21
I
3ai
0.98
2.00
2.09
1.01
15.08
3.10
f1 (ppm)

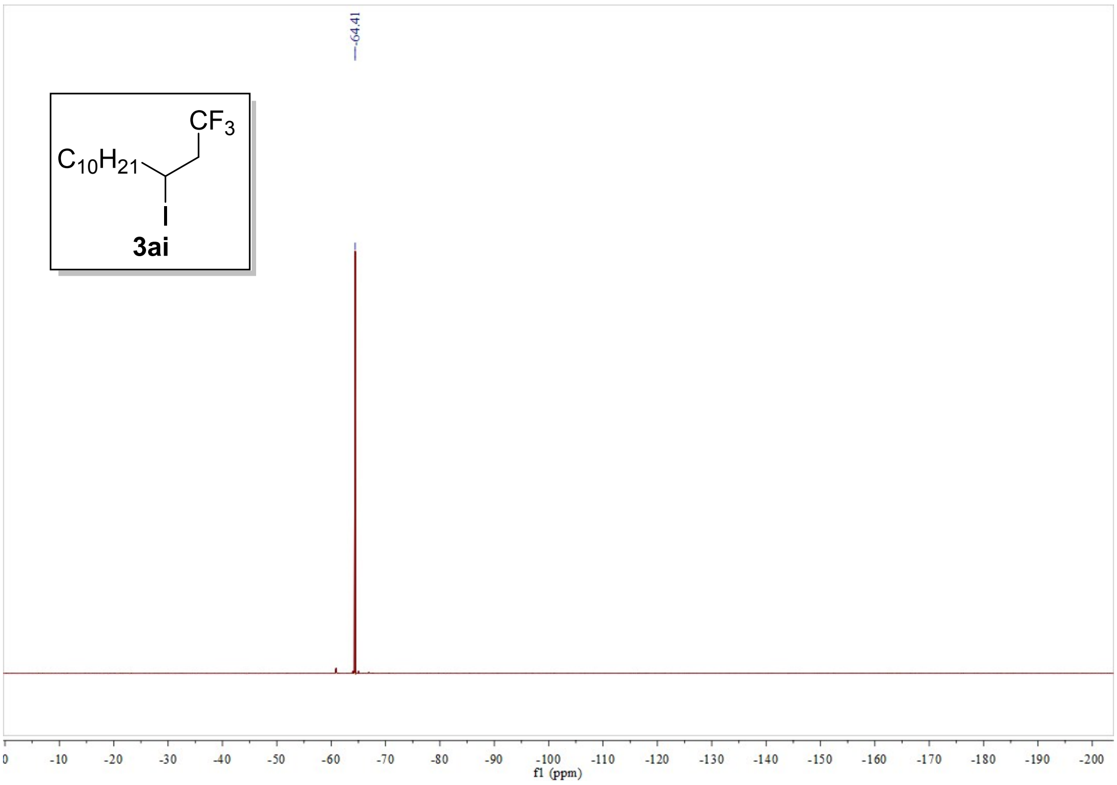
-64.41
CF3
C10H21
I
3ai
f1 (ppm)

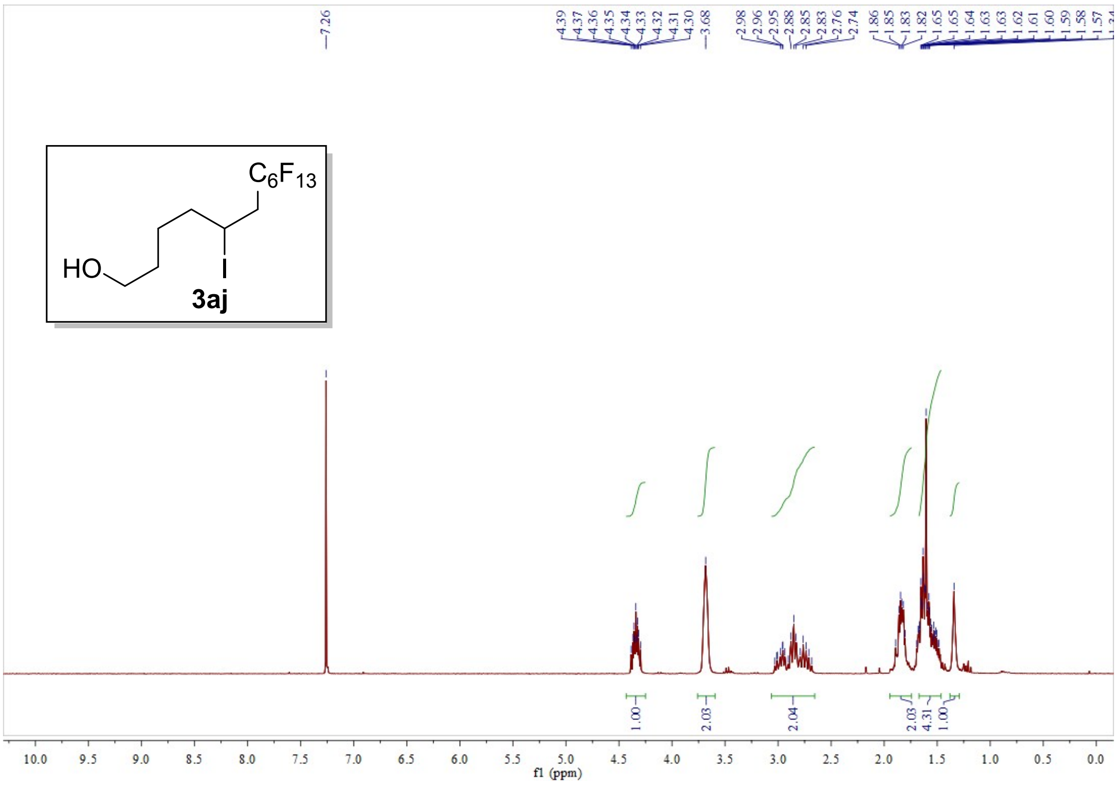

C_6F_{13}
HO
I
3aj
f1 (ppm)

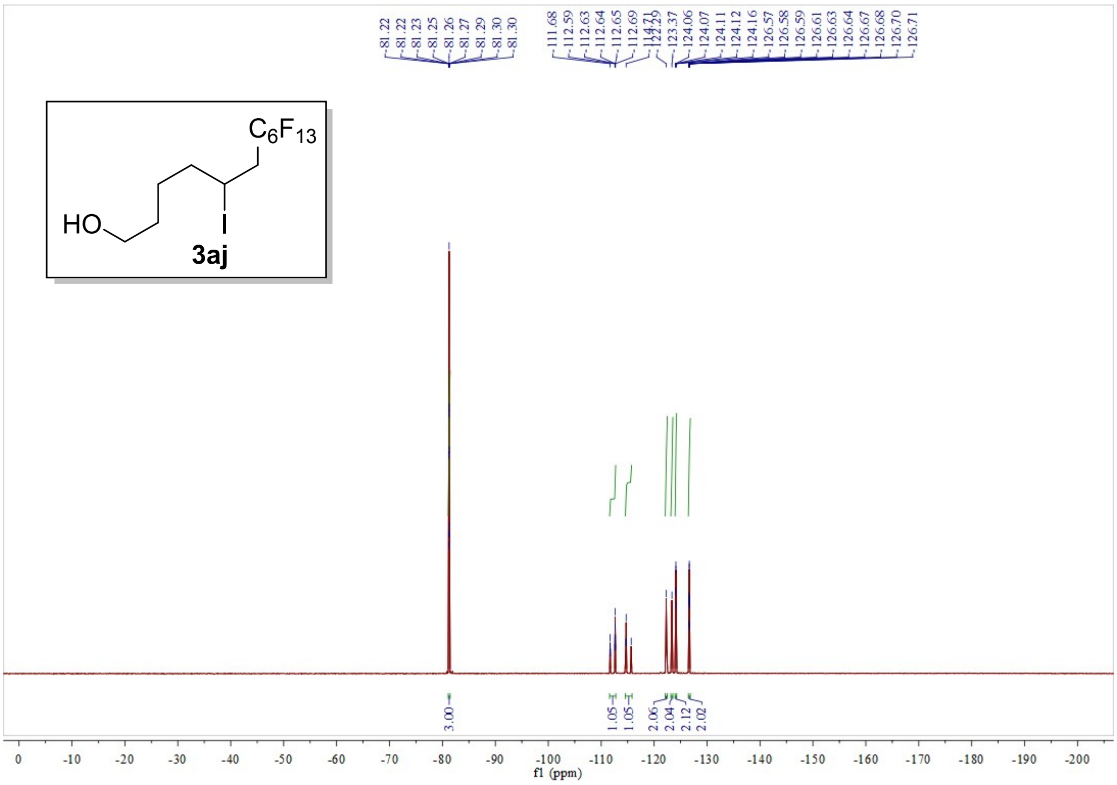

C_6F_{13}
HO
I
3aj
f1 (ppm)

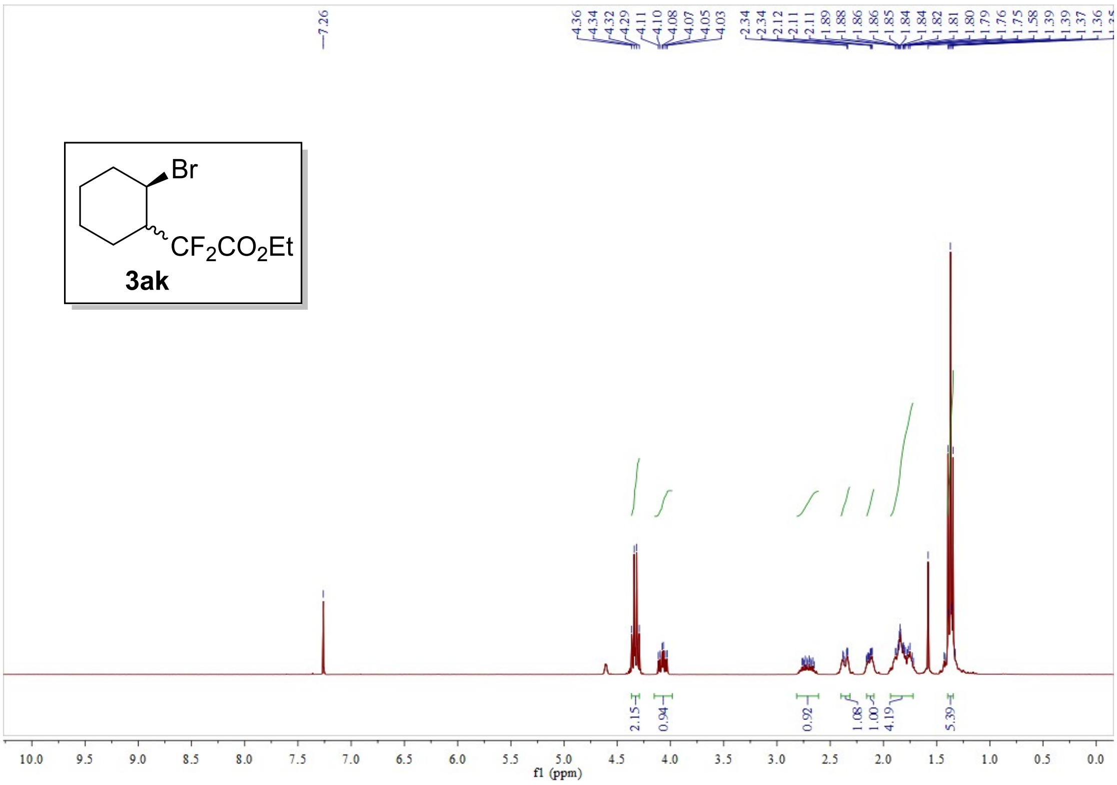

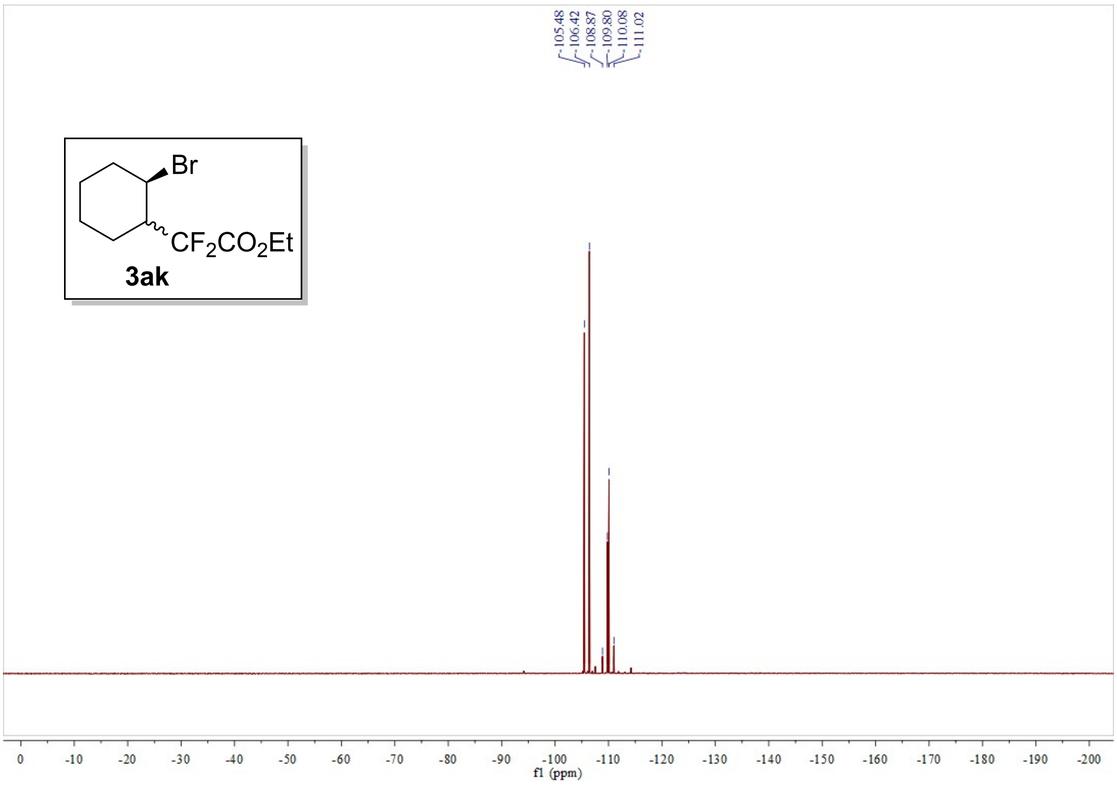

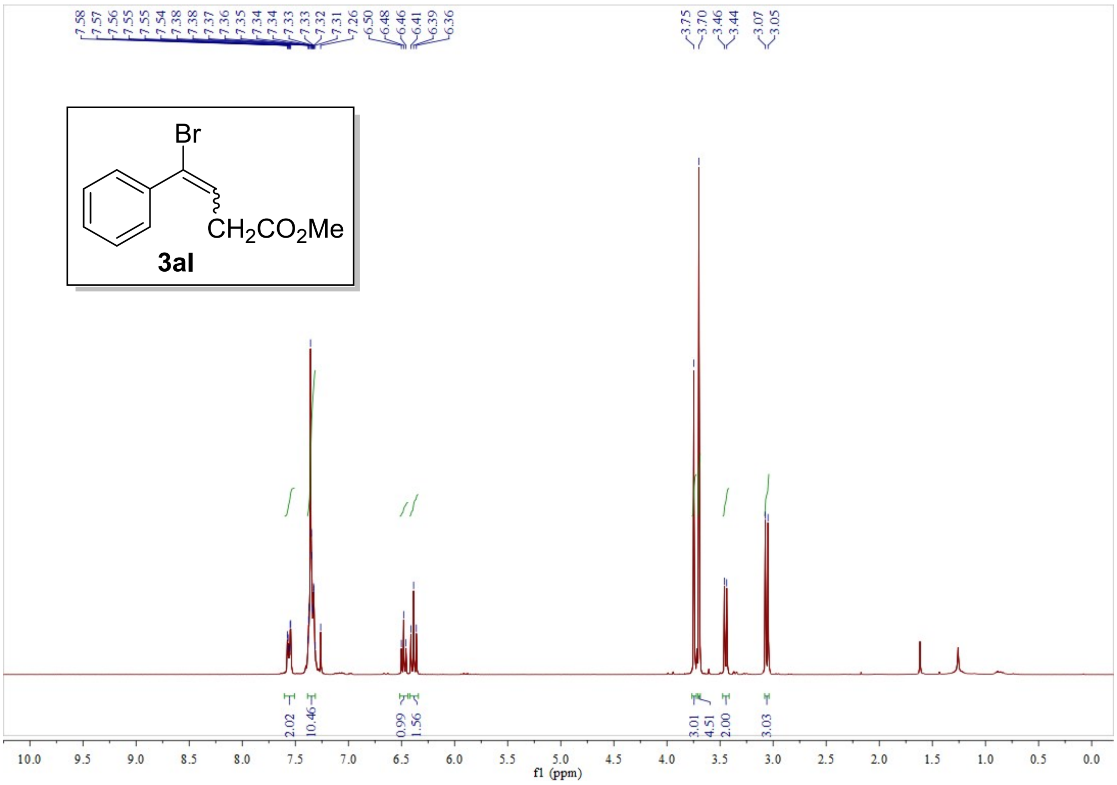
7.58
7.57
7.56
7.55
7.55
7.54
7.38
7.38
7.37
7.36
7.35
7.34
7.34
7.33
7.33
7.32
7.31
7.26
6.50
6.48
6.46
6.41
6.39
6.36
3.75
3.70
3.46
3.44
3.07
3.05
Br
CH_2CO_2Me
3al
2.02
10.46
0.99
1.56
3.01
4.51
2.00
3.03
f1 (ppm)

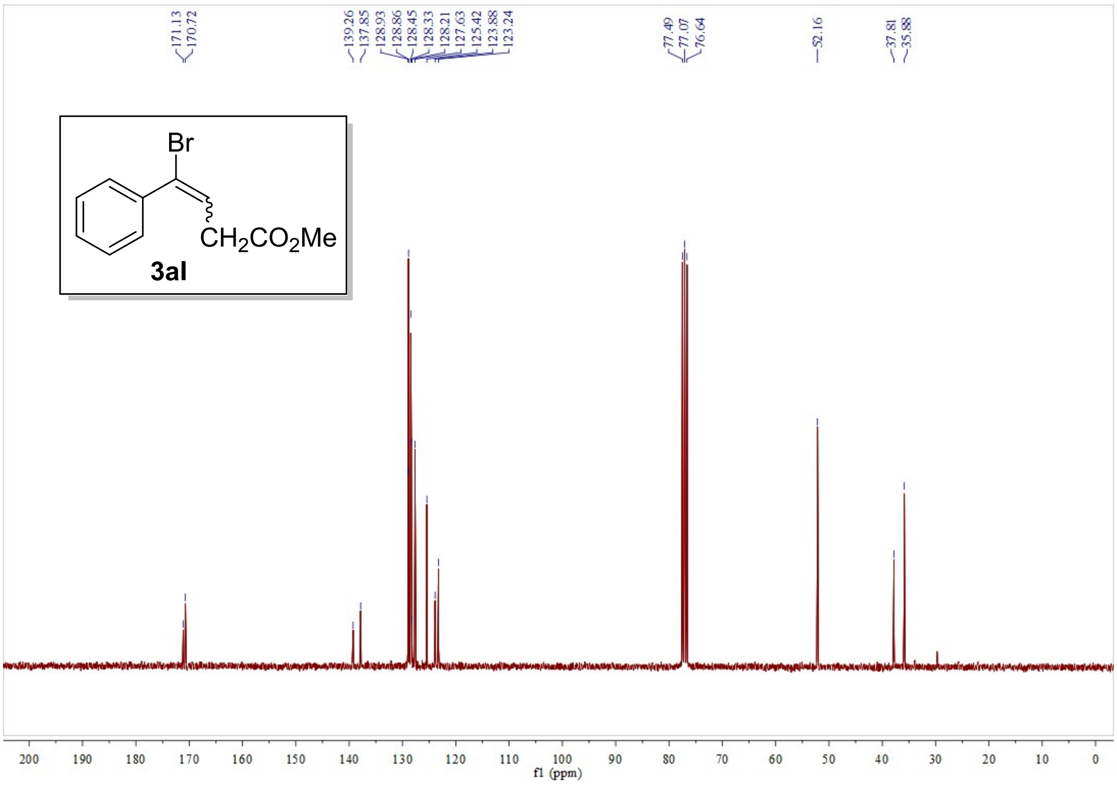
171.13
170.72
139.26
137.85
128.93
128.86
128.45
128.33
128.21
127.63
125.42
123.88
123.24
77.49
77.07
76.64
52.16
37.81
35.88
Br
CH_2CO_2Me
3al
f1 (ppm)

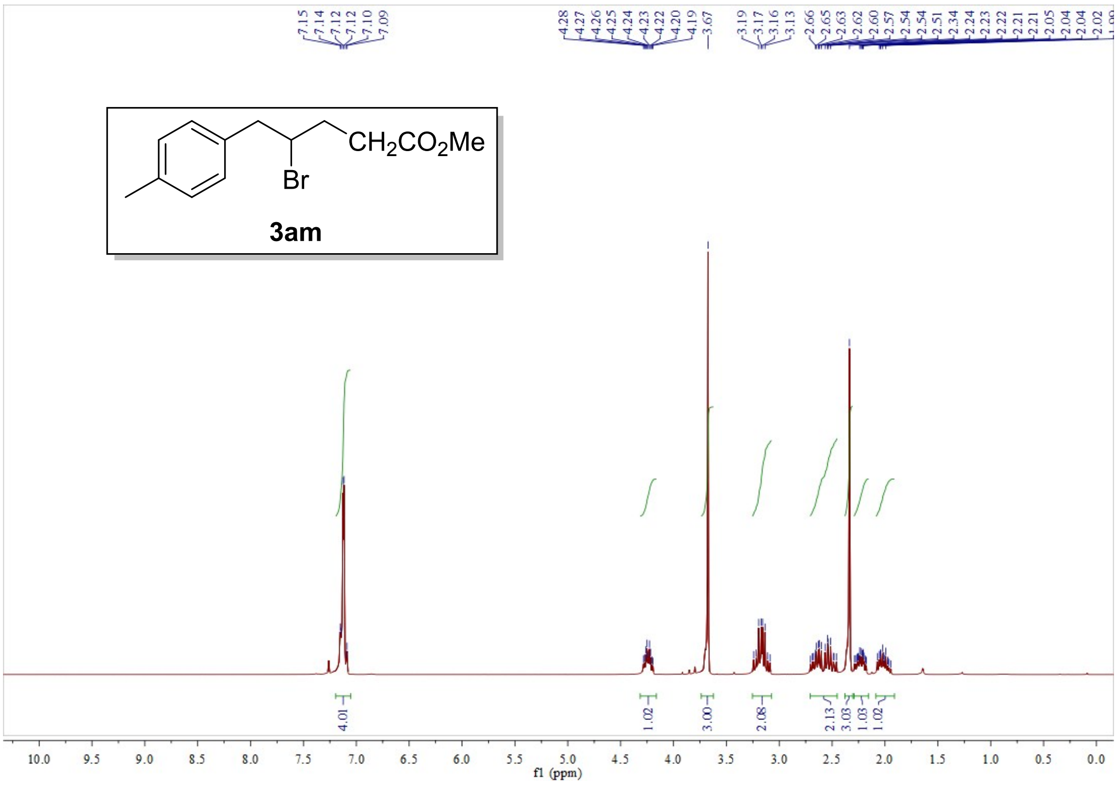
CH2CO2Me
Br
3am
f1 (ppm)

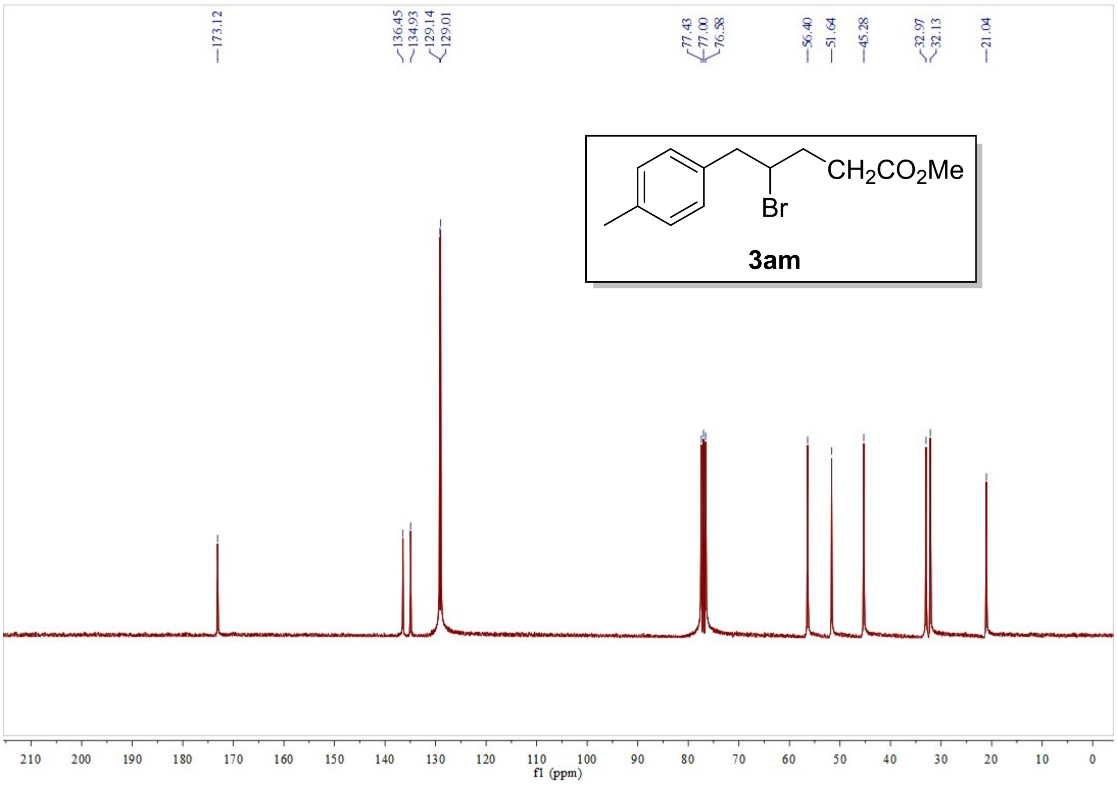
CH2CO2Me
Br
3am
f1 (ppm)

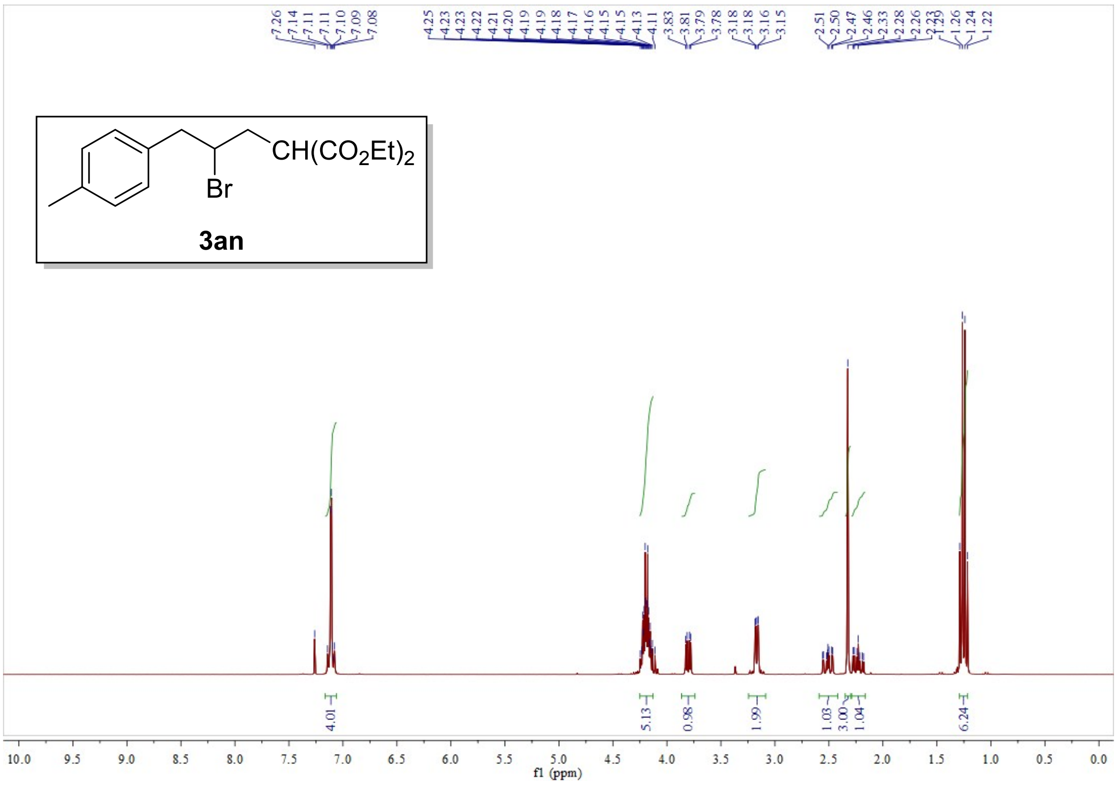
CH(CO2Et)2
Br
3an

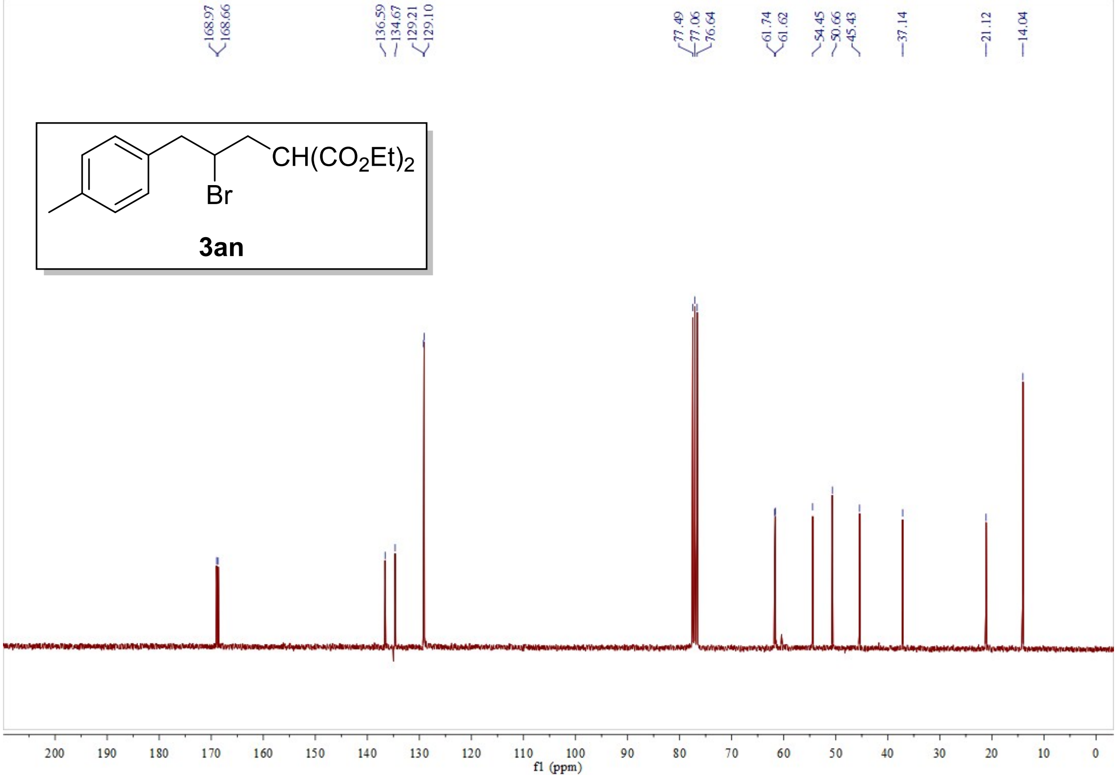
CH(CO2Et)2
Br
3an

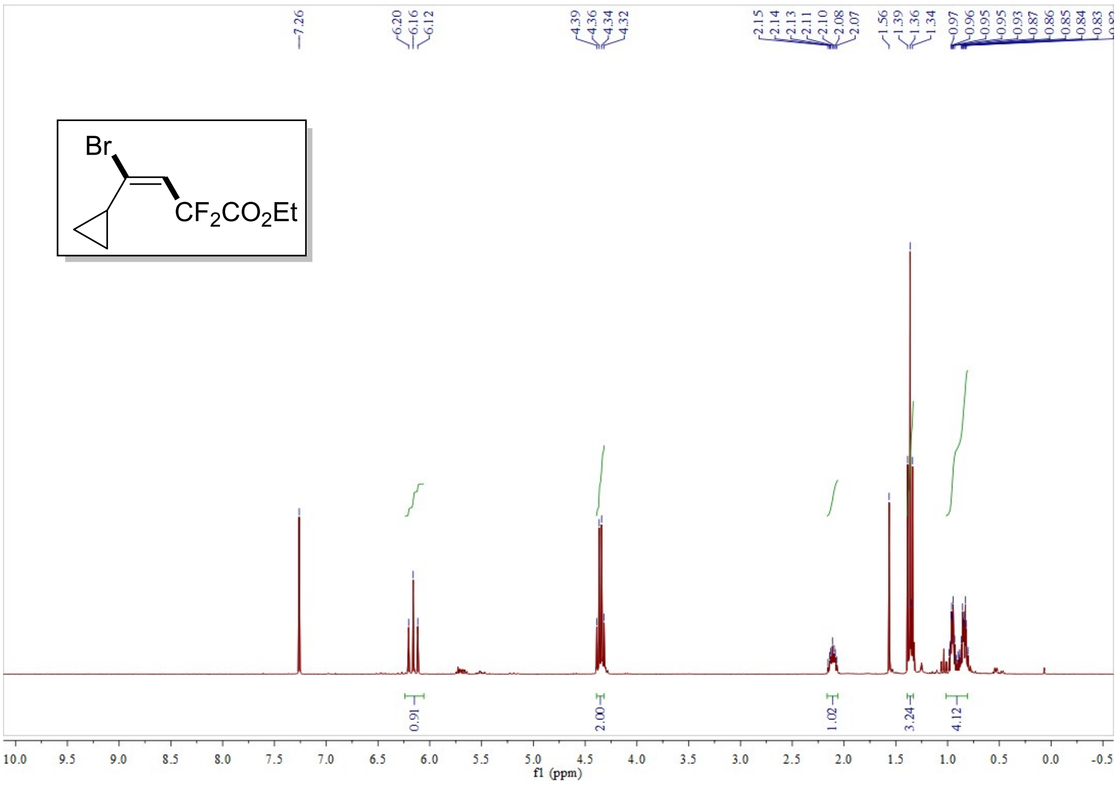
7.26
6.20 6.16 6.12
4.39 4.36 4.34 4.32
2.15 2.14 2.13 2.11 2.10 2.08 2.07
1.56 1.39 1.36 1.34
0.97 0.96 0.95 0.95 0.93 0.87 0.86 0.85 0.84 0.83
Br
CF2CO2Et
0.91
2.00
1.02
3.24
4.12
f1 (ppm)

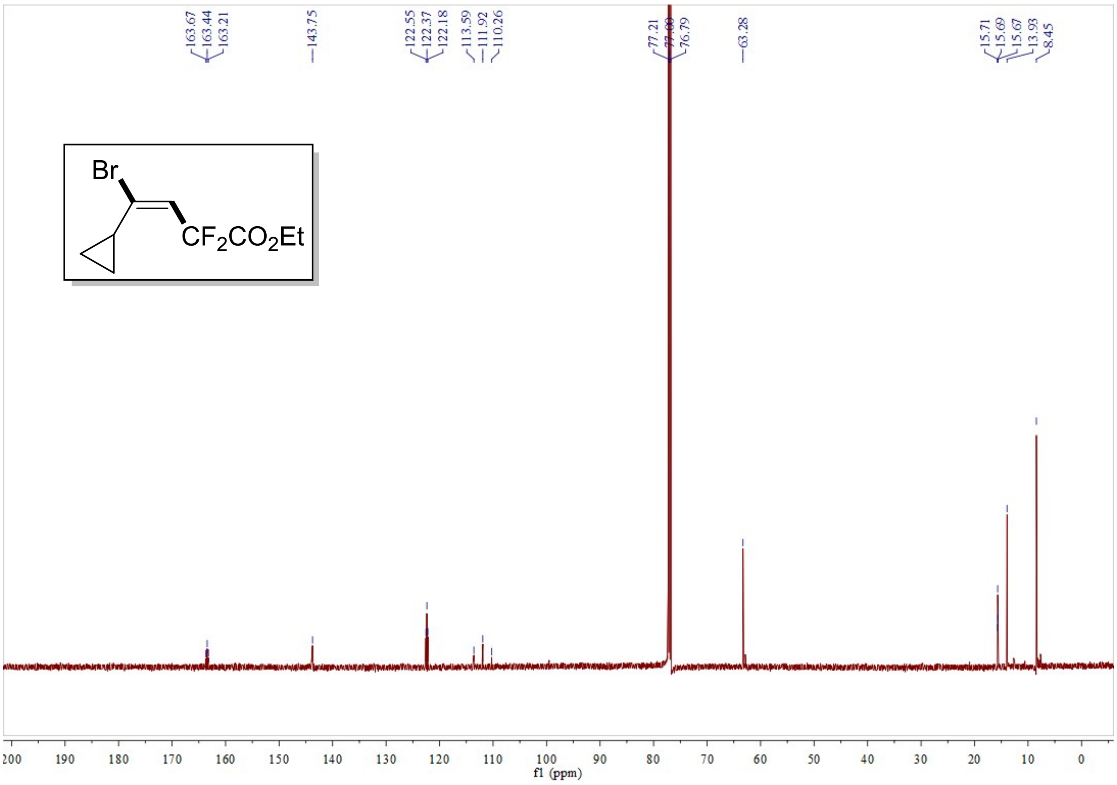
163.67 163.44 163.21
143.75
122.55 122.37 122.18
113.59 111.92 110.26
77.21 76.79
63.28
15.71 15.69 15.67 13.93 8.45
Br
CF2CO2Et
f1 (ppm)

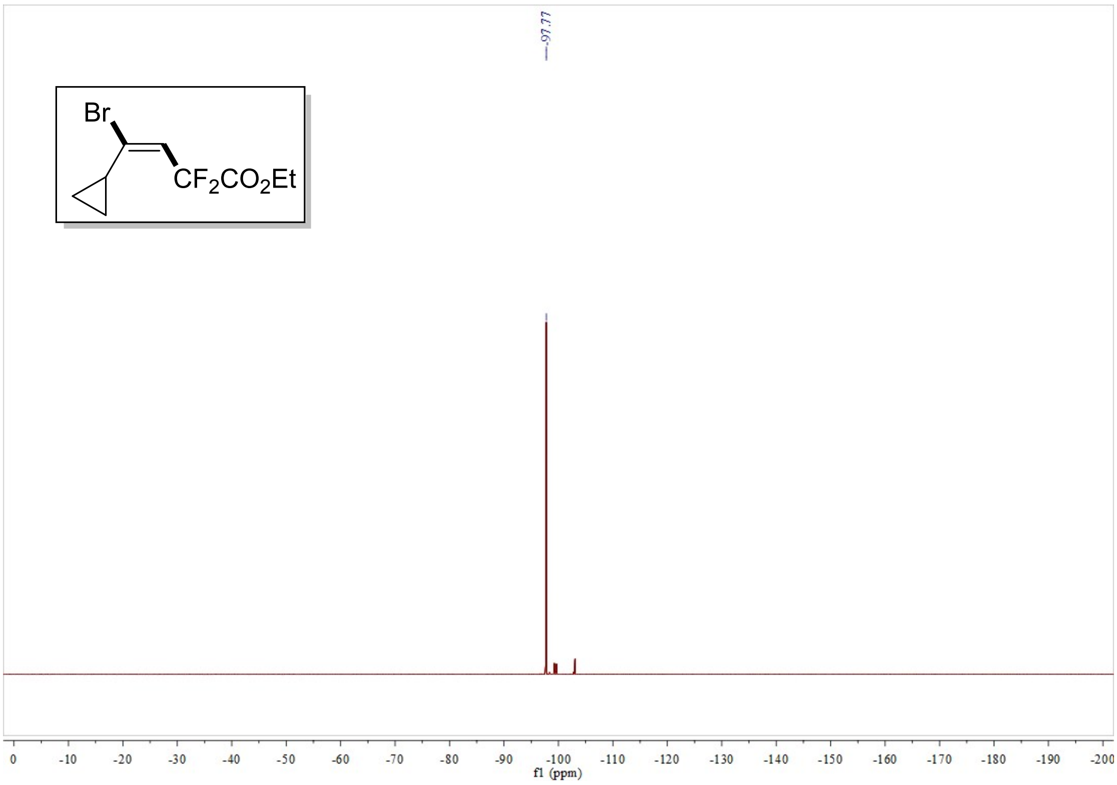
-97.77
Br
CF2CO2Et
f1 (ppm)

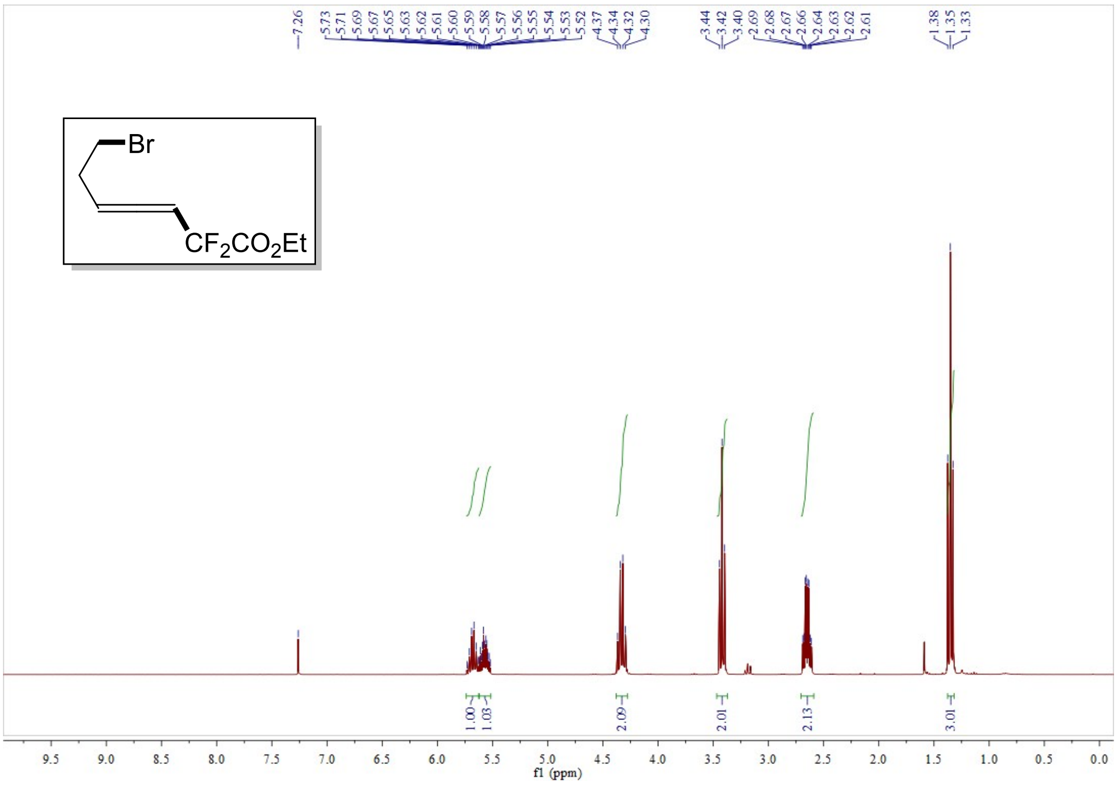
Br
CF2CO2Et
f1 (ppm)

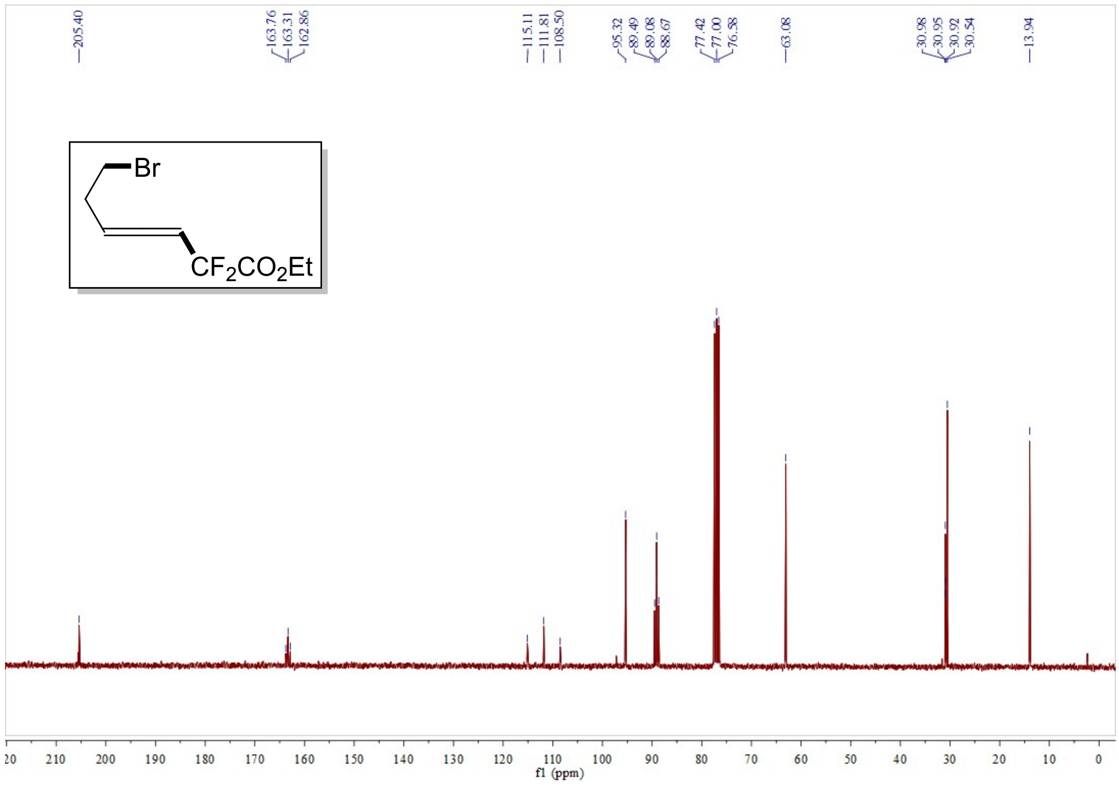

205.40
163.76
163.31
162.86
115.11
111.81
108.50
95.32
89.49
89.08
88.67
77.42
77.00
76.58
63.08
30.98
30.95
30.92
30.54
13.94
Br
CF2CO2Et
fl (ppm)

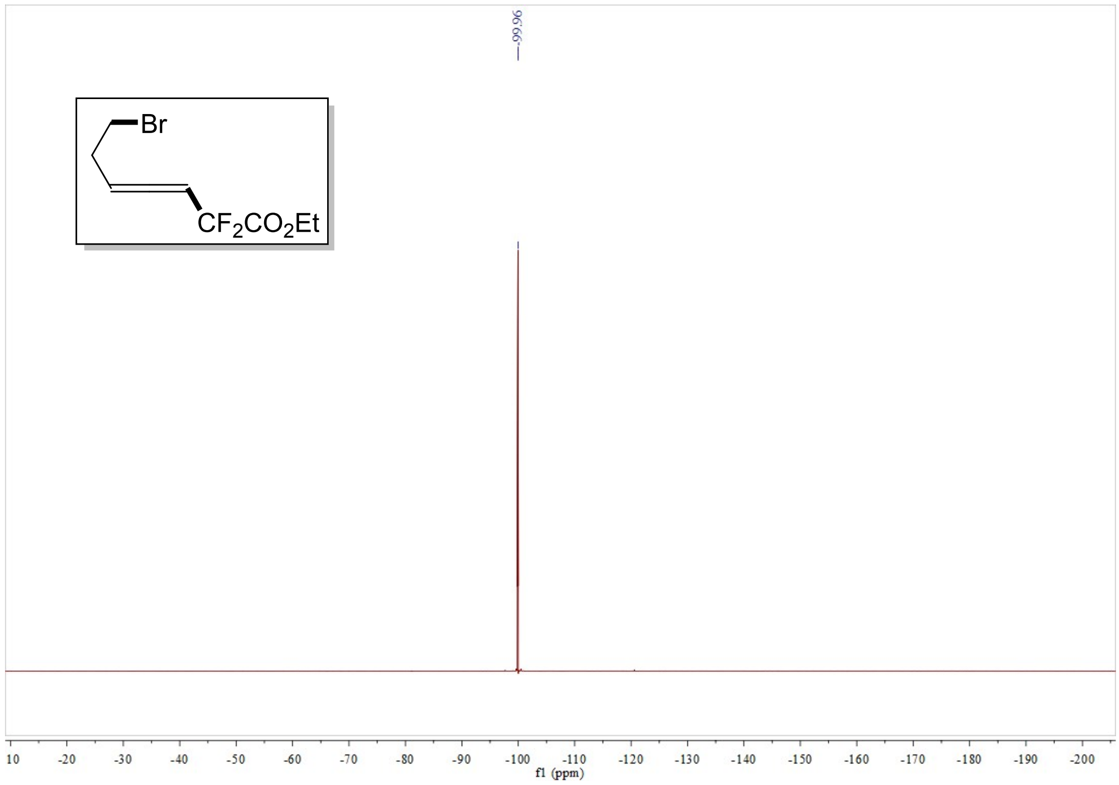

-99.96
Br
CF2CO2Et
fl (ppm)

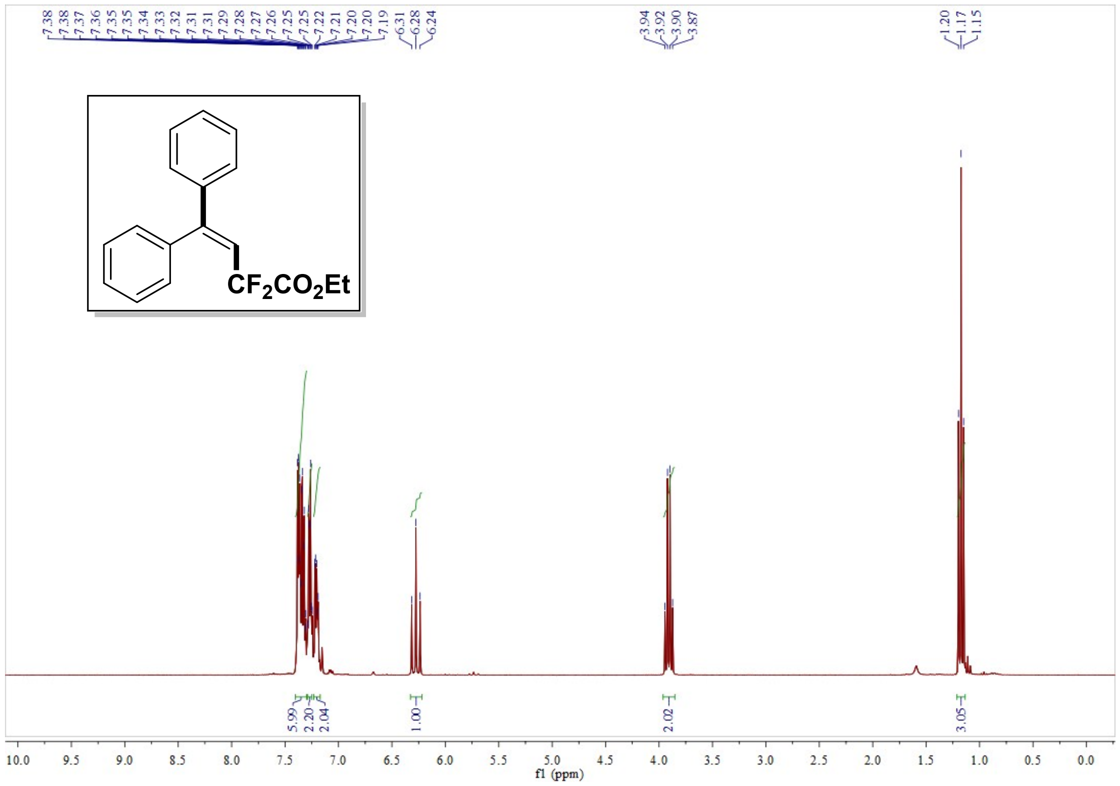
CF2CO2Et
f1 (ppm)

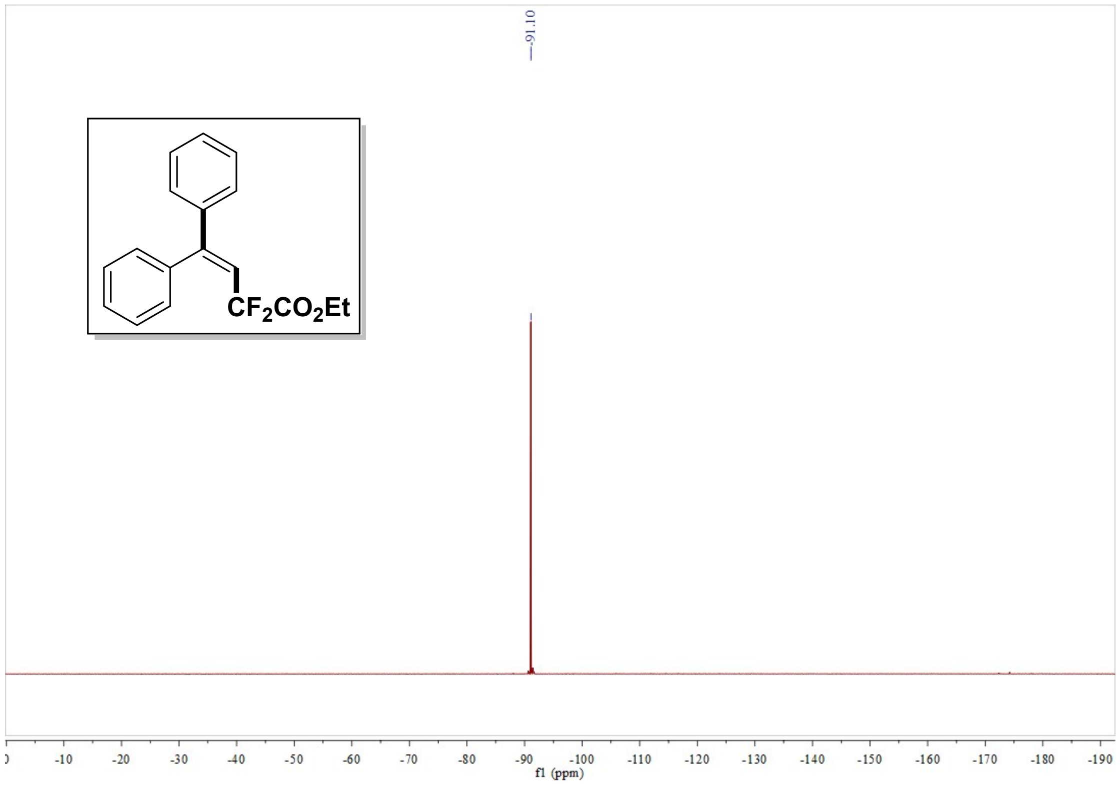
CF2CO2Et
-91.10
f1 (ppm)

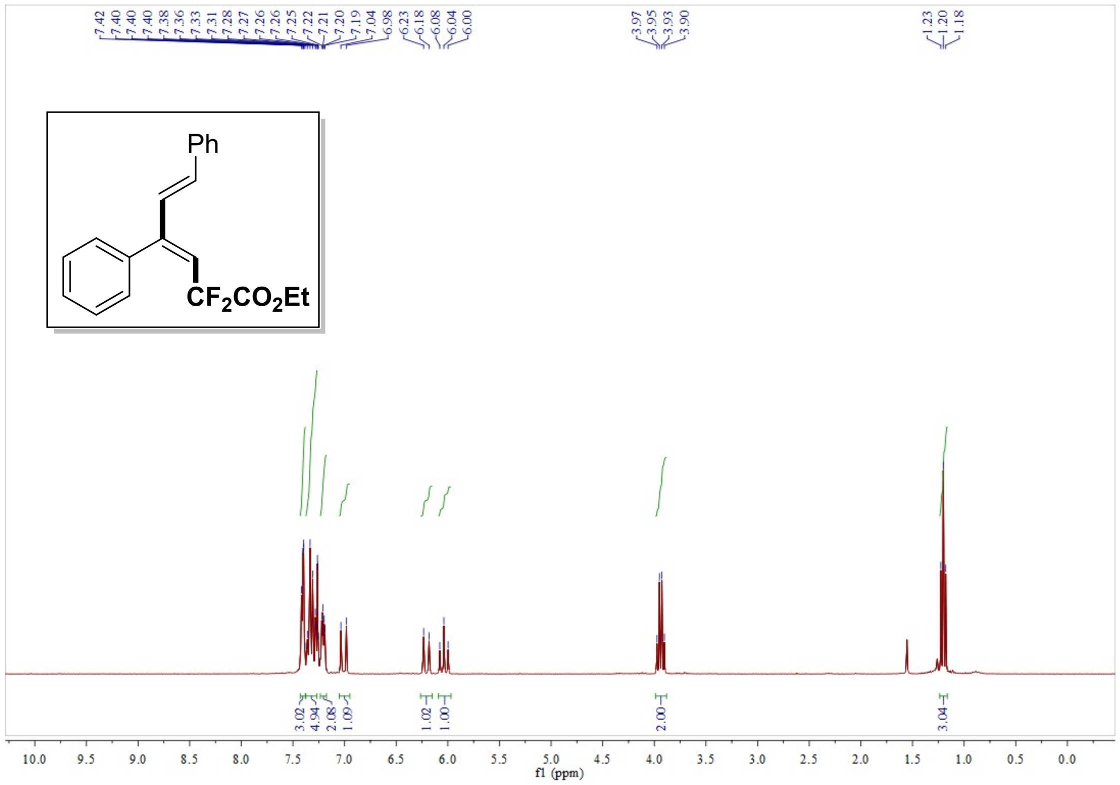

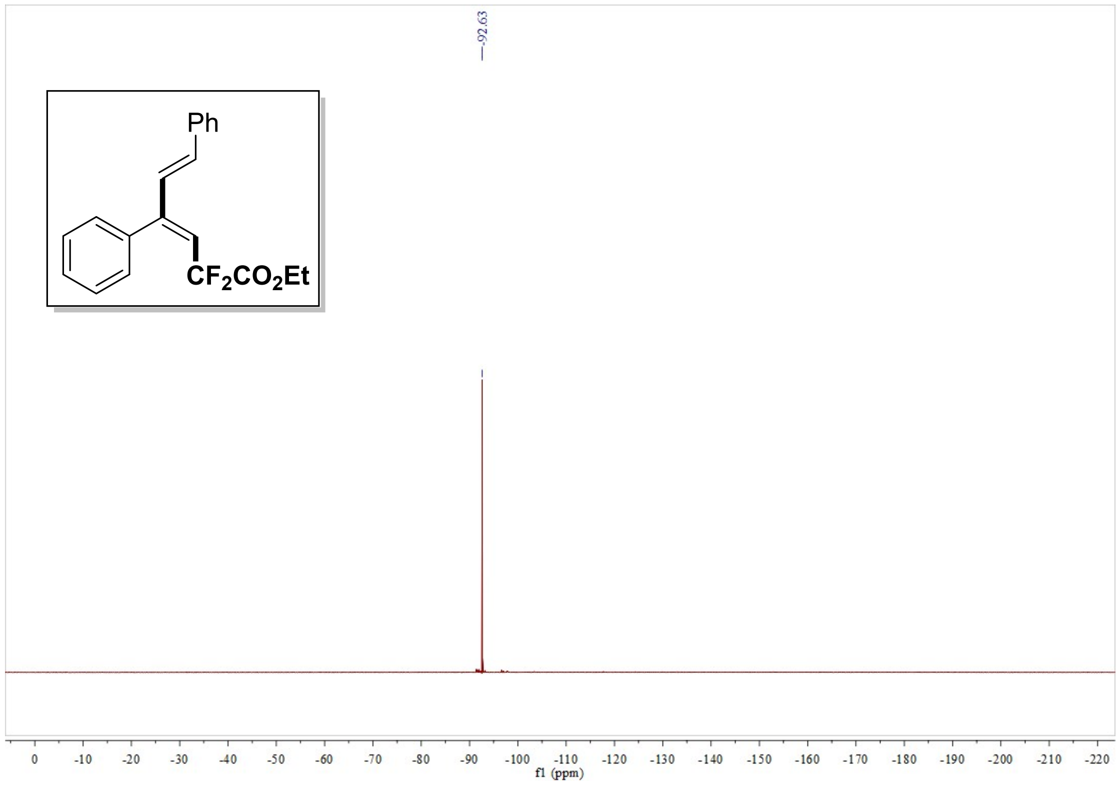

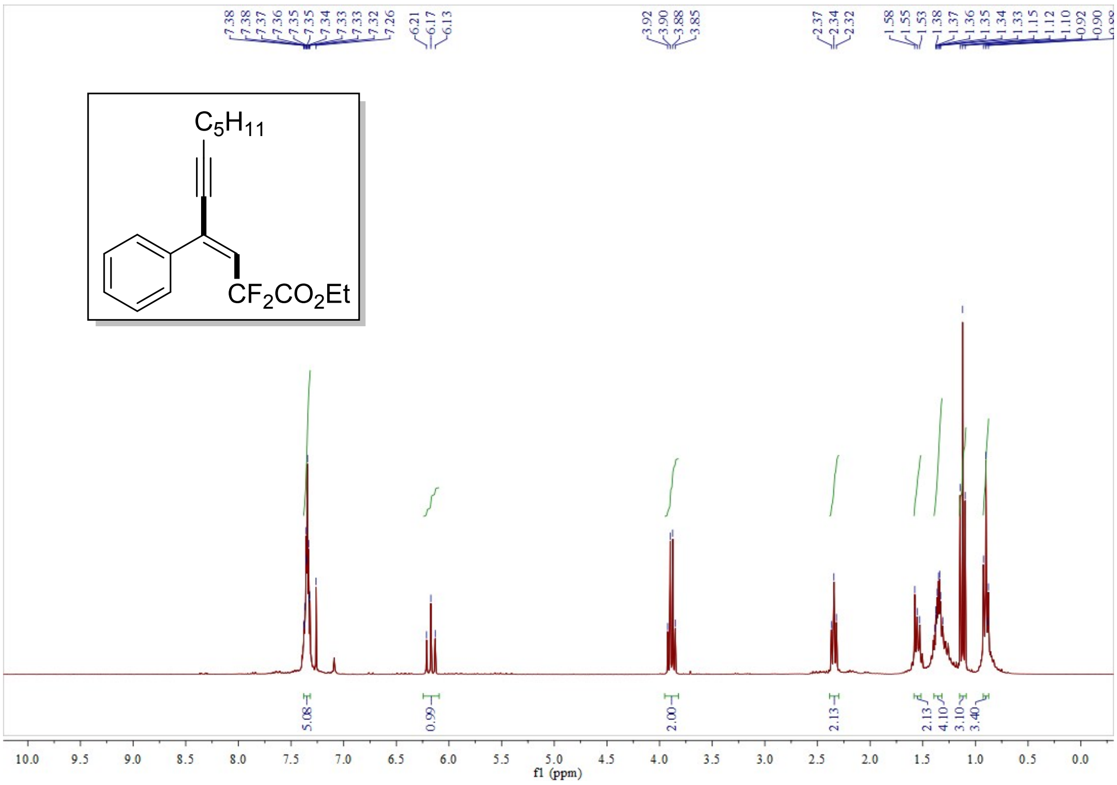

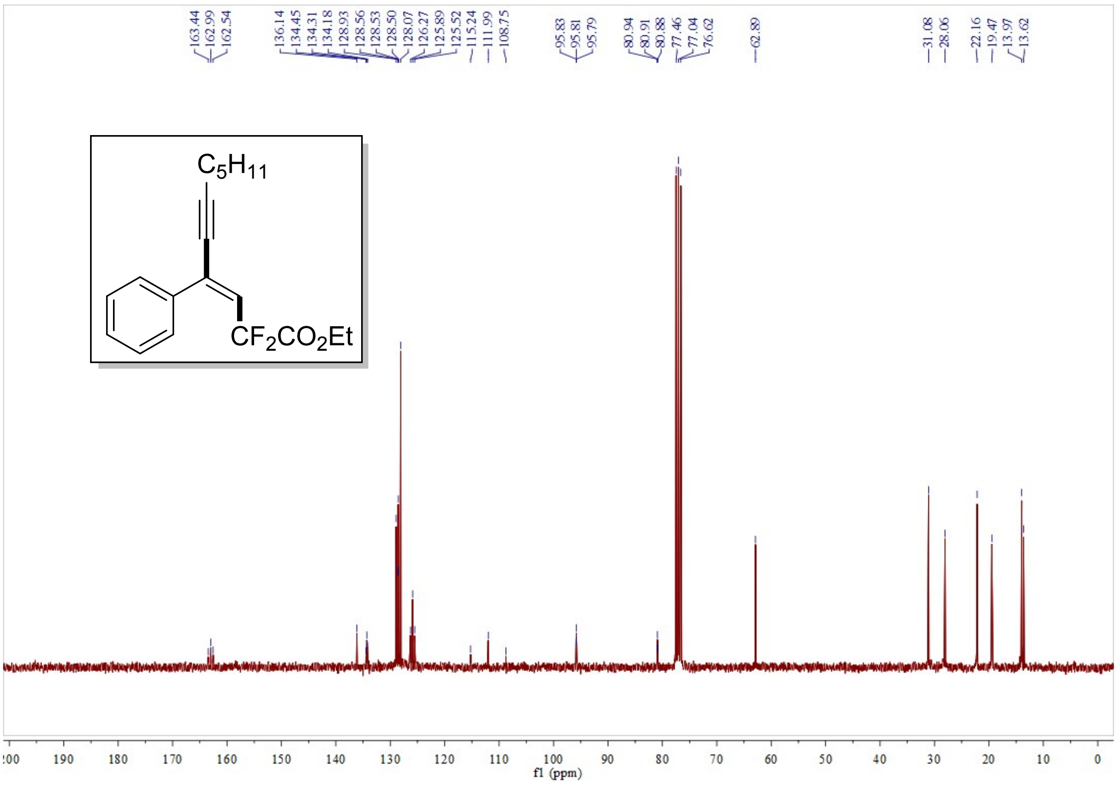

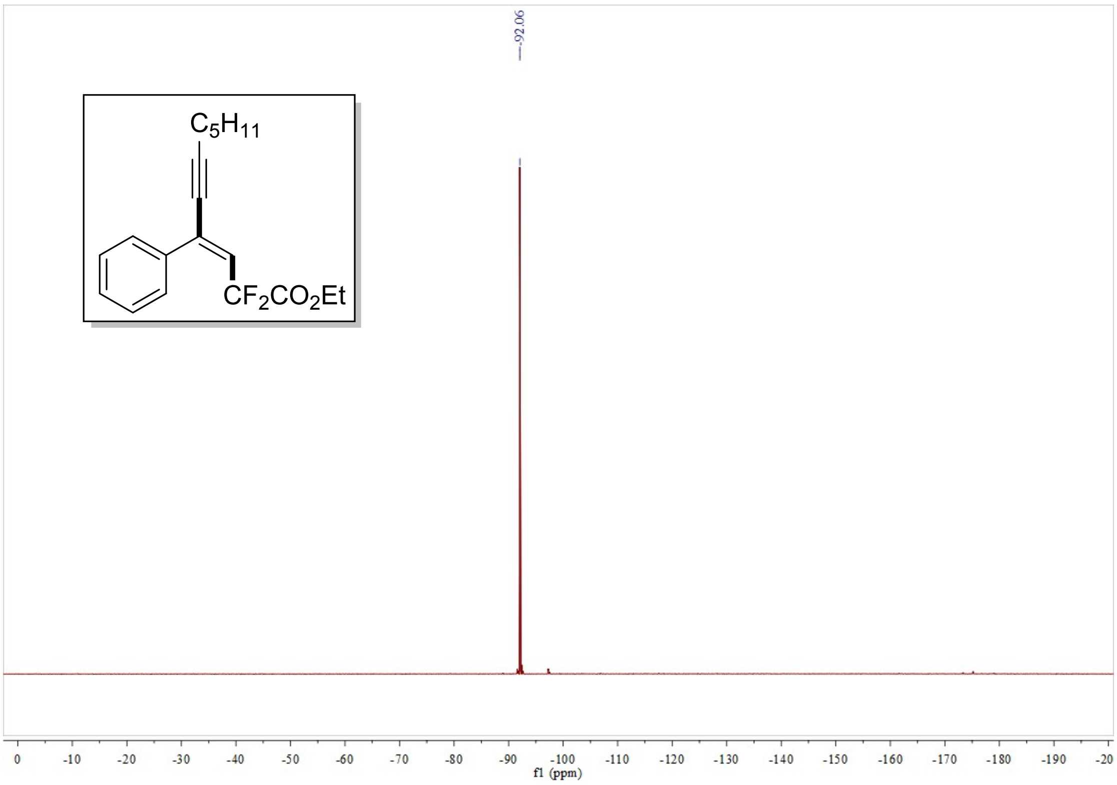
-92.06
C_5H_{11}
CF_2CO_2Et
0 -10 -20 -30 -40 -50 -60 -70 -80 -90 -100 -110 -120 -130 -140 -150 -160 -170 -180 -190 -20
f1 (ppm)
